# Comparison of SIV and HIV-1 Genomic RNA Structures Reveals Impact of Sequence Evolution on Conserved and Non-Conserved Structural Motifs

**DOI:** 10.1371/journal.ppat.1003294

**Published:** 2013-04-04

**Authors:** Elizabeth Pollom, Kristen K. Dang, E. Lake Potter, Robert J. Gorelick, Christina L. Burch, Kevin M. Weeks, Ronald Swanstrom

**Affiliations:** 1 Department of Biochemistry and Biophysics, University of North Carolina at Chapel Hill, Chapel Hill, North Carolina, United States of America; 2 Department of Biomedical Engineering, University of North Carolina at Chapel Hill, Chapel Hill, North Carolina, United States of America; 3 Department of Chemistry, University of North Carolina at Chapel Hill, Chapel Hill, North Carolina, United States of America; 4 AIDS and Cancer Virus Program, SAIC-Frederick, Inc., Frederick National Laboratory for Cancer Research, Frederick, Maryland, United States of America; 5 Department of Biology, University of North Carolina at Chapel Hill, Chapel Hill, North Carolina, United States of America; Duke University Medical Center, United States of America

## Abstract

RNA secondary structure plays a central role in the replication and metabolism of all RNA viruses, including retroviruses like HIV-1. However, structures with known function represent only a fraction of the secondary structure reported for HIV-1_NL4-3_. One tool to assess the importance of RNA structures is to examine their conservation over evolutionary time. To this end, we used SHAPE to model the secondary structure of a second primate lentiviral genome, SIVmac239, which shares only 50% sequence identity at the nucleotide level with HIV-1_NL4-3_. Only about half of the paired nucleotides are paired in both genomic RNAs and, across the genome, just 71 base pairs form with the same pairing partner in both genomes. On average the RNA secondary structure is thus evolving at a much faster rate than the sequence. Structure at the Gag-Pro-Pol frameshift site is maintained but in a significantly altered form, while the impact of selection for maintaining a protein binding interaction can be seen in the conservation of pairing partners in the small RRE stems where Rev binds. Structures that are conserved between SIVmac239 and HIV-1_NL4-3_ also occur at the 5′ polyadenylation sequence, in the plus strand primer sites, PPT and cPPT, and in the stem-loop structure that includes the first splice acceptor site. The two genomes are adenosine-rich and cytidine-poor. The structured regions are enriched in guanosines, while unpaired regions are enriched in adenosines, and functionaly important structures have stronger base pairing than nonconserved structures. We conclude that much of the secondary structure is the result of fortuitous pairing in a metastable state that reforms during sequence evolution. However, secondary structure elements with important function are stabilized by higher guanosine content that allows regions of structure to persist as sequence evolution proceeds, and, within the confines of selective pressure, allows structures to evolve.

## Author Summary

We have taken advantage of the rapid evolution of primate lentiviruses to assess the conservation of secondary structure in the viral RNA genome. We determined the structure of the SIVmac239 RNA genome to allow a detailed comparison with the previously determined structure of the HIV-1_NL4-3_ genome. In comparing the two structures, we find very few conserved base pairs with the same pairing partners, indicating that RNA structure is evolving even faster than the sequence. This suggests that most of the genome is in a metastable state that refolds during sequence evolution. Specific areas of structure that are required for function are maintained by the clustering of guanosines in the otherwise adenosine-rich genome, although the precise organization of the structure evolves. The strong effect of selection on maintainence of protein recognition sites can be seen in the conservation of pairing partners within the Rev binding sites in the RRE RNA. We propose that the more stable elements of RNA structure that are needed for function are susceptible to mutation during viral DNA synthesis. This causes the structures to evolve rapidly, yet still within the constricts of selective pressure, allowing maintenance of function.

## Introduction

RNA secondary structures play fundamental roles in the replication of all positive-strand RNA viruses. Because of their small genomes (which are largely devoted to encoding viral proteins), these viruses use available sequence space highly efficiently. The genomic RNA of viruses forms structures necessary for multiple replicative functions. For example, internal ribosome entry site elements interact with the cellular translation initiation machinery, diverse structural signals direct packaging of viral RNA into viral particles, and RNA structure can provide control signals for differential viral gene expression. The human immunodeficiency virus type 1 (HIV-1) is no exception, and well-characterized RNA structures within the coding domains of the genome play critical roles in regulation of replication. These include a structure in the *env* gene, the Rev Response Element (RRE), that binds the viral protein Rev leading to the transport of unspliced and singly-spliced viral mRNA out of the nucleus [1], [2], and a hairpin structure preceded by a poly(U) slippery sequence that mediates a frameshift during synthesis of the Gag-Pro-Pol polyprotein [3], [4]. The untranslated regions (UTRs) of HIV-1 and simian immunodeficiency virus (SIV) contain the TAR hairpin, which recruits the Tat protein to modulate transcription [5], [6] (reviewed in [7]) and other stem-loop structures that are important for dimer initiation (DIS) [8], splicing [9]–[11], and viral RNA packaging [12]–[14] (reviewed in [15]). Several lines of evidence emphasize that the HIV-1 genome contains extensive additional RNA secondary structures whose functional roles are not yet fully understood [16]–[18].

The structures of large RNAs, like viral RNA genomes, are too complex to be predicted with confidence from first principles or thermodynamic-based algorithms alone. Useful working models can often be obtained when additional information is available to restrain the number of possible secondary structure elements. Two such approaches are to compare evolutionarily related sequences to identify RNA motifs that co-vary to preserve base pairs, and to experimentally probe the RNA structure with chemicals or nucleases to infer the presence of paired versus unpaired regions. In the selective 2′-hydroxyl acylation analyzed by primer extension (SHAPE) chemical probing approach, nucleotide reactivities show a strong inverse correlation with the probability that a nucleotide is base-paired. SHAPE-informed prediction of RNA folding has been used to develop secondary structure models for diverse RNAs [19]–[23] including the full-length genomic RNA structure of HIV-1_NL4-3_ [17]. This HIV-1_NL4-3_ model shows a very strong correlation between regions that can be targeted by siRNAs to inhibit viral replication [18] and regions that are predicted to be single-stranded, suggesting that many global structural features are likely correct.

One approach to evaluating the broader significance of these structures is to examine the conservation of these structures in a related virus. To this end, we analyzed the secondary structure of the genomic RNA of a second primate lentivirus, SIVmac239, a representative of the SIVsm/HIV-2 lineage of primate lentiviruses. HIV-2 evolved from a different primate reservoir than HIV-1. HIV-2 arose from an SIV that infects the sooty mangabey (*Cercocebus atys*), and SIVsm has also infected rhesus macaques in primate centers to cause an AIDS-like illness. SIVmac239 [24] now serves as a prototype reference sequence for comparative analysis of the HIV-2/SIVsm lineage [25]. SIVmac239 has a larger evolutionary distance from HIV-1_NL4-3_ than the more similar SIVcpz or other HIV-1 clades, and conservation of structures between SIVmac239 and HIV-1_NL4-3_ represents an especially stringent test for functional relevance. In this analysis, we describe areas where RNA structure is maintained between SIVmac239 and HIV-1_NL4-3_, where it is divergent, and outline possible mechanisms for understanding the interplay between rapid sequence evolution and maintenance of function of RNA structural motifs.

## Results and Discussion

### Features of the SIVmac239 RNA structure

**Figure 1:**
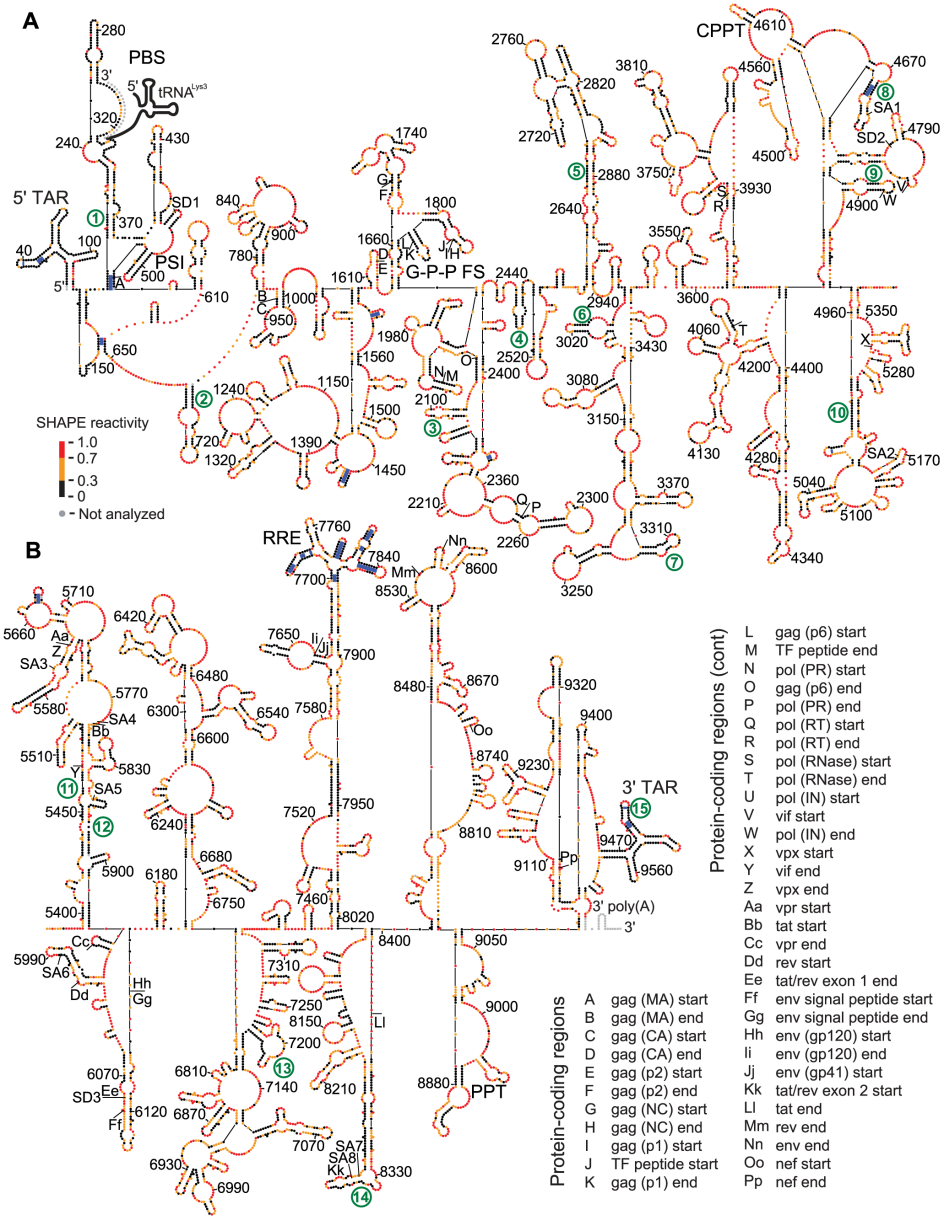
Model for the structure of the SIVmac239 RNA genome as determined by SHAPE probing and directed RNA structure refinement. The genome is divided into (A) 5′ and (B) 3′ halves. Colors of nucleotides indicate SHAPE reactivity on the scale shown on the left. Each sphere corresponds to a nucleotide, and side-by-side spheres indicate a base pair. Protein coding region boundaries are indicated by letters with the code shown at the bottom. Splice acceptor and donor sites [49] are labeled SA and SD, respectively. tRNA^Lys3^ interaction is shown in gray. Heavy blue bars indicate base pairs in stems that are conserved between codon-aligned SIVmac239 and HIV-1_NL4-3_ RNA structures (71 total pairs). Areas of structure with a median reactivity below 0.3 over a 75 nucleotide window are numbered in green and correspond to motif numbers in Figure 2B. All positions are numbered in reference to the GenBank accession number M33262 for SIVmac239. A full structure, including nucleotide identity, is shown in Figure S1. Helix files for SIVmac239 and HIV-1_NL4-3_ are included in Datasets S1 and S3, respectively.

To develop an experimentally based secondary structure model for the genomic RNA structure of SIVmac239 (GenBank accession M33262), we used a strategy similar to that used to develop a model for the secondary structure of genomic HIV-1_NL4-3_ RNA [17]. Viral RNA was purified from SIVmac239 particles and derivatized with the SHAPE reagent 1-methyl-7-nitroisatoic anhydride (1M7) under physiologically relevant conditions to discriminate between single-stranded (generally reactive) positions versus nucleotides constrained by base pairing or other interactions (and therefore unreactive) [21], [26]–[28]. The derivatized positions were identified as terminations to DNA synthesis by reverse transcriptase (primers listed in Table S1). SHAPE reactivities were measured for 9,605 nucleotides, 99.6% of the genome. These data were used as pseudo-free energy change constraints to constrain RNA secondary structure prediction. In the secondary structure model for the SIVmac239 RNA genome (Figure 1 and Figure S1), 4,970 nucleotides were predicted to be base-paired (51.5%), whereas 4,676 nucleotides were modeled as single-stranded (48.5%).

**Figure 2:**
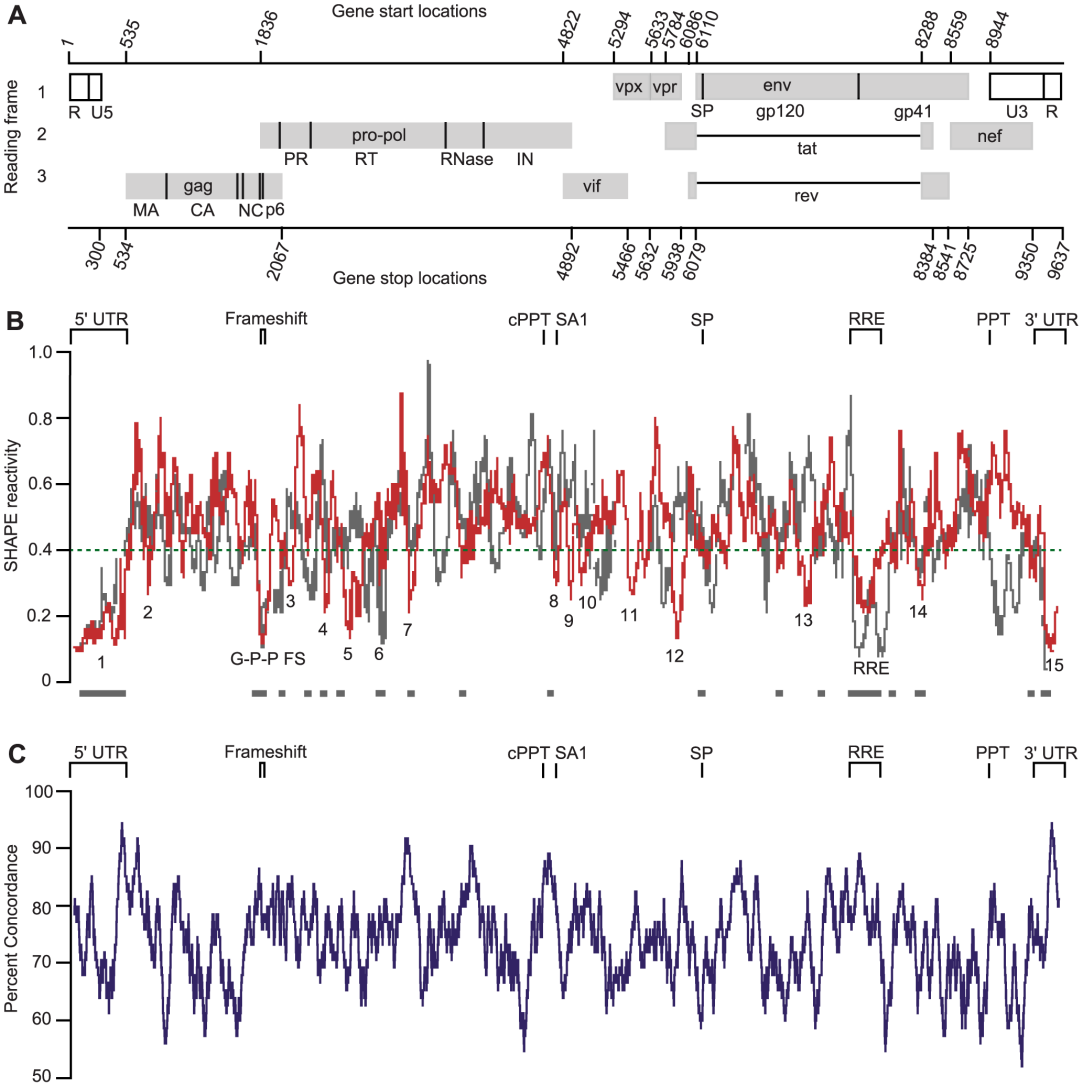
Genomic organization and SHAPE reactivity of SIVmac239 and comparison with HIV-1_NL4-3_. (A) Organization of the SIVmac239 genome. Gray boxes indicate protein coding regions, dark lines indicate the boundaries of the mature viral proteins. (B) SIVmac239 median SHAPE reactivity values calculated over a 75 nucleotide sliding window (red). Regions with SHAPE reactivities below 0.3 are numbered (and listed explicity in Table S2). SHAPE reactivity values of HIV-1_NL4-3_ (gray) are shown as medians calculated over a 75 nucleotide sliding window and overlayed with those of SIVmac239. Viruses were codon-aligned based on the Los Alamos Database alignment (www.hiv.lanl.gov). Green dashed line indicates SHAPE reactivity of 0.4, and the gray bars below indicate regions where median reactivity values for both viruses are below 0.4. SHAPE reactivities for SIVmac239 and HIV-1_NL4-3_ are included in Datasets S2 and S4, respectively. (C) Percent concordance of SHAPE reactivity and pairing prediction of the SIVmac239 genome over a 76 nucleotide sliding window. A concordant nucleotide is defined as having low SHAPE reactivity (below 0.4) and predicted to be paired or high reactivity (0.4 or above) and predicted to be unpaired.

Highly structured regions in an RNA can be inferred in a model-free way by identifying regions with low overall median SHAPE reactivities. Many areas of the SIVmac239 RNA genome have low median SHAPE reactivity (defined as less than 0.4 on a scale from 0 to ∼1.5) over a 75 nucleotide window, and these correspond to regions of structure with both known and unknown function. To isolate regions of low reactivity to a more select group, we assigned numerical values to the areas that have median SHAPE reactivities below 0.3 (labeled in Figures 1 and 2). The lowest median SHAPE reactivity values occurred at the 5′ and 3′ ends of the genome. The highly structured 5′ region extends until nucleotide 539 (Figures 1A and 2B, motif 1), and the structured 3′ region begins at position 9462 at the start of the 3′ TAR structure within the terminal repeat (R) regions (Figures 1B and 2B, motif 15). In addition, the Gag-Pro-Pol frameshift (G-P-P FS) element and the Rev Response Element (RRE) are highly structured (Figures 1B and 2B). Similarly, when we used RNA Decoder (a program that predicts evolutionarily conserved RNA secondary structure in the context of the protein-coding sequence of an RNA [29]) with an HIV-2/SIVsm sequence dataset to infer conserved regions of secondary structure, we also found that the 5′ and 3′ UTRs and the RRE showed the strongest signal for conservation of structure (Figure S2). Using the RNA Decoder approach we conclude that some features of secondary structure are slightly conserved within the coding region, but none at the level of the RRE. Other regions have low median SHAPE reactivities, yet currently unknown RNA functions (Figures 1 and 2B, motifs 2–14).

The secondary structure profile presented here is likely not the only structure that is possible for the SIVmac239 RNA to form. Instead, it is the lowest free energy structure predicted, given the SHAPE reactivity constraints. The potential for alternative or multiple structures can be partially inferred in the reactive nucleotides that are predicted to be paired and the unreactive nucleotides that are predicted to be single-stranded (Figure 1). Even so, over a 75 nucleotide window, the fraction of nucleotides whose pairing prediction is in concordance with the SHAPE reactivity (assuming nucleotides with SHAPE reactivity values below 0.4 are likely to be paired or those with reactivity values equal to or above 0.4 are preferentially unpaired) averaged 74% across the entire SIVmac239 genome (Figure 2C), with certain regions of the genome (including the 5′-UTR, the Gag-Pro-Pol frameshift, the RRE, the first splice acceptor, and the polypurine tracts) having an even higher concordance. It is possible that some regions fold to unique structure while others exist in multiple structures, with the SHAPE-informed structure being one of them and the reactivity representing the average of two or more states. In the following sections, we examine structure in regions with low SHAPE reactivity and high pairing concordance to infer biological importance based on their conservation with HIV-1_NL4-3_, first on a global scale and then in detail.

### Overview of base pairing within the SIVmac239 and HIV-1_NL4-3_ RNA genomes

SIVmac239 is the second full-length genomic RNA of a primate lentivirus to be evaluated by SHAPE-directed modeling; the first was that of HIV-1_NL4-3_ [17]. Comparison of the structural models of these two distantly related retroviral RNA genomes should reveal conserved structural elements. Visually, the patterns of 1M7 reactivity in the 5′ noncoding region, the Gag-Pro-Pol frameshift site, and the RRE — all regions with well-established conserved functions — are similar for SIVmac239 and HIV-1_NL4-3_ RNAs (Figure 2B). A bootstrapping analysis (see Methods) showed that the measured SHAPE profiles across both genomes were significantly more similar than expected by chance (10,000 trials, p<0.0001). Thus, in a broad view, there appears to be a propensity to conserve the overall level of local RNA structure across similar regions for these two genomes.

The RNA folding algorithm employed for structure prediction includes a pseudo-free energy change term to account for the SHAPE reactivity (Text S1). Newly optimized parameters for calculating the pseudo-free energy term [30] were used to predict the current SIVmac239 structural model and to revise the secondary structure model for HIV-1_NL4-3_ based on the original reactivity data [17] (Figure S3). These new parameters resulted in changes in the organization of some structures, especially those with higher average SHAPE reactivity, but the locations of highly structured regions did not change significantly. We compared the codon-aligned sequences of SIVmac239 and HIV-1_NL4-3_ for equivalency in terms of base pairing; these two genomes share only 50% identity at the nucleotide level. We found that roughly half of the nucleotides predicted to be base-paired in the HIV-1_NL4-3_ sequence were also paired in the SIVmac239 sequence; conversely, only half of the nucleotides predicted to be single-stranded in the HIV-1_NL4-3_ sequence were also single-stranded in the SIVmac239 sequence (Table 1). In spite of the limited conservation of paired bases, we did observe numerous regions in similar locations in the two genomes with low SHAPE reactivity (defined as median reactivity below 0.4 over a 75 nucleotide window). These areas (Figure 2B, gray dashes) largely fold into structures of unknown function. None of these structures of unknown function have conserved base pairs, and in only a few examples are there even small hairpins that share pairing partners within 40 nucleotides in both alignments.

**Table 1:** Comparison between SIVmac239 and HIV-1_NL4-3_ RNA genome secondary structure models.

	Codon- Aligned Bases	Base-Paired	Base-Paired In Both HIV and SIV	Base-Paired With Same Partner	Base-Paired With Similar Partner (w/in 40 nts.)	Single-Stranded	Single-Stranded In Both HIV and SIV
SIV	8490	4970	2421	142	432	4671	2420
HIV	8490	4500	2421	142	432	4672	2420

SHAPE-directed folding used Δ*G*_SHAPE_ parameters of m = 1.9 and b = −0.7.

As a more stringent definition of structural conservation, we tallied the number of base pairs in both genomes where both nucleotide positions of the base-pairing partners were maintained, using the protein codon sequence to establish the alignment. Only 58 codon-aligned base pairs were fully conserved between the two genomes within the 8,738 nucleotides of the SIVmac239 coding region; an additional 13 base pairs were conserved in the 5′-UTRs. Overall, only 71 base pairs, 3% of the total base pairs, were precisely conserved in these two primate lentiviral genomes (Figure 1, blue bars in paired regions). Overall, regions that share a low SHAPE reactivity are areas of base pairing in both viruses, but the exact structures are not conserved between the two genomes.

### Conserved structure in the 5′-UTR

**Figure 3:**
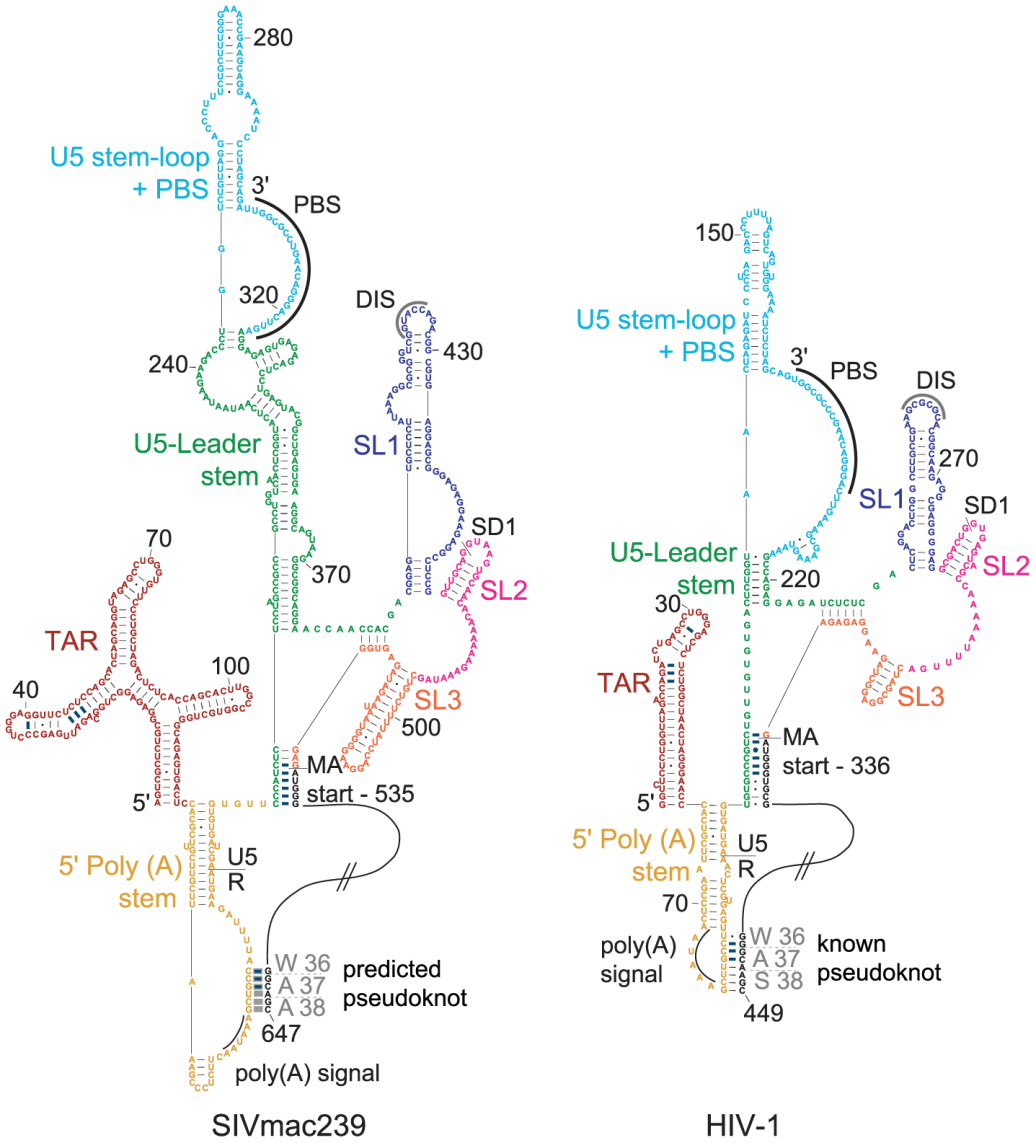
Structural similarity in the 5′ regions of the SIVmac239 and HIV-1_NL4-3_ genomes. Predicted structures of SIVmac239 (left) and HIV-1_NL4-3_ (right) in the 5′ region; distinctive structures are coded by color. Conserved base pairs are indicated by dark blue connecting lines. The primer binding site (PBS) and 5′ poly(A) signal AAUAAA are emphasized with curved lines. The hatched lines indicate nucleotides within the MA coding domain that are not shown. The known pseudoknot is labeled in HIV-1_NL4-3_, and the predicted pseudoknot in SIVmac239 is labeled and shown with thick gray lines; protein residues encoded by the pseudoknot region are shown.

We aligned the 5′-UTRs of each virus both using the structures as predicted by SHAPE-constrained modeling and by identifying the positions of the functionally conserved TAR region, 5′ polyadenylation [poly(A)] signal, primer binding site (PBS), major splice donor (SD1) sequence, dimerization initiation sequence (DIS), and the Psi packaging element (Figure 3). Each of these functional elements folds into a similar structure in both viral RNA genomes even though only 13 of the ∼180 predicted base pairs have the same two pairing partners in both 5′-UTR regions (Figure 3, emphasized with heavy blue lines to indicate conserved pairing partners). Additional conserved structures include a stem immediately 5′ of the PBS, and the SL1 (DIS), SL2 (SD1), and SL3 helices. The stem containing the *gag* start site (Figure 3, MA start), termed U5-AUG and previously identified by phylogenetic analysis and structure probing [21], [31], [32], accounts for six of the identical pairing partners between SIVmac239 and HIV-1_NL4-3_. This interaction has also recently been visualized by NMR analysis of the dimerized and packaged form of HIV-1 RNA [33]. There are structural differences in the TAR motif, which features three stem-loops in SIVmac239 but only a single stem in HIV-1, as previously described [34]–[36]. Notably, the stems in SIVmac239 5′-UTR are generally longer with more base pairs than the equivalent structures in HIV-1_NL4-3_ (Figure 3). This has also been shown for HIV-2 leader RNA in an analysis of the first 560 nucleotides [37].

Because of the sequence redundancies at each end of the retroviral genome, a poly(A) signal (AAUAAA) occurs at each end. The virus prevents use of the signal at the 5′ end to avoid producing truncated RNA transcripts. One way this is accomplished is through a stable hairpin directly 3′ of TAR, which contains the 5′ poly(A) signal [38]. This hairpin structure is similar in both genomes, with unreactive loop nucleotides in the same area in SIVmac239 as in HIV-1_NL4-3_. In the 5′ poly(A) region of HIV-1, *in vitro* analysis suggests formation of a pseudoknot [39]. The SIVmac239 SHAPE reactivity is also low in the region in *gag* that corresponds, based on the codon alignment, to one of the pseudoknot stems in HIV-1_NL4-3_. It is likely that a pseudoknot forms in this region of the SIVmac239 RNA as well (Figure 3). Nucleotide sequence conservation of the poly(A) stem-loop was previously noted for HIV-1, SIV, and HIV-2 sequences [39]; structural similarity in the MA region, based on SHAPE analysis, provides an independent line of evidence supporting this pseudoknot structure (Figure 3).

**Figure 4:**
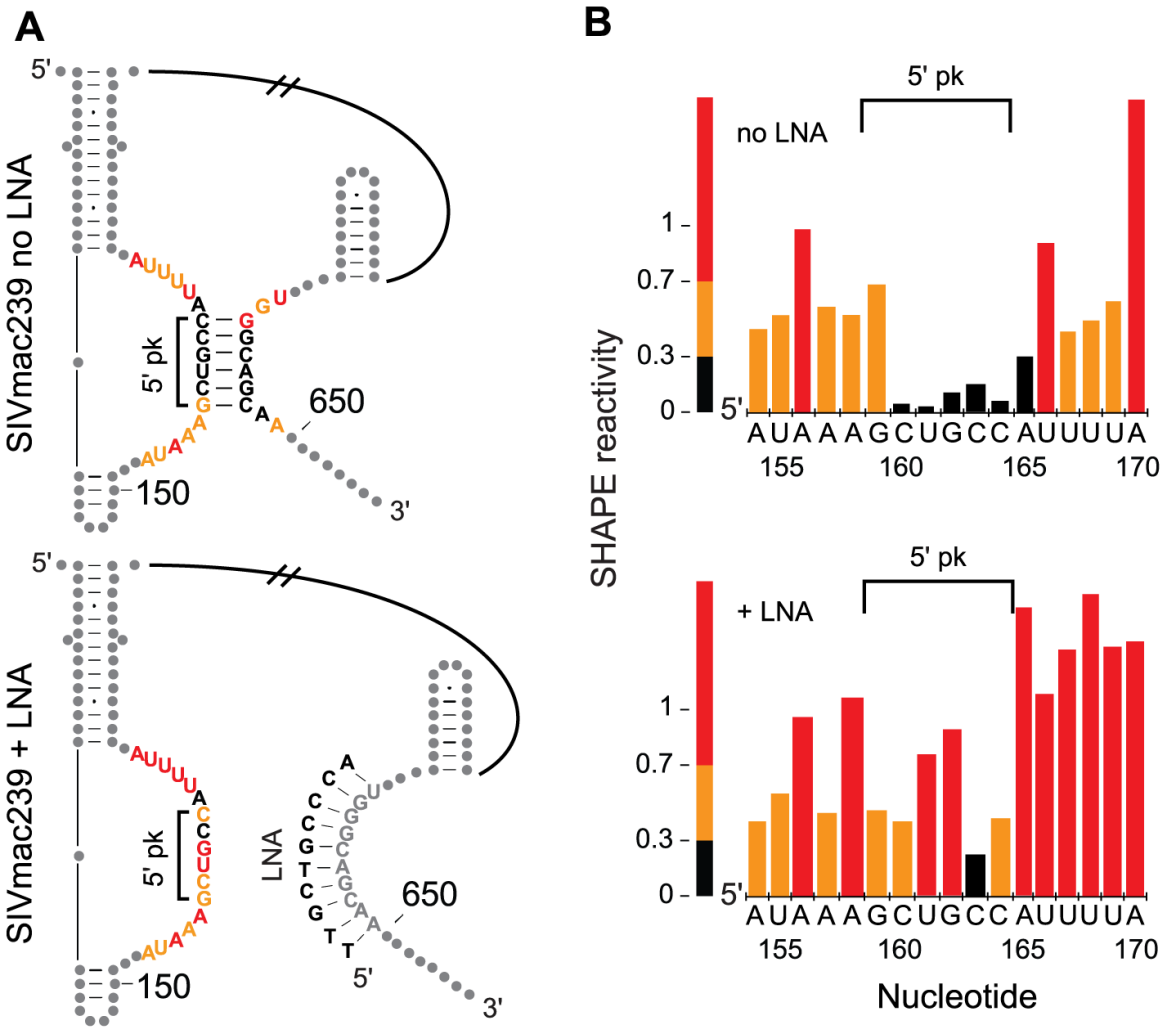
LNA oligo seclusion of half of the predicted 5′ poly(A) pseudoknot in SIVmac239. (A) The SHAPE-derived structures of SIVmac239 5′ poly(A) hairpin and the predicted pseudoknot interaction without (top) and with (bottom) addition of an oligo containing locked nucleic acids (LNA). The 5′ half of the pseudoknot is indicated by the label 5′ pk. Gray dots indicate nucleotides outside of the area of interest. Nucleotides not shown are indicated by hatched lines. (B) Corresponding SHAPE reactivity values at the 5′ half of the predicted pseudoknot of SIVmac239 without (top) and with (bottom) the addition of an oligo containing LNAs and are colored according to the given scale. The 5′ half of the pseudoknot is indicated by the label 5′ pk.

We directly tested the long-range pseudoknot interaction between the stem loop that forms near the 5′ poly(A) signal and the complementary sequence at the beginning of the MA coding domain using a locked nucleic acid (LNA) oligonucleotide to anneal to and sequester pairing partners in the folded SIVmac239 RNA. An LNA was designed to bind to the 3′ side (in the MA coding region) of the predicted pairing interaction and annealed to the RNA. We then probed the surrounding area (nts 1–882) for changes in SHAPE reactivity, excluding nucleotides bound by the LNA. The putative pairing partner GCUGCC in the poly(A) stem-loop showed a clear overall increase in reactivity after seclusion of the downstream pairing partner (Figure 4). A few additional nucleotides showed slight reactivity changes (data not shown), which likely reflect RNA structural shifts due to the disruption of the pairing interaction. Furthermore, an analysis performed using an RNA spanning nucleotides 1–560 of the 5′ region of HIV-2, which has about 88% nucleotide identity to SIVmac239 over these sequences, provides SHAPE data of the poly(A) hairpin when its potential pseudoknot pairing partner within the MA-coding region has been deleted [37]. The HIV-2 data for the truncated RNA show significantly higher reactivity over the 5′ half of the predicted pseudoknot (GCUGCC) as compared to the full-length SIVmac239 RNA analyzed here with the downstream pairing partner intact. Together, these results support formation of a pseudoknot in the loop region of the 5′-UTR poly(A) stem, similar to that observed in the same area in the HIV-1 genomic RNA [39]. In sum, although only 13 base pairs are formally identical, the 5′-UTR structure is highly conserved between the SIVmac239 and HIV-1_NL4-3_ RNA genomes both at the level of overall structure (Figure 2B) and in terms of the local architecture of multiple functional elements (Figure 3). Overall, this analysis emphasizes the striking capacity of RNA to form conserved functional structures despite a very low level of absolutely conserved base pairing.

### Conserved structure in the Gag-Pro-Pol frameshift region

**Figure 5:**
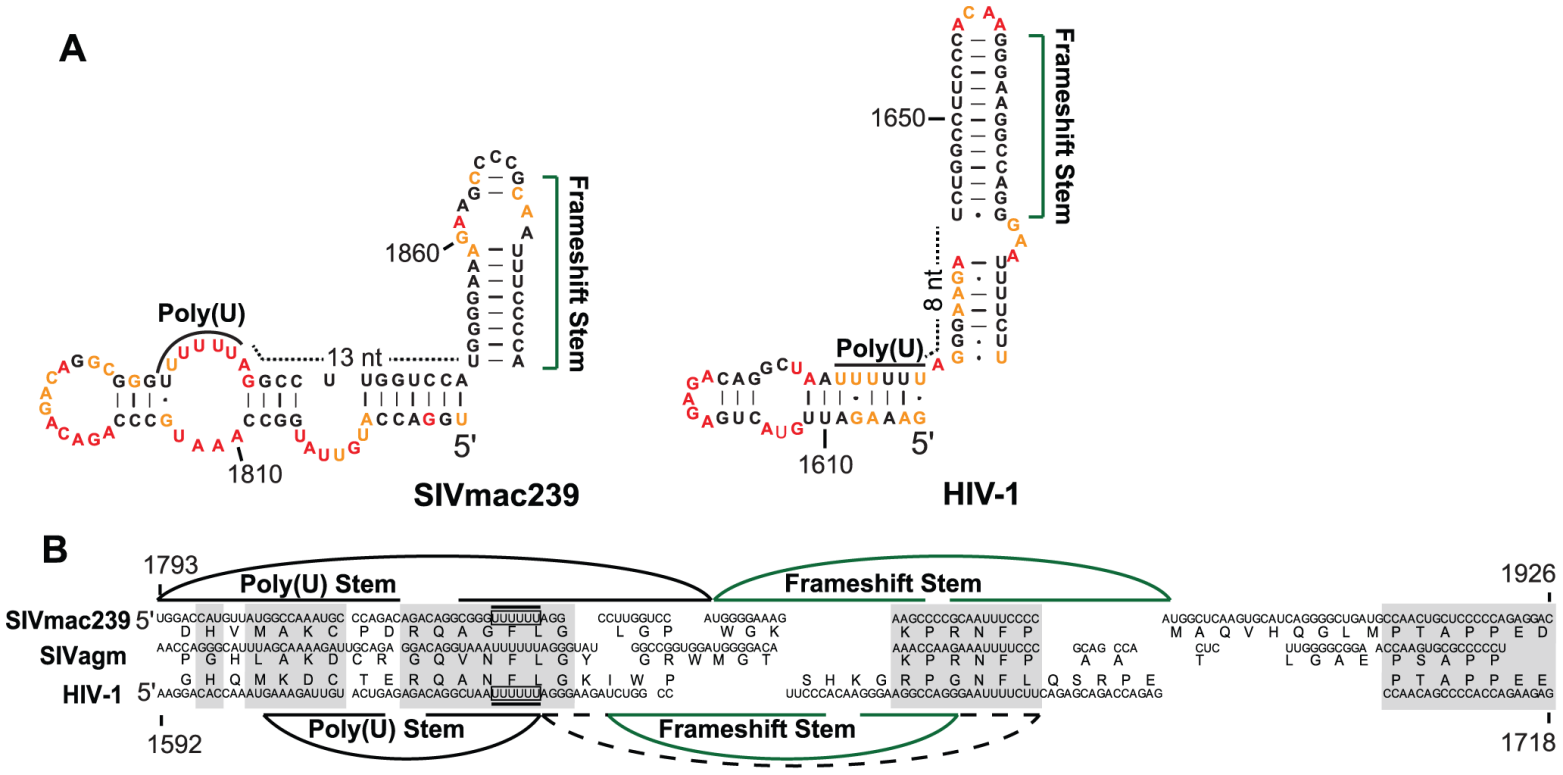
Codon alignment and predicted pairing partners in the Gag-Pro-Pol frameshift region of SIVmac239 and HIV-1_NL4-3_. (A) RNA structures at the Gag-Pro-Pol frameshift stem for SIVmac239 (left) and HIV-1 (right). The main stem is emphasized by green brackets and the poly(U) slippery sequence is emphasized by a curved line. The dotted line indicates nucleotides between the poly(U) sequence and the beginning of the frameshift stem. (B) The sequences of HIV-1, SIVagm, and SIVmac239 were aligned horizontally. The poly(U) slippery sequence is boxed. Curved lines represent base pairs between the nucleotides within HIV-1 and SIVmac239; the curved lines connecting nucleotides downstream of the poly(U) sequence correspond to the frameshift stems (green), the dotted line represents the extended frameshift stem in HIV-1. Gray boxes indicate regions of strong alignment, and spaces in the sequence indicate regions of poor alignment. The structure of the HIV-1 hairpin is modified from the previous model [17] based on the updated folding parameters as described in the Supporting Material.

The primate lentiviruses generate more of the Gag polyprotein than the Gag-Pro-Pol polyprotein by controlling expression using minus-one frameshifting to join translation in the Gag reading frame to translation in the Pro-Pol reading frame. Frameshifting occurs at a “slippery sequence”, a poly(U) region, and is facilitated by a downstream structure that stalls the ribosome [3], [4]. The RNA structure in the region of the frameshift site is similar in SIVmac239 and HIV-1_NL4-3_. In both cases, the poly(U) slippery sequence is part of a stem, although the poly(U) region is not paired in the SIVmac239 structure. In addition, there is a second stem just downstream of the U stretch; the frameshift stem is further from the poly(U) sequence in SIVmac239 than it is in HIV-1_NL4-3_ (Figure 5A). Despite the fact that this region carries out a conserved and essential function in retrovirus replication, the organization of these stems is different in the two genomes and they have no shared base pairs (Figure 5A).

We attempted to define the pathway through which these structures evolved by examining the sequences surrounding the poly(U) slippery sequence. To facilitate this analysis, we included a sequence from SIVagm (GenBank accession M30931), which is distantly related to both SIVmac239 and HIV-1_NL4-3_. All three sequences aligned well at the protein and nucleotide levels, both upstream and through the poly(U) slippery sequence (Figure 5B, gray boxes). However, the alignment is lost three nucleotides 3′ of the poly(U) sequence. The sequence becomes re-aligned at the conserved protease processing site (KPRNFP), lost, then aligns again at the conserved PTAPP motif in the Gag p6 coding domain (Figure 5B, 3′-most gray box). One explanation for the abrupt loss of sequence alignment in this region is that the frameshift hairpin is, itself, mutagenic, consistent with the idea that structure in the RNA would induce pausing of reverse transcriptase and thereby enhance recombination and mutation during viral DNA synthesis [40], [41]. Both frameshift hairpins are predicted to be among the most stable in their respective genomes. The frameshift hairpin ranks in the top 5% in its calculated stability among all hairpins in the SIVmac239 genome; the relevance of this calculation is supported by the low reactivities of paired residues in this hairpin relative to other hairpins (data not shown). A structure stable enough to stall the ribosome may also induce pausing of reverse transcriptase during DNA synthesis, ultimately promoting template strand exchange (recombination) [42], which can be mutagenic when pairing at the 3′ end of the cDNA is imprecise. We thus hypothesize that the rapid evolution of structure in the frameshift region is due to the mutagenic effect of the structure itself.

### Conserved structure in the Rev Response Element (RRE)

**Figure 6:**
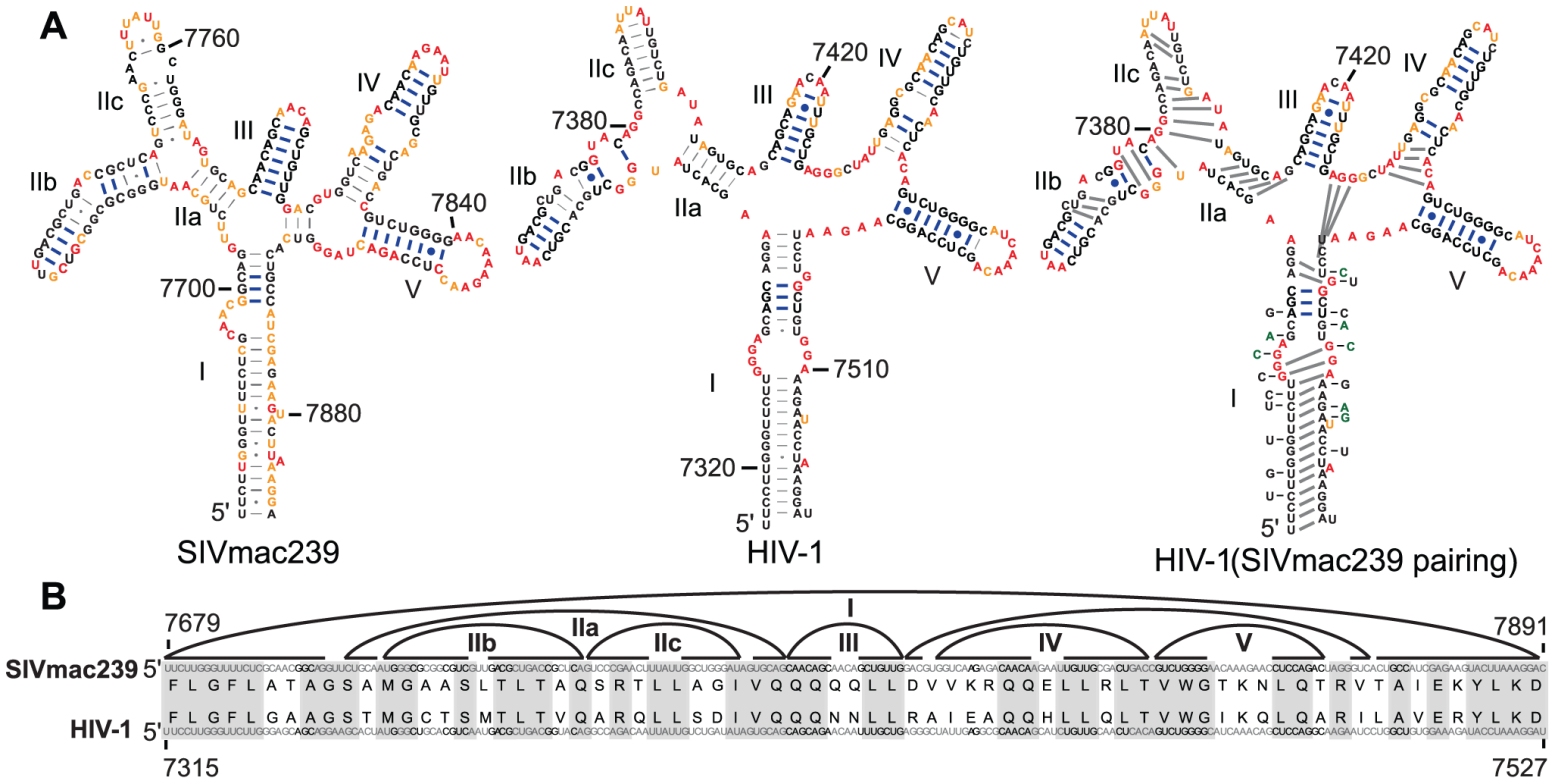
Codon alignment and predicted pairing partners in the RRE region of SIVmac239 and HIV-1_NL4-3_. (A) Predicted structures within the RRE are shown for SIVmac239 (left) and HIV-1 (middle). Codons of stem I are in brackets with their corresponding amino acids labeled and numbered from the first codon of stem I. Blue brackets indicate an area of conserved pairing partners; green brackets indicate an area of shifted pairing partners in stem I. The HIV-1_NL4-3_ structure with forced SIVmac239 pairing is also shown with sequence variations that occur in SIVmac239 indicated while those that change the amino acid sequence are in green (right). Blue lines indicate base pairs that are exactly conserved between the two viruses. (B) The sequences of SIVmac239 (top) and HIV-1_NL4-3_ (bottom) aligned horizontally. Curved lines indicate base-pairing partners. Gray boxes indicate regions of amino acid alignment. Roman numerals indicate helices discussed in the text.

The RRE includes binding sites that mediate oligomerization of the Rev protein; oligomerized Rev mediates export of unspliced and singly-spliced viral RNA from the nucleus [43]. The sequence is conserved in many primate lentiviral genomes [44]. The predicted RRE structure (Figure 1) consists of a long, irregular stem I helix terminated by a set of small hairpins including the IIb stem, previously described as the primary Rev binding site [45], [46], and the auxiliary hairpins (stems III, IV, and V) that facilitate Rev multimerization [47]. The RRE structures for HIV-1 and SIV have been previously evaluated [48], here we analyze the SHAPE-constrained structures from both genomes as they relate to one another. Twenty-nine of the 71 base pairs conserved between SIVmac239 and HIV-1_NL4-3_ occur in the small terminal hairpins in the RRE (Figure 6A, blue bars); these nucleotides are 78% conserved at the sequence level. By contrast, the long stem is mostly devoid of conserved pairing partners, and there is a shift by one nucleotide in the codon-aligned pairing (Figure 6A). In an effort to propose a mechanism by which this shifted pairing in stem I may have occurred, we mapped the variant nucleotides in stem I of SIVmac239 onto the structure of HIV-1_NL4-3_. There are 18 nucleotide differences total (36% of the 50 nucleotides that compose the SIVmac239 stem I), eight nucleotides in HIV-1 would break a base pair that exists in SIVmac239, and six nucleotides in SIVmac239 would do so for HIV-1_NL4-3_. Seven nucleotide differences in the stem I region account for the five amino acid differences in this region, and of these, four have no effect on pairing partners in the stem (Figure 6A). We suspect, therefore, that the shift in the pairing register is a product of multiple synonymous nucleotide changes that occurred over time and not due to one or two specific nucleotide mutations. We postulate that the viruses have accomplished structural divergence without introducing a deleterious intermediate through the placement of bulges within stem I. Furthermore, when we used the codon alignment (Figure 6B) to force superposition of the equivalent SIVmac239 pairing partners onto the RRE structure of HIV-1_NL4-3_, there was a significant reduction (30%) in the number of base pairs formed in stem I (Figure 6A, right structure). Thus, conservation of specific base pairs is limited to four of the small hairpins that serve as protein interaction regions, whereas the long stem is comprised of pairs that are shifted by one nucleotide in the two RNA genomes (Figure 6). We infer that neither the sequence nor the exact base-pairing partners of the long stem I are critical, although its presence and long length are conserved. In contrast, the strong conservation of pairing partners in the small, Rev-binding stems reflects the specificity of the Rev-RNA interaction as the source of the selective pressure that conserves both nucleotide identity and pairing partners.

### Conserved structure at the first splice acceptor site SA1

**Figure 7:**
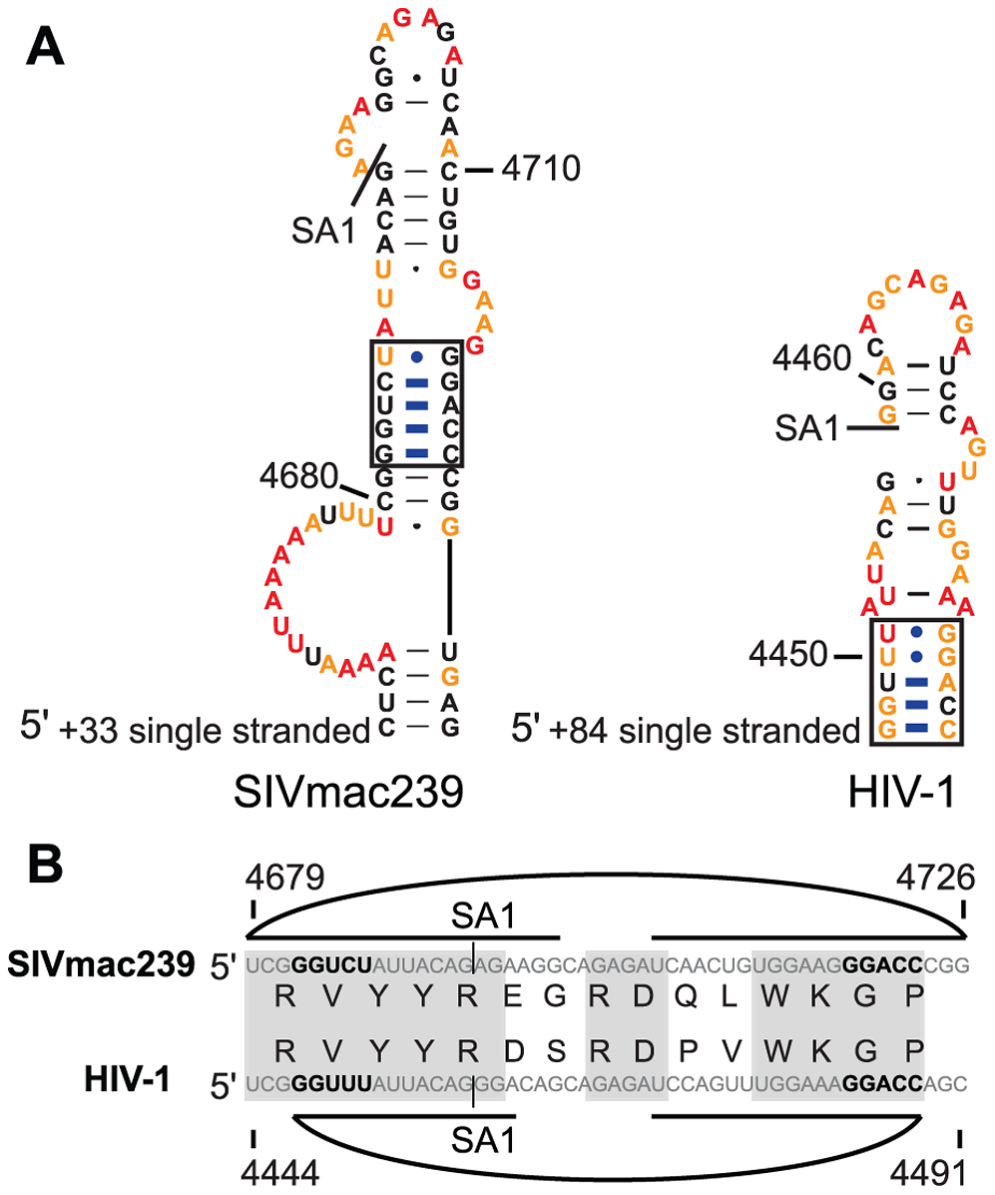
Codon alignment and predicted pairing partners in the stem-loop surrounding SA1 of SIVmac239 and HIV-1_NL4-3_. (A) Structures for the conserved stem in SIVmac239 (left) and HIV-1_NL4-3_ (right). Blue lines indicate the base pairs that are exactly conserved between the two viruses. (B) The sequences of SIVmac239 (top) and HIV-1_NL4-3_ are aligned horizontally. Curved lines indicate base-pairing partners. Gray boxes indicate regions of amino acid alignment. Bold letters represent the bases that are involved in the conserved pairing interactions.

Retroviruses use diverse splice donor (SD) and splice acceptor (SA) sites to generate numerous spliced mRNAs which direct synthesis of the small regulatory proteins and the Env protein while retaining some unspliced RNA for both translation of Gag and Gag-Pro-Pol and for packaging of the full-length genome into new virions [10]. Splicing to generate these mRNAs is highly regulated. This regulation takes place at both the sequence level and at the RNA structure level. Five of the base pairs that are precisely conserved between SIVmac239 and HIV-1_NL4-3_ are in the stem of the hairpin structure that contains the first splice acceptor site (SA1) (Figure 7), which generates the transcript that codes for the viral Vif protein [49]. We have termed this conserved structure SLSA1 (stem-loop at splice acceptor 1). Most of the other splice acceptor regions of SIVmac239 (SA2–SA8) downstream of SA1 are part of short hairpins as well, with the exception of SA4 (Figure 1). Each of these short hairpins has low median SHAPE reactivity (most are below 0.4); however, only SLSA1 is precisely conserved between SIVmac239 and HIV-1_NL4-3_ with several of the same pairing partners. The viral splice acceptors have weak splicing sequences to allow balanced usage of each with the major splice donor SD1 [50]. The relative strengths of HIV-1 splice acceptor sequences have been previously analyzed, and splicing is most efficient to SA1 of all the splice acceptor sites [51]. We hypothesize that the purpose of the conserved SLSA1 pairing is to regulate splicing to this site and to allow balanced use of this site and the other downstream splice acceptor sites.

**Figure 8:**
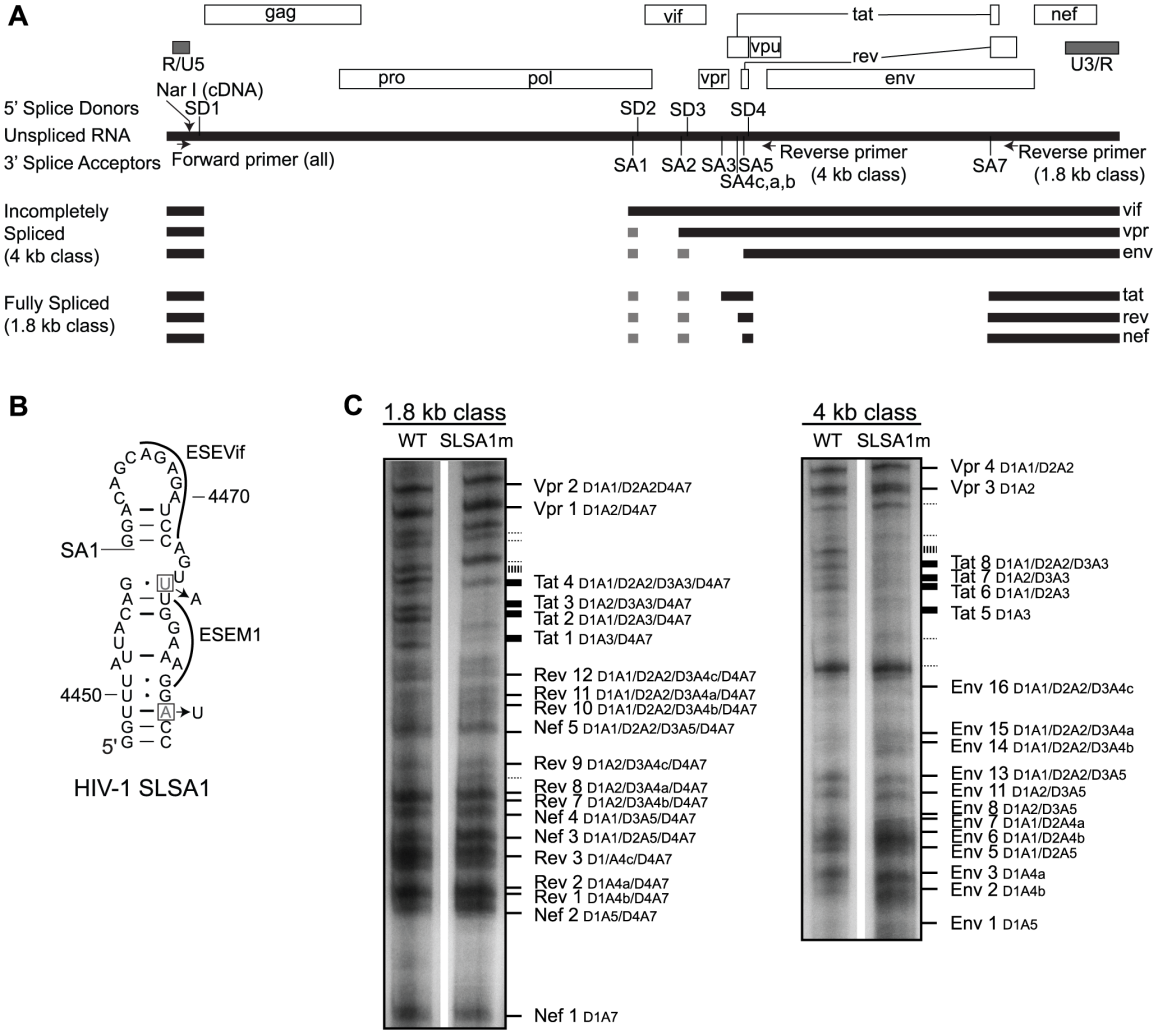
Profiles of HIV-1^wt^ and HIV-1^SLSA1m^ transcripts. (A) Diagram displaying reading frames (open boxes) of the HIV-1_NL4-3_ genome. Solid lines indicate different classes of mRNA including unspliced, 4 kb, and 1.8 kb, with their corresponding genes labeled on the right. Gray boxes represent exons 2 (between SA1 to SD2) and 3 (between SA2 and SD3). Splice donors (SD1-4) and acceptors (SA1-7) are labeled on the top of the unspliced length of RNA The sites of NarI cleavage and primer-binding for the forward and reverse primers used to create the splicing profile are shown on the unspliced RNA. (B) SHAPE-derived structure for the SLSA1 hairpin in HIV-1_NL4-3_. The first splice acceptor (SA1) is labeled. The mutated nucleotides in HIV^SLSA1m^ are boxed, and the mutations are identified next to the arrows. Exonic splicing enhancer sequences (ESEVif and ESEM1) [52], [71] are labeled. (C) Splicing profiles for HIV-1^wt^ and HIV-1^SLSA1m^ grown for three days in CEMx174 cells are shown. The cDNA from the 1.8 kb class (left) or the 4 kb class (right) of transcripts, amplified with the corresponding primers, was separated on a 6% polyacrylamide gel and labeled according to common nomenclature [10] (solid lines) or left unidentified (dotted lines). Decreased band intensity between the WT and SLSA1m transcripts is marked by thicker lines.

We tested the importance of the SLSA1 structure by making mutations that disrupt pairing interactions in this motif and then monitored the effect of these mutations on viral mRNA splicing patterns in HIV-1_NL4-3_. Mutations to SLSA1 incorporated the following constraints: (*i*) to avoid disrupting described splicing enhancer elements within the region downstream of the SA1 site [51], [52], (*ii*) to maintain the *gag-pro-pol* coding sequence, and (*iii*) to use alternative codons that also occur nearby in the sequence as not to require rare or non-viral codon usage (Figure 8B). Although limited in mutation choices, the resulting mutant virus (HIV-1^SLSA1m^) has two single nucleotide substitutions that disrupt the pairing interactions at SLSA1. We infected CEMx174 cell cultures for three days and examined the splicing profile of each viral mRNA pool. The spliced RNA sequences were converted to cDNAs using primers that were placed either after the SD4 or SA7 elements to monitor either the incompletely spliced 4 kb class or the completely spliced 1.8 kb class of viral mRNAs, respectively (Figure 8A). These same primers were then used in PCR that included a unique Nar I site at the 5′ end, upstream of SD1. Each pool of products was cleaved with Nar I and end-labeled, imparting single 5′ labels on each amplicon, indepenent of RNA length. These products were resolved by acrylamide gel electrophoresis and their identity inferred by comparison with a previous assignment [10], although not all products were observed (Figure 8C). This analysis did not resolve the product for the singly spliced Vif mRNA (D1A1); however, we did not see a significant difference in the ratio of products of D1/A1 and D1/A2 for Vpr mRNA, suggesting that the mutations did not have a major effect on the use of A1. The mutations did have a significant effect in suppressing the use of SA3, an acceptor that is used to generate a series of Tat mRNAs in the 1.8 kb class (and their incompletely spliced equivalents in the 4 kb class) (Figure 8C). This effect was specific to SA3 as the downstream splice acceptors for Rev mRNA (SA4a-c) were not affected. Given the complexity of the splicing patterns it will be important to improve the confidence of these assignments, for example using a deep sequencing approach [53]. However, disruption of the conserved SLSA1 RNA structure shifts the balance of spliced mRNA products, although by a mechanism that emphasizes the complexity of splicing regulation and a potential role for long-range RNA structural interactions in this regulation.

### Conserved structure in the cPPT and PPT

**Figure 9:**
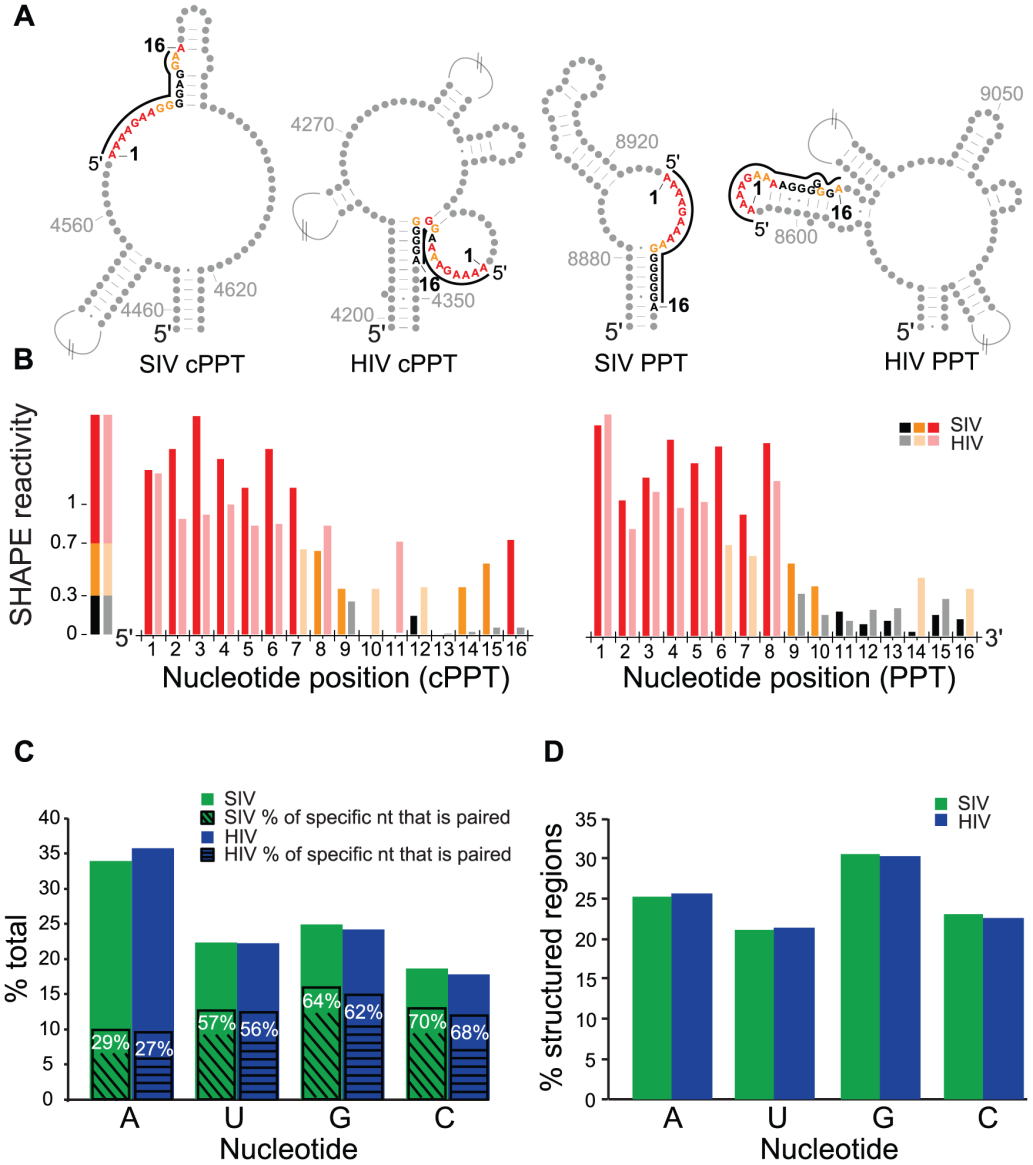
SHAPE analysis of the polypurine tracts of SIVmac239 and HIV-1_NL4-3_ and base composition of both genomes in structured and unstructured regions. (A) RNA structure models for the cPPT and PPT of SIVmac239 and HIV-1_NL4-3_. Nucleotides involved in the polypurine tracts are colored according to their SHAPE reactivity values as in Figure 1. Other nucleotides are light gray. Nucleotides not shown are indicated by hatched lines. (B) Histograms of SHAPE reactivity values, integrated and normalized, along the span of the polypurine tracts. HIV-1_NL4-3_ reactivity values are displayed in a lighter color scale. (C) Histogram of percentage of each individual nucleotide compared to the percentage of each in the entire genome. For each individual nucleotide, SIVmac239 (green) is on the left and HIV-1_NL4-3_ (blue) is on the right. The percent paired for each nucleotide is indicated by hatched lines. (D) Histogram of percentage of each nucleotide in the genome compared to the percentage in highly structured regions of known function (5′-UTR and RRE). SIVmac239 (green) is on the left and HIV-1_NL4-3_ (blue) is on the right.

Retroviruses prime plus-strand DNA synthesis from polypurine tracts (PPT) that are derived from viral RNA during minus-strand DNA synthesis [54]. These primers are resistant to degradation by RNase H, and their specificity as second-strand primers is enhanced by the viral NC protein [55]. Primate lentiviruses prime from two regions, one near the center of the genome (cPPT) and one just upstream of the U3 sequence near the 3′ end of the genome (PPT) [56]. We observed a common structural motif in these polypurine tracts in SIVmac239 and in HIV-1_NL4-3_. The cPPT and PPT motifs in both viruses contain a 5′ A-rich single-stranded region followed by a 3′ G-rich base-paired region (Figure 9A) that have strikingly similar patterns of SHAPE reactivity (Figure 9B). Since the PPT and cPPT function as second-strand primers while hybridized with the first/minus strand of viral DNA, it is unlikely that RNA secondary structure *per se* modulates the function of these sequences as plus strand primers. These patterns drew our attention to the possibility that guanosine and adenosine play very different roles in defining secondary structure more globally across the genome, and that these roles might be reflected in the structures of the PPTs as a byproduct of their high G content in the context of a purine-rich run. This caused us to consider the role of base composition in defining secondary structure more broadly across the genome.

### The role of base composition in defining structure

The base compositions of both the SIVmac239 and HIV-1_NL4-3_ RNA genomes are dominated by adenosine (34%); the percentage of cytidine is low, around 17%, and the percentages of guanosine and uridine are each about 25% (Figure 9C). Regions with large numbers of base pairs must have approximately the same number of pyrimidines and purines. In structured regions with known function, including the 5′-UTR and the RRE in both SIVmac239 and HIV-1_NL4-3_, the average base composition is 25% A, 29% G, 22% C, and 22% U (Figure 9D). The higher percentage of guanosines compared to adenosines in these base-paired regions has the effect of concentrating adenosines in unpaired regions, where adenosines represent fully half of the nucleotides. Only about 30% of adenosines are base-paired, whereas approximately 60–70% of guanosines, cytidines, and uridines are base-paired in the two lentivirus secondary structure models (Figure 9C). This trend toward favoring unpaired adenosines but paired guanosines, cytidines, and uridines is also observed in highly structured regions of RNAs, including bacterial ribosomal RNAs [57]. Given the A-rich primate lentiviral genome, the overall effect is to create A-rich single-stranded regions.

We considered the possibility that the greater stability of the G-C base pair relative to A-U or G-U base pairs might define structures that are conserved between the two genomes. We compared the base compositions of structures within SIVmac239 with known function (5′-UTR, Gag-Pro-Pol frameshift stem, and the RRE) to regions of extensive structure but unknown function. The conserved structures with known function have a higher average guanosine content and a significantly higher (p = 0.04) percentage of G-C pairs (57.9%) than structured regions of the genome that are not conserved (49.5%) (Table S2). Moreover, the SHAPE reactivity was higher for both adenosine and guanosine residues in regions of non-conserved structures (data not shown), suggesting that adenosine-rich structures may facilitate RNA unfolding and refolding or may allow for multiple conformations as compared to guanosine-rich structures in functional regions. We also considered the possibility that clustering of guanosine nucleotides may reflect restricted codon usage for functionally conserved amino acids in the translation product. This model, however, is not supported given that the fraction of codons whose first two positions contain an A and those that contain a G is similar across the SIVmac239 genome and not statistically different within the RRE or Gag-Pro-Pol frameshift. The variation in G or A abundance thus reflects changes that occur at wobble positions and is independent of the amino acid sequence of the translation product. We therefore hypothesize that selection to maintain functional secondary structures in primate lentiviruses such as SIVmac239 and HIV-1_NL4-3_ has resulted in regions defined by clusters of guanosines within an otherwise adenosine-rich genome.

**Figure 10:**
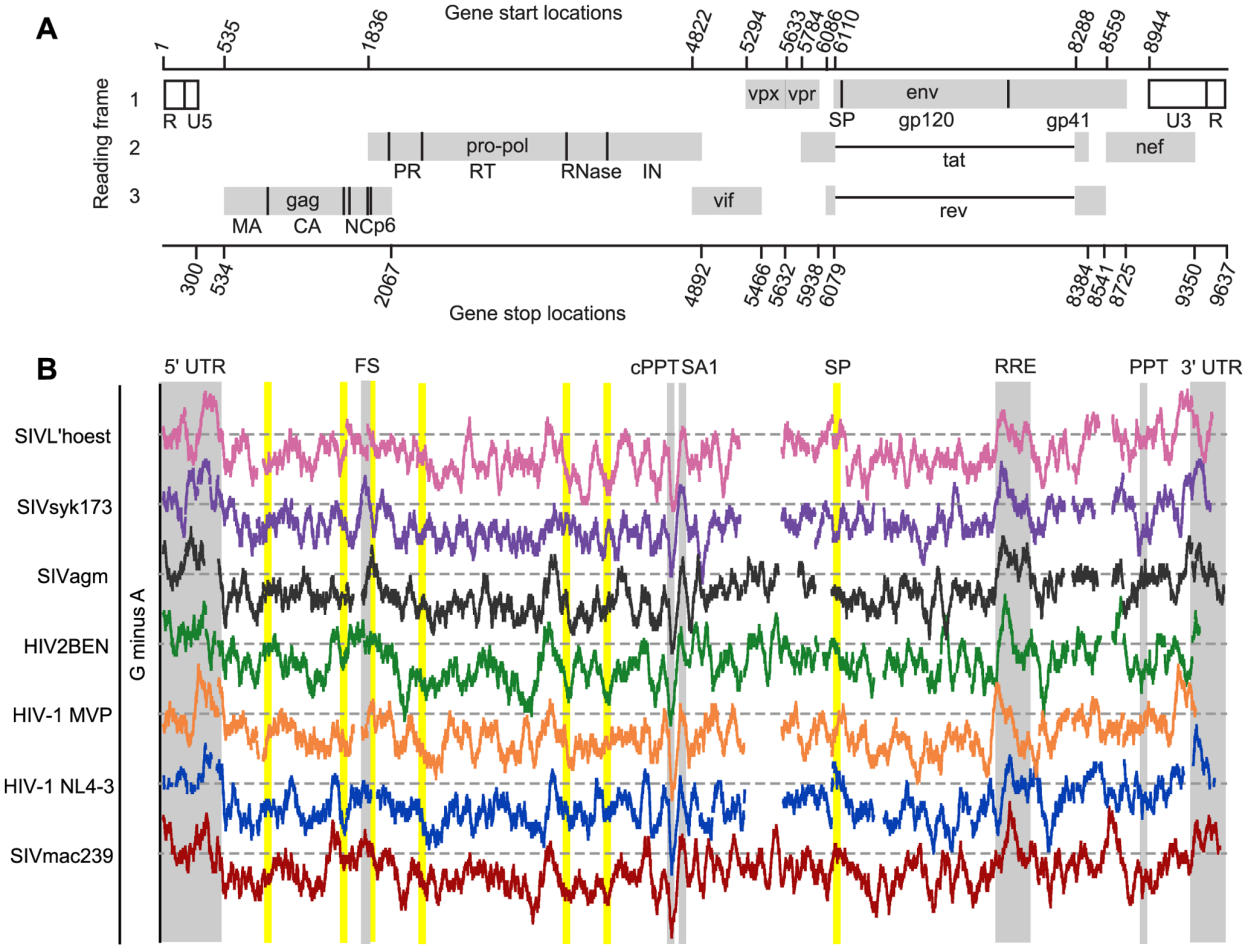
G minus A comparison for genomic sequences of various primate lentiviruses. (A) Organization of the SIVmac239 genome. Gray boxes indicate protein coding regions, dark lines indicate the boundaries of the mature viral proteins. (B) The number of adenosines was subtracted from the number of guanosines (G minus A) in a 75 nucleotide sliding window for selected primate lentiviruses. Dotted lines indicate the point where the difference between the number of G and A is zero for each individual virus. Values above the dotted line indicate higher guanosine concentration than adenosine. Viruses were aligned to SIVmac239 based on protein-coding regions. GenBank accession numbers for each virus are the following: SIVL'hoest, AF075269; SIVsyk173, L06042; SIVagm, M30931; HIV2BEN, M30502; HIV-1 MVP, L20571. Domain junctions on polyproteins are indicated by yellow bars. Other landmarks within the genome are indicated by gray bars.

In an initial attempt to use clusters of guanosines to reveal regions that may be under selection to maintain conserved structures, we scanned a set of aligned primate lentiviral genomes using a sliding window to record a “G minus A” score (Figure 10). As noted above, high guanosine content is easily seen in the 5′ and 3′ UTRs, the Gag-Pro-Pol frameshift site, and the RRE across seven selected genomes (Figure 10, vertical gray bars). In addition, the guanosine cluster at SA1 is readily apparent in these comparisons and is preceded by a dip in guanosine abundance near the cPPT. For the SIVmac239 RNA, we did not find strong evidence for conservation of structure at the boundaries of regions of the RNA that encode protein folding domains (data not shown), a feature reported for the HIV-1_NL4-3_ genome [17]. Consistent with this observation, there is no conserved pattern of guanosine clustering at these boundaries, perhaps with the exception of the region encoding the signal peptide (SP) of Env (Figure 10, vertical yellow bars). We suggest that the central region of primate lentiviral genomes, including the region around the cPPT and SA1, and perhaps the region near the 5′ end of the *env* coding domain, represent new functional structures that are conserved in location if not in detail within the primate lentivirus lineage.

### Summary

Ultimately, HIV-1 and SIVsm/HIV-2 genomic RNAs accomplish many of the same functions in the context of viral replication. There is abundant evidence that RNA structure is either critical for or directly modulates diverse functions including viral DNA synthesis, RNA splicing, genome packaging, and mediation of interactions with both viral and cellular proteins. We sought to identify functionally important RNA secondary structures in the primate lentiviral genome by comparison of SHAPE-directed nucleotide-resolution structure probing information and by developing structural models of representative HIV-1 and SIVsm genomes. We developed a secondary structure model for SIVmac239 and compared it to a modestly revised structural model for the HIV-1_NL4-3_ genome [17]. One paradigm for assessment of conserved function is the ribosomal RNAs where strong base-pairing patterns are highly conserved despite large sequence variations over the course of evolution. The HIV-1_NL4-3_ and SIVmac239 genomes share about 50% sequence identity and feature a similar fraction of base-pairing (60%). However, in contrast to ribosomal RNA-like behavior, only about 3% of predicted base pairs were with identical partners within the coding region of each genome. Almost one-half of these identical pairs were clustered in the Rev binding domain of the RRE. Thus, there has been massive reorganization of the patterns of RNA secondary structure between these two genomes suggesting a selection environment that is very different from that experienced by ribosomal RNA.

Even within regions of highly conserved function, there were large differences in sequence and pairing partners. Dramatic differences in the structure of TAR, longer stems in the 5′-UTR of SIVmac239 relative to that of HIV-1_NL4-3_, different pairing partners and poor sequence alignment in the Gag-Pro-Pol frameshift stem, and a one-base shift in the alignment in the large RRE stem all point to large-scale remodeling of these domains. Stable secondary structures can promote recombination during retroviral DNA synthesis [40], [41] and certain stable structural elements appear to be mutagenic. In regions like the Gag-Pro-Pol frameshift stem, selective pressure does not maintain a particular set of base pairs, but rather ensures that a sufficiently stable structure exists. In contrast, regions involved in RNA-protein interactions, such as the Rev oligomerization domain, displayed a higher level of conservation than other regions of the genome, indicating that selective pressure maintains a particular structure for interaction with protein.

In regions with conserved secondary structures, we observed significantly higher guanosine content compared to the overall base composition of the genome. Higher guanosine levels may serve to stabilize functionally critical structures. Lentiviral genomes are adenosine-rich, and the resulting less stable secondary structures may exist in alternative states, even if they are drawn as a single representative structure in our models. We propose that scanning for guanosine-rich regions in these and other adenosine-rich retroviral genomes may facilitate identification of important structural domains. One source of the selective pressure to maintain an adenosine-rich genome is the action of APOBEC3-G and -F, enzymes that deaminate cytidines on the DNA minus strand during viral DNA synthesis giving rise to G-to-A transitions on the plus strand [58]. Although these lentiviral genomes are adenosine-rich, they are not guanosine-poor (approximately 25% G content) but rather cytidine-poor (at 17% C content). Thus mechanisms must be in place to retain guanosines in regions of functional RNA secondary structures. Focusing on the clustering of guanosines across the primate lentiviral lineage has allowed us to suggest several small areas in the genome that should be explored as possible structural elements that contribute function in the viral replication cycle.

Our analysis shows that within the relatively short evolutionary distance between the HIV-2/SIVsm lineage and HIV-1, conserved secondary structures at the individual base pair level occur at the ends of the RNA and in the RRE. This observation is consistent with the interpretation that, for most of the lentiviral genome, there is little selective pressure to maintain specific pairing interactions. This contrasts the evolutionary pressure on ribosomal RNA. The sequences of 16S rRNA are less than 50% conserved when eubacterial, archaebacterial, and eukaryotic RNAs are compared, but their structures have been maintained through evolution by mutations that compensate for sequence changes that directly affect base pairing (reviewed in [59]). In strong contrast, the lentiviral RRE structure, particularly in stem I, did not evolve through base changes that maintained pairing. We previously examined a model where a higher rate of transition versus transversion mutations exists in paired regions of many RNA structures (presumably to maintain pairing partners), but found that the HIV-1 RRE was an exception as its mutation pattern did not fit this model [60]. The relatively low conservation of base pairs between SIVmac239 and HIV-1_NL4-3_ is consistent with this observation since very few pairing partners are maintained. Clearly the constraints on rRNA evolution, with its many bound proteins, are distinct from the dramatically less rigid constraints on these viral RNA genomes.

We propose that the lentiviral genomic structure is evolving in the context of two significant mutagens. First, APOBEC3-G and -F indirectly mutate guanosines to adenosines, which weakens stability of structural motifs. Second, the structural motifs themselves are mutagenic during DNA synthesis. The effect of these mutagens is filtered by selective pressure to maintain useful structural motifs. The majority of these genomes is thus depleted of both guanosines and strong secondary structure and, in this way, has evolved to be less susceptible to these mutagens. In contrast, the RNA regions that form essential structures are enriched in guanosine residues that provide stability, which also has the effect of ensuring some structure is retained in the face of these mutagens which impart selective pressure.

Since the submission of our manuscript, the following publications have appeared. Van der Kuyl *et al.* [61] show that retroviruses are strongly adenosine-rich and cytidine-poor, in agreement with our conclusions. Van Hemert *et al.* [62] show that the abundant adenosines seem to accumulate in single-stranded regions and not in base-paired regions of the HIV-1_NL4-3_ genome, in agreement with our conclusions.

## Materials and Methods

### Virus production

An infectious clone of SIVmac239 (GenBank accession M33262) was a gift from Dr. Ronald Desrosiers (New England Regional Primate Center, Harvard Medical School) [63]. SIVmac239 was used to infect SupT1 CCR5 CL.30 cells (a gift from Dr. James Hoxie, University of Pennsylvania); these cells are a non-Hodgkin's T cell lymphoma cell line (a modified version of the SupT1 cell line) [64]. The virus produced was purified as described [65].

### Genomic RNA

Viral genomic RNA was gently extracted from purified SIVmac239 viral particles as described [17] in a manner that avoided denaturation of RNA secondary or tertiary structure. Viral RNA was extracted using phenol/chloroform after lysis and treatment with Proteinase K. No heating steps, chelating agents, or chemical denaturants were used in the purification. The final RNA product was precipitated in 70% (v/v) ethanol with 300 mM NaCl and stored at −80°C until use.

### SHAPE analysis of RNA

RNA was treated as described [17]. Briefly, the precipitated RNA was collected by centrifugation and the ethanol removed. Each pellet, containing 62 pmol of SIVmac239 genomic RNA, was individually resuspended in 620 µl of 50 mM HEPES (pH 8.0), 200 mM potassium acetate (pH 8.0), 3 mM MgCl_2_ and incubated at 22°C for 10 min then at 37°C for 15 min. Aliquots of 32 µl of 45 mM 1M7 in dimethyl sulfoxide (DMSO) [27] or DMSO alone were warmed at 37°C for 30 sec, then 288 µl of the RNA solution was added to each and incubated at 37°C for 5 min. RNA was recovered by adding 32 µl of 50 mM EDTA (pH 8.0) and precipitated with ethanol.

### Locked Nucleic Acid binding

An oligonucleotide consisting of DNA and locked nucleic acid (LNA) with the sequence 5′-TTGCTGCCCA-3′ was designed to be complementary to the sequence to be tested for a pseudoknot interaction at the 5′ poly(A) loop. The LNA was added in 5-fold excess to the target RNA with 50 mM HEPES (pH 8.0), 200 mM potassium acetate (pH 8.0), 3 mM MgCl_2_. RNA modification with 1M7 was then performed as described above.

### Primers

Each primer contained a 5′ six carbon linker terminated with an amino group (IDT); a total of 38 primers were used (Table S1). The primers were tethered to 5-FAM or 6-JOE fluorophores (AnaSpec) using N-hydroxysuccinimide chemistry. Purified primers were spectrophotometrically determined to have at least 82% labeling efficiency, with most labeled to greater than 95%, as determined by the [dye]/[DNA] ratio.

### Primer extension

Both the (+) and (−) 1M7 reagent reactions were subjected to reverse transcription with FAM-labeled primers using SuperScript III Reverse Transcriptase (Invitrogen). A sequencing length ladder was generated using the JOE-labeled primers and termination with a dideoxynucleotide. After cDNA synthesis, the reverse transcription reaction products were combined with their corresponding JOE-labeled sequencing reactions, the latter performed using plasmids containing SIVmac239 sequences, p239SpSp5′, and p239SpE3′ (obtained through the AIDS Research and Reference Reagent Program, Division of AIDS, NIAID, NIH from Dr. Ronald Desrosiers [63]. Primer extension products were resolved by length using an Applied Biosystems AB3130 capillary electrophoresis instrument.

### Data processing

ShapeFinder software was used to convert the raw capillary electrophoresis electropherograms of fluorescence intensity to normalized SHAPE reactivities [21], [22], [28]. Data were processed as described [17].

### RNA secondary structure model

The SIVmac239 sequence (9646 nucleotides) with the addition of a poly(A) tail consisting of 10 adenosines was folded using the *RNAstructure* algorithm [66], [67]. SHAPE reactivities were incorporated into the thermodynamic folding algorithm to constrain secondary structure. Due to computational restrictions in the folding algorithm (caused by the length of the genomes), folding was accomplished in large overlapping pieces consisting of at least two-thirds of the entire genome. The structure of the whole genome was generated by combining the separately folded pieces over regions with identical structures. Multiple analyses with varying lengths gave consistent structures. Although we are unable to identify pseudoknots *de novo* with the current algorithm, the model includes the pseudoknot at the 5′ poly(A) stem that we predict based on low SHAPE reactivity of nucleotides in loop regions and by sequence alignment with HIV-1_NL4-3_. Recently updated folding parameters *m* = 1.9 and *b* = −0.7 [30] were used to generate a new version of the HIV-1_NL4-3_ RNA structure (Supporting Material), which was used for these analyses.

### Site-directed mutagenesis

The viral plasmid pNL4-3 was acquired from the National Institutes of Health ADS Research and Reference Reagent Program. For site-directed mutagenesis of pNL4-3, fragments digested with PflMI and AgeI (New England Biolabs) were inserted into vector pT7Blue (Novagen). Mutagenesis primers 5′-GAGATCCAGTATGGAAAGGTCCAGCAAAGCTCCTC-3′ and 5′-GCTTTGCTGGACCTTTCCATACTGGATCTCTGCTG-3′ were used in accordance with the previously described mutagenesis protocol [68]. The resulting plasmid and pNL4-3 were then digested with PflMI and AgeI (New England Biolabs) and ligated with T4 DNA Ligase (New England Biolabs) to create the plasmid pSLSA1m, which was sequenced to confirm the presence of the given mutation.

### Virus production

A total of 2 µg of pSLSA1m or pNL4-3 and was used to produce mutant and wild-type infectious virus (HIV-1^wt^ and HIV-1^SLSA1m^, respectively) by transfection into 3×10^5^ 293T cells in a volume of 2 ml DMEM following the FuGENE (Promega) protocol. After 48 hrs, supernatant from the cells was centrifuged, transferred to 1 ml aliquots, then stored at −80°C. One aliquot per virus was used in a viral infectivity assay [69] to determine infectious units per ml of supernatant.

### Isolation of viral mRNA from cells

To obtain viral mRNA, 1×10^6^ cells were infected with 100 µl virus (either HIV-1^wt^ or HIV-1^SLSA1m^) supernatant in a volume of 0.5 ml for 2 hrs at 37°C before being brought to a final volume of 10 ml and incubated at 37°C for three days. Cells were centrifuged and supernatant was removed. The cell pellet was homogenized through a QiaShredder column (Qiagen) and total mRNA was purified by the RNeasy Mini Prep Plus kit (Qiagen) according to the manufacturer's protocol.

### Viral mRNA profile

Using viral mRNA isolated from three flasks of cells infected with HIV-1^wt^ and three flasks of cells infected with HIV-1^SLSA1m^, we digested each sample with RQ1 DNase (Promega) for 2 hrs at 37°C and purified them again using the same RNeasy Mini Prep kit (Qiagen). We then performed One-Step RT-PCR (Qiagen) following the manufacturer's protocol and using primers 5′-AGTCAGTGTGGAAAATCTCTAGCAGTGG-3′ and either 5′-CCGCAGATCGTCCCAGATAAG-3′ (1.8 kb class) or 5′-CTATGATTACTATGGACCACAC-3′ (4 kb class) in a volume of 25 µl. Each was then digested at a unique restriction site with NarI (New England Biolabs) for 2 hr at 37°C and labeled with 0.78 µCi ^32^P-αdCTP (Perkin-Elmer) using Klenow fragment (New England Biolabs). Each was then purified through a PCR Purification column (Qiagen) and eluted with 30 µl elution buffer (Qiagen). A sample of 10 µl of each was run on a 6% polyacrylamide gel. Controls included mock-infected cultures and cDNA amplification reactions without reverse transcriptase.

### Sequence alignment

SIVmac239 and HIV-1_NL4-3_ sequences were aligned at the codon level using the Los Alamos lentivirus compendium (www.hiv.lanl.gov). Protein start and end positions, known RNA structures, and known protein functional regions were taken into consideration as well as conserved amino acids. Deletions and insertions were incorporated into the sequence alignment where appropriate.

### Statistical analyses

A Matlab 7.8 (R2009a) script was used to compare the actual average absolute difference in SHAPE reactivity value across all aligned SIVmac239 and HIV-1_NL4-3_ genome positions. We randomized the position assignments for reactivity values in the SIVmac239 genome to make a distribution of average absolute differences, then repeated this 100,000 times to generate a random distribution curve and plotted the observed average on this curve. We employed a two-tailed Fishers exact test to compare the GC content to AU and GU content of structures with known and unknown function in SIVmac239. We used a two-tailed Fishers exact test to compare codon composition in the Gag-Pro-Pol frameshift and RRE regions to the rest of the coding region of SIVmac239. The number of adenosines or guanosines in non-wobble positions were counted and compared in both the regions of known function and the rest of the genome.

### RNA structure display

The secondary structures of the RNA models were organized using xrna (http://rna.ucsc.edu/rnacenter/xrna).

### Grammar predictions of structure

Structure predictions using RNA-Decoder [29] were performed as previously described [17] with the following modifications. The input alignment was a reduced version of the HIV-2 web alignment available from the Los Alamos lentivirus compendium (www.hiv.lanl.gov). Codon positions in overlapping regions were designated according to the reading frame of the first member of the following pairs: gag-pro, pol-vif, vif-vpx, vpr-tat1, tat1-rev1, env-tat2, env-rev2, env-nef. The alignment was scanned using separate phylogenetic trees for the upstream and downstream sections, which were generated by Tree-Puzzle [70] using the GTR+*γ* (4) model, 10,000 puzzling steps, “accurate” parameter estimation, and other default settings. The tree for the first half of the genome was built on the third codon positions of the gag, pro, pol, and vif genes and the 5′ non-coding region, and the downstream tree was inferred from the third positions of the vpx, vpr, tat1, env, and nef genes and the 3′ non-coding region. Trees are available from the authors on request.

### Accession numbers

The following GenBank accession numbers refer to the given sequences mentioned in the text: SIVmac239, M33262; HIV-1_NL4-3_, AF324493; SIVL'hoest, AF075269; SIVsyk173, L06042; SIVagm, M30931; HIV2BEN, M30502; HIV-1 MVP, L20571.

## Supporting Information

Figure S1 Secondary structure for the SIVmac239 genome with nucleotide identities shown. The structure is shown in two panels, (A) the 5′ half and (B) the 3′ half.
(TIF)

Figure S2 Comparison of SHAPE reactivities with evolutionary pairing probabilities. (A) Organization of the SIVmac239 genome. Gray boxes indicate protein coding regions, dark lines indicate domain junctions of polyproteins. (B) Comparison of SIVmac239 median SHAPE reactivity values (red) and median phylogenic pairing probability values (cyan). Genome regions where the median pairing probabilities are above 0.6 are indicated by gray bars at the bottom of the plot.
(TIF)

Figure S3 Secondary structure for the HIV-1 genome with nucleotide identities shown. This structure has been revised from the original published version using newly optimized parameters for calculating the pseudo-free energy term. The structure is displayed in two panels, (A) the 5′ half and (B) the 3′ half.
(TIF)

Text S1 SHAPE-directed RNA structure modeling
(PDF)

Dataset S1 Helix file for the SIVmac239 RNA genome structure model folded with parameters m = 1.9 and b = −0.7.
(PDF)

Dataset S2 SHAPE reactivities and pairing probabilities for the SIVmac239 genome.
(PDF)

Dataset S3 Helix file for the HIV-1_NL4-3_ RNA genome structure model folded with parameters m = 1.9 and b = −0.7.
(PDF)

Dataset S4 SHAPE reactivites for the HIV-1_NL4-3_ genome normalized by box-plot normalization.
(PDF)

Table S1 Sequences of primers used for SHAPE.
(PDF)

Table S2 Start and end points corresponding to regions in the 75-nt moving window of median SIVmac239 SHAPE reactivities with values lower than 0.3 (from Figure 2B).
(PDF)
