# Supplementary figures and images for: Comparison of SIV and HIV-1 Genomic RNA Structures Reveals Impact of Sequence Evolution on Conserved and Non-Conserved Structural Motifs
Source: PLoS Pathog. 2013 Apr 4;9(4):e1003294. doi: 10.1371/journal.ppat.1003294 (PMC3616985; doi:10.1371/journal.ppat.1003294)

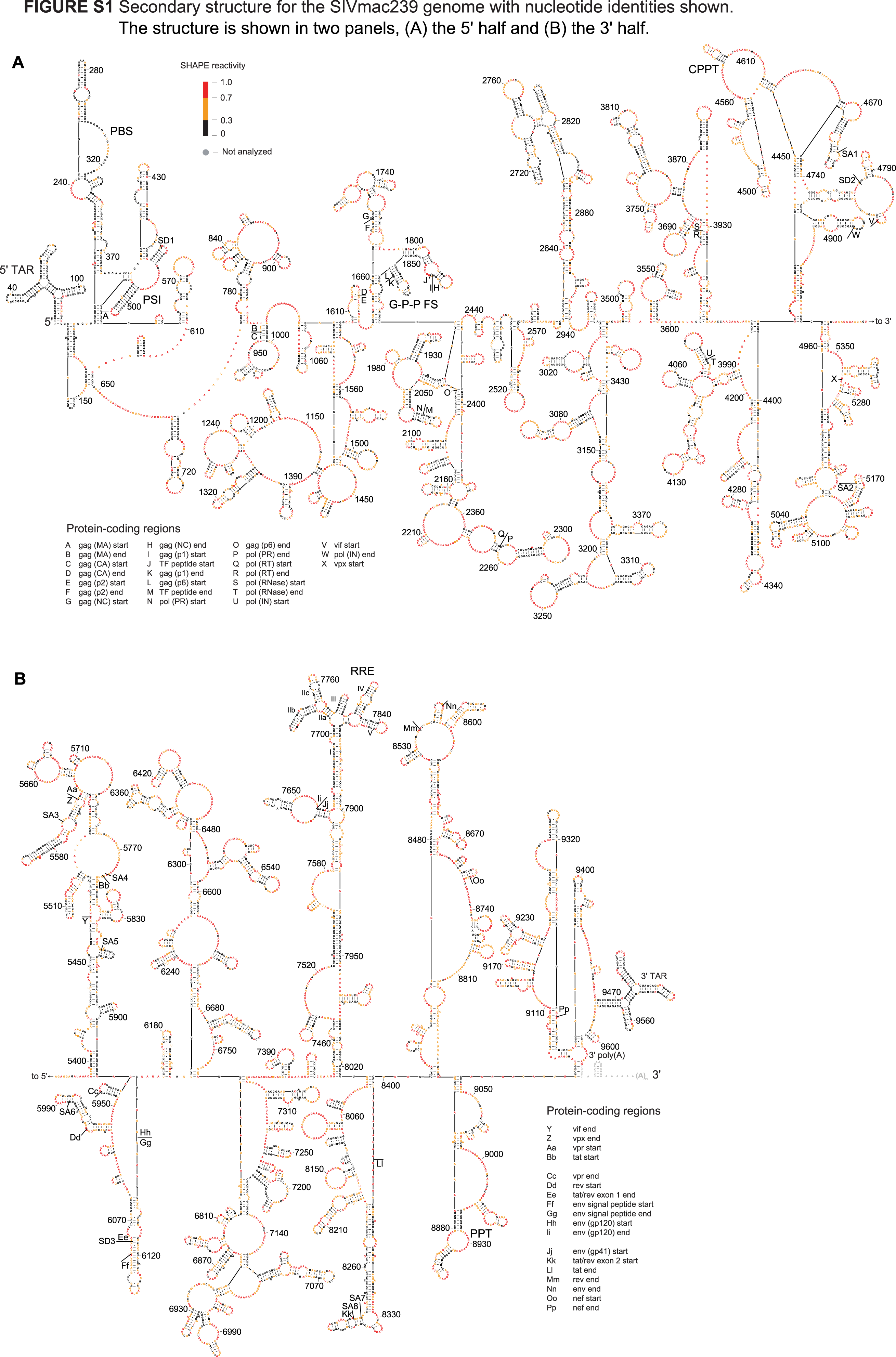

Supplement: Figure S1 — Secondary structure for the SIVmac239 genome with nucleotide identities shown. The structure is shown in two panels, (A) the 5′ half and (B) the 3′ half. (TIF) [file ppat.1003294.s001.tif]

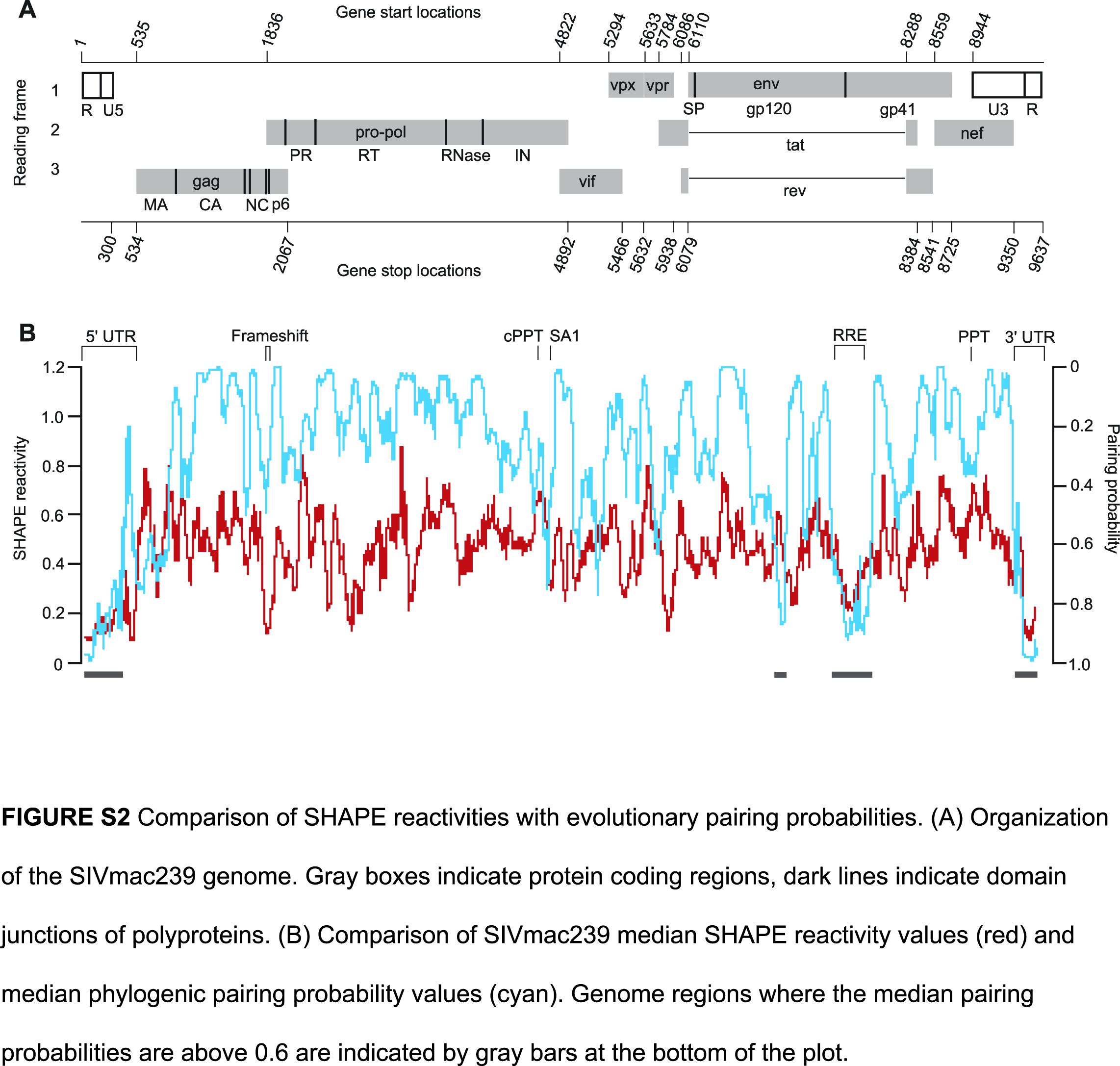

Supplement: Figure S2 — Comparison of SHAPE reactivities with evolutionary pairing probabilities. (A) Organization of the SIVmac239 genome. Gray boxes indicate protein coding regions, dark lines indicate domain junctions of polyproteins. (B) Comparison of SIVmac239 median SHAPE reactivity values (red) and median phylogenic pairing probability values (cyan). Genome regions where the median pairing probabilities are above 0.6 are indicated by gray bars at the bottom of the plot. (TIF) [file ppat.1003294.s002.tif]

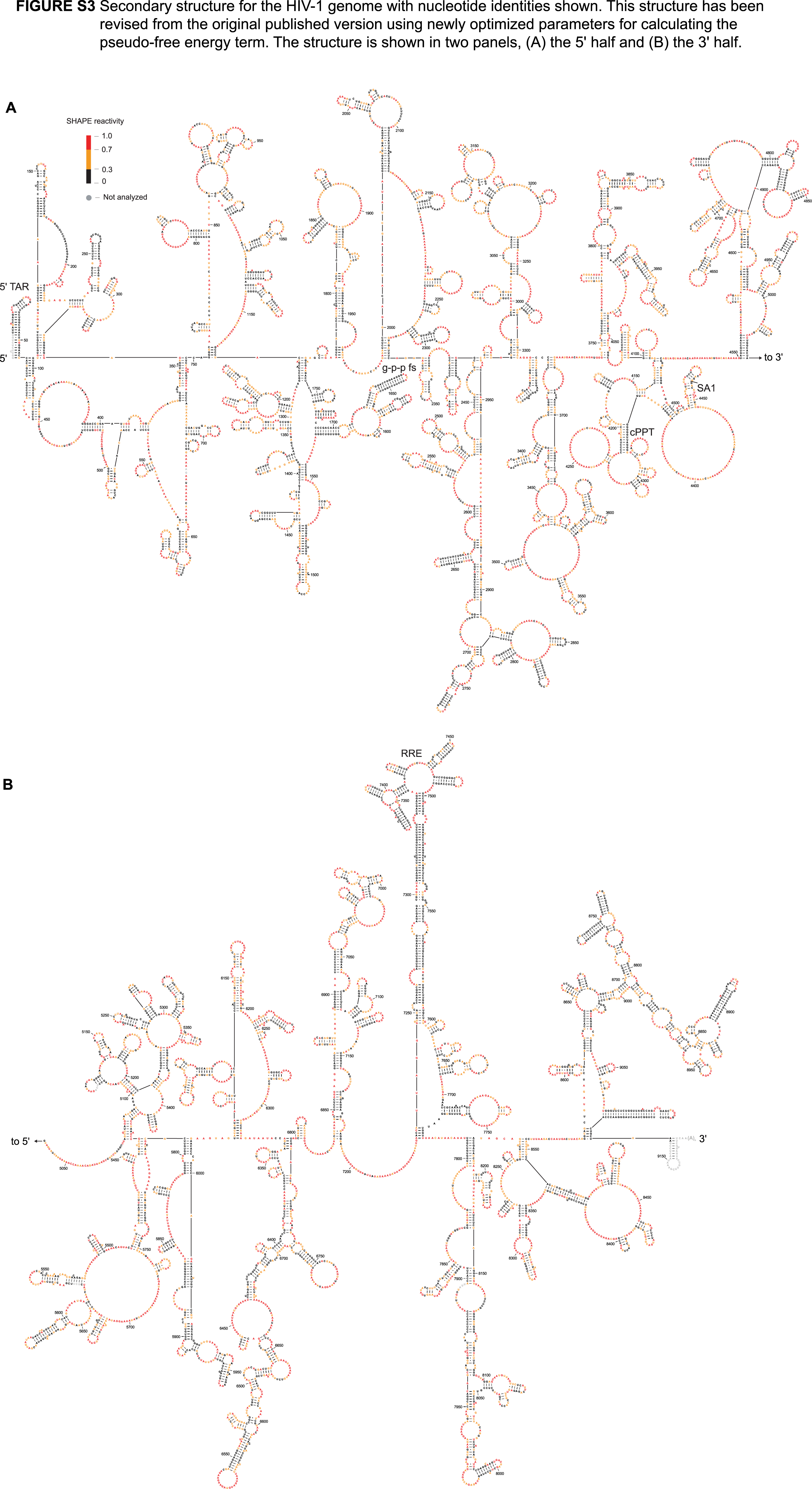

Supplement: Figure S3 — Secondary structure for the HIV-1 genome with nucleotide identities shown. This structure has been revised from the original published version using newly optimized parameters for calculating the pseudo-free energy term. The structure is displayed in two panels, (A) the 5′ half and (B) the 3′ half. (TIF) [file ppat.1003294.s003.tif]
