# Supplementary material for: Comparison of SIV and HIV-1 Genomic RNA Structures Reveals Impact of Sequence Evolution on Conserved and Non-Conserved Structural Motifs
Source: PLoS Pathog. 2013 Apr 4;9(4):e1003294. doi: 10.1371/journal.ppat.1003294 (PMC3616985; doi:10.1371/journal.ppat.1003294)
Supplement: Text S1 — SHAPE-directed RNA structure modeling (PDF) [file ppat.1003294.s004.pdf]

Text S1

SHAPE-directed RNA structure modeling. Inclusion of SHAPE information provides an experimental adjustment to the well-established nearest neighbor model for RNA folding [1]. For secondary structure prediction, SHAPE data are incorporated as a pseudo-free energy change term, ΔG_{SHAPE}, implemented in *RNAstructure* [2]:

$$\Delta G_{\text{SHAPE}} = m\ ln[\text{SHAPE} + 1] + b \quad (1)$$

The slope, *m*, corresponds to a penalty for base pairing that increases with the experimental SHAPE reactivity, and the intercept, *b*, reflects a favorable pseudo-free energy change term for base pairing at nucleotides with low SHAPE reactivities. These two parameters must be determined empirically. When Watts *et al.* analyzed the HIV-$1_{\text{NL4-3}}$ genome, m = 3.0 and b = -0.6 were the optimal parameters [3]; in general, these parameters still perform well. The recently updated parameters are *m* = 1.9 and *b* = -0.7 give the highest sensitivities in a bootstrapping statistical analysis of multiple RNAs [4]. Changing the slope and intercept parameters from *m* = 3.0, *b* = -0.6 to *m* = 1.9, *b* = -0.7 results in a reduction of 34% in the number of base pairs that were predicted in the HIV-$1_{\text{NL4-3}}$ genome, most were in weakly structured regions.

References

1. Mathews DH, Disney MD, Childs JL, Schroeder SJ, Zuker M, et al. (2004) Incorporating chemical modification constraints into a dynamic programming algorithm for prediction of RNA secondary structure. Proc Natl Acad Sci U S A 101: 7287-7292.
2. Deigan KE, Li TW, Mathews DH, Weeks KM (2009) Accurate SHAPE-directed RNA structure determination. Proc Natl Acad Sci U S A 106: 97-102.
3. Watts JM, Dang KK, Gorelick RJ, Leonard CW, Bess JW, Jr., et al. (2009) Architecture and secondary structure of an entire HIV-1 RNA genome. Nature 460: 711-716.
4. Hajdin CE, Bellaousov S, Huggins W, Leonard CW, Mathews DH, et al. (2013) Accurate SHAPE-directed RNA Secondary Structure Modeling, Including Pseudoknots. Proc Natl Acad Sci U S A 110: in press.
