# Supplementary material for: Comparison of SIV and HIV-1 Genomic RNA Structures Reveals Impact of Sequence Evolution on Conserved and Non-Conserved Structural Motifs
Source: PLoS Pathog. 2013 Apr 4;9(4):e1003294. doi: 10.1371/journal.ppat.1003294 (PMC3616985; doi:10.1371/journal.ppat.1003294)
Supplement: Dataset S1 — Helix file for the SIVmac239 RNA genome structure model folded with parameters m = 1.9 and b = −0.7. (PDF) [file ppat.1003294.s005.pdf]

DATASET S1 Helix file for the SIVmac239 RNA genome structure model folded with parameters m = 1.9 and b = -0.7.

First nucleotide in helix	Last nucleotide in helix	Number of base pairs in helix
1	124	11
13	91	5
18	52	5
24	47	3
29	44	5
54	85	8
64	77	4
93	113	8
126	186	6
133	179	8
142	151	3
191	539	8
199	380	4
204	376	6
210	364	4
216	360	2
219	358	7
226	348	5
244	328	3
249	302	9
264	287	10
330	342	4
386	531	4
392	460	5
397	441	7
411	434	4
417	430	3
461	478	6
494	526	7
501	518	6
541	549	2
550	609	5
562	603	3
570	596	3
619	636	7
679	748	10
697	730	2
699	725	5
765	932	5

776	926	3
780	923	4
790	915	6
797	818	5
820	846	3
824	840	3
853	864	4
884	902	4
936	1009	8
954	974	6
975	984	2
1036	1077	4
1043	1065	7
1079	1601	7
1087	1103	6
1111	1562	6
1120	1468	4
1128	1403	4
1161	1190	2
1165	1186	7
1198	1225	5
1227	1291	3
1256	1270	5
1273	1283	4
1297	1346	7
1309	1339	2
1312	1336	2
1317	1331	4
1357	1389	3
1360	1385	7
1409	1423	3
1425	1441	6
1469	1503	7
1514	1549	3
1518	1545	3
1526	1537	3
1577	1589	4
1605	1636	3
1615	1628	4
1644	1901	6
1651	1891	4
1659	1883	4
1664	1788	3

1671	1780	1
1673	1778	5
1679	1772	3
1688	1760	4
1693	1725	2
1702	1716	5
1737	1756	6
1793	1851	7
1806	1843	4
1814	1833	4
1852	1879	8
1863	1869	2
1903	2433	4
1909	2065	4
1913	2059	4
1918	2054	3
1926	1964	9
1935	1951	2
1938	1948	3
1968	1984	2
1996	2050	8
2014	2040	6
2021	2034	3
2067	2406	9
2076	2396	2
2088	2121	6
2094	2113	3
2099	2107	3
2123	2150	10
2151	2389	6
2159	2187	4
2164	2182	2
2166	2179	2
2189	2367	4
2211	2222	4
2237	2354	4
2253	2336	4
2268	2326	7
2275	2318	5
2303	2311	3
2373	2382	2
2408	2428	6
2442	2455	4

2462	2497	3
2465	2493	5
2474	2485	4
2505	2581	4
2510	2555	2
2512	2552	5
2518	2546	4
2564	2573	3
2588	2604	6
2610	2617	2
2618	2938	2
2620	2935	6
2626	2928	2
2630	2906	6
2641	2895	3
2649	2888	3
2653	2885	2
2655	2882	9
2666	2871	4
2671	2866	2
2673	2835	6
2680	2828	4
2685	2824	3
2689	2788	4
2694	2734	4
2700	2728	3
2706	2721	6
2745	2781	8
2790	2815	7
2797	2807	3
2845	2863	2
2848	2860	3
2909	2921	4
2950	2978	3
2953	2974	6
2980	3481	7
2996	3038	3
3007	3026	7
3039	3050	3
3052	3430	4
3057	3425	2
3066	3138	8
3075	3130	3

3085	3118	4
3089	3112	3
3146	3422	4
3150	3416	5
3166	3400	4
3171	3395	8
3193	3331	7
3210	3273	4
3218	3267	8
3228	3235	2
3285	3323	7
3297	3314	3
3334	3382	2
3338	3378	6
3344	3368	5
3436	3473	3
3440	3467	3
3487	3526	5
3496	3517	3
3528	3597	1
3529	3595	8
3551	3565	4
3570	3584	6
3607	3961	5
3613	3953	4
3618	3945	3
3623	3940	2
3626	3637	4
3645	3937	6
3653	3929	3
3657	3704	4
3662	3699	3
3666	3695	3
3712	3860	6
3725	3749	4
3733	3742	3
3759	3772	2
3775	3843	12
3792	3826	2
3795	3823	2
3800	3808	3
3877	3916	4
3883	3910	7

3977	4406	5
3988	4194	6
3997	4185	4
4002	4047	2
4005	4044	2
4009	4042	8
4051	4081	7
4085	4175	5
4095	4166	4
4104	4159	6
4111	4140	4
4142	4151	2
4203	4400	4
4208	4395	6
4228	4255	3
4231	4251	8
4257	4385	3
4263	4378	9
4272	4296	2
4276	4293	4
4301	4367	9
4310	4357	5
4321	4345	2
4325	4337	4
4407	4948	3
4417	4942	4
4422	4937	3
4425	4933	4
4429	4928	4
4434	4860	4
4442	4740	7
4449	4732	2
4458	4627	7
4467	4556	9
4481	4508	5
4489	4499	3
4520	4529	3
4531	4545	4
4575	4597	4
4581	4591	3
4661	4730	4
4679	4726	8
4689	4714	5

4698	4707	2
4744	4854	5
4752	4847	2
4756	4842	7
4770	4793	3
4773	4789	2
4777	4786	2
4814	4824	2
4862	4907	5
4872	4896	7
4950	5365	6
4958	5357	5
4965	5244	6
4973	5234	2
4976	5232	12
4988	5219	2
4994	5012	6
5015	5195	3
5029	5077	2
5032	5074	8
5040	5065	2
5048	5063	4
5079	5098	4
5083	5092	3
5110	5127	3
5130	5153	8
5154	5187	5
5161	5182	5
5168	5177	3
5202	5216	2
5205	5213	2
5246	5272	5
5252	5264	4
5275	5292	2
5277	5288	4
5295	5344	4
5302	5337	2
5304	5334	3
5307	5319	4
5322	5331	3
5393	5923	4
5398	5919	7
5410	5419	3

5421	5911	4
5431	5889	7
5439	5882	3
5445	5878	3
5449	5874	4
5458	5853	3
5461	5846	5
5471	5798	6
5478	5792	4
5483	5787	2
5486	5527	4
5491	5520	4
5495	5514	8
5535	5758	4
5541	5750	5
5548	5743	3
5551	5636	4
5557	5630	3
5561	5627	4
5570	5619	3
5576	5615	4
5581	5611	7
5589	5604	4
5643	5701	7
5663	5671	2
5675	5689	4
5703	5713	3
5801	5840	5
5806	5826	4
5855	5870	2
5858	5867	3
5890	5905	6
5925	6155	1
5927	5946	6
5957	6021	3
5960	6017	5
5965	6008	2
5968	6004	3
5972	6000	4
5980	5996	5
6026	6039	3
6047	6056	3
6062	6144	5

6069	6135	4
6078	6127	7
6086	6120	4
6090	6115	4
6098	6108	2
6160	6202	4
6165	6197	2
6168	6194	6
6176	6187	3
6205	6762	4
6210	6677	8
6218	6667	3
6221	6663	5
6232	6257	2
6235	6255	3
6239	6252	4
6259	6271	4
6272	6279	2
6285	6610	7
6294	6600	5
6301	6308	2
6310	6481	6
6316	6329	3
6331	6389	4
6339	6385	4
6347	6375	4
6352	6360	3
6362	6370	3
6397	6452	9
6407	6425	6
6494	6589	5
6499	6582	4
6517	6529	4
6531	6575	8
6541	6563	4
6628	6652	7
6684	6737	1
6685	6735	3
6688	6731	4
6695	6727	2
6698	6724	2
6701	6722	7
6739	6748	3

6764	7358	3
6777	7165	3
6781	7162	4
6786	7156	6
6793	6803	4
6804	6815	3
6820	6859	3
6827	6849	4
6832	6844	4
6861	6894	2
6863	6891	6
6869	6884	4
6897	7128	6
6903	7029	7
6929	6956	2
6931	6953	2
6934	6950	5
6957	7000	6
6973	6986	5
7003	7021	3
7007	7018	3
7045	7121	5
7051	7116	5
7059	7097	4
7065	7090	8
7099	7110	3
7167	7223	8
7179	7191	4
7204	7214	4
7224	7238	5
7240	7262	4
7244	7255	4
7268	7292	5
7274	7286	4
7300	7349	8
7313	7341	3
7317	7337	3
7373	7411	6
7379	7392	4
7417	7438	7
7444	8024	5
7451	8017	5
7458	8010	3

7466	8006	5
7476	7501	7
7527	7954	5
7533	7949	6
7539	7942	2
7541	7939	7
7549	7932	3
7556	7567	4
7583	7928	2
7587	7924	4
7591	7919	3
7594	7915	3
7601	7909	3
7606	7666	6
7624	7647	5
7630	7640	4
7668	7900	7
7677	7892	7
7684	7884	4
7688	7879	7
7699	7870	5
7707	7774	5
7713	7743	6
7719	7735	7
7744	7767	6
7752	7760	2
7775	7792	7
7793	7864	3
7798	7831	4
7803	7827	2
7808	7822	5
7832	7857	8
7961	7997	2
7963	7994	6
7971	7980	3
8026	8399	5
8042	8058	1
8044	8057	5
8063	8104	7
8106	8122	6
8127	8135	3
8138	8169	5
8175	8231	5

8180	8225	3
8190	8218	6
8198	8210	3
8233	8378	7
8240	8370	7
8251	8359	4
8256	8354	3
8262	8351	4
8267	8346	5
8273	8340	4
8278	8336	4
8284	8315	4
8290	8309	2
8295	8304	3
8319	8328	2
8404	8853	1
8406	8851	4
8418	8432	3
8435	8450	3
8439	8446	2
8452	8838	6
8458	8831	3
8470	8820	3
8474	8694	7
8489	8647	4
8493	8642	1
8496	8639	2
8498	8636	9
8508	8625	3
8515	8533	7
8548	8566	6
8568	8607	2
8571	8603	7
8581	8596	6
8649	8658	3
8660	8686	2
8663	8684	3
8669	8680	2
8702	8713	4
8715	8728	5
8744	8764	3
8766	8777	3
8778	8800	5

8854	9061	7
8862	9016	4
8867	9011	3
8873	8944	9
8888	8922	6
8896	8916	3
8902	8913	2
8949	8983	7
8962	8973	2
9018	9026	3
9028	9039	3
9072	9625	3
9076	9622	4
9081	9368	5
9089	9363	9
9101	9354	3
9105	9350	4
9120	9153	3
9128	9148	3
9131	9144	5
9157	9183	8
9166	9175	3
9185	9246	3
9188	9242	7
9196	9214	2
9199	9212	4
9216	9232	4
9221	9228	2
9252	9345	6
9266	9332	4
9271	9328	3
9274	9324	3
9293	9320	9
9371	9611	5
9382	9427	9
9392	9406	4
9409	9418	3
9444	9452	2
9462	9585	11
9474	9552	5
9479	9513	5
9485	9508	3
9490	9505	5

9515	9546	8
9525	9538	4
9554	9574	8
9594	9606	4
9627	9640	5
