# Supplementary material for: Comparison of SIV and HIV-1 Genomic RNA Structures Reveals Impact of Sequence Evolution on Conserved and Non-Conserved Structural Motifs
Source: PLoS Pathog. 2013 Apr 4;9(4):e1003294. doi: 10.1371/journal.ppat.1003294 (PMC3616985; doi:10.1371/journal.ppat.1003294)
Supplement: Dataset S2 — SHAPE reactivities and pairing probabilities for the SIVmac239 genome. (PDF) [file ppat.1003294.s006.pdf]

DATASET S2 SHAPE reactivities and pairing probabilities for the SIVmac239 genome.

Nucleotide position	Nucleotide identity	SHAPE reactivity	Pairing probability
1	A		0.99
2	G		1
3	U	0.014	0.99
4	C	0.163	1
5	G	0.203	1
6	C	0.122	1
7	U	0.122	1
8	C	0.095	1
9	U	0.136	1
10	G	0.244	1
11	C	0.122	0.96
12	G	0.76	0.04
13	G	0.041	0.97
14	A	0.095	1
15	G	0.068	1
16	A	0.176	1
17	G	0.203	0.97
18	G	0.068	0.97
19	C	0.163	1
20	U	0.597	1
21	G	0.068	1
22	G	0.149	0.97
23	C	0.258	0.02
24	A	0.095	0.95
25	G	0.095	0.99
26	A	0.271	0.94
27	U	0.271	0
28	U	0.203	5.40E-05
29	G	0.095	0.97
30	A	0.095	0.99
31	G	0.095	0.97
32	C	0.068	0.97
33	C	0.068	0.97
34	C	0.068	0.04
35	U	0.434	0.01
36	G	0.258	0.01
37	G	0.244	0
38	G	0.014	0
39	A	0.651	0.02
40	G	0.081	0.97

41	G	0.163	0.99
42	U	0.122	0.97
43	U	0.081	0.99
44	C	0.136	0.97
45	U	0.109	0.95
46	C	0.136	0.99
47	U	0.109	0.95
48	C	0.095	0.97
49	C	0.217	1
50	A	0.054	1
51	G	0.095	1
52	C	0.136	0.97
53	A	0.407	0.03
54	C	0.109	0.96
55	U	0.095	0.98
56	A	0.081	0.99
57	G	0.068	1
58	C	0.081	1
59	A	0.068	1
60	G	0.081	1
61	G	0.122	0.97
62	U	0.38	0
63	A	0.285	0.01
64	G	0.027	0.94
65	A	0.081	0.96
66	G	0.081	0.95
67	C	0.095	0.95
68	C	0.068	0.04
69	U	0.461	0.02
70	G	0.461	0.04
71	G	0.244	0.04
72	G	0.136	0.05
73	U	0.176	0.02
74	G	0.068	0.91
75	U	0.109	0.93
76	U	0.095	0.96
77	C	0.081	0.97
78	C	0.095	0.96
79	C	0.434	1
80	U	0.122	0.99
81	G	0.109	1
82	C	0.285	1
83	U	0.109	1

84	A	0.027	0.99
85	G	0.068	0.97
86	A	0.081	0.87
87	C	0.136	0.97
88	U	0.081	1
89	C	0.095	1
90	U	0.095	1
91	C	0.19	0.96
92	A	0.122	0.04
93	C	0.163	0.98
94	C	0.095	1
95	A	0.054	1
96	G	0.149	1
97	C	0.095	1
98	A	0.081	1
99	C	0.054	0.99
100	U	0.203	0.58
101	U	0.624	0
102	G	0.176	0
103	G	0.149	0
104	C	0.109	0
105	C	0.095	2.00E-06
106	G	0.014	0.58
107	G	0.068	0.99
108	U	0.068	1
109	G	0.068	1
110	C	0.014	1
111	U	0.054	1
112	G	0.746	1
113	G	1.478	0.98
114	G	0.095	0.96
115	C	0.041	1
116	A	0.8	1
117	G	0.746	1
118	A	2.658	1
119	G	0.353	1
120	U	1.506	1
121	G	0.163	1
122	A	0.217	0.99
123	C	2.672	1
124	U	0.054	0.99
125	C	0.217	0.2
126	C	0.054	0.97

127	A	0.203	1
128	C	0.122	1
129	G	0.068	0.98
130	C	0.027	0.99
131	U	0.081	0.94
132	U	0.068	0.77
133	G	0	0.89
134	C	0.041	0.99
135	U	0.014	1
136	U	0.122	0.99
137	G	0.19	0.96
138	C	0.136	0.97
139	U	0.041	0.98
140	U	0.054	0.73
141	A	0.366	0.12
142	A	0.597	0.08
143	A	0.244	0.06
144	G	0.163	0.04
145	C	0.163	0.05
146	C	0.095	0.04
147	C	0.068	0.5
148	U	0.081	0.77
149	C	0.041	0.89
150	U	0.054	0.83
151	U	0.041	0.58
152	C	0.081	0.57
153	A	0.163	0.56
154	A	0.434	0.42
155	U	0.502	0.67
156	A	0.949	0.55
157	A	0.543	0.5
158	A	0.502	0.42
159	G	0.665	0.58
160	C	0.054	0.59
161	U	0.027	0.67
162	G	0.109	0.67
163	C	0.149	0.57
164	C	0.068	0.56
165	A	0.298	0.14
166	U	0.882	0.22
167	U	0.42	0.9
168	U	0.475	0.94
169	U	0.583	0.91

170	A	2.089	0.98
171	G	1.031	0.93
172	A	1.153	0.97
173	A	0.312	0.98
174	G	0.068	1
175	U	0.081	1
176	A	0.122	1
177	A	0.244	0.99
178	G	0.136	0.98
179	C	0.068	0.67
180	U	0.041	0.4
181	A	0.19	0.79
182	G	0.231	0.82
183	U	0.095	0.8
184	G	0.312	0.96
185	U	0.109	0.96
186	G	0.366	0.96
187	U	0.19	0.69
188	G	0.285	0.76
189	U	0.407	0.73
190	U	0.19	0.62
191	C	0.231	0.95
192	C	0	0.99
193	C	0	1
194	A	0.217	1
195	U	0.122	1
196	C	0.081	1
197	U	0.041	1
198	C	0.054	0.98
199	U	0.244	0.97
200	C	0.068	1
201	C	0	1
202	U	0.122	0.96
203	A	0.8	0
204	G	0.855	0.78
205	C	0	0.79
206	C	0	0.8
207	G	0.014	1
208	C	0	1
209	C	0.014	0.98
210	G	0.258	0.85
211	C	0.448	0.87
212	C	0.041	0.99

213	U	0.122	0.77
214	G	0.176	0.31
215	G	0.434	0.01
216	U	1.302	0.96
217	C	0.407	0.99
218	A	0.732	0.97
219	A	1.099	0.38
220	C	0.095	0.51
221	U	0.014	0.9
222	C	0.014	0.89
223	G	0.027	0.51
224	G	0.068	0.54
225	U	0.027	0.98
226	A	0	0.98
227	C	0.068	0.97
228	U	0.041	0.95
229	C	0.068	0.81
230	A	0.163	0.1
231	A	0.556	0
232	U	0.99	0
233	A	5.968	0
234	A	1.017	0
235	U	0.909	0.04
236	A	8.206	0.06
237	A	1.601	0.05
238	G	1.234	0.3
239	A	1.248	0.81
240	A	1.533	0.83
241	G	1.628	0.84
242	A	0.407	0.59
243	C	0.203	0.98
244	C	0.041	1
245	C	0.136	1
246	U	0.027	0.99
247	G	0.19	0.93
248	G	0.19	0.23
249	U	0.122	0.71
250	C	0.122	0.99
251	U	1.234	1
252	G	0.543	1
253	U	0.42	0.99
254	U	0.041	1
255	A	0.095	0.99

256	G	0.434	1
257	G	0.068	0.98
258	A	0.027	0.36
259	C	0.109	0.42
260	C	0.176	0.84
261	C	0.149	0.46
262	U	1.533	0.16
263	U	0.109	0.12
264	U	0.203	0.05
265	C	0.095	0.04
266	U	0.041	0.06
267	G	0.014	0.97
268	C	0.054	0.99
269	U	0.719	0.98
270	U	0.081	0.98
271	U	0.054	0.98
272	G	0.068	0.99
273	G	0.027	0.93
274	G	0.203	0
275	A	0.041	0
276	A	0.014	0
277	A	0.461	0
278	C	0.244	0.94
279	C	0.42	0.99
280	G	0.014	0.98
281	A	0.081	0.97
282	A	0.095	0.97
283	G	0.163	0.99
284	C	0.231	0.97
285	A	0.027	0.11
286	G	0.014	0.91
287	G	3.201	0.91
288	A	5.344	0.08
289	A	0.217	0.09
290	A	0.129	0.06
291	A	0.136	0.03
292	U	0.176	0.35
293	C	0.434	0.37
294	C	0.109	0.81
295	C	0.42	0.81
296	U	0.109	0.99
297	A	0.095	1
298	G	0.19	0.99

299	C	0.081	1
300	A	0.041	1
301	G	0	0.96
302	A	0.041	0.58
303	U	0.217	0.83
304	U	0.109	0.82
305	G	0.176	0.44
306	G	0.041	0.03
307	C	0.109	0.03
308	G	0.258	0.02
309	C	0.326	0.05
310	C	0.271	0.05
311	U	0.326	0.44
312	G	0.244	0.47
313	A	0.353	0.83
314	A	0.624	0.48
315	C	0.488	0.94
316	A	0.42	0.99
317	G	0.163	1
318	G	0.095	1
319	G	0.176	0.99
320	A	0.271	0.56
321	C	0.61	0.81
322	U	0.353	0.91
323	U	0.231	0.9
324	G	0.122	0.44
325	A	0.122	0.14
326	A	0.095	0.01
327	G	0.203	0.03
328	G	0.149	0.1
329	A	0.014	0.91
330	G	0.041	0.94
331	A	0.176	0.74
332	G	0.326	0.95
333	U	0.353	0.75
334	G	0.109	0.01
335	A	0.217	0.01
336	G	0.081	0
337	A	0.095	0
338	G	0.136	0.75
339	A	0.041	0.95
340	C	0.041	0.72
341	U	0.054	0.91

342	C	0.109	0.91
343	C	0.014	0.1
344	U	0.176	0.11
345	G	0.122	0.64
346	A	0.244	0.77
347	G	1.275	0.97
348	U	0.407	0.99
349	A	0.122	0.98
350	C	1.695	0.43
351	G	0.61	0.09
352	G	0.366	0.06
353	C	0.136	0.2
354	U	0.244	0.45
355	G	0.271	0.86
356	A	0	0.88
357	G	0.285	0.82
358	U	0.393	0.97
359	G	0.448	0.99
360	A	0.217	0.95
361	A	0.258	0.43
362	G	0.353	0.66
363	G	0.244	0.66
364	C	0.217	0.96
365	A	0.203	0.32
366	G	0.163	0.22
367	U	0.149	0.01
368	A	0.19	0.01
369	A	0.624	0.02
370	G	0.393	0.04
371	G	0.149	0.89
372	G	0.326	0.98
373	C	0.298	1
374	G	0.217	1
375	G	0	0.99
376	C	0.014	0.97
377	A	0.258	0.96
378	G	0.529	1
379	G	0.244	1
380	A	0.339	0.96
381	A	0.258	0
382	C	0.231	0
383	C	0.217	0
384	A	0.19	0

385	A	0.285	0
386	C	0.122	0.98
387	C	0.258	1
388	A	0.244	0.99
389	C	0.38	0.95
390	G	0.176	0.64
391	A	0.258	0.69
392	C	0.326	0.98
393	G	0.258	0.98
394	G	0.366	0.97
395	A	0.258	0.95
396	G	0.095	0.88
397	U	0.556	0.39
398	G	0	0.97
399	C	0.231	1
400	U	0.298	1
401	C	0.312	1
402	C	0.176	0.99
403	U	0	0.65
404	A	0.014	0
405	U	0.156	0.06
406	A	0.054	0.07
407	A	0.149	0.07
408	A	0.068	0.01
409	G	0.054	0
410	G	0.732	0.54
411	C	0.346	0.99
412	G	0	1
413	C	0.041	0.98
414	G	0	0
415	G	0.285	0.08
416	G	0.699	0.98
417	U	0.17	0.92
418	C	0.142	0.81
419	G	0.136	0.01
420	G	0.434	0
421	U	0.515	0
422	A	0.488	0
423	C	0.76	0.08
424	C	0.326	0.08
425	A	0.38	0.01
426	G	6.144	0.81
427	A	0.353	0.92

428	C	0.217	0.91
429	G	0.081	0
430	G	0.732	0.97
431	C	0.109	0.99
432	G	0	0.99
433	U	0.163	0.62
434	G	0.285	0.09
435	A	1.967	0.71
436	G	0.665	0.99
437	G	0.095	1
438	A	0.163	1
439	G	0.163	1
440	C	0.136	0.98
441	G	0	0.22
442	G	2.618	0.26
443	G	0.692	0.2
444	A	0.841	0.17
445	G	0.692	0.16
446	A	0.583	0.03
447	G	0.814	0.01
448	G	1.112	0
449	A	0.285	0
450	A	0.515	0.02
451	G	0.434	0.03
452	A	0.298	0.02
453	G	0.312	0.02
454	G	0.176	0.51
455	C	0.149	0.62
456	C	0.041	0.98
457	U	0.068	0.99
458	C	0	1
459	C	0.203	1
460	G	1.56	0.95
461	G	0.081	0.43
462	U	0.041	0.99
463	U	0.027	1
464	G	0.014	1
465	C	0	0.89
466	A	0.149	0.79
467	G	0.488	0
468	G	0.868	0
469	U	0.027	0
470	A	1.424	0

471	A	1.18	0
472	G	2.224	0
473	U	0.163	0.79
474	G	0.027	0.89
475	C	0.014	1
476	A	0.068	1
477	A	0.068	0.99
478	C	0.027	0.43
479	A	0.99	0.19
480	C	0	0.15
481	A	1.153	0.11
482	A	0.99	0.3
483	A	1.194	1
484	A	1.031	1
485	A	1.126	0.24
486	A	1.261	0.24
487	G	1.397	0
488	A	1.356	0
489	A	1.139	0
490	A	1.261	6.90E-05
491	U	0.42	2.10E-05
492	A	1.994	0
493	G	1.004	0.46
494	C	0.054	1
495	U	0.014	1
496	G	0	1
497	U	0	1
498	C	0	0.04
499	U	0.014	0.22
500	U	0.014	0.64
501	U	0.041	0.51
502	U	0.027	0.01
503	A	0.054	0.02
504	U	0.014	0.07
505	C	0	0.1
506	C	0	0.04
507	A	1.492	0.01
508	G	0.719	0.56
509	G	1.343	0.62
510	A	1.126	0.19
511	A	0.705	0.12
512	G	1.56	0.08
513	G	0.054	0.06

514	G	0	0.04
515	G	0	1
516	U	0	1
517	A	0.109	1
518	A	0.448	1
519	U	0.515	0.66
520	A	0.109	0.2
521	A	0.081	0
522	G	0.081	0
523	A	0.081	0
524	U	0.014	0.03
525	A	0.149	0.04
526	G	0.231	1
527	A	1.248	1
528	G	0.448	0.97
529	U	0.298	1
530	G	0.203	1
531	G	0.095	0.98
532	G	0.041	0.98
533	A	0.081	1
534	G	0.041	1
535	A	0.095	1
536	U	0.054	1
537	G	0.027	1
538	G	0	0.98
539	G		0.85
540	C	0.095	0.71
541	G	0.556	0.5
542	U	0.393	0.41
543	G	0.895	0.7
544	A	0.787	0.61
545	G	0.787	0.39
546	A	1.194	0.3
547	A	1.75	0.32
548	A	0.068	0.53
549	C	0.014	0.94
550	U	0.014	0.93
551	C	0.014	0.86
552	C	0	0.95
553	G	0.027	0.89
554	U	0.054	0.96
555	C	0.095	0.95
556	U	0.726	0.82

557	U	0.624	0.73
558	G	0.61	0.47
559	U	0.38	0.1
560	C	0.244	0.02
561	A	0.739	0.07
562	G	0.115	0.37
563	G	0.075	0.79
564	G	0.149	0.82
565	A	0.237	0.73
566	A	0.346	0.15
567	G	0.292	0.13
568	A	0.637	0.18
569	A	0.651	0.18
570	A	0.346	0.17
571	G	0.19	0.3
572	C	0	0.63
573	A	0.637	0.75
574	G	0.583	0.62
575	A	0.712	0.47
576	U	0.543	0.45
577	G	0.495	0.43
578	A	0.346	0.7
579	A	0.441	0.76
580	U	0.305	0.86
581	U	0.665	0.83
582	A	0.949	0.24
583	G	0.482	0.17
584	A	0.746	0.18
585	A	0.556	0.16
586	A	0.692	0.09
587	A	1.004	0.06
588	A	0.882	0.67
589	A	0.732	0.73
590	U	0.488	0.87
591	U	0.787	0.79
592	A	1.017	0.42
593	G	0.705	0.79
594	G	0.136	0.97
595	C	0.081	0.99
596	U	0.203	1
597	A	0.597	1
598	C	0.231	1
599	G	0.244	0.97

600	A	0.448	0.99
601	C	0.122	0.98
602	C	0.095	0.94
603	C	0.068	0.81
604	A	0.488	0.8
605	A	0.543	0.8
606	C	0	0.89
607	G	0.19	0.92
608	G	0.8	0.91
609	A	0.909	0.89
610	A	0.977	0.8
611	A	1.044	0.81
612	G	1.044	0.81
613	A	1.261	0.75
614	A	1.234	0.75
615	A	1.356	0.75
616	A	1.017	0.75
617	A	1.221	0.74
618	G	1.18	0.75
619	U	1.329	0.76
620	A	0.203	0.76
621	C	0.122	0.68
622	A	0.027	0.68
623	U	0.081	0.68
624	G	0	0.68
625	U	0	0.84
626	U	0.57	0.99
627	G	1.953	0.96
628	A	1.465	0.45
629	A	0.8	0.22
630	G	1.194	0.14
631	C	0.543	0.4
632	A	0.081	0.51
633	U	0.095	0.67
634	G	0.095	0.68
635	U	0.054	0.65
636	A	0.149	0.49
637	G	0.448	0.52
638	U	0.99	0.65
639	A	0.949	0.59
640	U	1.587	0.8
641	G	0.583	0.75
642	G	0.855	0.9

643	G	0.054	0.87
644	C	0.027	0.9
645	A	0.081	0.78
646	G	0.163	0.65
647	C	0.231	0.69
648	A	0.149	0.4
649	A	0.692	0.42
650	A	0.637	0.42
651	U	0.909	0.61
652	G	0.651	0.76
653	A	1.166	0.86
654	A	1.085	0.91
655	U	0.855	0.97
656	U	0.502	0.77
657	A	1.56	0.24
658	G	1.275	0.22
659	A	1.126	0.22
660	U	1.302	0.21
661	A	1.411	0.27
662	G	1.221	0.72
663	A	0.868	0.89
664	U	0.827	0.96
665	U	0.434	0.93
666	U	0.61	0.8
667	G	0.488	0.58
668	G	0.719	0.42
669	A	0.719	0.59
670	U	0.8	0.91
671	U	0.488	0.63
672	A	1.072	0.36
673	G	0.882	0.4
674	C	0.868	0.41
675	A	0.678	0.37
676	G	1.587	0.37
677	A	0.895	0.38
678	A	1.912	0.37
679	A	1.424	0.33
680	G	0.393	0.13
681	C	0.122	0.9
682	C	0.054	0.98
683	U	0.054	0.99
684	G	0.041	0.99
685	U	0.041	0.99

686	U	0.109	0.99
687	G	0.068	0.97
688	G	0.041	0.89
689	A	0.122	0.76
690	G	0.366	0.85
691	A	1.112	0.77
692	A	1.031	0.61
693	C	0.99	0.4
694	A	0.434	0.36
695	A	0.868	0.41
696	A	1.207	0.73
697	G	1.546	0.75
698	A	0.76	0.76
699	A	0.746	0.62
700	G	0.855	0.18
701	G	0.271	0.29
702	A	0.258	0.56
703	U	0.502	0.66
704	G	0.231	0.4
705	U	0.597	0.24
706	C	0.732	0.2
707	A	0.326	0.24
708	A	1.085	0.27
709	A	0.827	0.24
710	A	1.044	0.23
711	A	0.963	0.26
712	A	0.922	0.41
713	U	1.112	0.84
714	A	0.841	0.91
715	C	0.448	0.83
716	U	0.136	0.77
717	U	0.271	0.33
718	U	0.326	0.28
719	C	0.448	0.27
720	G	0.298	0.18
721	G	0.366	0.67
722	U	0.041	0.75
723	C	0.203	0.75
724	U	0.149	0.57
725	U	0.326	0.54
726	A	0.624	0.18
727	G	1.234	0.24
728	C	0.42	0.63

729	U	0.054	0.79
730	C	0	0.74
731	C	0	0.23
732	A	0.068	0.63
733	U	0.624	0.79
734	U	0.529	0.72
735	A	1.072	0.21
736	G	1.031	0.42
737	U	1.478	0.43
738	G	1.397	0.16
739	C	0.081	0.87
740	C	0	0.96
741	A	0.081	0.96
742	A	0.027	0.97
743	C	0.122	0.97
744	A	0.095	0.96
745	G	0	0.95
746	G	0	0.92
747	C	0	0.95
748	U		0.97
749	C	0	0.68
750	A	0.665	0.46
751	G	1.506	0.35
752	A	0.76	0.26
753	A	1.139	0.43
754	A	0.922	0.53
755	A	0.624	0.66
756	U	0.61	0.89
757	U	0.353	0.93
758	U	0.448	0.78
759	A	1.166	0.66
760	A	1.343	0.8
761	A	1.072	0.78
762	A	1.112	0.64
763	A	1.044	0.31
764	G	0.543	0.26
765	C	0.271	0.25
766	C	0.068	0.27
767	U	0	0.65
768	U	0	0.93
769	U	0.543	0.97
770	A	1.194	0.89
771	U	1.465	0.79

772	A	1.261	0.74
773	A	1.221	0.72
774	U	1.207	0.84
775	A	1.356	0.55
776	C	0.407	0.56
777	U	0.109	0.57
778	G	0.068	0.61
779	U	0.312	0.89
780	C	0.149	0.99
781	U	0.014	0.99
782	G		0.99
783	C	0.136	0.98
784	G	0.014	0.97
785	U	0.298	0.8
786	C	0.258	0.64
787	A	0.19	0.64
788	U	0.353	0.08
789	C	0.163	0.07
790	U	0.081	0.09
791	G	0.041	0.06
792	G	0.203	0.02
793	U	0.163	0.71
794	G	0.041	0.71
795	C	0.19	0.04
796	A	0.122	0.03
797	U	0.515	0.05
798	U	0.176	0.04
799	C	0.326	0.1
800	A	0.149	0.86
801	C	0.448	0.97
802	G	0.163	0.98
803	C	0.61	0.98
804	A	0.339	0.93
805	G	0.637	0.89
806	A	0.57	0.74
807	A	0.882	0.34
808	G	0.543	0.46
809	A	1.004	0.58
810	G	1.017	0.62
811	A	0.882	0.44
812	A	0.936	0.41
813	A	1.221	0.68
814	G	1.18	0.77

815	U	0.258	0.76
816	G	0.041	0.47
817	A	0.258	0.26
818	A	0.624	0.35
819	A	0.556	0.24
820	C	0.678	0.18
821	A	0.339	0.26
822	C	0.407	0.63
823	A	0.136	0.73
824	C	0.353	0.83
825	U	0.095	0.88
826	G	0.203	0.57
827	A	1.207	0.36
828	G	0.529	0.23
829	G	0.42	0.33
830	A	0.448	0.42
831	A	0.515	0.56
832	G	0.529	0.68
833	C	0.407	0.71
834	A	0.42	0.69
835	A	0.949	0.62
836	A	2.814	0.65
837	A	1.77	0.67
838	C	0.916	0.88
839	A	0.244	0.86
840	G	0.549	0.83
841	A	0.597	0.7
842	U	0.861	0.74
843	A	0.529	0.72
844	G	0.902	0.67
845	U	0.522	0.56
846	G	0.339	0.21
847	C	0.583	0.23
848	A	0.414	0.65
849	G	1.051	0.89
850	A	0.76	0.85
851	G	1.051	0.68
852	A	0.861	0.45
853	C	1.356	0.07
854	A	0.366	0.17
855	C	0.285	0.94
856	C	0.156	0.99
857	U	1.478	0.95

858	A	1.519	0.71
859	G	0.848	0.86
860	U	0.597	0.91
861	G	0.448	0.85
862	G	0.203	0.91
863	U	0.244	0.76
864	G	0.047	0.64
865	G	0.19	0.31
866	A	0.732	0.04
867	A	0.875	0.03
868	A	0.983	0.03
869	C	0.929	0.16
870	A	0.678	0.17
871	G	0.841	0.14
872	G	0.536	0.09
873	A	0.665	0.08
874	A	0.855	0.16
875	C	0.922	0.71
876	A	0.285	0.83
877	A	1.261	0.83
878	C	1.316	0.76
879	A	0.203	0.47
880	G	1.356	0.32
881	A	1.194	0.22
882	A	1.478	0.07
883	A	1.37	0.17
884	C	0.855	0.24
885	U	0.163	0.24
886	A	0.231	0.21
887	U	1.424	0.85
888	G	0.543	0.93
889	C	0.285	0.88
890	C	0.081	0.82
891	A	0.258	0.59
892	A	0.719	0.56
893	A	0.841	0.57
894	A	1.438	0.77
895	A	1.221	0.79
896	C	1.289	0.74
897	A	0.231	0.42
898	A	0.977	0.48
899	G	0.814	0.61
900	U	0.814	0.4

901	A	0.054	0.29
902	G	1.044	0.07
903	A	0.841	0.06
904	C	0.705	0.16
905	C	0.149	0.28
906	A	0	0.29
907	A	1.085	0.34
908	C	1.017	0.4
909	A	0.461	0.39
910	G	0.895	0.31
911	C	0.515	0.19
912	A	0.203	0.17
913	C	0.434	0.1
914	C	0.122	0.11
915	A	0.068	0.16
916	U	0.38	0.12
917	C	0.149	0.78
918	U	0.298	0.96
919	A	0.461	0.9
920	G	0.882	0.52
921	C	0.8	0.34
922	G	0.231	0.22
923	G	0.149	0.02
924	C	0.271	0.01
925	A	0	0.03
926	G	0.488	0.09
927	A	0.827	0.14
928	G	1.017	0.53
929	G	0.42	0.51
930	A	0.732	0.21
931	G	0.705	0.29
932	G	0.448	0.17
933	A	0.882	0.02
934	A	0.787	0.02
935	A	0.8	0.03
936	U	0.827	0.26
937	U	0.488	0.33
938	A	0.339	0.21
939	C	0.42	0.47
940	C	0.109	0.55
941	C	0.014	0.17
942	A	0.109	0.02
943	G	0.787	0.01

944	U	0.719	0.01
945	A	0.637	0
946	C	1.058	0
947	A	0.244	0
948	A	0.977	0
949	C	1.031	0
950	A	0	0
951	A	1.031	0
952	A	1.248	0
953	U	1.465	0.02
954	A	1.275	0.08
955	G	0.895	0.07
956	G	0.353	0
957	U	0.258	0.01
958	G	0.271	0.03
959	G	0.068	0.02
960	U	0.136	0
961	A	0.475	0
962	A	0.814	0
963	C	0.583	0.07
964	U	0.258	0.09
965	A	0.366	0.03
966	U	1.248	0
967	G	0.61	0
968	U	0.692	0.01
969	C	0.298	0.02
970	C	0.054	0.03
971	A	0.122	0.04
972	C	0.163	0.01
973	C	0	0
974	U	0.014	0.92
975	G	0.231	0.93
976	C	0.393	0.49
977	C	0.163	0.46
978	A	0	0.14
979	U	0.76	0.31
980	U	0.882	0.88
981	A	0.42	0.9
982	A	1.126	0.46
983	G	0.895	0.47
984	C	0.298	0.07
985	C	0.081	0.02
986	C	0.081	0.53

987	G	0	0.53
988	A	0.76	0.53
989	G	0.692	0.54
990	A	0.61	0.55
991	A	0.705	0.55
992	C	0.57	0.54
993	A	0.081	0.55
994	U	0.949	0.72
995	U	0.8	0.9
996	A	0.502	0.84
997	A	1.438	0.45
998	A	1.017	0.11
999	U	0.99	0.44
1000	G	0.326	0.42
1001	C	0.393	0.36
1002	C	0.122	0.05
1003	U	0.095	0.16
1004	G	0.217	0.19
1005	G	0.095	0.5
1006	G	0.041	0.61
1007	U	0.149	0.82
1008	A	0.176	0.82
1009	A	0.692	0.37
1010	A	0.76	0.31
1011	A	0.814	0.29
1012	U	0.814	0.48
1013	U	0.692	0.55
1014	G	0.61	0.47
1015	A	0.773	0.3
1016	U	1.248	0.05
1017	A	0.461	0.05
1018	G	1.329	0.04
1019	A	1.166	0.03
1020	G	1.072	0.03
1021	G	1.031	0.02
1022	A	0.814	0.01
1023	A	1.261	0.01
1024	A	1.221	0.01
1025	A	1.18	0.01
1026	G	1.316	0.01
1027	A	1.085	0.04
1028	A	1.126	0.05
1029	A	0.909	0.08

1030	U	0.827	0.29
1031	U	0.583	0.31
1032	U	0.637	0.29
1033	G	0.42	0.17
1034	G	0.529	0.21
1035	A	0.719	0.21
1036	G	0.651	0.69
1037	C	0.353	0.76
1038	A	0.122	0.74
1039	G	0.895	0.58
1040	A	0.949	0.56
1041	A	1.139	0.36
1042	G	1.044	0.33
1043	U	0.76	0.33
1044	A	0.8	0.73
1045	G	0.515	0.81
1046	U	0.203	0.84
1047	G	0.149	0.81
1048	C	0.041	0.9
1049	C	0	0.86
1050	A	0.054	0.05
1051	G	0.109	0.08
1052	G	0.488	0.07
1053	A	0.515	0.11
1054	U	0.8	0.13
1055	U	1.017	0.13
1056	U	0.488	0.11
1057	C	0.665	0.11
1058	A	0.692	0.07
1059	G	0.271	0.83
1060	G	0.936	0.9
1061	C	0.136	0.83
1062	A	0.095	0.89
1063	C	0.068	0.91
1064	U	0.326	0.83
1065	G	0.054	0.26
1066	U	0.285	0.6
1067	C	0.746	0.67
1068	A	0.556	0.44
1069	G	0.271	0.23
1070	A	1.438	0.31
1071	A	1.072	0.38
1072	G	1.072	0.5

1073	G	1.275	0.49
1074	U	0.258	0.36
1075	U	0.217	0.86
1076	G	0.258	0.89
1077	C	0.19	0.86
1078	A	0.692	0.59
1079	C	0.434	0.59
1080	C	0.149	0.32
1081	C	0.081	0.43
1082	C	0.068	0.48
1083	C	0.054	0.35
1084	U	0.041	0.36
1085	A	0.122	0.18
1086	U	0.434	0.78
1087	G	0.665	0.78
1088	A	0.42	0.54
1089	C	0.298	0.22
1090	A	0.231	0.11
1091	U	0.081	0.1
1092	U	0.339	0.08
1093	A	0.312	0.06
1094	A	1.411	0.16
1095	U	1.194	0.19
1096	C	1.085	0.21
1097	A	0.651	0.2
1098	G	0.393	0.02
1099	A	0.99	0.07
1100	U	0.665	0.22
1101	G	0.583	0.19
1102	U	0.258	0.12
1103	U	0.353	0.84
1104	A	0.271	0.95
1105	A	0.705	0.83
1106	A	1.37	0.31
1107	U	1.085	0.64
1108	U	1.044	0.9
1109	G	0.624	0.83
1110	U	0.461	0.59
1111	G	0.502	0.55
1112	U	0.176	0.4
1113	G	0.353	0.06
1114	G	0.312	0.43
1115	G	0.136	0.41

1116	A	0.068	0.11
1117	G	0.217	0.02
1118	A	0.353	0.03
1119	C	0.244	0.04
1120	C	0.326	0.22
1121	A	0.054	0.23
1122	U	0.095	0.11
1123	C	0.407	0.22
1124	A	0.434	0.21
1125	A	0.312	0.11
1126	G	0.841	0.29
1127	C	0.895	0.35
1128	G	0.461	0.31
1129	G	0.041	0.11
1130	C	0.217	0.41
1131	U	0.407	0.45
1132	A	0.122	0.45
1133	U	0.326	0.77
1134	G	0.977	0.84
1135	C	0.475	0.85
1136	A	0.488	0.62
1137	G	0.203	0.55
1138	A	0.814	0.38
1139	U	0.773	0.92
1140	U	0.949	0.98
1141	A	0.651	0.84
1142	U	1.072	0.11
1143	C	1.099	0.1
1144	A	0.515	0.1
1145	G	0.556	0.02
1146	A	1.126	0.01
1147	G	1.614	0
1148	A	1.207	0
1149	U	1.004	0
1150	A	1.207	0.08
1151	U	1.166	0.1
1152	U	1.261	0.04
1153	A	0.583	0.04
1154	U	0.76	0.04
1155	A	1.695	0.05
1156	A	1.316	0.02
1157	A	1.736	0.08
1158	C	1.166	0.39

1159	G	1.058	0.32
1160	A	0.597	0.02
1161	G	1.017	0
1162	G	1.506	0.01
1163	A	0.271	0.01
1164	G	0.597	0.02
1165	G	0.583	0.02
1166	C	0.515	0.4
1167	U	0.014	0.45
1168	G	0.027	0.34
1169	C	0.068	0.18
1170	A	0.258	0.18
1171	G	0.176	0.01
1172	A	0.434	0.02
1173	U	0.434	0.35
1174	U	1.078	0.39
1175	G	0.807	0.32
1176	G	1.044	0.02
1177	G	1.207	0.02
1178	A	0.637	0
1179	C	0.522	0
1180	U	0.488	0
1181	U	0.298	0
1182	G	0.298	0.01
1183	C	0.719	0.02
1184	A	1.004	0.02
1185	G	0.583	0
1186	C	0.434	0.01
1187	A	0.393	0.01
1188	C	0.217	0.08
1189	C	0.488	0.56
1190	C	0.936	0.49
1191	A	0.217	0.01
1192	C	0.42	0
1193	A	0.407	0
1194	A	0.271	0.02
1195	C	0	0.59
1196	C	0.719	0.59
1197	A	0.936	0.01
1198	G	0.217	0.01
1199	C	0.461	0.01
1200	U	0.285	0
1201	C	0.231	0.01

1202	C	0.339	0.01
1203	A	0	0
1204	C	1.343	0.95
1205	A	0.909	0.95
1206	A	0.393	0.01
1207	C	1.194	0.01
1208	A	0.936	0.01
1209	A	0.692	0
1210	G	0.882	0.92
1211	G	1.343	0.93
1212	A	0.461	0.01
1213	C	0.855	0
1214	A	0.515	0
1215	A	0.014	0
1216	C	0.312	0.01
1217	U	0.855	0.05
1218	U	2.143	0.04
1219	A	0.339	0.02
1220	G	0.122	0.01
1221	G	0.217	0.92
1222	G	0.231	0.94
1223	A	0.109	0.95
1224	G	0.57	0.95
1225	C	0.109	0.95
1226	C	0.353	0.84
1227	G	0.502	0
1228	U	0.027	0
1229	C	0.366	0
1230	A	1.044	0
1231	G	0.963	0.84
1232	G	0.393	0.95
1233	A	0.176	0.96
1234	U	1.885	0.97
1235	C	0.787	0.96
1236	A	0.624	0.94
1237	G	1.736	0.04
1238	A	0.42	0.03
1239	U	0.855	0.03
1240	A	0.665	0.08
1241	U	0.461	0.16
1242	U	0.651	0.1
1243	G	0.054	0.11
1244	C	1.072	0.08

1245	A	1.031	0.02
1246	G	1.18	0.02
1247	G	1.044	0.03
1248	A	0.868	0.02
1249	A	0	0.02
1250	C	1.248	0.08
1251	A	0.922	0.07
1252	A	0.38	0.07
1253	C	0.461	0.08
1254	U	1.451	0.06
1255	A	0.014	0.03
1256	G	0.041	0.09
1257	U	0.122	0.21
1258	U	0.014	0.14
1259	C	0.231	0.04
1260	A	0.543	0.04
1261	G	0.895	0.08
1262	U	1.736	0.14
1263	A	1.302	0.08
1264	G	1.478	0.04
1265	A	0.583	0.09
1266	U	0.326	0.25
1267	G	0.543	0.19
1268	A	0.42	0.01
1269	A	0	0
1270	C	1.18	0.01
1271	A	1.126	0.01
1272	A	0.787	0
1273	A	0.448	0.03
1274	U	0.122	0.04
1275	C	0	0.02
1276	C	0.882	0.02
1277	A	0.461	0.02
1278	G	0.271	0.02
1279	U	0.298	0.11
1280	G	0.42	0.11
1281	G	0.543	0.02
1282	A	0.068	0.02
1283	U	0.556	0.04
1284	G	0.312	0.04
1285	U	0.99	0.06
1286	A	0.054	0.09
1287	C	1.058	0.06

1288	A	0.732	0.06
1289	G	0.827	0.02
1290	A	0	0.02
1291	C	0.909	0.01
1292	A	0.922	0.01
1293	A	0	0.01
1294	C	0.936	0.01
1295	A	0.773	0.01
1296	G	0.678	0
1297	A	0.285	0.01
1298	A	0.068	0.01
1299	C	0.122	0.01
1300	C	0.163	0.01
1301	C	0	0.01
1302	C	0.583	0.01
1303	A	0.529	0.01
1304	U	1.031	0.01
1305	A	0.298	0
1306	C	0.014	0.01
1307	C	0.651	0.01
1308	A	0.732	0.01
1309	G	0.393	0.01
1310	U	0.827	0.01
1311	A	0.461	0.01
1312	G	0.488	0.01
1313	G	0.231	0.01
1314	C	0.515	0
1315	A	0.448	0
1316	A	0.081	0
1317	C	0.461	0.2
1318	A	0.312	0.25
1319	U	0.515	0.26
1320	U	0.814	0.26
1321	U	1.166	0.24
1322	A	0	0.01
1323	C	0.922	0
1324	A	0.475	0
1325	G	0.855	0.01
1326	G	0.868	0.01
1327	A	0.624	0.24
1328	G	0.868	0.28
1329	A	0.448	0.28
1330	U	0.42	0.27

1331	G	0.271	0.22
1332	G	0.61	0.01
1333	A	0.176	0.01
1334	U	0.149	0.03
1335	C	0	0.05
1336	C	0.461	0.04
1337	A	0.529	0.02
1338	A	0.149	0
1339	C	0.203	0
1340	U	0.149	0.01
1341	G	0.054	0.01
1342	G	0.081	0.02
1343	G	0.109	0.02
1344	G	0.38	0
1345	U	0.081	0
1346	U	0.963	0.01
1347	G	0.068	0.01
1348	C	1.099	0.02
1349	A	1.044	0.02
1350	A	1.099	0
1351	A	1.072	0
1352	A	1.438	0
1353	A	0.583	0
1354	U	0.827	0.02
1355	G	0.353	0.02
1356	U	0.203	0.01
1357	G	0.068	0.01
1358	U	0.054	0
1359	C	0.312	0
1360	A	0.285	0
1361	G	0.732	0
1362	A	0.38	0
1363	A	0.054	0
1364	U	0.068	0.01
1365	G	0.109	0.01
1366	U	1.044	0.01
1367	A	0.719	0
1368	U	1.031	0
1369	A	0.502	0
1370	A	0.109	0
1371	C	0.149	0
1372	C	0.149	0
1373	C	0.624	0

1374	A	0.773	0
1375	A	0.339	0
1376	C	1.017	0
1377	A	0.936	0
1378	A	0.271	0
1379	A	0.054	0
1380	C	0.203	0
1381	A	0.136	0
1382	U	0.312	0.02
1383	U	0.068	0.02
1384	C	0.326	0.19
1385	U	1.397	0.97
1386	A	0.543	0.93
1387	G	0.624	0
1388	A	0.109	0
1389	U	0.922	2.70E-05
1390	G	0.841	0.01
1391	U	1.628	0.94
1392	A	1.017	0.95
1393	A	1.248	0.16
1394	A	1.085	0.01
1395	A	0.705	0
1396	C	1.356	0
1397	A	1.004	0
1398	A	0.868	0.01
1399	G	0.163	0.01
1400	G	0.041	0.01
1401	G	0.014	0
1402	C	0.203	0
1403	C	1.207	0
1404	A	1.411	0
1405	A	1.383	0
1406	A	1.234	0
1407	A	0.705	0
1408	G	1.383	0
1409	A	0.041	0
1410	G	0.054	0
1411	C	0.271	0.01
1412	C	0.773	0.01
1413	A	0.515	0.01
1414	U	0.692	0.19
1415	U	0.8	0.22
1416	U	1.126	0.32

1417	C	1.37	0.32
1418	A	1.112	0.21
1419	G	0.855	0.46
1420	A	0.326	0.35
1421	G	0.109	0.51
1422	C	0.624	0.55
1423	U	0.963	0.59
1424	A	0.434	0.1
1425	U	0.366	0.9
1426	G	0.42	0.98
1427	U	0.543	0.99
1428	A	0.488	0.99
1429	G	0.203	0.99
1430	A	0.149	0.87
1431	C	0.922	0
1432	A	0.8	0
1433	G	0.42	0
1434	G	0.597	0
1435	U	0.041	0
1436	U	0.014	0.87
1437	C	0.041	0.99
1438	U	0.122	1
1439	A	0.014	0.99
1440	C	0.76	0.98
1441	A	0.787	0.9
1442	A	1.004	0.1
1443	A	0.787	0.09
1444	A	0.21	0.5
1445	G	0.597	0.55
1446	U	1.533	0.51
1447	U	1.458	0.64
1448	U	0.97	0.58
1449	A	1.2	0.04
1450	A	0.556	0.03
1451	G	0.366	0.39
1452	A	0.658	0.4
1453	G	1.004	0.08
1454	C	1.017	0.2
1455	A	0.787	0.25
1456	G	0.644	0.17
1457	A	0.109	0.06
1458	A	0.895	0.06
1459	C	0.529	0.11

1460	A	0.583	0.17
1461	G	0	0.19
1462	A	0.515	0.13
1463	C	0.136	0.21
1464	A	0.895	0.22
1465	G	0.42	0.17
1466	A	0.488	0.06
1467	U	0	0.11
1468	G	0.326	0.16
1469	C	0.095	0.13
1470	A	0.326	0.11
1471	G	0	0.03
1472	C	0.258	0.13
1473	A	0.678	0.15
1474	G	0.285	0.17
1475	U	0.895	0.17
1476	A	0.99	0.06
1477	A	0.583	0.04
1478	A	0.38	0
1479	G	0.909	0
1480	A	0.651	0.01
1481	A	0.515	0.03
1482	U	0.529	0.24
1483	U	0.922	0.34
1484	G	0.692	0.24
1485	G	0.393	0.1
1486	A	0.665	0.02
1487	U	0.285	0.17
1488	G	0.326	0.24
1489	A	0.76	0.07
1490	C	0.231	0.01
1491	U	0.637	0.01
1492	C	0.176	0.01
1493	A	0.678	0.01
1494	A	0.176	0
1495	A	0.461	0
1496	C	0.244	0
1497	A	0	0
1498	C	0.353	0
1499	U	0	0
1500	G	0.353	0
1501	C	0.136	0.01
1502	U	0.448	0.01

1503	G	1.044	0.01
1504	A	0.244	0.05
1505	U	1.397	0.07
1506	U	0.461	0.04
1507	C	0.827	0.04
1508	A	0.963	0.01
1509	A	1.099	0
1510	A	0.448	0
1511	A	0.339	0.06
1512	U	0.963	0.76
1513	G	1.465	0.7
1514	C	0.963	0.01
1515	U	0.597	0
1516	A	0.095	0.02
1517	A	0	0.01
1518	C	0.61	0
1519	C	0.461	0.01
1520	C	0.57	0.7
1521	A	1.56	0.7
1522	G	0.678	0.04
1523	A	1.56	0.03
1524	U	0.814	0.01
1525	U	0.732	0.02
1526	G	0.895	0.03
1527	C	0.136	0.01
1528	A	0	0.02
1529	A	0.109	0.01
1530	G	0.42	0.01
1531	C	0.637	0.1
1532	U	0.746	0.11
1533	A	0.407	0.08
1534	G	0.719	0.02
1535	U	0.583	0.01
1536	G	0.705	0.01
1537	C	0.624	0.03
1538	U	0.909	0.09
1539	G	1.166	0.1
1540	A	0.285	0.13
1541	A	0.651	0.06
1542	G	0.312	0
1543	G	0.38	0.01
1544	G	0.705	0.02
1545	G	0.461	0.01

1546	C	1.18	0.13
1547	U	0.366	0.37
1548	G	0.217	0.33
1549	G	0.597	0.42
1550	G	7.352	0.32
1551	U	1.261	0.02
1552	G	1.818	0
1553	U	1.356	0.06
1554	G	1.356	0.06
1555	A	0.773	0.01
1556	A	0.19	0.01
1557	U	0	0.05
1558	C	0	0.14
1559	C	0.136	0.12
1560	C	0.19	0.08
1561	A	0.014	0.07
1562	C	1.261	0.01
1563	C	1.261	0.17
1564	C	1.655	0.3
1565	U	1.194	0.34
1566	A	1.329	0.33
1567	G	1.492	0.09
1568	A	0.841	0.01
1569	A	1.506	0.01
1570	G	0.963	0.01
1571	A	0.99	0.04
1572	A	0.637	0.04
1573	A	0.543	0
1574	U	0.054	0
1575	G	0.176	0
1576	C	0.922	0.02
1577	U	0.76	0.02
1578	G	0.556	0
1579	A	0.271	0
1580	C	0.475	0
1581	G	0.203	0
1582	G	0.407	0
1583	C	0.732	0.12
1584	U	1.153	0.14
1585	U	1.004	0.13
1586	G	0.787	0.1
1587	U	1.112	0.1
1588	C	0.719	0.1

1589	A	0.543	0.1
1590	A	0.258	0.13
1591	G	0.57	0.14
1592	G	0.217	0.12
1593	A	1.017	0.01
1594	G	0.583	0.02
1595	U	0.787	0.97
1596	A	0	0.97
1597	G	0.176	0.13
1598	G	0.597	0.11
1599	G	0.19	0.94
1600	G	0.163	0.95
1601	G	0.271	0.82
1602	G	0.298	0.02
1603	C	0.882	0.76
1604	C	0.502	0.84
1605	G	0.787	0.8
1606	G	0.231	0.26
1607	G	0.149	0.17
1608	A	0.271	0.01
1609	C	0.665	0.06
1610	A	0.163	0.06
1611	G	0.787	0.08
1612	A	0.393	0.12
1613	A	2.889	0.09
1614	G	0.339	0.43
1615	G	0.665	0.43
1616	C	1.139	0.02
1617	U	0.502	0.01
1618	A	0.448	0.01
1619	G	0.597	0.01
1620	A	0.488	0.01
1621	U	0	0.85
1622	U	0.909	0.86
1623	A	1.261	0.04
1624	A	0.76	0.02
1625	U	0.339	0.77
1626	G	0.339	0.89
1627	G	0.38	0.8
1628	C	0.719	0.08
1629	A	0.732	0.02
1630	G	0.624	0.01
1631	A	0.57	0

1632	A	0.231	0.02
1633	G	0.841	0.07
1634	C	0.597	0.86
1635	C	0.57	0.98
1636	C	1.112	0.98
1637	U	0.949	0.98
1638	G	0.773	0.96
1639	A	0.746	0.05
1640	A	1.261	0.05
1641	A	1.261	0.06
1642	G	0.312	0.06
1643	A	0.353	0.04
1644	G	0.285	0.04
1645	G	0.298	0.04
1646	C	0.339	0.04
1647	C	0.326	0.06
1648	C	0.244	0.06
1649	U	1.546	0.13
1650	C	0.895	0.12
1651	G	0.407	0.18
1652	C	0.258	0.38
1653	A	0.122	0.42
1654	C	0.515	0.79
1655	C	0.827	0.89
1656	A	0.909	0.85
1657	G	0.339	0.64
1658	U	0.407	0.41
1659	G	0.203	0.47
1660	C	0.054	0.48
1661	C	0.434	0.5
1662	A	0.258	0.51
1663	A	0.176	0.32
1664	U	0.176	0.54
1665	C	0.163	0.52
1666	C	0.407	0.5
1667	C	0.393	0.5
1668	U	0.787	0.51
1669	U	1.18	0.85
1670	U	1.044	0.95
1671	U	0.556	0.87
1672	G	0.068	0.77
1673	C	0.678	0.68
1674	A	0.203	0.62

1675	G	0.203	0.64
1676	C	0.543	0.63
1677	A	0.109	0.89
1678	G	0.095	1
1679	C	0.217	1
1680	C	0.203	0.01
1681	C	0.298	0.08
1682	A	0.61	0.09
1683	A	0.502	0.06
1684	C	0	0.59
1685	A	0.407	0.71
1686	G	0.651	0.74
1687	A	0.922	0.78
1688	G	0.136	0.28
1689	G	0.203	0.36
1690	G	0.339	0.47
1691	G	0.814	0.4
1692	A	0.258	0.73
1693	C	0	0.82
1694	C	0.882	0.79
1695	A	1.601	0.05
1696	A	1.397	0.01
1697	G	1.072	0.01
1698	A	1.085	0
1699	A	0.868	0
1700	A	0.787	0.01
1701	G	0.543	0.01
1702	C	0.366	0.01
1703	C	0.271	0.98
1704	A	0.366	0.99
1705	A	0.339	0.24
1706	U	1.628	0.14
1707	U	0.97	0
1708	A	1.512	0
1709	A	0.556	0
1710	G	0.536	0.03
1711	U	0.156	0.72
1712	G	0.176	0.84
1713	U	0.271	0.21
1714	U	0.163	0.27
1715	G	0.326	0.26
1716	G	0.448	0.01
1717	A	0.563	0

1718	A	0.726	0.02
1719	U	0.827	0.02
1720	U	0.814	0.02
1721	G	0.651	0.02
1722	U	0.827	0
1723	G	0.651	0
1724	G	0.705	0
1725	G	0.604	0
1726	A	0.814	0
1727	A	1.241	0
1728	A	1.295	0
1729	G	1.316	0
1730	A	0.814	0
1731	G	1.153	0
1732	G	0.665	0
1733	G	0.597	0
1734	A	0.393	0
1735	C	0.081	0
1736	A	0.19	0
1737	C	0.068	0
1738	U	0.122	0.01
1739	C	0.176	0.01
1740	U	0.122	0.01
1741	G	0.488	0.01
1742	C	1.37	0.01
1743	A	1.356	0
1744	A	1.506	0
1745	G	0.773	0.01
1746	G	0.882	0
1747	C	1.994	0
1748	A	0.448	0
1749	A	0.136	0
1750	U	0.136	0.01
1751	G	0.203	0.02
1752	C	0.244	0.09
1753	A	0.203	0.19
1754	G	0.42	0.18
1755	A	0.339	0.16
1756	G	0.163	0.18
1757	C	0.149	0.16
1758	C	0.014	0.03
1759	C	0.081	0.32
1760	C	0.99	0.5

1761	A	1.275	0.43
1762	A	1.275	0.38
1763	G	1.533	0.46
1764	A	1.248	0.33
1765	A	1.275	0.31
1766	G	0.922	0.21
1767	A	1.926	0.09
1768	C	1.194	0.44
1769	A	0.353	0.44
1770	G	0.258	0.45
1771	G	0.258	0.22
1772	G	0.488	0.16
1773	A	0.271	0.03
1774	U	0.244	0.32
1775	G	0.136	0.48
1776	C	0.244	0.82
1777	U	0.38	0.82
1778	G	0.8	0.67
1779	G	0.61	0.4
1780	A	0.76	0.2
1781	A	0.787	0.17
1782	A	0.353	0.71
1783	U	0.515	0.8
1784	G	0.515	0.83
1785	U	0.407	0.8
1786	G	0.366	0.71
1787	G	0.8	0.11
1788	A	1.397	0.02
1789	A	1.533	0.01
1790	A	1.261	0
1791	A	1.289	0.01
1792	A	0.624	0.07
1793	U	0.366	0.12
1794	G	0.122	0.15
1795	G	1.302	0.1
1796	A	0	0.07
1797	C	0.054	0.18
1798	C	0.081	0.77
1799	A	0.42	0.86
1800	U	0.353	0.83
1801	G	1.316	0.76
1802	U	0.637	0.7
1803	U	1.397	0.15

1804	A	1.777	0.18
1805	U	0.814	0.25
1806	G	0.095	0.25
1807	G	0.054	0.15
1808	C	0.014	0.11
1809	C	0.203	0.31
1810	A	1.031	0.38
1811	A	0.895	0.35
1812	A	1.139	0.3
1813	U	0.814	0.79
1814	G	0.393	0.8
1815	C	0.122	0.76
1816	C	0.095	0.85
1817	C	0.217	0.2
1818	A	1.153	0.12
1819	G	1.234	0.1
1820	A	1.058	0.06
1821	C	1.56	0.06
1822	A	1.818	0.06
1823	G	0.651	0.05
1824	A	0.637	0.05
1825	C	0.556	0.09
1826	A	0.76	0.11
1827	G	0.258	0.81
1828	G	0.366	0.83
1829	C	0.393	0.83
1830	G	0.271	0.79
1831	G	0.624	0.26
1832	G	0.109	0.38
1833	U	0.298	0.7
1834	U	0.678	0.79
1835	U	0.909	0.81
1836	U	0.841	0.82
1837	U	1.044	0.79
1838	U	2.862	0.63
1839	A	2.306	0.26
1840	G	1.112	0.49
1841	G	0.095	0.49
1842	C	0.041	0.32
1843	C	0.095	0.41
1844	U	0.136	0.81
1845	U	0.176	0.95
1846	G	0.041	0.47

1847	G	0.014	0.07
1848	U	0.163	0.05
1849	C	0.068	0.04
1850	C	0	0.04
1851	A	0.041	0.33
1852	U	0.095	0.88
1853	G	0.014	0.85
1854	G	0.054	0.72
1855	G	0.027	0.72
1856	G	0.027	0.78
1857	A	0.041	0.87
1858	A	0.122	0.83
1859	A	0.38	0.32
1860	G	0.597	0.36
1861	A	0.882	0.34
1862	A	0.285	0.27
1863	G	0.122	0.23
1864	C	0.407	0.2
1865	C	0	0.24
1866	C	0.054	0.23
1867	C	0.244	0.12
1868	G	0.217	0.13
1869	C	0.312	0.15
1870	A	0.475	0.14
1871	A	0.109	0.03
1872	U	0.068	0.17
1873	U	0.014	0.79
1874	U	0.041	0.84
1875	C	0.054	0.84
1876	C	0.027	0.76
1877	C	0	0.73
1878	C	0.041	0.65
1879	A	0	0.52
1880	U	0.095	0.25
1881	G	0	0.27
1882	G	0.014	0.67
1883	C	0.42	0.72
1884	U	0.095	0.34
1885	C	0.203	0.31
1886	A	0.068	0.68
1887	A	0.149	0.64
1888	G	0.122	0.62
1889	U	0.109	0.61

1890	G	0.665	0.13
1891	C	0.075	0.56
1892	A	0.929	0.56
1893	U	0.644	0.19
1894	C	0.712	0.22
1895	A	1.194	0.22
1896	G	0.359	0.39
1897	G	0.163	0.73
1898	G	0.109	0.69
1899	G	0.244	0.19
1900	C	0.068	0.19
1901	U	0.142	0.51
1902	G	0.224	0.5
1903	A	0.42	0.15
1904	U	0.393	0.33
1905	G	0.217	0.4
1906	C	0.224	0.17
1907	C	0.61	0.15
1908	A	0.807	0.12
1909	A	0.936	0.06
1910	C	0.136	0.16
1911	U	0.156	0.42
1912	G	0.136	0.47
1913	C	0.054	0.43
1914	U	0.075	0.32
1915	C	0.068	0.22
1916	C	0.081	0.25
1917	C	0.047	0.2
1918	C	0.156	0.19
1919	C	0.183	0.12
1920	A	0.807	0.01
1921	G	0.983	0
1922	A	1.017	0
1923	G	0.631	0.05
1924	G	0.536	0.47
1925	A	0.841	0.53
1926	C	0.088	0.24
1927	C	0.054	0.15
1928	C	0.088	0.01
1929	A	0.373	0
1930	G	0.292	0.02
1931	C	0.136	0.05
1932	U	0.224	0.06

1933	G	0.285	0.45
1934	U	0.224	0.45
1935	G	0.414	0.14
1936	G	0.312	0.14
1937	A	0.604	0.1
1938	U	0.176	0.2
1939	C	0.088	0.13
1940	U	0.278	0.01
1941	G	0.366	0.03
1942	C	0.251	0.04
1943	U	0.943	0.04
1944	A	1.126	0.12
1945	A	1.146	0.09
1946	A	0.875	0.01
1947	G	0.807	0
1948	A	0.875	0
1949	A	0.529	0
1950	C	0.251	0.01
1951	U	0.685	0.01
1952	A	1.051	0
1953	C	0.332	0
1954	A	1.363	0
1955	U	0.78	0
1956	G	0.251	2.04E-05
1957	C	0.095	0
1958	A	0.237	0
1959	G	0.156	1.96E-05
1960	U	0.115	8.97E-05
1961	U	0.17	0
1962	G	0.441	0
1963	G	0.047	0
1964	G	0.17	0
1965	C	0.047	6.76E-05
1966	A	0.746	5.97E-05
1967	A	0.563	1.76E-05
1968	G	0.366	1.78E-05
1969	C	0.231	3.09E-05
1970	A	0.61	0
1971	G	0.624	0
1972	C	0.482	0
1973	A	0.495	0
1974	G	0.488	0
1975	A	0.753	0

1976	G	0.793	0
1977	A	1.153	0
1978	G	0.353	0
1979	A	0.827	0
1980	A	0.793	6.78E-06
1981	A	0.909	0
1982	A	1.004	0
1983	G	0.454	0
1984	C	0.387	0
1985	A	0.563	0
1986	G	0.42	0
1987	A	0.705	0
1988	G	0.834	0
1989	A	0.99	3.17E-05
1990	G	0.861	0
1991	A	0.943	0
1992	A	1.038	0
1993	A	1.017	0
1994	G	0.821	0
1995	C	0.414	0
1996	A	0.366	0.02
1997	G	0.366	0.07
1998	A	0.454	0.05
1999	G	0.556	0.05
2000	A	0.665	0.04
2001	G	0.543	0.05
2002	A	0.665	0.02
2003	A	0.773	0
2004	G	0.495	0.54
2005	C	0.21	0.59
2006	C	0.17	0.14
2007	U	0.461	0.17
2008	U	1.044	0.51
2009	A	2.179	0.48
2010	C	0.875	0.54
2011	A	1.261	0.64
2012	A	1.194	0.72
2013	G	1.958	0.7
2014	G	0.217	0.72
2015	A	0.104	0.58
2016	G	0.104	0.42
2017	G	0.298	0.08
2018	U	0.09	0.02

2019	G	0.33	0.1
2020	A	1.885	0.26
2021	C	0.321	0.22
2022	A	0.289	0.41
2023	G	0.511	0.78
2024	A	0.538	0.89
2025	G	0.624	0.85
2026	G	1.13	0.64
2027	A	1.546	0.36
2028	U	0.61	0.11
2029	U	0.995	0.66
2030	U	0.606	0.68
2031	G	0.425	0.66
2032	C	0.09	0.03
2033	U	0.063	0.06
2034	G	0.226	0.05
2035	C	0.208	0.01
2036	A	0.181	0.01
2037	C	0.131	0.23
2038	C	0	0.25
2039	U	0.099	0.25
2040	C	0.172	0.88
2041	A	1.456	0.73
2042	A	1.275	0.7
2043	U	0.095	0.46
2044	U	0.081	0.72
2045	C	0.045	0.84
2046	U	0.086	0.79
2047	C	0.045	0.89
2048	U	0.099	0.9
2049	C	0.118	0.88
2050	U	0.497	0.91
2051	U	1.397	0.9
2052	U	0.999	0.9
2053	G	0.493	0.62
2054	G	0.583	0.38
2055	A	0.606	0.06
2056	G	0.434	0.03
2057	G	0.42	0.09
2058	A	0.502	0.13
2059	G	0.52	0.37
2060	A	0.665	0.39
2061	C	0.357	0.61

2062	C	0.353	0.26
2063	A	0.244	0.19
2064	G	0.538	0.31
2065	U	0.741	0.37
2066	A	0.651	0.29
2067	G	0.316	0.84
2068	U	0.185	0.87
2069	C	0.172	0.39
2070	A	0.181	0.08
2071	C	0.14	0.05
2072	U	0.075	0.11
2073	G	0.109	0.13
2074	C	0.054	0.07
2075	U	0.163	0.07
2076	C	0.142	0.29
2077	A	0.78	0.27
2078	U	0.685	0.09
2079	A	0.8	0.36
2080	U	0.448	0.6
2081	U	0.576	0.43
2082	G	0.929	0.26
2083	A	0.875	0.08
2084	A	1.072	0.08
2085	G	0.631	0.09
2086	G	0.617	0.27
2087	A	0.461	0.26
2088	C	0.102	0.25
2089	A	0.258	0.24
2090	G	0.305	0.16
2091	C	0.095	0.37
2092	C	0.081	0.34
2093	U	0.068	0.64
2094	G	0.59	0.75
2095	U	0.556	0.8
2096	A	0.793	0.74
2097	G	0.827	0.63
2098	A	1.017	0.04
2099	A	0.773	0.05
2100	G	0.292	0.09
2101	U	0.427	0.26
2102	A	0.61	0.21
2103	U	0.319	0.9
2104	U	0.746	0.98

2105	A	1.038	0.98
2106	C	0.163	0.98
2107	U	0.21	0.91
2108	G	0.407	0.29
2109	G	0.576	0.04
2110	A	0.827	0.03
2111	U	0.393	0.04
2112	A	0.475	0.04
2113	C	0.068	0.02
2114	A	0.549	0.02
2115	G	1.472	0.14
2116	G	0.041	0.3
2117	G	0.068	0.19
2118	G	0.19	0.24
2119	C	0.027	0.61
2120	U	0.217	0.87
2121	G	0.407	0.68
2122	A	0.895	0.36
2123	U	0.57	0.26
2124	G	0.298	0.05
2125	A	0.271	0.38
2126	U	0.122	0.6
2127	U	0	0.8
2128	C	0	0.28
2129	U	0.068	0.28
2130	A	0.19	0.25
2131	U	0.312	0.36
2132	U	0.393	0.57
2133	G	2.455	0.59
2134	U	0.868	0.63
2135	A	0.61	0.38
2136	A	0.814	0.28
2137	C	0	0.35
2138	A	0.705	0.25
2139	G	0.746	0.31
2140	G	1.072	0.15
2141	A	0.353	0.07
2142	A	0.203	0.17
2143	U	0.217	0.44
2144	A	0.258	0.4
2145	G	0.231	0.15
2146	A	0.19	0.29
2147	G	0.136	0.68

2148	U	0.149	0.79
2149	U	0.285	0.83
2150	A	1.438	0.46
2151	G	0.244	0.35
2152	G	0.109	0.16
2153	U	0.122	0.03
2154	C	0.041	0.04
2155	C	0.027	0.04
2156	A	0.461	0.05
2157	C	0	0.25
2158	A	0.814	0.55
2159	U	0.38	0.75
2160	U	0.231	0.76
2161	A	0.882	0.3
2162	U	0.298	0.13
2163	A	0.42	0.08
2164	C	0.068	0.09
2165	C	0.014	0.46
2166	C	0	0.6
2167	C	0.014	0.28
2168	A	0.651	0.11
2169	A	0.705	0.1
2170	A	0.637	0.06
2171	A	0.719	0.12
2172	A	1.072	0.14
2173	U	0.244	0.45
2174	A	0.936	0.39
2175	G	0.773	0.43
2176	U	0.298	0.52
2177	A	0.855	0.38
2178	G	0.285	0.48
2179	G	0.583	0.46
2180	A	0.705	0.23
2181	G	0.298	0.18
2182	G	0.326	0.38
2183	A	0.583	0.6
2184	A	0.637	0.63
2185	U	0.231	0.7
2186	A	0.909	0.68
2187	G	0.42	0.52
2188	G	0.719	0.53
2189	A	0.556	0.5
2190	G	0.163	0.44

2191	G	0.19	0.44
2192	U	0.136	0.59
2193	U	0.353	0.77
2194	U	0.502	0.79
2195	U	0.244	0.41
2196	A	0.827	0.35
2197	U	0.393	0.5
2198	U	0.393	0.4
2199	A	0.922	0.27
2200	A	1.004	0.21
2201	U	0.515	0.19
2202	A	0.665	0.24
2203	C	0.136	0.24
2204	U	0.597	0.15
2205	A	1.031	0.16
2206	A	0.99	0.18
2207	A	1.031	0.18
2208	G	0.99	0.18
2209	A	1.031	0.22
2210	A	1.438	0.41
2211	U	0.597	0.72
2212	A	0.732	0.58
2213	C	0.244	0.53
2214	A	1.044	0.46
2215	A	0.76	0.1
2216	A	1.017	0.09
2217	A	0.909	0.1
2218	A	1.044	0.32
2219	U	0.488	0.87
2220	G	0.461	0.73
2221	U	0.285	0.77
2222	A	0.895	0.55
2223	G	1.044	0.12
2224	A	0.99	0.1
2225	A	0.882	0.13
2226	A	0.963	0.37
2227	U	0.787	0.63
2228	A	1.356	0.44
2229	G	1.072	0.18
2230	A	1.234	0.23
2231	A	1.18	0.25
2232	G	0.705	0.49
2233	U	0.434	0.55

2234	U	0.393	0.15
2235	U	0.76	0.19
2236	U	0.977	0.25
2237	A	1.058	0.18
2238	G	0.515	0.06
2239	G	0.543	0.1
2240	C	0.434	0.46
2241	A	0.949	0.47
2242	A	0.868	0.15
2243	A	1.221	0.17
2244	A	1.248	0.16
2245	G	0.949	0.12
2246	G	0.76	0.09
2247	A	0.855	0.16
2248	U	1.004	0.81
2249	U	1.573	0.78
2250	A	1.533	0.25
2251	A	1.37	0.09
2252	A	1.465	0.04
2253	G	0.298	0.04
2254	G	0.231	0.05
2255	G	0.244	0.05
2256	A	0.42	0.06
2257	C	0.326	0.09
2258	A	0.678	0.11
2259	A	0.61	0.15
2260	U	0.543	0.73
2261	C	0.692	0.74
2262	A	1.37	0.25
2263	U	0.732	0.22
2264	G	1.289	0.17
2265	A	1.356	0.17
2266	C	0.787	0.17
2267	A	1.451	0.18
2268	G	0.515	0.19
2269	G	0.068	0.18
2270	G	0.041	0.14
2271	G	0.095	0.14
2272	A	0.298	0.14
2273	C	0.122	0.14
2274	A	0.231	0.14
2275	C	0.054	0.14
2276	C	0.027	0.15

2277	C	0.014	0.2
2278	C	0.122	0.21
2279	G	0.773	0.22
2280	A	1.058	0.13
2281	U	0.637	0.1
2282	U	1.031	0.05
2283	A	0.787	0.09
2284	A	0.57	0.11
2285	C	0.42	0.08
2286	A	0.393	0.06
2287	U	0.353	0.14
2288	U	0.203	0.13
2289	U	0.258	0.39
2290	U	0.448	0.75
2291	U	0.732	0.91
2292	G	0.8	0.77
2293	G	1.139	0.75
2294	U	0.99	0.32
2295	A	1.397	0.53
2296	G	1.628	0.49
2297	A	1.112	0.27
2298	A	0.895	0.41
2299	A	0.624	0.3
2300	U	0.393	0.11
2301	U	0.448	0.18
2302	U	0.271	0.27
2303	G	0.136	0.48
2304	C	0.095	0.79
2305	U	0.434	0.8
2306	A	0.732	0.6
2307	A	1.004	0.5
2308	C	0.705	0.05
2309	A	0.787	0.05
2310	G	0.692	0.03
2311	C	0.488	0.05
2312	U	0.38	0.04
2313	C	0.312	0.46
2314	U	0.515	0.63
2315	G	0.122	0.58
2316	G	0.014	0.53
2317	G	0	0.16
2318	G	0.041	0.06
2319	A	1.126	0.17

2320	U	0.136	0.23
2321	G	0.136	0.21
2322	U	0.109	0.08
2323	C	0.109	0.04
2324	U	0.271	0.1
2325	C	0.217	0.59
2326	U	0.841	0.69
2327	A	1.533	0.71
2328	A	1.058	0.58
2329	A	0.882	0.43
2330	U	0.461	0.32
2331	U	0.231	0.81
2332	U	0.19	0.68
2333	U	0.095	0.28
2334	C	0.054	0.48
2335	C	0.041	0.53
2336	C	0.109	0.54
2337	A	0.502	0.38
2338	U	0.597	0.22
2339	A	0.624	0.28
2340	G	0.298	0.22
2341	C	0.109	0.18
2342	U	1.031	0.25
2343	A	1.377	0.36
2344	A	1.234	0.39
2345	A	1.268	0.26
2346	G	0.692	0.05
2347	U	0.977	0.96
2348	A	1.194	0.94
2349	G	0.936	0.02
2350	A	0.99	0.01
2351	G	0.264	0.01
2352	C	0.136	0.01
2353	C	0.17	0.01
2354	U	0.041	0.02
2355	G	0.956	0.02
2356	U	1.004	0.94
2357	A	1.79	0.94
2358	A	1.289	0.02
2359	A	1.39	0.02
2360	A	1.336	0.01
2361	G	0.536	0.01
2362	U	0.353	0.03

2363	C	0.38	0.03
2364	G	0.319	0
2365	C	0.136	0
2366	C	0.034	0.03
2367	U	0.448	0.74
2368	U	1.397	0.98
2369	A	1.546	0.28
2370	A	1.35	0.02
2371	A	1.194	0.01
2372	G	0.366	0.01
2373	C	0.176	0.02
2374	C	0.136	0.09
2375	A	0.583	0.33
2376	G	0.895	0.44
2377	G	0.909	0.45
2378	A	1.18	0.33
2379	A	1.329	0.14
2380	A	0.855	0.08
2381	G	0.624	0.01
2382	G	0.543	0.02
2383	A	1.18	0.02
2384	U	0.393	0.02
2385	G	0.38	0.08
2386	G	0.285	0.28
2387	A	1.004	0.27
2388	C	0	0.92
2389	C	0.271	0.95
2390	A	0.732	0.89
2391	A	0.787	0.02
2392	A	0.922	0.02
2393	A	0.719	0.01
2394	U	0.637	0.03
2395	U	0.434	0.03
2396	G	0.651	0
2397	A	1.139	0.01
2398	A	0.895	0.01
2399	G	0.366	0
2400	C	0.339	0
2401	A	0.298	0.02
2402	G	0.38	0.03
2403	U	0.203	0.9
2404	G	0.122	0.95
2405	G	0.109	0.92

2406	C	0.081	0.29
2407	C	0	0.26
2408	A	0.502	0.07
2409	U	0.271	0.23
2410	U	0.448	0.93
2411	A	0.868	0.77
2412	U	0.054	0.21
2413	C	0.081	0.04
2414	A	0.963	0
2415	A	0.787	0.05
2416	A	1.139	0.06
2417	A	1.383	0.07
2418	G	1.194	0.09
2419	A	1.424	0.04
2420	A	1.004	0.01
2421	A	0.963	0.01
2422	A	1.465	0.01
2423	G	0.868	0.02
2424	A	0.977	0.2
2425	U	0.678	0.78
2426	A	0.773	0.78
2427	G	0.461	0.06
2428	U	0.42	0
2429	U	0.258	0.01
2430	G	0.543	0.01
2431	C	0.027	0.02
2432	A	0.597	0.02
2433	U	0.461	0.14
2434	U	0.637	0.18
2435	A	1.302	0.06
2436	A	1.126	0.05
2437	G	1.017	0.04
2438	A	0.963	0.02
2439	G	0.624	0.02
2440	A	0.841	0.01
2441	A	0.936	0
2442	A	0.475	0.03
2443	U	0.149	0.19
2444	C	0.041	0.24
2445	U	0	0.83
2446	G	0.434	0.81
2447	U	0.163	0.8
2448	G	0.827	0.04

2449	A	0.855	0
2450	A	0.895	0
2451	A	1.072	0
2452	A	0.773	0.02
2453	G	0.502	0.02
2454	A	0.515	0.21
2455	U	0.231	0.22
2456	G	0.488	0.11
2457	G	1.112	0.09
2458	A	1.641	0.1
2459	A	0.99	0.61
2460	A	1.031	0.77
2461	A	1.465	0.76
2462	G	0.393	0.09
2463	G	0.271	0.07
2464	A	0.38	0.05
2465	U	0.095	0.01
2466	G	0.027	0.01
2467	G	0.054	0.01
2468	U	0.081	0
2469	C	0.068	0.02
2470	A	0.488	0.02
2471	G	0.326	0.01
2472	U	0.366	0.05
2473	U	0.244	0.06
2474	G	0.176	0.02
2475	G	0.244	0.02
2476	A	0.19	0.01
2477	G	0.081	0
2478	G	0.271	0
2479	A	0.475	0.01
2480	A	0.434	0.01
2481	G	0.543	0.01
2482	C	0.054	0
2483	U	0.027	0
2484	C	0.014	0
2485	C	0.041	0
2486	C	0.041	0.02
2487	C	0.081	0.02
2488	C	0.122	0.02
2489	G	0.081	0.03
2490	A	0.136	0.02
2491	C	0	0.02

2492	C	0	0.2
2493	A	0.407	0.2
2494	A	0.624	0.01
2495	U	0.122	0
2496	C	0	0
2497	C	0	0.01
2498	A	0.556	0.01
2499	U	0.231	0.19
2500	A	1.031	0.19
2501	C	0	0
2502	A	0.515	0
2503	A	0.475	0
2504	C	0.163	0
2505	A	0.109	0
2506	C	0.041	0
2507	C	0.027	8.26E-05
2508	C	0.014	0
2509	C	0.014	0
2510	C	0	0.01
2511	A	0.773	0.01
2512	C	0	0
2513	A	0.38	0.01
2514	U	0.217	0.14
2515	U	0.339	0.16
2516	U	0.149	0.04
2517	G	0.217	0.02
2518	C	0.081	0
2519	U	0	0
2520	A	0.353	0
2521	U	0.502	0
2522	A	1.126	0.02
2523	A	0.949	0.12
2524	A	0.949	0.11
2525	G	1.058	0.02
2526	A	1.18	0.03
2527	A	1.004	0.02
2528	A	0.936	0.02
2529	A	0.977	0.01
2530	A	0.936	0.01
2531	G	0.732	0
2532	G	0.868	0
2533	A	0.963	0
2534	U	0	0.01

2535	A	1.356	0.01
2536	A	1.166	0
2537	G	1.424	0
2538	A	1.044	0.01
2539	A	0.502	0.01
2540	C	0	0.01
2541	A	0.732	0.02
2542	A	0.705	0.01
2543	A	0.597	0.07
2544	U	0.136	0.09
2545	G	0.237	0.03
2546	G	0.502	0.02
2547	A	0.651	0.03
2548	G	0.57	0.03
2549	A	0.78	0.01
2550	A	0.855	0.1
2551	U	0.454	0.32
2552	G	0.285	0.32
2553	C	0.122	0.44
2554	U	0.373	0.47
2555	G	0.604	0.18
2556	A	0.773	0.1
2557	U	0.617	0.52
2558	A	1.166	0.54
2559	G	0.766	0.48
2560	A	0.956	0.19
2561	U	0.543	0.66
2562	U	0.475	0.79
2563	U	0.509	0.43
2564	U	0.678	0.18
2565	A	0.705	0.21
2566	G	0.373	0.2
2567	G	0.359	0.09
2568	G	0.8	0.3
2569	A	0.678	0.62
2570	A	0.631	0.64
2571	C	0.183	0.94
2572	U	0.76	0.94
2573	A	0.787	0.71
2574	A	0.787	0.46
2575	A	0.97	0.43
2576	U	0.895	0.36
2577	A	0.807	0.49

2578	G	0.427	0.43
2579	G	0.088	0.25
2580	G	0.054	0.07
2581	U	0.041	0.17
2582	C	0.068	0.15
2583	A	0.38	0.08
2584	C	0.109	0.07
2585	U	0.597	0.02
2586	C	0.373	0.02
2587	A	1.465	0.01
2588	G	0.142	0.01
2589	G	0.122	0.12
2590	A	0.149	0.19
2591	C	0.081	0.23
2592	U	0.068	0.27
2593	U	0.305	0.23
2594	U	2.109	0
2595	A	1.207	0.01
2596	C	0.339	0.02
2597	G	0.793	0.15
2598	G	1.289	0.14
2599	A	0.217	0.19
2600	A	0.115	0.59
2601	G	0.034	0.82
2602	U	0.02	0.87
2603	C	0	0.85
2604	C	0.529	0.67
2605	A	0.543	0.14
2606	A	0.665	0.76
2607	U	0.556	0.8
2608	U	0.671	0.82
2609	A	1.282	0.33
2610	G	0.617	0.82
2611	G	0.441	0.78
2612	A	0.522	0.1
2613	A	0.766	0.01
2614	U	0.488	0.1
2615	A	0.597	0.13
2616	C	0.075	0.17
2617	C	0.156	0.17
2618	A	0.434	0.02
2619	C	0.19	0.01
2620	A	0.366	0.09

2621	C	0.068	0.44
2622	C	0.041	0.63
2623	C	0.034	0.46
2624	U	0.047	0.84
2625	G	0.264	0.85
2626	C	0.081	0.74
2627	A	0.617	0.14
2628	G	0.637	0.67
2629	G	0.61	0.73
2630	A	0.353	0.71
2631	C	0.068	0.78
2632	U	0.081	0.55
2633	A	0.339	0.06
2634	G	0.149	0.04
2635	C	0.142	0.01
2636	A	1.017	0.01
2637	A	0.373	0.02
2638	A	0.78	0.03
2639	A	0.827	0.04
2640	A	0.861	0.02
2641	G	0.719	0.01
2642	G	0.841	0.01
2643	A	1.017	0.02
2644	A	0.848	0.03
2645	A	0.936	0.39
2646	A	0.943	0.84
2647	G	0.848	0.78
2648	A	0.936	0.15
2649	A	0.793	0.53
2650	U	0.502	0.43
2651	U	0.529	0.09
2652	A	1.234	0.03
2653	C	0.109	0.03
2654	A	0.522	0.09
2655	G	0.271	0.26
2656	U	0.149	0.29
2657	A	0.163	0.18
2658	C	0.027	0.28
2659	U	0.041	0.37
2660	G	0.095	0.21
2661	G	0.231	0.13
2662	A	0.448	0.43
2663	U	0.176	0.53

2664	A	0.99	0.16
2665	U	0.543	0.7
2666	A	0.895	0.76
2667	G	0.258	0.77
2668	G	0.149	0.61
2669	U	0.068	0.04
2670	G	0.339	0.05
2671	A	0.515	0.21
2672	U	0.19	0.23
2673	G	0.461	0.05
2674	C	0.068	0.6
2675	A	0.57	0.75
2676	U	0	0.83
2677	A	0.963	0.69
2678	U	0.407	0.46
2679	U	0.231	0.62
2680	U	0.163	0.5
2681	C	0.081	0.12
2682	U	0.122	0.17
2683	C	0.041	0.18
2684	C	0	0.28
2685	A	0.407	0.67
2686	U	0.203	0.65
2687	A	0.556	0.2
2688	C	0.054	0.03
2689	C	0.095	0.02
2690	U	0.081	0.01
2691	C	0.136	0.12
2692	U	0.068	0.13
2693	A	0.882	0.05
2694	G	0.19	0.19
2695	A	0.203	0.73
2696	U	0.176	0.75
2697	G	0.217	0.37
2698	A	0.475	0.18
2699	A	0.678	0.09
2700	G	0.393	0.14
2701	A	0.42	0.18
2702	A	0.434	0.18
2703	U	0.285	0.35
2704	U	0.285	0.34
2705	U	0.095	0.04
2706	A	0.095	0.06

2707	G	0.014	0.06
2708	G	0.014	0.02
2709	C	0	0.02
2710	A	0.014	0.48
2711	G	0.041	0.61
2712	U	0.665	0.39
2713	A	0.827	0.09
2714	C	0.095	0.05
2715	A	0.624	0.04
2716	C	0.041	0.06
2717	U	0	0.1
2718	G	0.027	0.12
2719	C	0.054	0.1
2720	C	0.068	0.41
2721	U	0.095	0.48
2722	U	0.393	0.43
2723	U	0	0.17
2724	A	0.366	0.03
2725	C	0.163	0
2726	U	0.136	0.01
2727	U	0.38	0.97
2728	U	0.041	0.97
2729	A	0.773	0.04
2730	C	0.109	0.01
2731	C	0	0.02
2732	A	0.163	0.03
2733	U	0.109	0.03
2734	C	0	0.04
2735	A	0.665	0.95
2736	G	0.57	0.95
2737	U	0.122	0.02
2738	A	0.814	0.02
2739	A	0.556	0.01
2740	A	0.448	0.01
2741	U	0.393	0
2742	A	0.855	0.06
2743	A	0.773	0.64
2744	U	0.624	0.98
2745	G	0.027	0.51
2746	C	0	0.14
2747	A	0.095	0.12
2748	G	0.136	0.05
2749	A	0.136	0

2750	G	0.014	0
2751	C	0.014	0
2752	C	0	0.27
2753	A	0.326	0.28
2754	G	0.57	0.02
2755	G	0.637	0.04
2756	A	0.773	0.04
2757	A	0.624	0.03
2758	A	0.556	0.01
2759	A	0.624	0.01
2760	C	0.312	0.01
2761	G	0.515	0.03
2762	A	0.719	0.5
2763	U	0.38	0.61
2764	A	0.841	0.12
2765	C	0.122	0.12
2766	A	0.61	0.12
2767	U	0.339	0.09
2768	U	0.461	0
2769	U	0.407	0
2770	A	1.275	0
2771	U	0.665	0
2772	A	0.949	0
2773	A	1.18	0
2774	G	0.095	0
2775	G	0.054	0
2776	U	0.041	0
2777	U	0.041	0.05
2778	C	0.014	0.09
2779	U	0.027	0.09
2780	G	0.041	0.01
2781	C	0.095	0.31
2782	C	0.366	0.31
2783	U	0.081	0.01
2784	C	0.258	0.01
2785	A	0.773	0
2786	G	0.475	0.4
2787	G	0.434	0.4
2788	G	0.298	0.36
2789	A	0.461	0.03
2790	U	0.19	0.41
2791	G	0.176	0.45
2792	G	0.163	0.55

2793	A	0.366	0.51
2794	A	0.407	0.52
2795	G	0.258	0.44
2796	G	0.068	0.39
2797	G	0.041	0.39
2798	G	0.081	0.27
2799	U	0.122	0.29
2800	C	0.136	0.04
2801	A	0.488	0.01
2802	C	0.068	0.13
2803	C	0.095	0.14
2804	A	0.434	0.25
2805	G	0.203	0.25
2806	C	0.041	0.01
2807	C	0.014	0.05
2808	A	0.353	0.28
2809	U	0.203	0.39
2810	C	0.027	0.66
2811	U	0.041	0.85
2812	U	0.054	0.69
2813	C	0.014	0.61
2814	C	0	0.61
2815	A	0.38	0.36
2816	A	0.678	0.01
2817	U	0.448	0.1
2818	A	0.909	0.09
2819	C	0.054	0.01
2820	A	0.61	0.01
2821	C	0.163	0.01
2822	U	0.068	0.01
2823	A	0.705	0.02
2824	U	0.231	0.24
2825	G	0.543	0.25
2826	A	0.448	0.23
2827	G	0.475	0.21
2828	A	0.312	0.19
2829	C	0.041	0.01
2830	A	0.502	0.02
2831	U	0.298	0.02
2832	G	0.203	0.03
2833	U	0.136	0.68
2834	G	0.258	0.71
2835	C	0.027	0.77

2836	U	0.095	0.65
2837	A	0.475	0.28
2838	G	0.597	0.35
2839	A	0.502	0.26
2840	A	0.258	0
2841	C	0.041	0
2842	C	0.014	0.01
2843	C	0.068	0.08
2844	U	0.203	0.27
2845	U	0.271	0.38
2846	C	0.068	0.21
2847	A	0.977	0.46
2848	G	0.814	0.53
2849	G	0.936	0.56
2850	A	0.936	0.52
2851	A	1.092	0.13
2852	G	0.59	0.05
2853	G	0.461	0.03
2854	C	0.57	0.01
2855	A	0.814	0.02
2856	A	0.882	0.05
2857	A	0.922	0.03
2858	U	0.298	0
2859	C	0.244	0
2860	C	0.292	0
2861	A	0.59	0.02
2862	G	0.604	0.8
2863	A	0.868	0.99
2864	U	0.414	0.92
2865	G	0.502	0.71
2866	U	0.346	0.41
2867	G	0.637	0.34
2868	A	0.8	0.27
2869	C	0.115	0.2
2870	C	0.17	0.16
2871	U	0.515	0.15
2872	U	1.038	0.3
2873	A	2.028	0.6
2874	G	0.38	0.94
2875	U	0.197	0.96
2876	C	0.197	0.89
2877	C	0.102	0.59
2878	A	0.244	0.59

2879	G	0.42	0.6
2880	U	0.983	0.02
2881	A	1.051	0.01
2882	U	0.658	0.03
2883	A	1.716	0.19
2884	U	0.237	0.71
2885	G	0.359	0.77
2886	G	0.237	0.63
2887	A	0.488	0.64
2888	U	0.244	0.65
2889	G	0.902	0.61
2890	A	0.631	0.53
2891	C	0.19	0.48
2892	A	0.407	0.55
2893	U	0.136	0.6
2894	C	0.149	0.3
2895	U	0.387	0.56
2896	U	1.139	0.63
2897	A	1.255	0.32
2898	A	1.078	0.1
2899	U	0.827	0.14
2900	A	1.058	0.06
2901	G	0.312	0.04
2902	C	0.17	0.01
2903	U	0.407	0.29
2904	A	0.977	0.56
2905	G	0.461	0.49
2906	U	0.203	0.01
2907	G	0.644	0.03
2908	A	1.072	0.03
2909	C	0.217	0.04
2910	A	0.285	0.41
2911	G	0.231	0.46
2912	G	0.231	0.33
2913	A	0.997	0.06
2914	C	0.359	0.02
2915	A	0.875	0.01
2916	G	1.031	0.01
2917	A	0.855	0.03
2918	C	0.081	0.34
2919	C	0.068	0.54
2920	U	0	0.47
2921	G	0.176	0.09

2922	G	0.461	0.03
2923	A	0.78	0.01
2924	A	0.814	0.02
2925	C	0.258	0.05
2926	A	1.316	0.06
2927	U	0.488	0.08
2928	G	0.895	0.05
2929	A	1.119	0.01
2930	C	0.387	0.01
2931	A	0.597	0.01
2932	G	0.326	0.02
2933	G	0.251	0.03
2934	G	0.19	0.05
2935	U	0.312	0.75
2936	A	1.383	0.93
2937	G	0.298	0.91
2938	U	0.353	0.68
2939	U	0.373	0.03
2940	U	0.556	0.03
2941	U	0.699	0.03
2942	A	0.895	0.01
2943	C	0.312	0.01
2944	A	0.855	0.03
2945	G	0.42	0.67
2946	U	0.326	0.89
2947	C	0.448	0.91
2948	A	1.072	0.73
2949	A	1.058	0.02
2950	A	1.18	0.01
2951	G	0.488	0.05
2952	G	0.461	0.06
2953	A	0.583	0.07
2954	A	0.298	0.07
2955	C	0.068	0.03
2956	U	0.122	0.23
2957	C	0.054	0.39
2958	U	0.271	0.59
2959	U	0.556	0.67
2960	G	1.858	0.58
2961	A	1.478	0.04
2962	A	1.465	0.01
2963	U	0.583	0.01
2964	A	1.275	0.04

2965	G	0.895	0.05
2966	C	0	0.02
2967	A	1.18	0.03
2968	U	0.665	0.58
2969	A	1.004	0.76
2970	G	0.244	0.79
2971	G	0.244	0.5
2972	G	0.041	0.3
2973	U	0.041	0.22
2974	U	0.502	0.24
2975	U	0.76	0.31
2976	U	0.515	0.27
2977	C	0.176	0.07
2978	U	0.149	0.02
2979	A	0.285	0.01
2980	C	0.122	0.01
2981	C	0.081	0.01
2982	C	0.136	0.03
2983	C	0.149	0.03
2984	A	0.773	0.07
2985	G	0.841	0.09
2986	A	0.963	0.07
2987	A	0.963	0.02
2988	G	0.827	0.11
2989	A	0.936	0.11
2990	G	0.773	0.13
2991	A	1.072	0.04
2992	A	1.221	0.17
2993	A	0.719	0.21
2994	U	0.298	0.25
2995	U	0.38	0.29
2996	C	0	0.26
2997	C	0	0.21
2998	A	1.031	0.13
2999	A	1.139	0.02
3000	A	1.166	0.04
3001	A	1.112	0.05
3002	A	1.234	0.05
3003	G	1.058	0.03
3004	A	1.18	0.01
3005	U	0.081	0.31
3006	C	0.122	0.34
3007	C	0.014	0.29

3008	C	0.027	0.15
3009	C	0.014	0.39
3010	C	0.054	0.4
3011	A	0.231	0.37
3012	U	0.19	0.12
3013	U	0.583	0.01
3014	U	1.356	0.02
3015	C	1.261	0.07
3016	A	1.316	0.1
3017	A	1.044	0.23
3018	U	0.692	0.89
3019	G	0.61	0.87
3020	G	0.393	0.81
3021	A	0.271	0.54
3022	U	0.136	0.45
3023	G	0.109	0.5
3024	G	0.041	0.47
3025	G	0.054	0.31
3026	G	0.054	0.04
3027	U	0.271	0.22
3028	A	0.977	0.43
3029	C	0.298	0.44
3030	G	0.814	0.18
3031	A	0.827	0.14
3032	A	0.936	0.17
3033	U	0.61	0.4
3034	U	0.42	0.46
3035	G	0.285	0.35
3036	U	0.081	0.45
3037	G	0.122	0.25
3038	G	0.136	0.18
3039	C	0.217	0.75
3040	C	0.163	0.8
3041	A	0.353	0.75
3042	A	0.109	0.06
3043	C	0	0.02
3044	A	0.895	0.01
3045	A	0.827	0.03
3046	A	0.678	0.08
3047	A	0.773	0.1
3048	U	0.231	0.52
3049	G	0.488	0.51
3050	G	0.529	0.44

3051	A	0.76	0.07
3052	A	0.719	0.27
3053	G	0.583	0.52
3054	U	0.353	0.65
3055	U	0.326	0.67
3056	G	0.122	0.21
3057	C	0	0.08
3058	A	0.909	0.08
3059	A	0.583	0.02
3060	A	1.058	0.07
3061	A	1.085	0.08
3062	G	1.099	0.26
3063	A	1.207	0.39
3064	U	0.868	0.45
3065	A	0.719	0.29
3066	G	0.258	0.29
3067	A	0.244	0.28
3068	G	0.014	0.06
3069	U	0.027	0.25
3070	U	0.014	0.26
3071	G	0.054	0.06
3072	C	0.027	0.1
3073	C	0.068	0.1
3074	A	0.366	0.02
3075	C	0.054	0.01
3076	A	0.597	0.01
3077	A	0.475	0.02
3078	A	0.841	0.09
3079	G	1.031	0.1
3080	A	0.827	0.12
3081	G	0.868	0.12
3082	A	0.977	0.02
3083	G	0.868	0.03
3084	A	0.515	0.05
3085	C	0	0.09
3086	C	0.014	0.48
3087	U	0.095	0.98
3088	G	0.366	0.87
3089	G	0.244	0.58
3090	A	0.339	0.51
3091	C	0.027	0.64
3092	A	0.515	0.66
3093	G	0.597	0.14

3094	U	0.326	0.15
3095	G	0.678	0.06
3096	A	0.665	0.04
3097	A	0.787	0.14
3098	U	0.366	0.74
3099	G	0.637	0.67
3100	A	0.949	0.09
3101	U	0.312	0.37
3102	A	1.153	0.37
3103	U	0.366	0.8
3104	A	0.773	0.57
3105	C	0.122	0.6
3106	A	0.787	0.58
3107	G	0.76	0.01
3108	A	0.99	0.01
3109	A	0.8	0.01
3110	G	0.597	0
3111	U	0.556	0.2
3112	U	0.339	0.91
3113	A	1.017	0.94
3114	G	0.936	0.94
3115	U	0.231	0.91
3116	A	0.949	0.9
3117	G	0.461	0.07
3118	G	0.624	0.02
3119	A	0.705	0.01
3120	G	0.61	0.02
3121	U	0.264	0.85
3122	A	0.99	0.86
3123	U	0.509	0.99
3124	U	0.875	0.99
3125	A	1.519	0.93
3126	A	1.35	0.23
3127	A	0.848	0.06
3128	U	0.658	0.27
3129	U	0.482	0.96
3130	G	0.475	0.96
3131	G	0.251	0.94
3132	G	0.251	0.71
3133	C	0.149	0.02
3134	A	0.434	0.21
3135	G	0.224	0.2
3136	C	0.142	0.01

3137	U	0.183	0
3138	C	0.224	0.01
3139	A	1.051	0.01
3140	A	0.99	0
3141	A	0.855	0.02
3142	U	0.515	0.04
3143	U	0.658	0.22
3144	U	0.502	0.22
3145	A	0.631	0.02
3146	U	0.115	0.71
3147	C	0.115	0.94
3148	C	0.054	0.94
3149	A	0.414	0.96
3150	G	0.075	0.29
3151	G	0.061	0.06
3152	U	0.027	0.05
3153	A	0.617	0.08
3154	U	0.475	0.09
3155	A	1.519	0.08
3156	A	0.827	0.07
3157	A	0.99	0.07
3158	A	1.031	0.07
3159	A	0.61	0.07
3160	C	0.027	0.08
3161	C	0.122	0.29
3162	A	0.793	0.57
3163	A	0.8	0.54
3164	A	0.732	0.53
3165	C	0.231	0.61
3166	A	0.787	0.6
3167	U	0.441	0.31
3168	C	0.047	0.36
3169	U	0.136	0.4
3170	C	0.061	0.37
3171	U	0.027	0.7
3172	G	0.224	0.71
3173	U	0.142	0.59
3174	A	0.61	0.56
3175	G	0.326	0.56
3176	G	0.305	0.55
3177	U	0.244	0.97
3178	U	0.237	0.98
3179	A	1.187	0.94

3180	A	0.882	0.02
3181	U	0.624	0.03
3182	U	0.909	0.02
3183	A	0.956	0.9
3184	G	1.004	0.9
3185	A	0.977	0.02
3186	G	0.936	0.03
3187	G	0.977	0.02
3188	A	1.261	0.01
3189	A	1.207	0.01
3190	A	1.316	0.01
3191	A	1.105	0.03
3192	A	1.37	0.04
3193	U	0.4	0.04
3194	G	0.604	0.02
3195	A	0.454	0.01
3196	C	0.014	0
3197	U	0.027	0.01
3198	C	0.02	0.03
3199	U	0.509	0.03
3200	A	1.051	0
3201	A	0.549	0
3202	C	0	0.01
3203	A	0.346	0.01
3204	G	0.631	0
3205	A	1.092	0
3206	G	0.888	0.02
3207	G	0.719	0.02
3208	A	0.929	0
3209	A	1.146	0.02
3210	G	0.712	0.07
3211	U	0.088	0.07
3212	U	0.034	0.01
3213	C	0.129	0.02
3214	A	1.248	0.31
3215	G	8.477	0.4
3216	U	0.515	0.57
3217	G	0.746	0.21
3218	G	0.095	0.01
3219	A	0.136	0.01
3220	C	0.081	0.02
3221	U	0.068	0.03
3222	G	0.109	0.02

3223	A	0.068	0.02
3224	G	0	0.43
3225	A	0.095	0.55
3226	U	0.298	0.44
3227	G	0.678	0.04
3228	G	0.488	0.13
3229	C	0	0.32
3230	A	0.949	0.2
3231	G	1.072	0.01
3232	A	1.004	0.05
3233	A	1.017	0.06
3234	G	0.651	0.13
3235	C	0.081	0.13
3236	A	1.139	0.03
3237	G	0.895	0.01
3238	A	1.017	0.01
3239	A	1.112	0.03
3240	U	0	0.03
3241	A	1.492	0.03
3242	U	0.515	0.01
3243	G	1.478	0
3244	A	0.882	0
3245	G	1.329	0
3246	G	0.936	0
3247	A	1.139	0
3248	A	0.977	0
3249	A	0.99	0
3250	A	0.977	0
3251	U	0.448	0
3252	A	1.478	0.08
3253	A	1.085	0.11
3254	A	1.139	0.07
3255	A	1.18	0.41
3256	U	0.353	0.67
3257	A	1.424	0.66
3258	A	1.139	0.01
3259	U	0.461	0.05
3260	U	0.095	0.06
3261	C	0.041	0.21
3262	U	0.027	0.21
3263	C	0.054	0.01
3264	A	0.149	0.64
3265	G	0.068	0.66

3266	U	0.095	0.39
3267	C	0.081	0.02
3268	A	0.515	0.03
3269	G	0.475	0.04
3270	G	0.19	0.04
3271	A	0.217	0.03
3272	A	0.19	0.01
3273	C	0.014	0.01
3274	A	0.8	0.03
3275	A	1.031	0.04
3276	G	1.004	0.04
3277	A	1.058	0.03
3278	A	1.044	0.02
3279	G	0.705	0.02
3280	G	0.665	0.01
3281	A	0.922	0.01
3282	U	0.326	0
3283	G	0.488	0.01
3284	U	0.176	0.09
3285	U	0.136	0.19
3286	A	0.109	0.12
3287	U	0.041	0.09
3288	U	0.054	0.02
3289	A	0.095	0
3290	C	0.027	0
3291	C	0.068	0.03
3292	A	0.624	0.04
3293	A	0.841	0.12
3294	G	1.099	0.12
3295	A	1.424	0.08
3296	A	1.709	0.04
3297	G	0	0.04
3298	G	0.068	0.01
3299	C	0.027	0
3300	A	0.529	0
3301	A	0.855	0
3302	G	0.678	0
3303	C	0.203	0
3304	C	0.068	0.04
3305	A	0.583	0.97
3306	U	0.407	0.98
3307	U	0.258	0.83
3308	A	0.99	0.02

3309	G	0.787	0.01
3310	A	0.855	0.01
3311	A	0.909	0
3312	G	0.054	0
3313	C	0.041	0
3314	C	0.027	0
3315	A	0.258	0
3316	C	0.163	0.01
3317	G	0.122	0.82
3318	G	0.109	0.99
3319	U	0.041	0.97
3320	A	0.203	0.05
3321	A	0.19	0.01
3322	U	0.231	0.01
3323	A	0.651	0.01
3324	A	0.827	0.01
3325	A	0.665	0.01
3326	G	0.529	0.01
3327	A	0.38	0.02
3328	G	0.136	0.16
3329	U	0.095	0.28
3330	C	0	0.28
3331	A	0.407	0.39
3332	G	0.502	0.52
3333	G	0.556	0.24
3334	A	0.149	0.01
3335	C	0	0.02
3336	A	0.502	0.03
3337	A	0.461	0.2
3338	U	0.163	0.61
3339	C	0	0.42
3340	A	0.285	0
3341	G	0.61	0.03
3342	U	0.163	0.29
3343	G	0.312	0.28
3344	G	0.027	0.62
3345	U	0.027	0.51
3346	C	0	0.09
3347	U	0.149	0.32
3348	U	0.312	0.42
3349	A	1.153	0.17
3350	U	0.475	0.02
3351	A	0.99	0.02

3352	A	0.651	0
3353	A	0.678	0
3354	A	0.624	0
3355	U	0.407	0.93
3356	U	0.339	0.94
3357	C	0.041	0.7
3358	A	0.448	0
3359	C	0.068	0
3360	C	0	0
3361	A	0.583	0.01
3362	A	0.665	0.04
3363	G	0.732	0.73
3364	A	0.298	0.79
3365	A	0.271	0.73
3366	G	0.203	0.15
3367	A	0.149	0.14
3368	C	0.068	0
3369	A	0.732	0.01
3370	A	0.61	0.01
3371	A	0.827	0.01
3372	A	0.895	0.05
3373	U	1.044	0.16
3374	A	0.434	0.53
3375	C	0.081	0.84
3376	U	0.136	1
3377	G	0.271	0.98
3378	A	0.366	0
3379	A	0.515	0
3380	A	0.515	0
3381	G	0.285	0
3382	U	0	0.97
3383	A	0.807	0.99
3384	G	0.76	0.84
3385	G	0.298	0.53
3386	A	0.678	0.17
3387	A	0.685	0.06
3388	A	0.454	0.07
3389	A	0.475	0.17
3390	U	0.244	0.45
3391	U	0.448	0.41
3392	U	0.271	0.5
3393	G	0.224	0.26
3394	C	0.176	0.02

3395	A	0.834	0.01
3396	A	0.502	0.02
3397	A	0.536	0.07
3398	G	0.59	0.08
3399	A	0.359	0.57
3400	U	0.583	0.61
3401	A	0.936	0.12
3402	A	0.875	0.12
3403	A	1.092	0.12
3404	G	0.8	0.13
3405	A	0.895	0.05
3406	A	0.855	0.43
3407	U	0.4	0.49
3408	A	0.787	0.12
3409	C	0.041	0.02
3410	A	0.509	0.02
3411	C	0.142	0.15
3412	A	0.726	0.16
3413	U	0.366	0.12
3414	A	0.482	0.1
3415	C	0.054	0.01
3416	C	0.068	0.02
3417	A	0.353	0.01
3418	A	0.61	0
3419	U	0.441	0
3420	G	0.576	0.01
3421	G	0.624	0.01
3422	A	0.549	0.03
3423	G	0.346	0.03
3424	U	0.136	0.02
3425	G	0.543	0
3426	A	0.427	0.01
3427	G	0.59	0.34
3428	A	0.495	0.74
3429	C	0.088	0.85
3430	U	0.156	0.91
3431	A	0.665	0.94
3432	U	0.319	0.86
3433	U	0.298	0.29
3434	A	0.543	0.05
3435	G	0.353	0.01
3436	C	0.047	0.01
3437	A	0.387	0.01

3438	C	0.088	0.01
3439	A	0.766	0
3440	U	0.244	0.22
3441	G	0.549	0.81
3442	U	0.332	0.95
3443	A	1.126	0.95
3444	A	1.051	0.87
3445	U	0.576	0.78
3446	A	1.017	0.34
3447	C	0.17	0.01
3448	A	1.282	0
3449	G	0.793	0
3450	A	1.329	0.01
3451	A	1.261	0.03
3452	A	1.01	0.03
3453	A	0.712	0.05
3454	U	0.461	0.43
3455	A	1.411	0.82
3456	G	0.956	0.84
3457	G	0.882	0.64
3458	A	1.187	0.07
3459	A	1.322	0.05
3460	A	1.275	0.05
3461	G	0.834	0.08
3462	G	1.024	0.11
3463	A	1.119	0.05
3464	A	1.166	0.04
3465	G	0.529	0.04
3466	C	0.339	0.05
3467	A	0.76	0.59
3468	A	0.963	0.82
3469	U	0.922	0.83
3470	A	1.051	0.43
3471	G	0.637	0.09
3472	U	0.529	0.13
3473	G	0.651	0.15
3474	A	0.916	0.65
3475	U	0.278	0.84
3476	C	0.407	0.98
3477	U	0.556	0.94
3478	G	0.393	0.95
3479	G	0.231	0.96
3480	G	0.068	0.76

3481	G	0.38	0.27
3482	A	0.678	0.09
3483	C	0.068	0
3484	A	1.085	0
3485	G	0.705	0.01
3486	G	0.407	0.01
3487	U	0.203	0.08
3488	C	0.461	0.09
3489	C	0.054	0.88
3490	C	1.451	0.94
3491	A	0.963	0.83
3492	A	1.085	0.89
3493	A	1.356	0.93
3494	A	1.139	0.83
3495	U	0.488	0.76
3496	U	0.122	0.17
3497	C	0.122	0.08
3498	C	0.109	0.05
3499	A	0.244	0.02
3500	C	0.068	0.11
3501	U	0.529	0.19
3502	U	0.787	0.21
3503	A	0.692	0.13
3504	C	0.19	0.06
3505	C	0.407	0.07
3506	A	1.207	0.06
3507	G	0.57	0.04
3508	U	0.936	0.09
3509	U	0.814	0.09
3510	G	1.044	0.07
3511	A	1.031	0.01
3512	G	0.949	0.01
3513	A	1.79	0.02
3514	A	1.994	0.02
3515	G	0.475	0.02
3516	G	0.366	0.01
3517	A	0.637	0
3518	U	0.312	0
3519	G	0.665	0.02
3520	U	0.393	0.03
3521	A	1.099	0.41
3522	U	0.312	0.83
3523	G	0.353	0.82

3524	G	0.326	0.71
3525	G	0.556	0.67
3526	A	0.855	0.63
3527	A	1.017	0.4
3528	C	0.719	0.82
3529	A	0.909	0.76
3530	G	1.383	0.71
3531	U	0.326	0.81
3532	G	0.203	0.83
3533	G	0.339	0.92
3534	U	0.326	0.88
3535	G	0.258	0.06
3536	G	0.448	0.05
3537	A	0.922	0.01
3538	C	0.434	0
3539	A	1.356	0
3540	G	1.126	0.01
3541	A	1.383	0.78
3542	C	0.326	0.89
3543	U	1.126	0.93
3544	A	1.261	0.91
3545	U	0.583	0.77
3546	U	0.732	0.74
3547	G	0.936	0.46
3548	G	1.139	0.02
3549	C	0.515	0.43
3550	A	1.709	0.43
3551	G	0.231	0.14
3552	G	0.244	0.12
3553	U	0.298	0.11
3554	A	0.624	0.08
3555	A	0.855	0.07
3556	C	0.19	0.09
3557	C	0.109	0.07
3558	U	0.231	0.44
3559	G	0.339	0.44
3560	G	0.556	0.32
3561	A	0.746	0.4
3562	U	0.597	0.88
3563	A	0.624	0.97
3564	C	0.163	0.98
3565	C	0.176	0.94
3566	G	0.827	0.67

3567	G	1.424	0.38
3568	A	1.465	0.01
3569	A	1.655	0
3570	U	0.61	0
3571	G	0.122	0.29
3572	G	0.041	0.33
3573	G	0.176	0.31
3574	A	0.624	0.25
3575	U	0.529	0.02
3576	U	0.855	0.02
3577	U	0.665	0.02
3578	U	0.977	0.02
3579	A	0.936	0.02
3580	U	0.149	0.02
3581	C	0.081	0
3582	U	0.149	0
3583	C	0.163	0
3584	A	0.787	0
3585	A	1.248	0.01
3586	C	0.665	0.01
3587	A	0.732	0.01
3588	C	0.068	0.01
3589	C	0.095	0.01
3590	A	0.298	0.02
3591	C	0.136	0.01
3592	C	0.109	0.02
3593	G	0.095	0.03
3594	C	0.027	0.23
3595	U	0.203	0.24
3596	A	0.787	0.02
3597	G	0.624	0.02
3598	U	0.597	0.01
3599	A	1.289	0.01
3600	A	1.316	0.17
3601	G	0.692	0.28
3602	A	0.773	0.26
3603	U	0.475	0.5
3604	U	0.855	0.79
3605	A	1.275	0.68
3606	G	0.298	0.36
3607	U	0.163	0.02
3608	C	0.081	0.02
3609	U	0.19	0.04

3610	U	0.312	0.03
3611	C	0.353	0.04
3612	A	1.139	0.17
3613	A	1.058	0.15
3614	U	0.312	0
3615	C	0.149	0.36
3616	U	0.298	0.6
3617	A	0.949	0.54
3618	G	0.827	0.38
3619	U	0.366	0.33
3620	G	1.004	0.15
3621	A	1.126	0.14
3622	A	1.18	0.14
3623	G	0.99	0.27
3624	G	1.031	0.28
3625	A	0.692	0.05
3626	C	0.027	0.05
3627	C	0.027	0.06
3628	C	0.027	0.04
3629	U	0.556	0.01
3630	A	1.309	0.25
3631	U	0.563	0.34
3632	A	1.974	0.12
3633	G	1.492	0.02
3634	A	1.451	0.02
3635	G	0.19	0.01
3636	G	0.163	0
3637	G	0.373	0
3638	A	1.478	0
3639	G	1.173	0
3640	A	1.662	0.03
3641	A	2.068	0.99
3642	G	0.97	0.99
3643	A	1.16	0.1
3644	A	0.977	0
3645	A	0.76	0
3646	C	0.231	0
3647	C	0.047	0.5
3648	U	0.102	0.52
3649	A	0.671	0.12
3650	U	0.353	0.49
3651	U	0.529	0.49
3652	A	1.784	0.02

3653	U	0.658	0.01
3654	A	0.99	0.01
3655	C	0.109	0.03
3656	A	1.56	0.05
3657	G	0.719	0.05
3658	A	1.051	0.03
3659	U	0.739	0
3660	G	0.719	0.01
3661	G	0.38	0
3662	A	0.339	0
3663	U	0.21	0.03
3664	C	0.007	0.03
3665	A	0.61	0.01
3666	U	0.502	0.04
3667	G	0.19	0.04
3668	U	0.129	0
3669	A	0.732	0
3670	A	0.848	7.88E-05
3671	U	0.556	0
3672	A	1.2	0
3673	A	0.997	0
3674	A	0.732	0
3675	C	0.298	1.68E-05
3676	A	1.085	0
3677	G	0.604	0
3678	U	0.237	0.78
3679	C	0.339	0.78
3680	A	1.099	0
3681	A	1.078	0
3682	A	1.16	0
3683	A	1.519	0
3684	G	0.909	0.66
3685	A	1.078	0.66
3686	A	1.37	0.12
3687	G	0.888	0.12
3688	G	0.712	0.12
3689	G	0.407	0.12
3690	A	0.576	0.12
3691	A	0.617	0.12
3692	A	1.105	0.19
3693	G	0.346	0.19
3694	C	0	0.2
3695	A	0.868	0.21

3696	G	0.644	0.17
3697	G	0.387	0.1
3698	A	0.651	0.5
3699	U	0.298	0.56
3700	A	0.875	0.21
3701	U	0.427	0.46
3702	A	0.746	0.48
3703	U	0.339	0.45
3704	C	0.068	0.05
3705	A	0.8	0.02
3706	C	0.217	0.11
3707	A	1.2	0.22
3708	G	0.624	0.19
3709	A	0.658	0.45
3710	U	0.244	0.5
3711	A	1.038	0.49
3712	G	0.427	0.23
3713	G	0.156	0.22
3714	G	0.21	0.17
3715	G	0.136	0.08
3716	C	0.19	0.08
3717	A	0.78	0.03
3718	A	0.916	0.04
3719	A	1.39	0.08
3720	G	0.882	0.06
3721	A	0.319	0.02
3722	C	0.407	0.01
3723	A	0.922	0.01
3724	A	1.038	0.01
3725	A	0.97	0.02
3726	G	0.292	0.02
3727	U	0.739	0.04
3728	A	1.56	0.02
3729	A	1.105	0.01
3730	A	1.248	0
3731	A	1.322	0
3732	G	0.766	0
3733	U	0.332	0.01
3734	G	0.387	0.03
3735	U	0.387	0.16
3736	U	0.617	0.78
3737	A	1.282	0.84
3738	G	0.909	0.57

3739	A	1.078	0.65
3740	A	0.78	0.45
3741	C	0.332	0.19
3742	A	1.051	0.23
3743	G	1.038	0.26
3744	A	0.617	0.23
3745	C	0.21	0.83
3746	U	0.264	0.91
3747	A	0.502	0.68
3748	C	0.109	0.1
3749	U	0.475	0.11
3750	A	0.658	0.11
3751	A	0.658	0.09
3752	U	0.454	0.07
3753	C	0.339	0.07
3754	A	0.909	0.08
3755	A	0.651	0.08
3756	C	0.264	0.1
3757	A	0.888	0.11
3758	A	0.875	0.25
3759	G	0.617	0.28
3760	C	0.366	0.09
3761	A	1.343	0.07
3762	G	1.044	0.07
3763	A	0.963	0.07
3764	A	0.949	0.02
3765	U	0.678	0.24
3766	U	0.637	0.84
3767	G	0.827	0.9
3768	G	0.651	0.82
3769	A	0.882	0.04
3770	A	0.882	0.04
3771	G	0.488	0.07
3772	C	0.231	0.06
3773	A	0.597	0.05
3774	U	0.231	0.86
3775	U	0.136	0.91
3776	U	0.054	0.91
3777	C	0	0.81
3778	U	0.122	0.45
3779	C	0	0.41
3780	A	0.461	0.09
3781	U	0.366	0.23

3782	G	0.136	0.21
3783	G	0	0.21
3784	C	0	0.12
3785	A	0.19	0.07
3786	U	0.19	0.26
3787	U	0.678	0.3
3788	G	0.475	0.27
3789	A	0.298	0.01
3790	C	0.285	0.01
3791	A	1.072	0.02
3792	G	0.448	0.6
3793	A	0.366	0.89
3794	C	0.203	0.91
3795	U	0.244	0.8
3796	C	0.041	0.05
3797	A	0.746	0.02
3798	G	0.597	0.03
3799	G	0.61	0.07
3800	G	0	0.06
3801	C	0	0.07
3802	C	0.19	0.03
3803	A	0.827	0.03
3804	A	1.058	0.01
3805	A	1.031	0.01
3806	G	0.231	0.75
3807	G	0.109	0.87
3808	C	0	0.88
3809	A	0.515	0.59
3810	A	0.665	0.01
3811	A	1.031	0.03
3812	U	0.231	0.04
3813	A	0.963	0.1
3814	U	0.42	0.18
3815	U	0.678	0.2
3816	A	1.438	0.22
3817	U	0.42	0.47
3818	A	1.126	0.44
3819	G	0.583	0.15
3820	U	0.407	0.15
3821	A	1.072	0.07
3822	G	0.285	0.16
3823	A	0.42	0.4
3824	U	0.258	0.47

3825	U	0.285	0.59
3826	C	0.149	0.56
3827	A	0.285	0.45
3828	C	0.326	0.47
3829	A	0.705	0.36
3830	A	0.909	0.12
3831	U	0.38	0.27
3832	A	1.085	0.22
3833	U	0.231	0.41
3834	G	0.231	0.36
3835	U	0.244	0.18
3836	U	0	0.04
3837	A	0.434	0.17
3838	U	0.176	0.75
3839	G	0.312	0.72
3840	G	0.203	0.57
3841	G	0.326	0.54
3842	A	0.366	0.5
3843	A	0.244	0.48
3844	U	0.556	0.53
3845	A	1.37	0.3
3846	A	0.882	0.2
3847	U	0.543	0.46
3848	A	1.139	0.32
3849	A	0.597	0.17
3850	C	0.068	0.18
3851	A	0.746	0.11
3852	G	0.515	0.09
3853	G	0.339	0.02
3854	A	0.746	0.04
3855	U	0.366	0.12
3856	G	0.122	0.12
3857	C	0	0.11
3858	C	0	0.23
3859	C	0.041	0.17
3860	U	0.231	0.14
3861	A	0.61	0.08
3862	C	0.136	0.12
3863	A	0.895	0.12
3864	G	0.855	0.07
3865	A	0.461	0.06
3866	A	0.678	0.05
3867	U	0.502	0.07

3868	C	0.081	0.08
3869	A	0.949	0.1
3870	G	1.031	0.1
3871	A	1.004	0.08
3872	G	1.072	0
3873	A	0.882	0
3874	G	0.57	0
3875	C	0.42	0
3876	A	0.773	0
3877	G	0.041	0
3878	G	0	0.01
3879	C	0.014	0.56
3880	U	0.014	0.97
3881	A	0.231	0.46
3882	G	0.19	0.05
3883	U	0	0.08
3884	U	0.109	0.05
3885	A	0.014	0.53
3886	A	0.054	0.5
3887	U	0	0.01
3888	C	0.014	0.01
3889	A	0.339	0.11
3890	A	0.637	0.11
3891	A	0.597	0.2
3892	U	0.583	0.3
3893	A	0.936	0.22
3894	A	0.882	0.28
3895	U	0.57	0.82
3896	A	1.261	0.76
3897	G	1.044	0.12
3898	A	1.139	0.01
3899	A	1.166	0
3900	G	1.302	0.01
3901	A	1.004	0.02
3902	A	0.732	0.1
3903	A	0.977	0.19
3904	U	0.326	0.23
3905	G	0	0.18
3906	A	0.122	0.22
3907	U	0.041	0.24
3908	U	0.244	0.16
3909	A	0.285	0.14
3910	A	0.298	0.02

3911	A	0.353	0
3912	A	0.38	0.01
3913	A	0.19	0.01
3914	G	0.041	0.01
3915	U	0.122	0.01
3916	C	0.095	0.07
3917	A	0.909	0.23
3918	G	1.112	0.21
3919	A	1.085	0.67
3920	A	0.882	0.66
3921	A	0.746	0.42
3922	U	0.529	0.24
3923	U	0.841	0.05
3924	U	0.665	0.13
3925	A	1.601	0.13
3926	U	0.57	0.24
3927	G	0.909	0.36
3928	U	0.475	0.7
3929	A	0.787	0.78
3930	G	0.407	0.4
3931	C	0.38	0.01
3932	A	0.746	0.09
3933	U	0.271	0.67
3934	G	0.176	0.71
3935	G	0	0.61
3936	G	0	0.51
3937	U	0.203	0.2
3938	A	0.624	0
3939	C	0.027	0
3940	C	0.122	0.04
3941	A	0.434	0.04
3942	G	0.475	0.19
3943	C	0.332	0.52
3944	A	0.366	0.58
3945	C	0	0.59
3946	A	0.176	0.53
3947	C	0.461	0.06
3948	A	0.922	0.04
3949	A	0.882	0.01
3950	A	1.031	0
3951	G	0.285	0.01
3952	G	0.373	0.01
3953	U	0.122	0.01

3954	A	0.99	0.01
3955	U	0.461	0.05
3956	A	1.248	0.05
3957	G	0.482	0.04
3958	G	0.543	0.04
3959	A	0.387	0.04
3960	G	0.271	0.04
3961	G	0.339	0.03
3962	A	0.509	0.02
3963	A	0.712	0.02
3964	A	0.583	0.02
3965	C	0.237	0.04
3966	C	0.149	0.04
3967	A	0.766	0.01
3968	A	0.821	0.01
3969	G	1.099	0
3970	A	1.18	0
3971	A	0.888	0.02
3972	A	1.146	0.02
3973	U	0.644	0.55
3974	A	0.977	0.57
3975	G	0.509	0.53
3976	A	0.509	0.02
3977	C	0	0.03
3978	C	0.197	0.03
3979	A	0.278	0.03
3980	C	0	0.02
3981	C	0.075	0.58
3982	U	0.393	0.63
3983	A	0.916	0.56
3984	G	0.78	0.01
3985	U	0.468	0.01
3986	U	0.773	0.03
3987	A	1.044	0.02
3988	G	0.393	0.03
3989	U	0.326	0
3990	C	0.129	0
3991	A	0.719	0
3992	A	0.8	0.01
3993	G	0.319	0.02
3994	G	0.305	0.05
3995	G	0.631	0.05
3996	A	0.699	0.05

3997	U	0.448	0.04
3998	U	0.475	0.04
3999	A	1.078	0.24
4000	G	0.719	0.24
4001	A	0.916	0.03
4002	C	0.231	0.02
4003	A	0.963	0.02
4004	A	0.997	0.02
4005	G	0.434	0.04
4006	U	0.353	0.05
4007	U	0.488	0.07
4008	C	0.339	0.31
4009	U	0.19	0.3
4010	C	0.136	0.08
4011	U	0.176	0.09
4012	U	0.285	0.07
4013	C	0.231	0.01
4014	U	0.163	0.05
4015	U	0.014	0.06
4016	G	0.068	0.04
4017	G	0.583	0
4018	A	1.777	0
4019	A	0.773	0.01
4020	A	1.275	0.01
4021	A	1.017	0.02
4022	G	0.705	0.03
4023	A	1.004	0.13
4024	U	0.57	0.21
4025	A	1.641	0.21
4026	G	1.248	0.09
4027	A	0.922	0.02
4028	G	0.773	0.02
4029	C	0.231	0.01
4030	C	0.271	0.01
4031	A	0.502	0.12
4032	G	0.678	0.16
4033	C	0.176	0.16
4034	A	1.451	0.17
4035	C	0	0.04
4036	A	0.366	0.04
4037	A	0.366	0.01
4038	G	0.543	0.01
4039	A	0.366	0

4040	A	0.488	0.04
4041	G	0.42	0.04
4042	A	0.773	0.01
4043	A	0.705	0.01
4044	C		0.02
4045	A	1.112	0.24
4046	U	0.217	0.28
4047	G	1.058	0.24
4048	A	1.831	0.03
4049	U	1.329	0.02
4050	A	2.414	0.03
4051	A	0.42	0.03
4052	A	0.461	0.07
4053	U	0.19	0.38
4054	A	0.298	0.37
4055	C	0	0.3
4056	C	0	0.25
4057	A	0	0.77
4058	U	0.298	0.85
4059	A	0.597	0.26
4060	G	0.095	0.18
4061	U	0.624	0.16
4062	A	0.963	0.15
4063	A	1.112	0.63
4064	U	0.461	0.74
4065	G	0.882	0.49
4066	U	0.977	0.57
4067	A	1.614	0.45
4068	A	1.058	0.09
4069	A	1.329	0.08
4070	A	0.949	0.09
4071	G	0.909	0.08
4072	A	1.004	0.05
4073	A	1.112	0.05
4074	U	0.42	0.2
4075	U	0.163	0.21
4076	G	0.014	0.05
4077	G	0.258	0
4078	U	0.176	0.01
4079	A	0.339	0.05
4080	U	0.122	0.85
4081	U	1.194	0.87
4082	C	0.787	0.65

4083	A	1.356	0.45
4084	A	1.112	0.1
4085	A	0.651	0.48
4086	U	0.353	0.72
4087	U	0.556	0.69
4088	U	0.244	0.75
4089	G	0.231	0.74
4090	G	0.651	0.25
4091	A	0.556	0.17
4092	U	0.583	0.45
4093	U	0.448	0.43
4094	A	0.285	0.12
4095	C	0.326	0.18
4096	C	0	0.14
4097	C	0.095	0.04
4098	A	0.543	0.11
4099	G	0.76	0.45
4100	A	1.126	0.52
4101	A	1.139	0.83
4102	U	0.353	0.8
4103	A	1.451	0.21
4104	G	0.827	0.17
4105	U	0.434	0.27
4106	G	0.149	0.15
4107	G	0.203	0.04
4108	C	0.081	0.05
4109	C	0.285	0.11
4110	A	0.855	0.55
4111	G	0.855	0.5
4112	A	0.583	0.22
4113	C	0.068	0.6
4114	A	1.126	0.55
4115	G	0.732	0.56
4116	A	0.936	0.6
4117	U	0.407	0.68
4118	A	1.234	0.29
4119	G	0.827	0.23
4120	U	0.787	0.62
4121	A	1.37	0.59
4122	G	0.692	0.24
4123	A	1.044	0.3
4124	C	0.529	0.22
4125	A	0.502	0.07

4126	C	0.217	0.1
4127	C	0.244	0.15
4128	U	0.244	0.42
4129	G	0.922	0.57
4130	U	0.326	0.4
4131	G	0.543	0.11
4132	A	0.868	0.04
4133	U	0.597	0.01
4134	A	1.397	0.06
4135	A	1.261	0.21
4136	A	0.827	0.48
4137	U	0.434	0.87
4138	G	0.339	0.78
4139	U	0.176	0.04
4140	C	0.163	0.03
4141	A	0.57	0.02
4142	U	0.42	0.01
4143	C	0	0.11
4144	A	1.072	0.11
4145	G	1.221	0.02
4146	A	1.424	0.03
4147	A	1.275	0.02
4148	A	1.451	0.02
4149	G	0.787	0.03
4150	G	0.746	0.03
4151	A	0.773	0.01
4152	G	0.922	0.03
4153	A	1.356	0.03
4154	G	0.326	0.03
4155	G	0.224	0.05
4156	C	0	0.21
4157	U	0.319	0.23
4158	A	1.173	0.32
4159	U	0.773	0.2
4160	A	1.424	0.1
4161	C	0.814	0.26
4162	A	1.567	0.31
4163	U	0.848	0.83
4164	G	0.597	0.61
4165	G	0.353	0.26
4166	G	0.475	0.06
4167	C	0.102	0.22
4168	A	0.387	0.23

4169	G	0.359	0.09
4170	G	0.393	0.15
4171	C	0.183	0.27
4172	A	0.563	0.23
4173	A	0.482	0.31
4174	A	0.515	0.41
4175	U	0.366	0.7
4176	U	0.787	0.42
4177	C	0.434	0.37
4178	A	1.268	0.31
4179	G	0.977	0.3
4180	A	1.133	0.26
4181	U	0.556	0.62
4182	C	0.373	0.76
4183	U	0	0.66
4184	A	0.719	0.28
4185	G	0.549	0.1
4186	G	0.949	0.08
4187	G	1.207	0.02
4188	A	1.085	0.17
4189	C	0.678	0.2
4190	U	0.258	0.31
4191	U	0.4	0.76
4192	G	0.292	0.78
4193	G	0.203	0.7
4194	C	0.109	0.71
4195	A	0.766	0.5
4196	A	0.943	0.49
4197	A	0.76	0.83
4198	U	0.488	0.87
4199	G	0.475	0.76
4200	G	0.719	0.26
4201	A	0.821	0.63
4202	U	0.468	0.8
4203	U	0.224	0.85
4204	G	0.258	0.83
4205	U	0.034	0.67
4206	A	0.461	0.3
4207	C	0.868	0.2
4208	C	0.841	0.2
4209	C	0.841	0.2
4210	A	0.468	0.31
4211	U	0.231	0.47

4212	C	0.285	0.72
4213	U	0.163	0.51
4214	A	0.359	0.15
4215	G	0.821	0.06
4216	A	0.665	0.05
4217	G	0.549	0.16
4218	G	0.536	0.19
4219	G	1.207	0.25
4220	A	1.336	0.37
4221	A	1.316	0.31
4222	A	1.044	0.21
4223	A	1.146	0.14
4224	A	0.855	0.2
4225	U	1.194	0.42
4226	A	0.699	0.27
4227	A	0.732	0.16
4228	U	0.536	0.23
4229	C	0.271	0.14
4230	A	0.916	0.2
4231	U	0.766	0.35
4232	A	0.624	0.32
4233	G	0.556	0.54
4234	U	0.298	0.51
4235	U	0.19	0.18
4236	G	0.258	0.66
4237	C	0.454	0.78
4238	A	0.278	0.79
4239	G	0.502	0.74
4240	U	1.024	0.67
4241	A	0.8	0.26
4242	C	0.346	0.68
4243	A	1.099	0.73
4244	U	0.088	0.87
4245	G	0.109	0.25
4246	U	0.19	0.55
4247	A	0.285	0.59
4248	G	0.115	0.79
4249	C	0.081	0.81
4250	U	0.034	0.81
4251	A	0.007	0.77
4252	G	4.469	0.38
4253	U	0.278	0.43
4254	G	1.058	0.38

4255	G	0.461	0.29
4256	A	0.244	0.32
4257	U	0.597	0.51
4258	U	0.448	0.53
4259	C	0.855	0.49
4260	A	0.875	0.19
4261	U	1.39	0.26
4262	A	1.16	0.14
4263	G	0.556	0.08
4264	A	0.427	0.07
4265	A	0.366	0.06
4266	G	0.176	0.11
4267	C	0.258	0.22
4268	A	0.109	0.21
4269	G	0.339	0.18
4270	A	0.475	0.16
4271	G	0	0.12
4272	G	0	0.13
4273	U	0.936	0.14
4274	A	0.909	0.08
4275	A	1.316	0.14
4276	U	0.258	0.16
4277	U	0	0.06
4278	C	0	0.07
4279	C	1.546	0.06
4280	A	0	0.04
4281	C	0.665	0.05
4282	A	1.072	0.1
4283	A	0.203	0.13
4284	G	1.451	0.24
4285	A	0.76	0.49
4286	G	1.112	0.78
4287	A	0.407	0.49
4288	C	0.814	0.33
4289	A	1.302	0.87
4290	G	0	0.97
4291	G	0.827	0.98
4292	A	1.519	0.97
4293	A	0.637	0.97
4294	G	0.705	0.91
4295	A	0.99	0.76
4296	C	0.719	0.04
4297	A	1.072	0.11

4298	G	1.153	0.13
4299	A	1.424	0.06
4300	C	1.044	0.03
4301	A	0	0.03
4302	G	0	0.03
4303	C	0.407	0.03
4304	A	0.61	0.1
4305	C	0.624	0.16
4306	U	0.258	0.84
4307	A	0.203	0.9
4308	U	0.109	0.97
4309	U	0.014	0.99
4310	U	0	0.99
4311	C	0.081	0.97
4312	U	0.122	0.87
4313	G	0	0.45
4314	U	0.217	0.81
4315	U	1.112	0.79
4316	A	1.017	0.39
4317	A	0.895	0.28
4318	A	0.787	0.28
4319	A	1.207	0.29
4320	U	0.977	0.36
4321	U	1.126	0.44
4322	G	0.081	0.32
4323	G	0.136	0.29
4324	C	0.081	0.26
4325	A	0.488	0.26
4326	G	0.312	0.33
4327	G	0.285	0.28
4328	C	0.529	0.22
4329	A	0.963	0.46
4330	G	0.773	0.49
4331	A	0.366	0.37
4332	U	0.543	0.73
4333	G	0.57	0.86
4334	G	0.231	0.64
4335	C	0.027	0.37
4336	C	0.543	0.28
4337	U	0.868	0.27
4338	A	0.76	0.33
4339	U	1.058	0.27
4340	U	2.048	0.13

4341	A	1.139	0.11
4342	C	1.194	0.2
4343	A	0.353	0.17
4344	C	0.814	0.36
4345	A	0.475	0.34
4346	U	0.217	0.44
4347	C	0.882	0.77
4348	U	1.831	0.62
4349	A	0.502	0.33
4350	C	2.021	0.25
4351	A	0.827	0.19
4352	C	0.326	0.2
4353	A	0.271	0.21
4354	C	0.366	0.27
4355	A	0.041	0.43
4356	G	0.529	0.45
4357	A	1.004	0.22
4358	U	0.38	0.21
4359	A	0	0.22
4360	A	0.326	0.25
4361	U	0.122	0.34
4362	G	0.529	0.43
4363	G	0	0.36
4364	U	0.231	0.42
4365	G	0	0.39
4366	C	0.42	0.49
4367	U	0.827	0.55
4368	A	1.112	0.5
4369	A	0.787	0.34
4370	C	0.285	0.51
4371	U	0.339	0.53
4372	U	0.502	0.43
4373	U	1.044	0.42
4374	G	0	0.39
4375	C	0.556	0.38
4376	U	0.583	0.39
4377	U	0.081	0.44
4378	C	0.719	0.44
4379	G	0.868	0.44
4380	C	0	0.24
4381	A	0.515	0.03
4382	A	0.136	0.04
4383	G	0.515	0.15

4384	A	0.217	0.14
4385	A	0.38	0.07
4386	G	0.163	0.33
4387	U	0.502	0.34
4388	A	0.448	0.3
4389	A	0.475	0.28
4390	A	0.719	0.13
4391	G	0.475	0.23
4392	A	0.624	0.17
4393	U	0	0.47
4394	G	0.271	0.55
4395	G	0.068	0.5
4396	U	0.651	0.5
4397	U	0.597	0.4
4398	G	0.515	0.05
4399	C	0.19	0.09
4400	A	0.475	0.08
4401	U	0.637	0.31
4402	G	0.176	0.35
4403	G	0.19	0.6
4404	U	0.176	0.66
4405	G	0.271	0.64
4406	G	0.665	0.48
4407	G	1.397	0.55
4408	C	0.041	0.87
4409	A	1.207	0.79
4410	G	0.909	0.51
4411	G	0.922	0.46
4412	G	0.244	0.04
4413	A	0.705	0.06
4414	U	0.57	0.48
4415	A	1.085	0.48
4416	G	0.977	0.22
4417	A	0.855	0.25
4418	G	1.234	0.25
4419	C	0.353	0.09
4420	A	0.651	0.07
4421	C	0.475	0.04
4422	A	0.529	0.07
4423	C	0.543	0.15
4424	C	0.882	0.23
4425	U	0.977	0.44
4426	U	0.326	0.32

4427	U	0.787	0.21
4428	G	0.502	0.24
4429	G	0.203	0.23
4430	G	0	0.07
4431	G	0.068	0.15
4432	U	0.285	0.2
4433	A	0.42	0.12
4434	C	0.068	0.31
4435	C	0.149	0.42
4436	A	0.678	0.24
4437	U	0.407	0.32
4438	A	0.543	0.25
4439	C	1.072	0.09
4440	A	1.166	0.03
4441	A	0.543	0.29
4442	U	0.231	0.43
4443	C	0.149	0.45
4444	C	0.176	0.42
4445	A	0.285	0.39
4446	C	0.027	0.37
4447	A	0.543	0.08
4448	G	0.692	0.05
4449	A	0.583	0.02
4450	G	0.38	0.14
4451	U	0.515	0.17
4452	C	0.448	0.24
4453	A	0.678	0.2
4454	G	0.448	0.04
4455	G	0.543	0.2
4456	G	0.61	0.21
4457	A	0.814	0.07
4458	G	0.448	0.24
4459	U	0.081	0.46
4460	A	0.665	0.45
4461	G	0.285	0.51
4462	U	0.393	0.32
4463	G	0.515	0.15
4464	G	0.393	0.15
4465	A	0.637	0.1
4466	A	0.8	0.09
4467	G	0.393	0.26
4468	C	0.068	0.44
4469	A	0.231	0.43

4470	A	0.298	0.4
4471	U	0.149	0.42
4472	G	0.217	0.31
4473	A	0.163	0.03
4474	A	0.095	0.05
4475	U	0.746	0.14
4476	C	0.231	0.13
4477	A	0.909	0.1
4478	C	0.699	0.12
4479	C	0.719	0.21
4480	A	0.8	0.14
4481	C	0.414	0.08
4482	C	0.705	0.15
4483	U	0.488	0.15
4484	G	0.387	0.07
4485	A	0.482	0
4486	A	0.868	0
4487	A	0.766	0
4488	A	0.468	0
4489	A	0.339	0
4490	U	1.038	0
4491	C	0.38	0.01
4492	A	1.065	0.01
4493	A	1.268	0.02
4494	A	2.13	0.39
4495	U	0.461	0.41
4496	A	0.861	0.13
4497	G	0.285	0.12
4498	A	0.4	0.11
4499	U	1.241	0.12
4500	A	1.241	0.3
4501	G	0.888	0.35
4502	A	1.051	0.17
4503	A	0.414	0.54
4504	U	0.197	0.54
4505	C	0.203	0.21
4506	A	0.427	0.22
4507	G	0.264	0.22
4508	G	0.19	0.28
4509	G	0.482	0.28
4510	A	0.61	0.29
4511	A	0.495	0.3
4512	C	0.617	0.35

4513	A	0.644	0.33
4514	A	0.536	0.21
4515	G	0.434	0.36
4516	C	0.414	0.64
4517	A	0.583	0.66
4518	A	0.549	0.39
4519	A	0.563	0.21
4520	U	0.332	0.75
4521	U	0.366	0.69
4522	C	0.353	0.37
4523	A	0.305	0.49
4524	G	0.536	0.51
4525	U	0.509	0.51
4526	A	1.031	0.43
4527	G	0.576	0.31
4528	A	0.814	0.05
4529	A	1.146	0.03
4530	A	0.353	0.18
4531	C	0.468	0.21
4532	C	0.42	0.07
4533	A	0.4	0.09
4534	U	0.42	0.71
4535	A	1.295	0.75
4536	G	1.031	0.35
4537	U	0.834	0.43
4538	A	1.173	0.29
4539	U	0.963	0.66
4540	U	0.753	0.65
4541	A	1.16	0.3
4542	A	0.705	0.34
4543	U	0.244	0.71
4544	G	0.095	0.66
4545	G	0.244	0.58
4546	C	0.176	0.74
4547	A	0.434	0.73
4548	G	0.203	0.36
4549	U	0.122	0.24
4550	U	0.19	0.22
4551	C	0.081	0.41
4552	A	0.393	0.4
4553	U	0.461	0.45
4554	U	0.095	0.91
4555	G	0.339	0.93

4556	C	0.38	0.88
4557	A	1.397	0.73
4558	U	0.705	0.95
4559	G	1.099	0.96
4560	A	0.882	0.97
4561	A	0.977	0.97
4562	U	0.502	0.99
4563	U	0.502	1
4564	U	0.841	0.99
4565	U	0.977	0.95
4566	A	1.261	0.06
4567	A	1.424	0.04
4568	A	1.668	0.05
4569	A	1.343	0.04
4570	G	1.126	0.03
4571	A	1.424	0.05
4572	A	1.126	0.05
4573	G	0.651	0.09
4574	G	0.366	0.13
4575	G	0	0.87
4576	G	0.014	0.92
4577	A	0.163	0.96
4578	G	0	0.98
4579	G	0.38	0.98
4580	A	0.556	0.97
4581	A	0.732	0.97
4582	U	0.692	0.89
4583	A	0.746	0.2
4584	G	0.529	0.38
4585	G	0.38	0.6
4586	G	0.665	0.61
4587	G	0.543	0.38
4588	A	0.963	0.15
4589	U	0.38	0.37
4590	A	0.746	0.29
4591	U	0.353	0.41
4592	G	0.814	0.28
4593	A	0.556	0.04
4594	C	0.231	0.35
4595	U	0.258	0.5
4596	C	0.081	0.55
4597	C	0.081	0.43
4598	A	0.61	0.13

4599	G	0.868	0.08
4600	C	0.054	0.26
4601	A	1.031	0.31
4602	G	0.841	0.16
4603	A	0.922	0.1
4604	A	0.814	0.03
4605	A	1.058	0.05
4606	G	0.665	0.23
4607	A	0.705	0.32
4608	U	0.231	0.4
4609	U	0.855	0.48
4610	A	1.017	0.26
4611	A	1.017	0.24
4612	U	0.475	0.56
4613	U	1.112	0.4
4614	A	1.411	0.08
4615	A	1.099	0.06
4616	C	0.678	0.07
4617	A	1.465	0.22
4618	U	0.705	0.78
4619	G	0.475	0.75
4620	A	0.448	0.37
4621	U	0.163	0.41
4622	C	0.054	0.23
4623	A	0.095	0.12
4624	C	0.081	0.18
4625	U	0.244	0.15
4626	A	0.339	0.06
4627	C	0.203	0.3
4628	A	1.356	0.3
4629	G	1.248	0.13
4630	A	1.112	0.09
4631	A	0.76	0.03
4632	C	0.19	0.19
4633	A	0.895	0.19
4634	A	1.072	0.04
4635	G	1.465	0.14
4636	A	0.977	0.12
4637	G	0.841	0.14
4638	A	0.963	0.4
4639	U	0.705	0.57
4640	A	0.705	0.3
4641	C	0.068	0.17

4642	A	0.692	0.14
4643	A	0.76	0.77
4644	U	0.61	0.97
4645	U	0.624	1
4646	U	0.461	0.99
4647	C	0.19	0.99
4648	A	0.556	0.98
4649	A	0.42	0.98
4650	C	0.353	0.89
4651	A	0.529	0.02
4652	A	0.8	0.02
4653	U	1.533	0.01
4654	C	0.041	0.01
4655	A	1.329	0.01
4656	A	0.773	0.07
4657	A	1.153	0.9
4658	A	0.977	0.94
4659	A	0.76	0.95
4660	A	0.434	0.96
4661	C	0.081	0.99
4662	U	0	0.99
4663	C	0.109	0.96
4664	A	0.8	0.92
4665	A	0.773	0.86
4666	A	0.787	0.96
4667	A	0.597	1
4668	U	0.326	0.99
4669	U	0.936	0.85
4670	U	1.628	0
4671	A	1.438	0
4672	A	1.004	0
4673	A	1.112	0
4674	A	1.072	0.85
4675	A	0.678	0.99
4676	U	0.326	1
4677	U	0.57	0.99
4678	U	0.57	0.96
4679	U	0.8	0.9
4680	C	0.203	0.92
4681	G	0.244	0.92
4682	G	0.109	0.95
4683	G	0.041	0.95
4684	U	0.014	0.97

4685	C	0.027	0.97
4686	U	0.637	0.55
4687	A	0.719	0.11
4688	U	0.678	0.78
4689	U	0.583	0.84
4690	A	0.38	0.39
4691	C	0	0.58
4692	A	0.258	0.39
4693	G	0.339	0.15
4694	A	0.407	0.18
4695	G	0.583	0.18
4696	A	0.678	0.21
4697	A	0.76	0.28
4698	G	0.298	0.18
4699	G	0.095	0.11
4700	C	0	0.34
4701	A	0.543	0.47
4702	G	0.746	0.31
4703	A	0.909	0.36
4704	G	0.271	0.34
4705	A	1.397	0.05
4706	U	0.163	0.15
4707	C	0.109	0.19
4708	A	0.393	0.1
4709	A	0.543	0.02
4710	C	0.203	0.75
4711	U	0.217	0.76
4712	G	0.244	0.21
4713	U	0.109	0.73
4714	G	0.597	0.75
4715	G	0.705	0.47
4716	A	0.665	0.62
4717	A	0.448	0.63
4718	G	0.922	0.4
4719	G	0.217	0.45
4720	G	0.244	0.79
4721	A	0.176	0.86
4722	C	0.109	0.95
4723	C	0	0.95
4724	C	0	0.89
4725	G	0.163	0.89
4726	G	0.543	0.82
4727	U	0.149	0.29

4728	G	0.407	0.31
4729	A	0	0.45
4730	G	0.203	0.36
4731	C	0.203	0.49
4732	U	0.109	0.53
4733	A	0.326	0.4
4734	U	0.244	0.69
4735	U	0.298	0.78
4736	G	0.624	0.63
4737	U	0.149	0.47
4738	G	0.434	0.42
4739	G	0.61	0.1
4740	A	0.637	0.04
4741	A	0.746	0.01
4742	A	0.827	0.04
4743	G	0.407	0.04
4744	G	0.258	0.01
4745	G	0.231	0.01
4746	G	0.122	0.06
4747	A	0.922	0.11
4748	A	0.678	0.72
4749	G	0.42	0.73
4750	G	0.448	0.83
4751	A	0.434	0.75
4752	G	0.271	0.01
4753	C	0	0.01
4754	A	0.38	0.01
4755	G	0.285	0.02
4756	U	0.054	0.83
4757	C	0.312	0.9
4758	A	0.732	0.74
4759	U	0.122	0.71
4760	C	0.19	0.03
4761	U	0.502	0.01
4762	U	0.787	0.05
4763	A	0.773	0.04
4764	A	1.044	0.01
4765	A	0.949	0.01
4766	G	0.461	0.86
4767	G	0.19	0.94
4768	U	0.19	0.94
4769	A	0.678	0.8
4770	G	0.122	0.02

4771	G	0.42	0.02
4772	G	0.312	0.01
4773	A	0.42	0.06
4774	C	0.122	0.06
4775	A	0.583	0.01
4776	G	0.8	0.01
4777	A	0.529	0.02
4778	C	0.407	0.11
4779	A	1.044	0.39
4780	U	0.543	0.38
4781	U	1.316	0.02
4782	A	1.438	0
4783	A	0.936	0
4784	G	0.61	0.01
4785	G	0.543	0.3
4786	U	0.895	0.34
4787	A	0.773	0.14
4788	G	0.475	0.15
4789	U	0.448	0.91
4790	A	0.109	0.93
4791	C	0.203	0.94
4792	C	0.122	0.88
4793	C	0	0.01
4794	A	0.556	0.02
4795	G	0.658	0.01
4796	A	1.343	0
4797	A	1.953	0
4798	G	0.258	0
4799	A	1.126	0
4800	A	1.255	0
4801	A	1.051	0
4802	G	0.305	8.37E-05
4803	G	0.142	0
4804	C	0.922	0
4805	U	0.237	0
4806	A	1.445	0
4807	A	1.072	6.30E-05
4808	A	0.712	4.83E-05
4809	A	0.624	0
4810	U	0.38	0
4811	U	1.974	0
4812	A	0.861	0
4813	U	1.255	0

4814	C	0.529	0
4815	A	0.719	0
4816	A	0.773	0
4817	A	0.814	0
4818	G	0.515	0.01
4819	A	0.57	0.34
4820	U	0.312	0.8
4821	U	2.998	0.95
4822	A	1.451	0.92
4823	U	0.19	0.89
4824	G	0.095	0.01
4825	G	0.42	0.01
4826	A	0.393	0.09
4827	G	0.027	0.09
4828	G	0.597	0.01
4829	A	0.543	0.02
4830	G	0.434	0.05
4831	G	0.244	0.06
4832	A	0.692	0.51
4833	A	0.814	0.5
4834	A	0.692	0.01
4835	A	0.827	0.01
4836	G	0.665	0.01
4837	A	0.529	0
4838	G	0.014	0
4839	G	0.258	0.67
4840	U	0.217	0.67
4841	G	0.149	0.01
4842	G	0.217	0.01
4843	A	0.651	0.89
4844	U	1.56	0.93
4845	A	1.085	0.67
4846	G	0.176	0.45
4847	C	0.556	0.01
4848	A	0.448	0.28
4849	G	0.041	0.37
4850	U	0.109	0.45
4851	U	0.163	0.09
4852	C	0.068	0.08
4853	C	0.027	0.16
4854	C	0.109	0.12
4855	A	0.339	0.14
4856	C	0.515	0.25

4857	A	0.285	0.25
4858	U	0.149	0.89
4859	G	0.095	1
4860	G	0.149	0.88
4861	A	0.339	0.05
4862	G	0.014	0
4863	G	0.068	0
4864	A	0.312	0
4865	U	0.624	0.02
4866	A	0.42	0.06
4867	C	0.109	0.89
4868	C	0.298	0.98
4869	G	0.42	0.87
4870	G	0.827	0.29
4871	A	0.732	0.28
4872	G	0.054	0.09
4873	A	0.149	0.13
4874	G	0.054	0.06
4875	G	0.041	0.06
4876	C	0.027	0.1
4877	U	0.38	0.26
4878	A	0.366	0.18
4879	G	0.475	0.14
4880	A	0.8	0.03
4881	G	0.827	0.01
4882	A	0.692	0.07
4883	G	0.217	0.08
4884	G	0.244	0.02
4885	U	0.448	0.27
4886	G	0.298	0.87
4887	G	0	0.76
4888	C	0.136	0.05
4889	A	0.692	0.11
4890	U	0.095	0.09
4891	A	0.231	0.04
4892	G	0.014	0.02
4893	C	0	0.7
4894	C	0.027	0.79
4895	U	0.054	0.2
4896	C	0	0.09
4897	A	1.438	0.09
4898	U	0.841	0.13
4899	A	0.855	0.16

4900	A	0.543	0.15
4901	A	0.488	0.02
4902	A	0.488	0.01
4903	U	0.895	0.02
4904	A	0.353	0.01
4905	U	0.176	0
4906	C	0.176	0.02
4907	U	0.298	0.03
4908	G	0.624	0.01
4909	A	0.543	0
4910	A	0.488	0.01
4911	A	0.678	0.01
4912	U	1.017	0.22
4913	A	1.899	0.22
4914	U	0.597	0.02
4915	A	0.936	0.03
4916	A	0.705	0.03
4917	A	0.787	0.39
4918	A	0.556	0.44
4919	C	0.543	0.14
4920	U	1.777	0.12
4921	A	0.963	0.08
4922	A	1.316	0.03
4923	A	0.949	0.03
4924	G	0.597	0.08
4925	A	0.57	0.07
4926	U	0.068	0.05
4927	C	0.122	0.45
4928	U	0.461	0.64
4929	A	0.746	0.22
4930	C	0.665	0.01
4931	A	1.031	0.01
4932	A	0.651	0.01
4933	A	1.112	0.02
4934	A	0.895	0.02
4935	G	0.597	0.02
4936	G	0.027	0.03
4937	U	0.163	0.03
4938	U	0.271	0.02
4939	U	0.285	0.02
4940	G	0.163	0.08
4941	C	0.054	0.56
4942	U	0.407	0.68

4943	A	0.76	0.66
4944	U	0.393	0.84
4945	G	0.61	0.82
4946	U	0.231	0.27
4947	G	0.203	0.11
4948	C	0.109	0.14
4949	C	0.027	0.43
4950	C	0.081	0.44
4951	C	0.298	0.73
4952	A	0.515	0.74
4953	U	0.38	0.45
4954	U	0.475	0.48
4955	U	1.099	0.31
4956	U	1.736	0.24
4957	A	1.343	0.23
4958	A	1.017	0.14
4959	G	0.366	0.19
4960	G	0.298	0.19
4961	U	0.339	0.25
4962	C	0.339	0.3
4963	G	0.678	0.31
4964	G	0.76	0.44
4965	A	0.353	0.46
4966	U	0.203	0.67
4967	G	0.041	0.8
4968	G	0.014	0.81
4969	G	0	0.73
4970	C	0.136	0.51
4971	A	1.546	0.55
4972	U	0.868	0.68
4973	G	0.366	0.73
4974	G	0.298	0.8
4975	U	0.407	0.78
4976	G	0	0.81
4977	G	0	0.81
4978	A	0	0.8
4979	C	0.014	0.85
4980	C	0	0.76
4981	U	0.027	0.94
4982	G	0.095	0.96
4983	C	0.081	0.97
4984	A	0.095	0.92
4985	G	0.027	0.91

4986	C	0.054	0.92
4987	A	0.339	0.91
4988	G	0.61	0.9
4989	A	0.448	0.66
4990	G	0.502	0.72
4991	U	1.275	0.74
4992	A	0.922	0.88
4993	A	1.004	0.89
4994	U	0.353	0.88
4995	C	0	0.82
4996	U	0.054	0.58
4997	U	0.054	0.65
4998	C	0.027	0.44
4999	C	0.027	0.14
5000	C	0.176	0.12
5001	A	0.393	0.24
5002	C	0.326	0.56
5003	U	1.166	0.56
5004	A	0.963	0.11
5005	C	0.719	0.08
5006	A	0.705	0.06
5007	G	0.339	0.15
5008	G	0.38	0.4
5009	A	0.529	0.38
5010	A	0.502	0.59
5011	G	0.353	0.55
5012	G	0.529	0.5
5013	A	0.543	0.35
5014	A	0.515	0.07
5015	G	0.163	0.34
5016	C	0.095	0.41
5017	C	0.448	0.51
5018	A	0.42	0.52
5019	U	0.298	0.77
5020	U	0.543	0.87
5021	U	1.085	0.76
5022	A	1.533	0.55
5023	G	1.024	0.66
5024	A	0.861	0.61
5025	A	0.482	0.81
5026	G	0.339	0.58
5027	U	0.651	0.48
5028	A	0.699	0.11

5029	C	0.271	0.2
5030	A	0.882	0.2
5031	A	0.556	0.64
5032	G	0.244	0.65
5033	G	0.203	0.64
5034	G	0.298	0.23
5035	U	0.298	0.41
5036	A	0.298	0.45
5037	U	0.17	0.6
5038	U	0.393	0.67
5039	G	0.61	0.48
5040	G	0.129	0.38
5041	C	0.102	0.11
5042	A	0.244	0.08
5043	U	0.231	0.06
5044	U	0.692	0.69
5045	U	0.529	0.69
5046	G	0.495	0.04
5047	A	0.583	0.02
5048	C	0.231	0.02
5049	A	0.312	0.01
5050	C	0.109	0.37
5051	C	0.264	0.38
5052	A	0.949	0.26
5053	G	1.017	0.1
5054	A	1.241	0.04
5055	A	1.275	0.06
5056	A	0.875	0.53
5057	A	1.065	0.51
5058	A	0.963	0.09
5059	G	1.119	0.2
5060	G	0.19	0.34
5061	G	0.312	0.57
5062	U	0.075	0.68
5063	G	0.373	0.35
5064	G	0.231	0.65
5065	C	0.007	0.71
5066	U	0.42	0.63
5067	C	0.278	0.24
5068	A	0.407	0.24
5069	G	0.482	0.09
5070	U	0.719	0.14
5071	A	0.38	0.22

5072	C	0.109	0.15
5073	U	0.461	0.37
5074	U	0.827	0.44
5075	A	1.044	0.45
5076	U	0.366	0.74
5077	G	0.475	0.77
5078	C	0.468	0.69
5079	A	0.4	0.5
5080	G	0.509	0.6
5081	U	0.298	0.75
5082	G	0.671	0.41
5083	A	0.76	0.15
5084	G	1.166	0.14
5085	G	0.353	0.02
5086	A	0.556	0.03
5087	U	0.787	0.26
5088	A	1.411	0.25
5089	A	0.543	0.36
5090	C	0.176	0.5
5091	C	0	0.55
5092	U	0.095	0.34
5093	G	0.42	0.31
5094	G	0.366	0.76
5095	U	0.366	0.83
5096	A	0.054	0.81
5097	C	0.19	0.59
5098	U	0.678	0.22
5099	C	0.678	0.24
5100	A	1.302	0.32
5101	A	0.827	0.5
5102	A	1.221	0.5
5103	G	0.705	0.08
5104	A	0.814	0.28
5105	A	0.57	0.67
5106	C	0.163	0.61
5107	U	0.298	0.65
5108	U	0.543	0.94
5109	U	0.448	0.92
5110	U	0.597	0.85
5111	G	0.583	0.34
5112	G	0.461	0.14
5113	A	0.732	0.17
5114	C	0.231	0.16

5115	A	0.841	0.47
5116	G	0.57	0.59
5117	A	1.085	0.6
5118	U	0.461	0.78
5119	G	0.597	0.89
5120	U	0.515	0.68
5121	A	1.316	0.44
5122	A	0.936	0.11
5123	C	0.461	0.09
5124	A	1.112	0.05
5125	C	0.353	0.12
5126	C	0.461	0.51
5127	A	1.004	0.57
5128	A	1.424	0.21
5129	A	1.18	0.11
5130	C	0.19	0.1
5131	U	0.217	0.15
5132	A	0.353	0.13
5133	U	0.326	0.45
5134	G	0.014	0.54
5135	C	0.339	0.32
5136	A	0.285	0.45
5137	G	0	0.35
5138	A	1.044	0.2
5139	C	0.543	0.14
5140	A	1.628	0.66
5141	U	0.882	0.65
5142	U	0.719	0.13
5143	U	1.18	0.23
5144	U	1.18	0.34
5145	A	1.533	0.28
5146	C	0.027	0.79
5147	U	0.38	0.79
5148	G	0.285	0.25
5149	C	0.312	0.25
5150	A	0.122	0.26
5151	U	0	0.2
5152	A	0.054	0.26
5153	G	0.068	0.24
5154	C	0.434	0.05
5155	A	0.868	0.98
5156	C	0.298	0.98
5157	U	0.217	0.91

5158	U	0.556	0.52
5159	A	1.316	0.06
5160	U	0.732	0.62
5161	U	0.407	0.91
5162	U	0.19	0.94
5163	C	0.109	0.91
5164	C	0.231	0.87
5165	C	0.109	0.92
5166	U	0.407	0.96
5167	U	0.746	0.97
5168	G	0.814	0.95
5169	C	0.543	0.97
5170	U	1.017	0.97
5171	U	1.356	0.92
5172	U	1.424	0.57
5173	A	1.058	0.53
5174	C	0.353	0.27
5175	A	0.502	0.11
5176	G	0.434	0.02
5177	C	0.42	0.02
5178	G	0.176	0.01
5179	G	0.312	0
5180	G	0.407	0.16
5181	A	0.637	0.59
5182	G	0.705	0.61
5183	A	0.461	0.89
5184	A	0.732	0.94
5185	G	0.488	0.96
5186	U	0.339	0.94
5187	G	0.855	0.94
5188	A	1.112	0.91
5189	G	1.072	0.71
5190	A	1.682	0.34
5191	A	2.007	0.16
5192	G	0.692	0.63
5193	G	0.014	0.63
5194	G	0.014	0.08
5195	C	0.217	0.2
5196	C	0.57	0.21
5197	A	0.787	0.04
5198	U	0.719	0.66
5199	C	1.031	0.64
5200	A	1.926	0.07

5201	G	0.773	0.07
5202	G	0.448	0.07
5203	G	0.163	0.27
5204	G	0.624	0.65
5205	A	0.76	0.72
5206	G	0.949	0.77
5207	A	1.139	0.85
5208	A	1.112	0.73
5209	C	0.773	0.89
5210	A	1.79	0.91
5211	A	1.031	0.97
5212	C	0.407	0.98
5213	U	0.488	0.68
5214	G	0.732	0.78
5215	C	0.217	0.84
5216	U	0.407	0.89
5217	G	1.289	0.76
5218	U	0.583	0.75
5219	C	0.407	0.67
5220	U	1.194	0.91
5221	U	1.234	0.94
5222	G	0.529	0.94
5223	C	0.298	0.92
5224	U	0.203	0.94
5225	G	0.122	0.95
5226	C	0.42	0.95
5227	A	0.339	0.92
5228	G	0.244	0.77
5229	G	0.203	0.43
5230	U	0.353	0.58
5231	U	0.271	0.52
5232	C	0.285	0.4
5233	C	0.163	0.52
5234	C	0.434	0.32
5235	G	2.414	0.18
5236	A	2.591	0.14
5237	G	1.709	0.12
5238	A	1.438	0.12
5239	G	0.109	0.28
5240	C	0.068	0.31
5241	U	0.298	0.3
5242	C	0	0.37
5243	A	0.895	0.37

5244	U	0.99	0.28
5245	A	1.763	0.24
5246	A	0.8	0.21
5247	G	0.285	0.29
5248	U	0.393	0.28
5249	A	0.326	0.17
5250	C	0.393	0.24
5251	C	0.19	0.27
5252	A	0.271	0.64
5253	G	0.57	0.78
5254	G	0.488	0.64
5255	U	0	0.51
5256	A	1.153	0.53
5257	C	0.353	0.48
5258	C	0.651	0.5
5259	A	0.868	0.51
5260	A	0.99	0.46
5261	G	0.434	0.35
5262	C	0.448	0.75
5263	C	0.136	0.79
5264	U	0.475	0.64
5265	A	1.072	0.03
5266	C	0.882	0.14
5267	A	0.448	0.14
5268	G	0.502	0.1
5269	U	0.543	0.29
5270	A	0.61	0.27
5271	C	0.326	0.31
5272	U	0.529	0.39
5273	U	0.949	0.49
5274	A	1.248	0.49
5275	G	0.692	0.4
5276	C	0.271	0.58
5277	A	0.285	0.8
5278	C	0.014	0.81
5279	U	0.461	0.91
5280	G	1.072	0.76
5281	A	1.18	0.92
5282	A	1.261	0.9
5283	A	0.949	0.14
5284	G	0.719	0.1
5285	U	0.353	0.1
5286	A	0.529	0.04

5287	G	0.773	0.3
5288	U	0.855	0.34
5289	A	1.261	0.3
5290	A	1.207	0.28
5291	G	1.126	0.01
5292	C	0.529	0
5293	G	0.949	0.01
5294	A	0.746	0.01
5295	U	0.326	0.04
5296	G	0.38	0.06
5297	U	0.366	0.05
5298	C	0.461	0.02
5299	A	0.705	0.01
5300	G	0.665	0.01
5301	A	0.475	0.8
5302	U	0.258	0.65
5303	C	0.041	0.57
5304	C	0.176	0.1
5305	C	0.217	0.06
5306	A	0.149	0.07
5307	G	0.665	0.06
5308	G	0.122	0.03
5309	G	0.339	0.04
5310	A	0.502	0.03
5311	G	0.963	0.02
5312	A	1.438	0.01
5313	G	0.895	0.02
5314	A	0.637	0.03
5315	A	0.705	0.6
5316	U	0.109	0.68
5317	C	0.014	0.17
5318	C	0.122	0.41
5319	C	0	0.4
5320	A	0.176	0.01
5321	C	0.217	0.09
5322	C	0.163	0.09
5323	U	0.271	0.09
5324	G	0.488	0.44
5325	G	0.692	0.47
5326	A	1.126	0.09
5327	A	0.326	0.12
5328	A	0.637	0.46
5329	C	0.583	0.46

5330	A	0.556	0.06
5331	G	0.339	0.1
5332	U	0.217	0.1
5333	G	0.326	0.51
5334	G	0.529	0.5
5335	A	0.814	0.22
5336	G	0.556	0.31
5337	A	0.705	0.13
5338	A	0.637	0.16
5339	G	0.827	0.19
5340	A	0.719	0.13
5341	G	0.651	0.14
5342	A	0.651	0.05
5343	C	0.624	0.33
5344	A	0.936	0.34
5345	A	0.909	0.39
5346	U	1.004	0.45
5347	A	1.953	0.13
5348	G	0.821	0.62
5349	G	0.773	0.62
5350	A	0.793	0.6
5351	G	0.76	0.21
5352	A	0.902	0.04
5353	G	0.814	0.01
5354	G	0.651	0.02
5355	C	0.136	0.05
5356	C	0.142	0.24
5357	U	0.482	0.87
5358	U	3.269	0.95
5359	C	0.434	0.89
5360	G	0.319	0.07
5361	A	0.482	0.16
5362	A	0.902	0.89
5363	U	0.848	0.96
5364	G	0.122	0.52
5365	G	0.339	0.62
5366	C	0.075	0.6
5367	U	0.726	0.7
5368	A	0.78	0.21
5369	A	0.556	0.06
5370	A	0.515	0.03
5371	C	1.594	0.02
5372	A	0.943	0.27

5373	G	1.139	0.33
5374	A	0.827	0.08
5375	A	0.475	0.03
5376	C	0.326	0.24
5377	A	0.312	0.31
5378	G	0.041	0.21
5379	U	1.492	0.31
5380	A	0.543	0.29
5381	G	0.353	0.16
5382	A	0.787	0.04
5383	G	0.637	0.03
5384	G	0.651	0.06
5385	A	0.515	0.04
5386	G	0.163	0.14
5387	A	1.655	0.73
5388	U	1.75	0.76
5389	A	1.072	0.09
5390	A	1.004	0.03
5391	A	0.963	0.08
5392	C	1.289	0.09
5393	A	0.814	0.14
5394	G	0.366	0.17
5395	A	0.624	0.12
5396	G	0.42	0.1
5397	A	1.153	0.05
5398	G	0.258	0.18
5399	G	0.231	0.22
5400	C	0.109	0.2
5401	G	0.109	0.02
5402	G	0.258	0.09
5403	U	0.515	0.09
5404	A	0.977	0.01
5405	A	0.922	0.03
5406	A	0.583	0.23
5407	C	0.054	0.23
5408	C	0.529	0
5409	A	0.855	0
5410	C	0.041	0.01
5411	C	0.19	0.48
5412	U	0.529	0.48
5413	A	0.597	0
5414	C	0.136	0
5415	C	0.705	0.01

5416	A	0.488	0.01
5417	A	0.583	0.47
5418	G	0.298	0.47
5419	G	0.068	0.02
5420	G	0.556	0.01
5421	A	0.637	0
5422	G	0.326	0.01
5423	C	0.109	0.01
5424	U	1.017	0.01
5425	A	0.936	0
5426	A	0.42	0.07
5427	U	0.217	0.09
5428	U	0.353	0.93
5429	U	0.339	0.94
5430	U	0.312	0.97
5431	C	0.081	1
5432	C	0.176	1
5433	A	0.136	0.99
5434	G	0.19	0.93
5435	G	0.176	0.71
5436	U	0.271	0.03
5437	U	0.502	0.05
5438	U	0.448	0.06
5439	G	0.515	0.18
5440	G	0.271	0.28
5441	C	0.326	0.24
5442	A	0.855	0.01
5443	A	1.302	0.01
5444	A	0.814	0.05
5445	G	0.502	0.05
5446	G	0.149	0.26
5447	U	0.095	0.28
5448	C	0	0.89
5449	U	0.041	0.96
5450	U	0.149	0.99
5451	G	0.081	0.99
5452	G	0.068	0.99
5453	G	0.244	0.96
5454	A	0.488	0.93
5455	A	1.004	0.99
5456	U	0.882	0.09
5457	A	0.665	0.01
5458	C	0.136	0

5459	U	0.19	0.77
5460	G	0.203	0.77
5461	G	0	0.71
5462	C	0.231	0.75
5463	A	0.217	0.74
5464	U	0.136	0.86
5465	G	0.326	0.3
5466	A	0.882	0.12
5467	U	0.76	0
5468	G	0.719	0
5469	A	0.692	0
5470	A	0.827	0.11
5471	C	0.529	0.29
5472	A	0.285	0.28
5473	A	0.475	0
5474	G	0.312	0
5475	G	0.176	0
5476	G	0.109	0
5477	A	0.909	0.56
5478	U	0.149	0.94
5479	G	0.027	0.96
5480	U	0.136	0.94
5481	C	0.19	0.99
5482	A	0.244	0.99
5483	C	0	0
5484	C	0.203	0.09
5485	A	0.244	0.94
5486	A	0.366	0.89
5487	G	0.19	0.45
5488	C	0	0.87
5489	U	0.488	0.93
5490	A	0.448	0.85
5491	U	0.068	0.21
5492	G	0.366	0.05
5493	U	0.678	0.04
5494	A	0.719	0.02
5495	A	0.326	0.04
5496	A	0.366	0.04
5497	A	0.434	0.24
5498	U	0.244	0.31
5499	A	0.285	0.23
5500	C	0.231	0.71
5501	A	0.312	0.75

5502	G	0.258	0.67
5503	A	0.448	0.15
5504	U	0.732	0.44
5505	A	0.705	0.36
5506	C	0.203	0.3
5507	U	0.298	0.81
5508	U	0.271	0.85
5509	G	0.109	0.67
5510	U	0.163	0.64
5511	G	0.149	0.25
5512	U	0.081	0.01
5513	U	0.353	0.04
5514	U	0.977	0.05
5515	A	1.166	0.04
5516	A	0.977	0.04
5517	U	0.882	0.26
5518	A	0.475	0.65
5519	C	0.719	0.65
5520	A	0.773	0.63
5521	A	0.773	0.64
5522	A	0.8	0.25
5523	A	0.76	0.15
5524	G	0.407	0.12
5525	G	0.244	0.23
5526	C	0.054	0.26
5527	U	0.19	0.22
5528	U	0.475	0.28
5529	U	0.841	0.25
5530	A	0.827	0.2
5531	U	0.312	0.5
5532	U	0.353	0.39
5533	U	0.637	0.09
5534	A	1.031	0.02
5535	U	0.271	0.03
5536	G	0.258	0.05
5537	C	0.271	0.05
5538	A	0.583	0.07
5539	U	0.637	0.03
5540	U	0.529	0
5541	G	0.353	0
5542	C	0.298	0.01
5543	A	1.004	0.02
5544	A	1.424	0.02

5545	G	0.176	0.06
5546	A	1.004	0.77
5547	A	0.963	0.77
5548	A	1.221	0.17
5549	G	0.76	0.17
5550	G	0.298	0.17
5551	C	0.041	0.03
5552	U	0.258	0.06
5553	G	0.692	0.33
5554	U	0.882	0.33
5555	A	1.275	0.02
5556	G	1.004	0
5557	A	0.502	0.02
5558	U	0.298	0.04
5559	G	0.095	0.38
5560	U	0.203	0.62
5561	C	0	0.9
5562	U	0.393	0.86
5563	A	0.651	0.21
5564	G	0.231	0.16
5565	G	0.231	0.18
5566	G	0.949	0.21
5567	G	0.543	0.21
5568	A	1.289	0.21
5569	A	1.519	0.13
5570	G	0.624	0.26
5571	G	0.651	0.21
5572	A	0.895	0.03
5573	C	1.18	0.29
5574	A	2.835	0.33
5575	U	1.153	0.91
5576	G	0	0.8
5577	G	0.054	0.76
5578	G	0	0.12
5579	G	0.136	0.25
5580	C	1.356	0.25
5581	A	0.109	0.09
5582	G	0	0.49
5583	G	0.014	0.59
5584	G	0.122	0.54
5585	G	0.271	0.77
5586	G	0.176	0.77
5587	A	0.841	0.55

5588	U	2.482	0.21
5589	G	0.38	0.83
5590	G	0.203	0.8
5591	A	0.407	0.74
5592	G	1.411	0.64
5593	A	0.665	0.09
5594	C	3.242	0.16
5595	C	3.133	0.16
5596	A	3.513	0.47
5597	G	2.441	0.63
5598	G	0.651	0.62
5599	A	0	0.11
5600	C	0	0.05
5601	C	0.014	0.11
5602	U	0.136	0.15
5603	C	0.014	0.5
5604	C	0	0.54
5605	U	0.271	0.47
5606	C	0.041	0.35
5607	C	0.014	0.71
5608	U	0.041	0.76
5609	C	0.109	0.77
5610	C	0	0.74
5611	U	0.027	0.64
5612	C	0.014	0.74
5613	C	0.176	0.71
5614	C	0.38	0.6
5615	C	0.882	0.64
5616	C	0.326	0.56
5617	U	0.827	0.25
5618	C	0.949	0.76
5619	C	1.031	0.84
5620	A	1.058	0.9
5621	G	0.604	0.46
5622	G	0.42	0.43
5623	A	0.624	0.16
5624	C	1.044	0.26
5625	U	0.014	0.25
5626	A	0.163	0.02
5627	G	0.203	0.02
5628	C	0.529	0.09
5629	A	0.359	0.97
5630	U	0.393	0.97

5631	A	0.312	0.02
5632	A	0.732	0
5633	A	0.434	0
5634	U	0.014	0
5635	G	0.827	0
5636	G	0.665	0
5637	A	0.529	0.02
5638	A	0.366	0.94
5639	G	0.76	0.94
5640	A	0.543	0.08
5641	A	1.221	0.02
5642	A	0.882	0.02
5643	G	0.475	0.04
5644	A	0.068	0.03
5645	C	0.136	0.01
5646	C	0.19	0.01
5647	U	0.326	0
5648	C	0.637	0.01
5649	C	0.719	0.01
5650	A	0.515	0.03
5651	G	1.275	0.05
5652	A	1.424	0.03
5653	A	1.112	0
5654	A	0.42	0
5655	A	0.787	0
5656	U	0.434	0
5657	G	0.732	0
5658	A	1.004	0
5659	A	0.922	0
5660	G	0.882	0
5661	G	0.977	0.03
5662	A	1.085	0.63
5663	C	0.61	0.97
5664	C	0.136	0.67
5665	A	0.922	0.36
5666	C	0	0.29
5667	A	0.448	0.28
5668	A	0.502	0.03
5669	A	0.651	0.03
5670	G	0.326	0.06
5671	G	0.109	0.04
5672	G	0.19	0.04
5673	A	0.922	0.01

5674	A	0.719	0.01
5675	C	0	0.07
5676	C	0.339	0.08
5677	A	0.19	0.08
5678	U	0	0.4
5679	G	1.221	0.71
5680	G	0.827	0.51
5681	G	1.438	0.21
5682	A	0.827	0.03
5683	U	0.271	0.02
5684	G	1.506	0.02
5685	A	1.777	0.01
5686	A	0.732	0.05
5687	U		0.41
5688	G	0.068	0.6
5689	G	0.583	0.6
5690	G	0.827	0.1
5691	U	1.302	0.16
5692	A	1.736	0.14
5693	G	0.963	0.09
5694	U	0.787	0.11
5695	G	1.465	0.1
5696	G	0.475	0.08
5697	A	0	0.03
5698	G	0.122	0.02
5699	G		0.05
5700	U	0.42	0.11
5701	U	0.61	0.13
5702	C	0.936	0.38
5703	U	0.977	0.59
5704	G	0.217	0.29
5705	G	0.515	0.22
5706	A	0.298	0.11
5707	A	0.868	0.07
5708	G	0.977	0.05
5709	A	0.841	0.18
5710	A	0.705	0.2
5711	C	0.149	0.25
5712	U	0.095	0.39
5713	G	0.543	0.41
5714	A	0.502	0.31
5715	A	0.821	0.09
5716	A	0.821	0.06

5717	G	0.929	0.01
5718	A	1.044	0.49
5719	A	0.861	0.69
5720	G	0.943	0.61
5721	A	0.848	0.43
5722	A	0.658	0.55
5723	G	0.583	0.48
5724	C	0.312	0.32
5725	U	0.814	0.2
5726	U	1.062	0.44
5727	U	1.515	0.52
5728	A	1.723	0.69
5729	A	0.977	0.72
5730	A	0.85	0.67
5731	A	0.918	0.07
5732	C	0.723	0.03
5733	A	0.515	0.03
5734	U	0.271	0.2
5735	U	0.515	0.9
5736	U	0.981	0.98
5737	U	1.221	0.96
5738	G	1.289	0.32
5739	A	0.972	0.19
5740	U	0.203	0
5741	C	0.529	0.01
5742	C	0.298	0.01
5743	U	0.549	0
5744	C	0.563	0
5745	G	0.226	0.3
5746	C	0.09	0.33
5747	U	0.127	0.58
5748	U	0.326	0.6
5749	G	0.264	0.52
5750	C	0.136	0.7
5751	U	0.565	0.65
5752	A	0.85	0.71
5753	A	0.628	0.63
5754	C	0.244	0.06
5755	U	0.212	0.07
5756	G	0.398	0.12
5757	C	0.402	0.11
5758	A	0.665	0.01
5759	C	0.312	0.23

5760	U	0.529	0.88
5761	U	0.237	0.95
5762	G	0.461	0.62
5763	G	0.57	0.64
5764	U	0.552	0.48
5765	A	0.832	0.36
5766	A	0.918	0.28
5767	U	0.524	0.01
5768	C	0.443	0.88
5769	A	0.678	0.91
5770	U	0.344	0.96
5771	A	0.66	0.88
5772	U	0.407	0.81
5773	C	0.332	0.89
5774	U	0.441	0.86
5775	A	1.252	0.61
5776	U	0.986	0.27
5777	A	1.659	0.2
5778	A	1.049	0.09
5779	U	0.827	0.12
5780	A	0.714	0.23
5781	G	0.466	0.17
5782	A	1.017	0.04
5783	C	0.497	0.04
5784	A	0.561	0.23
5785	U	0.353	0.66
5786	G	0.52	0.95
5787	G	0.549	0.85
5788	A	0.617	0.52
5789	G	0.42	0.22
5790	A	0.414	0.15
5791	C	0.237	0.59
5792	A	0.373	0.59
5793	C	0.102	0.68
5794	C	0.041	0.76
5795	C	0.115	0.83
5796	U	0.353	0.82
5797	U	0.326	0.74
5798	G	0.644	0.6
5799	A	0.977	0.63
5800	G	0.665	0.69
5801	G	0.475	0.67
5802	G	0.346	0.92

5803	A	0.434	0.98
5804	G	0.21	0.79
5805	C	0.495	0.13
5806	A	0.814	0.06
5807	G	0.305	0.13
5808	G	0.692	0.31
5809	A	0.705	0.27
5810	G	0.719	0.13
5811	A	0.943	0.09
5812	A	0.617	0.17
5813	C	0.149	0.76
5814	U	0.346	0.92
5815	C	0.631	0.87
5816	A	1.004	0.4
5817	U	0.597	0.76
5818	U	2.028	0.94
5819	A	1.689	0.96
5820	G	0.936	0.86
5821	A	0.814	0.88
5822	A	0.766	0.72
5823	U	0.136	0.78
5824	C	0.068	0.71
5825	C	0.027	0.64
5826	U	0.075	0.58
5827	C	0.068	0.4
5828	C	0.149	0.4
5829	A	0.8	0.43
5830	A	1.099	0.43
5831	C	0.699	0.4
5832	G	0.936	0.74
5833	A	0.787	0.89
5834	G	0.522	0.58
5835	C	0.237	0.1
5836	G	0.176	0.09
5837	C	0.129	0.34
5838	U	0.088	0.55
5839	C	0.183	0.68
5840	U	1.058	0.64
5841	U	0.882	0.59
5842	C	0.407	0.17
5843	A	0.719	0.01
5844	U	0.339	0.01
5845	G	0.461	0.14

5846	C	0.42	0.82
5847	A	0.841	0.75
5848	U	0.529	0.39
5849	U	0.665	0.48
5850	U	0.597	0.55
5851	C	0.732	0.52
5852	A	0.732	0.04
5853	G	0.502	0.02
5854	A	0.556	0.21
5855	G	0.366	0.33
5856	G	0.448	0.14
5857	C	0.434	0.06
5858	G	0.163	0.22
5859	G	0.122	0.58
5860	A	0.19	0.52
5861	U	0.095	0.8
5862	G	0.176	0.8
5863	C	0.122	0.06
5864	A	0.109	0
5865	U	0.081	6.43E-05
5866	C	0.081	0
5867	C	0.149	0.01
5868	A	0.312	0.01
5869	C	0.136	0.49
5870	U	0.136	1
5871	C	0.041	0.97
5872	C	0.353	0.78
5873	A	0.515	0.91
5874	G	0.732	0.84
5875	A	0.827	0.02
5876	A	0.936	0.03
5877	U	0.543	0.14
5878	C	0.271	0.3
5879	G	0.285	0.87
5880	G	0.136	0.84
5881	C	0.068	0.09
5882	C	0.217	0.11
5883	A	0.258	0.11
5884	A	0.366	0.07
5885	C	0.149	0.04
5886	C	0	0.02
5887	U	0	0.04
5888	G	0	0.35

5889	G		0.8
5890	G	0.095	0.83
5891	G	0.054	0.7
5892	G	0.041	0.92
5893	A	0.027	0.93
5894	G	0.027	0.86
5895	G	0.136	0.78
5896	A	0.597	0.03
5897	A	1.126	0
5898	A	0.705	0.01
5899	U	0.529	0.07
5900	C	0.068	0.81
5901	C	0	0.84
5902	U	0	0.93
5903	C	0	0.95
5904	U	0.068	0.69
5905	C	0.068	0.79
5906	U	0.217	0.79
5907	C		0.38
5908	A	0.231	0.13
5909	G	0.203	0.13
5910	C	0.244	0.08
5911	U	0.678	0.11
5912	A	1.228	0.06
5913	U	0.292	0.05
5914	A	0.109	0.06
5915	C	0.088	0.27
5916	C	0.034	0.28
5917	G	0.136	0.19
5918	C	0.129	0.05
5919	C	0.014	0.22
5920	C	0	0.48
5921	U	0.149	0.3
5922	C	0.081	0.86
5923	U	1.16	0.92
5924	A	1.533	0.79
5925	G	1.099	0.95
5926	A	1.37	0.95
5927	A	0.346	0.01
5928	G	0.007	0
5929	C	0.081	0.24
5930	A	0.095	0.25
5931	U	0.034	0.71

5932	G	0.047	0.73
5933	C	0.183	0.74
5934	U	0.943	0.68
5935	A	0.977	0.46
5936	U	1.234	0.55
5937	A	0.855	0.41
5938	A	0.712	0.03
5939	C	0.217	0.03
5940	A	0.19	0.34
5941	C	0.041	0.46
5942	A	0.081	0.24
5943	U	0.068	0.27
5944	G	0.027	0.27
5945	C	0.034	0.12
5946	U	0.366	0.61
5947	A	1.078	0.63
5948	U	0.644	0.33
5949	U	0.42	0.7
5950	G	0.902	0.78
5951	U	1.77	0.67
5952	A	1.194	0.64
5953	A	0.929	0.02
5954	A	1.051	0
5955	A	1.166	0
5956	A	1.343	0.01
5957	G	0.977	0.09
5958	U	0.488	0.93
5959	G	0.387	0.95
5960	U	0.312	0.86
5961	U	1.004	0.66
5962	G	0.529	0.24
5963	C	0.041	0.23
5964	U	0.122	0.09
5965	A	0.298	0.08
5966	C	0.027	0.04
5967	C	0.095	0.45
5968	A	0.102	0.48
5969	U	0.339	0.3
5970	U	0.529	0.11
5971	G	0.007	0.3
5972	C	0.014	0.3
5973	C	0.088	0.42
5974	A	0.095	0.44

5975	G	0.047	0.26
5976	U	0.163	0.83
5977	U	0.855	0.88
5978	U	0.909	0.91
5979	U	0.746	0.94
5980	G	0.434	0.81
5981	U	0.095	0.81
5982	U	0.285	0.93
5983	U	0.366	0.85
5984	U	0.271	0.32
5985	C	0.326	0.15
5986	U	1.099	0.11
5987	U	0.665	0.01
5988	A	1.892	0.08
5989	A	1.031	0.48
5990	A	1.024	0.83
5991	A	1.16	0.59
5992	A	1.194	0.2
5993	A	0.732	0.15
5994	G	0.292	0.15
5995	G	0.115	0.09
5996	C	0.102	0.02
5997	U	0.21	0.07
5998	U	0.271	0.08
5999	G	0.21	0.02
6000	G	0.163	0.1
6001	G	0.19	0.16
6002	G	0.353	0.21
6003	A	0.57	0.7
6004	U	0.536	0.78
6005	A	0.922	0.86
6006	U	0.292	0.88
6007	G	0.631	0.86
6008	U	0.312	0.92
6009	U	0.868	0.36
6010	A	1.119	0.35
6011	U	0.604	0.74
6012	G	0.739	0.7
6013	A	0.366	0.66
6014	G	0.353	0.95
6015	C	0.149	0.95
6016	A	0.543	0.83
6017	A	0.624	0.02

6018	U	0.244	0.01
6019	C	0.285	0
6020	A	0.719	0.02
6021	C	0.38	0.08
6022	G	1.139	0.1
6023	A	0.936	0.04
6024	A	1.004	0.06
6025	A	0.977	0.11
6026	G	0.651	0.09
6027	A	0.8	0.04
6028	G	0.651	0.59
6029	A	0.963	0.59
6030	A	0.868	0.34
6031	G	0.739	0.38
6032	A	1.01	0.09
6033	A	0.814	0.03
6034	G	0.983	0.03
6035	A	1.383	0.02
6036	A	0.712	0.02
6037	C	0	0.4
6038	U	0.285	0.98
6039	C	0.109	0.79
6040	C	0.353	0.24
6041	G	1.187	0.02
6042	A	1.383	0.01
6043	A	0.773	0.01
6044	A	1.133	0.03
6045	A	1.397	0.03
6046	A	1.356	0.01
6047	G	0.509	0.01
6048	G	0.149	0.01
6049	C	0	0.03
6050	U	0.292	0.97
6051	A	0.834	0.95
6052	A	1.058	0.68
6053	G	0.678	0.73
6054	G	0.122	0.67
6055	C	0	0.16
6056	U	0.38	0.16
6057	A	1.35	0.05
6058	A	1.2	0.02
6059	U	1.051	0.01
6060	A	0.529	0

6061	C	0	0.01
6062	A	0.414	0.01
6063	U	0.034	0.56
6064	C	0	0.79
6065	U	0.251	0.8
6066	U	0.136	0.34
6067	C	0.088	0.85
6068	U	0.346	0.85
6069	G	0.509	0.03
6070	C	0.129	0.11
6071	A	0.176	0.23
6072	U	0.285	0.2
6073	C	0.244	0.06
6074	A	1.058	0.11
6075	A	1.492	0.08
6076	A	1.261	0.08
6077	C	0.637	0.04
6078	A	0.76	0.08
6079	A	0.604	0.45
6080	G	0.353	0.6
6081	U	1.37	0.22
6082	A	1.072	0.22
6083	A	0.631	0.61
6084	G	0.556	0.63
6085	U	1.139	0.98
6086	A	1.031	0.97
6087	U	0.515	0.05
6088	G	0.339	0.02
6089	G	0.332	0
6090	G	0.366	7.16E-05
6091	A	0.556	7.10E-05
6092	U	0.393	0
6093	G	0.509	0
6094	U	0.258	0
6095	C	0.156	0
6096	U	0.217	0
6097	U	0.285	0.02
6098	G	0.332	0.04
6099	G	0.271	0.03
6100	G	0.556	0
6101	A	0.692	0.99
6102	A	0.861	0.99
6103	U	0.434	0.05

6104	C	0.414	0.04
6105	A	0.868	0
6106	G	0.502	4.19E-05
6107	C	0.115	0
6108	U	0.576	0
6109	G	0.285	0.05
6110	C	0.509	0.05
6111	U	1.078	0.99
6112	U	1.153	0.98
6113	A	0.482	0
6114	U	0.298	0.03
6115	C	0.644	0.04
6116	G	0	0.02
6117	C	0.102	0
6118	C	0.149	7.98E-05
6119	A	0.427	0
6120	U	0.197	0
6121	C	0.075	0
6122	U	0.387	0
6123	U	0.332	0.15
6124	G	0.556	0.2
6125	C	0.163	0.23
6126	U	0.57	0.26
6127	U	0.244	0.11
6128	U	1.234	0.08
6129	U	1.912	0.64
6130	A	1.621	0.66
6131	A	1.302	0.39
6132	G	0.549	0.24
6133	U	0.278	0.2
6134	G	0.312	0.17
6135	U	0.109	0.06
6136	C	0.061	0.25
6137	U	0.685	0.7
6138	A	1.099	0.62
6139	U	0.393	0.01
6140	G	0.549	0.03
6141	G	0.319	0.03
6142	G	0.366	0.01
6143	A	0.631	0.01
6144	U	0.203	0.04
6145	C	0.109	0.04
6146	U	0.909	0.05

6147	A	0.888	0.05
6148	U	0.549	0.04
6149	U	0.346	0.05
6150	G	0.624	0.05
6151	U	0.515	0.05
6152	A	0.827	0.06
6153	C	0.054	0.08
6154	U	0.122	0.95
6155	C	0.176	0.95
6156	U	1.085	0.68
6157	A	1.451	0.38
6158	U	1.411	0.34
6159	A	1.302	0.14
6160	U	0.353	0.13
6161	G	0.19	0.17
6162	U	0.149	0.15
6163	C	0.014	0.02
6164	A	0.393	0.18
6165	C	0.353	0.21
6166	A	0.787	0.66
6167	G	0.502	0.7
6168	U	0	0.68
6169	C	0	0.29
6170	U	0.258	0.75
6171	U	0.461	0.79
6172	U	0.529	0.58
6173	U	1.004	0.74
6174	A	1.438	0.81
6175	U	0.448	0.65
6176	G	0.271	0.61
6177	G	0.312	0.29
6178	U	0.027	0.15
6179	G	0.407	0.2
6180	U	0.543	0.22
6181	A	0.963	0.17
6182	C	0.515	0.2
6183	C	0.488	0.27
6184	A	1.072	0.08
6185	G	0.081	0.09
6186	C	0.041	0.47
6187	U	0.244	0.67
6188	U	0.366	0.76
6189	G	0.122	0.81

6190	G	0.244	0.74
6191	A	0.407	0.66
6192	G	0.109	0.64
6193	G	0	0.21
6194	A	0.543	0.37
6195	A	0.692	0.48
6196	U	0.393	0.44
6197	G	0.597	0.19
6198	C	0.163	0.16
6199	G	1.085	0.27
6200	A	0.488	0.16
6201	C	0	0.01
6202	A	0.868	0.06
6203	A	0.271	0.37
6204	U	0.353	0.91
6205	U	0.312	0.94
6206	C	0.244	0.71
6207	C	0.244	0.67
6208	C	0.109	0.63
6209	C	0	0.62
6210	U	0.231	0.15
6211	C	0.122	0.09
6212	U	0.231	0.36
6213	U	0.027	0.51
6214	U	0.651	0.56
6215	U	0.326	0.77
6216	G	0.556	0.76
6217	U	0.665	0.8
6218	G	0.298	0.35
6219	C	0	0.05
6220	A	0.773	0.05
6221	A	0.719	0.03
6222	C	0.068	0.1
6223	C	0.014	0.38
6224	A	0.773	0.39
6225	A	0.936	0.16
6226	G	0.909	0.13
6227	A	1.112	0.12
6228	A	1.533	0.43
6229	U	1.044	0.41
6230	A	1.668	0.52
6231	G	0.746	0.48
6232	G	0.203	0.41

6233	G	0.068	0.27
6234	A	0.909	0.23
6235	U	0.705	0.16
6236	A	0.651	0.17
6237	C	0.312	0.23
6238	U	0.637	0.23
6239	U	0.665	0.22
6240	G	0.081	0.85
6241	G	0.244	0.9
6242	G	0.041	0.74
6243	G	0.163	0.71
6244	A	0.8	0.62
6245	A	0.407	0.57
6246	C	0.461	0.13
6247	A	0.895	0.09
6248	A	1.736	0.28
6249	C	0.136	0.29
6250	U	0.271	0.29
6251	C	0.407	0.32
6252	A	0.773	0.33
6253	G	0.705	0.38
6254	U	0.502	0.55
6255	G	0.488	0.3
6256	C	0.122	0.23
6257	C	0.217	0.21
6258	U	0.434	0.15
6259	A	0.515	0.08
6260	C	0.244	0.21
6261	C	0.109	0.29
6262	A	0.434	0.35
6263	G	0.42	0.27
6264	A	1.356	0.19
6265	U	0.42	0.24
6266	A	0.719	0.65
6267	A	1.207	0.67
6268	U	0.285	0.76
6269	G	0.014	0.17
6270	G	0.149	0.5
6271	U	0.068	0.54
6272	G	0.366	0.08
6273	A	0.61	0.38
6274	U	0.624	0.41
6275	U	0.637	0.45

6276	A	0.515	0.56
6277	U	0.651	0.46
6278	U	0.76	0.03
6279	C	0.136	0.03
6280	A	1.275	0.01
6281	G	0.732	0.01
6282	A	1.587	0
6283	A	0.705	0
6284	G	0.909	0
6285	U	0.529	0
6286	G	0.285	0
6287	G	0.203	0
6288	C	0	0
6289	C	0.353	0.04
6290	C	0.163	0.1
6291	U	0.366	0.78
6292	U	0.665	0.9
6293	A	1.017	0.25
6294	A	0.719	0.17
6295	U	0.407	0.02
6296	G	0.42	0.02
6297	U	0.014	0.65
6298	U	0.217	0.66
6299	A	0.814	0.15
6300	C	0	0.3
6301	A	0.963	0.48
6302	G	0.393	0.29
6303	A	0.882	0.14
6304	A	0.637	0.05
6305	A	0.922	0.07
6306	G	0.448	0.09
6307	C	0.136	0.26
6308	U	0.095	0.52
6309	U	0.855	0.62
6310	U	0.366	0.44
6311	G	0.515	0.35
6312	A	0.556	0.41
6313	U	0.271	0.39
6314	G	0.068	0.37
6315	C	0.081	0.51
6316	C	0.081	0.53
6317	U	0.041	0.73
6318	G	0.855	0.73

6319	G	0.909	0.55
6320	A	0.827	0.12
6321	A	0.461	0.49
6322	U	0.4	0.5
6323	A	0.576	0.32
6324	A	0.76	0.24
6325	U	0.692	0.27
6326	A	0.793	0.38
6327	C	0.047	0.46
6328	A	0.305	0.47
6329	G	0.882	0.28
6330	U	0.495	0.31
6331	C	0.366	0.13
6332	A	0.522	0.26
6333	C	0.081	0.48
6334	A	0.888	0.52
6335	G	0.732	0.22
6336	A	0.97	0.18
6337	A	0.475	0.3
6338	C	0.163	0.43
6339	A	0.095	0.37
6340	G	0.339	0.04
6341	G	0.258	0.09
6342	C	0.258	0.28
6343	A	0.298	0.37
6344	A	0.393	0.34
6345	U	0.326	0.36
6346	A	0.461	0.31
6347	G	0.353	0.12
6348	A	0.624	0.09
6349	G	0.285	0.01
6350	G	0.434	0.05
6351	A	0.543	0.1
6352	U	0.285	0.82
6353	G	0.448	0.82
6354	U	0	0.66
6355	A	0.475	0.11
6356	U	0	0.69
6357	G	0.366	0.71
6358	G	0.42	0.45
6359	C	0.244	0.19
6360	A	0.461	0.29
6361	A	0.556	0.43

6362	C	0.353	0.37
6363	U	0.244	0.73
6364	C	0.203	0.64
6365	U	0.624	0.79
6366	U	1.221	0.66
6367	U	0.895	0.1
6368	G	0.556	0.47
6369	A	0.203	0.53
6370	G	0.461	0.1
6371	A	0.529	0.15
6372	C	0.136	0.14
6373	C	0.217	0.05
6374	U	0.109	0.59
6375	C	0.258	0.57
6376	A	0.57	0.29
6377	A	0.692	0.6
6378	U	1.072	0.62
6379	A	1.316	0.48
6380	A	0.434	0.23
6381	A	0.461	0.23
6382	G	0.122	0.21
6383	C	0.163	0.81
6384	C	0.122	0.81
6385	U	0.176	0.81
6386	U	0.203	1
6387	G	0.407	0.97
6388	U	0.556	0.96
6389	G	0.339	0.71
6390	U	0.42	0.65
6391	A	1.004	0.41
6392	A	1.017	0.36
6393	A	0.488	0.27
6394	A	0.583	0.2
6395	U	0.705	0.2
6396	U	0.515	0.24
6397	A	0.095	0.08
6398	U	0.163	0.05
6399	C	0.149	0.05
6400	C	0.041	0.01
6401	C	0.231	0.01
6402	C	0	0.01
6403	A	0.312	0.01
6404	U	0.163	0.03

6405	U	0.882	0.06
6406	A	2.184	0.06
6407	U	0.42	0.13
6408	G	0.19	0.12
6409	C	0	0.1
6410	A	0.38	0.1
6411	U	0.176	0.15
6412	U	0.461	0.13
6413	A	0.787	0.45
6414	C	0.298	0.84
6415	U	0.583	0.78
6416	A	0.963	0.77
6417	U	0.434	0.03
6418	G	1.112	0.01
6419	A	1.329	0.05
6420	G	1.072	0.15
6421	A	0.624	0.97
6422	U	0.244	0.96
6423	G	0	0.86
6424	C	0.027	0.46
6425	A	0.637	0.13
6426	A	1.275	0.02
6427	U	0.38	0.01
6428	A	1.261	0
6429	A	0.936	0
6430	A	1.153	0.04
6431	A	0.963	0.04
6432	G	0.678	0.04
6433	U	0.393	0.04
6434	G	1.438	4.88E-06
6435	A	1.329	1.98E-06
6436	G	1.587	2.00E-06
6437	A	1.411	0
6438	C	0.543	0
6439	A	1.004	0
6440	G	0.936	0
6441	A	1.37	0
6442	U	1.031	0
6443	A	1.451	0
6444	G	0.597	0
6445	A	0.149	0
6446	U	0.068	0
6447	G	0.136	0

6448	G	0.502	0
6449	G	0.326	0
6450	G	0.054	0
6451	A	0.163	4.97E-06
6452	U	0.041	1.40E-05
6453	U	0.855	0
6454	G	1.207	0
6455	A	1.017	0
6456	C	0.515	4.01E-05
6457	A	1.207	0
6458	A	0.665	0
6459	A	1.194	0
6460	A	0.651	0
6461	U	0.475	0
6462	C	0.583	1.09E-05
6463	A	0.882	0
6464	A	1.316	0
6465	U	1.519	3.99E-05
6466	A	2.116	4.99E-06
6467	A	1.465	0
6468	C	1.017	0
6469	A	1.302	0
6470	A	0.922	0
6471	C	0.732	3.89E-06
6472	A	1.194	0
6473	A	0.909	0
6474	C	0.665	7.90E-05
6475	A	0.8	0
6476	G	0.339	0
6477	C	0.068	0
6478	A	0.502	0
6479	U	0.149	0
6480	C	0.298	9.64E-06
6481	A	0.977	1.63E-05
6482	A	0.705	0
6483	C	0.502	0
6484	A	1.126	0
6485	A	0.76	0
6486	C	0.583	4.69E-05
6487	A	1.126	0
6488	U	0.285	0
6489	C	0.448	0
6490	A	0.746	0

6491	A	0.61	0
6492	C	0.231	0.01
6493	G	0.76	0.05
6494	A	0.746	0.04
6495	C	0.488	0.02
6496	A	0.814	0.39
6497	G	0.068	0.39
6498	C	0.014	0.04
6499	A	0.19	0
6500	U	0.109	0
6501	C	0.217	0.04
6502	A	0.583	0.07
6503	G	0.868	0.05
6504	C	0.136	0.23
6505	A	1.072	0.38
6506	A	1.017	0.18
6507	A	1.316	0
6508	A	0.827	0
6509	G	1.004	3.91E-06
6510	U	1.519	9.96E-07
6511	A	1.302	0
6512	G	0.895	0
6513	A	0.637	9.99E-07
6514	C	0.543	9.98E-07
6515	A	1.044	5.59E-07
6516	U	0.556	0
6517	G	0.244	3.79E-06
6518	G	0.081	0.01
6519	U	0.054	0.01
6520	C	0.027	0
6521	A	1.316	0
6522	A	1.506	2.50E-06
6523	U	0.692	0
6524	G	0.949	0
6525	A	1.085	0.01
6526	G	0.515	0.01
6527	A	0.339	0
6528	C	0.109	0
6529	U	0.814	3.59E-06
6530	A	1.221	5.99E-07
6531	G	0.244	4.75E-07
6532	U	0.217	2.13E-07
6533	U	0.054	5.06E-05

6534	C	0.163	0
6535	U	0.488	0
6536	U	0.244	8.33E-05
6537	G	0.366	4.60E-07
6538	U	0.787	4.24E-07
6539	A	1.316	5.09E-05
6540	U	0.434	5.21E-05
6541	A	0.38	0
6542	G	0.122	0
6543	C	0.095	1.52E-05
6544	C	0.122	1.99E-05
6545	C	0.258	1.90E-05
6546	A	1.017	3.11E-05
6547	G	0.583	0
6548	G	0.434	0
6549	A	0.773	0.16
6550	U	0.8	0.17
6551	A	1.004	0.02
6552	A	0.814	0.02
6553	U	0.705	0.59
6554	U	0.393	0.6
6555	G	0.895	0.01
6556	C	0.393	0
6557	A	0.678	0
6558	C	0.258	0.01
6559	A	0.38	0.57
6560	G	0.217	0.58
6561	G	0.176	0.2
6562	C	0.027	0.18
6563	U	0.075	0.01
6564	U	0.543	0.03
6565	G	1.662	0.03
6566	G	1.716	0.01
6567	A	1.024	9.19E-06
6568	A	0.298	1.51E-07
6569	C	0.068	2.15E-06
6570	A	0.482	0
6571	A	0.841	0
6572	G	0.692	7.24E-06
6573	A	0.427	2.64E-06
6574	G	0.176	3.13E-07
6575	C	0	2.49E-07
6576	A	0.692	0

6577	A	0.631	0
6578	A	0.895	2.34E-05
6579	U	0.766	5.57E-05
6580	G	0.604	5.53E-05
6581	A	0.685	6.35E-05
6582	U	0.339	0
6583	A	1.309	0
6584	A	0.515	0
6585	G	0	0.38
6586	C	0	0.78
6587	U	0	0.76
6588	G	0.197	0.09
6589	U	0	0.04
6590	A	0.949	0.01
6591	A	0.929	0.01
6592	A	0.617	0.79
6593	U	0.305	0.9
6594	U	0.726	0.47
6595	C	0	0.07
6596	A	0.956	0.08
6597	A	0.651	0.09
6598	C	0	0.07
6599	A	1.051	0.19
6600	U	0.271	0.19
6601	G	0.427	0.01
6602	A	0.631	0.02
6603	C	0.305	0.02
6604	A	0.509	0.02
6605	G	0.115	0.02
6606	G	0.264	0.02
6607	G	0	0.04
6608	U	0.231	0.29
6609	U	0.583	0.77
6610	A	1.35	0.54
6611	A	0.705	0.05
6612	A	1.39	0.07
6613	A	0.739	0.07
6614	A	1.004	0.07
6615	G	0.827	0.04
6616	A	0.909	0.16
6617	G	0.895	0.16
6618	A	0.719	0.07
6619	C	0	0.57

6620	A	0.678	0.55
6621	A	1.099	0.07
6622	G	0.244	0.02
6623	A	1.261	0.04
6624	A	1.478	0.04
6625	A	1.343	0.01
6626	A	1.261	0.01
6627	A	2.17	0.01
6628	A	0.285	0
6629	G	0.231	0
6630	A	0.217	0.11
6631	G	0.149	0.12
6632	U	0.19	0.91
6633	A	0.353	0.98
6634	C	0.19	0.86
6635	A	0.909	0.65
6636	A	0.895	0.15
6637	U	0.746	0.14
6638	G	1.248	0.04
6639	A	0.977	0
6640	A	1.153	0
6641	A	0.827	0.01
6642	C	0.203	0.01
6643	U	0.543	0.76
6644	U	0.285	0.82
6645	G	0.827	0.15
6646	G	0.258	0.29
6647	U	0.109	0.8
6648	A	0.366	0.77
6649	C	0.095	0.09
6650	U	0.149	0.16
6651	C	0	0.2
6652	U	0.366	0.08
6653	G	1.072	0
6654	C	0.42	0.3
6655	A	1.248	0.31
6656	G	1.18	0.13
6657	A	1.18	0.23
6658	U	0.461	0.21
6659	U	0.258	0.2
6660	U	0.19	0.43
6661	G	0.285	0.43
6662	G	0.244	0.07

6663	U	0.136	0.65
6664	A	0.637	0.65
6665	U	0.122	0.59
6666	G	0.353	0.64
6667	U	0.217	0.66
6668	G	0.665	0.54
6669	A	1.194	0.49
6670	A	1.058	0.49
6671	C	0.217	0
6672	A	0.732	7.25E-06
6673	A	0.651	9.96E-06
6674	G	0.543	8.86E-06
6675	G	0.339	1.81E-06
6676	G	0.692	4.72E-06
6677	A	0.637	4.64E-06
6678	A	1.017	1.87E-06
6679	U	0.57	6.95E-07
6680	A	0.977	7.33E-07
6681	A	0.827	1.82E-05
6682	C	0.258	0
6683	A	0.515	0
6684	C	0.014	8.91E-06
6685	U	0.122	5.89E-06
6686	G	0	0
6687	G	0.054	0
6688	U	0.109	0
6689	A	0.651	0
6690	A	0.637	1.04E-05
6691	U	0.461	7.44E-06
6692	G	1.438	2.89E-06
6693	A	1.519	9.65E-06
6694	A	1.912	0
6695	A	0.949	0
6696	G	0.637	0.01
6697	U	0.407	0.01
6698	A	0.827	0.01
6699	G	0.665	0.01
6700	A	1.641	0.67
6701	U	0.448	0.93
6702	G	0.136	0.41
6703	U	0.068	0.36
6704	U	0.081	0.28
6705	A	0.353	0.19

6706	C	0.027	0.16
6707	A	0.448	0.64
6708	U	0.61	0.6
6709	G	1.166	0.08
6710	A	1.18	0.41
6711	A	1.044	0.41
6712	C	0.176	0.21
6713	C	0.19	0.19
6714	A	1.058	0.17
6715	C	0.203	0.17
6716	U	0.095	0.39
6717	G	0.122	0.58
6718	U	0.095	0.58
6719	A	0.407	0.58
6720	A	0.461	0.44
6721	C	0.258	0.64
6722	A	0.42	0.93
6723	C	0.095	0.94
6724	U	0.393	0.93
6725	U	0.488	0.85
6726	C	0.122	0.18
6727	U	0.434	0.16
6728	G	0.203	0.27
6729	U	0.244	0.33
6730	U	0.393	0.11
6731	A	0.841	0.53
6732	U	0.366	0.53
6733	C	0.19	0.13
6734	C	0.298	0.01
6735	A	0.637	0.01
6736	A	0.705	0.02
6737	G	0.882	0.12
6738	A	0.814	0.2
6739	G	0.38	0.62
6740	U	0.081	0.64
6741	C	0.054	0.14
6742	U	1.058	0.83
6743	U	0.461	0.95
6744	G	0.773	0.98
6745	U	0.339	0.99
6746	G	0.461	0.69
6747	A	0.787	0.47
6748	C	0.176	0.5

6749	A	0.949	0.79
6750	A	0.963	0.84
6751	A	0.855	0.73
6752	C	0.095	0.39
6753	A	0.868	0.45
6754	U	0.543	0.33
6755	U	0.597	0.54
6756	A	1.411	0.5
6757	U	0.651	0.68
6758	U	0.637	0.74
6759	G	0.637	0.54
6760	G	0.434	0.12
6761	G	0.42	0.12
6762	A	0.868	0.15
6763	U	0.176	0.15
6764	G	0.163	0.13
6765	C	0.041	0.12
6766	U	0.475	0.14
6767	A	0.624	0.19
6768	U	0.448	0.55
6769	U	0.827	0.74
6770	A	0.99	0.48
6771	G	0.678	0.24
6772	A	0.705	0.33
6773	U	0.312	0.61
6774	U	0.665	0.69
6775	U	0.57	0.91
6776	A	1.18	0.86
6777	G	0.461	0.85
6778	G	0.583	0.88
6779	U	0.339	0.91
6780	A	0.597	0.43
6781	U	0.407	0.27
6782	U	0.448	0.65
6783	G	0.692	0.68
6784	U	0.298	0.57
6785	G	0.434	0.46
6786	C	0.149	0.3
6787	A	0.244	0.83
6788	C	0.041	0.77
6789	C	0.122	0.7
6790	U	0.57	0.01
6791	C	0.109	0.03

6792	C	0.136	0.05
6793	A	0.366	0.09
6794	G	0.244	0.77
6795	G	0.231	0.79
6796	U	0.312	0.74
6797	U	0.665	0.24
6798	A	1.628	0.45
6799	U	0.99	0.47
6800	G	0.068	0.44
6801	C	0.136	0.39
6802	U	0.353	0.46
6803	U	0.271	0.84
6804	U	0.393	0.71
6805	G	0.095	0.15
6806	C	0.326	0.2
6807	U	1.153	0.44
6808	U	0.963	0.5
6809	A	0.502	0.46
6810	G	0.882	0.39
6811	A	0.203	0.27
6812	U	0.583	0.33
6813	G	0.339	0.48
6814	U	0.841	0.37
6815	A	0.977	0.39
6816	A	0.461	0.38
6817	U	0.814	0.66
6818	G	0.909	0.61
6819	A	0.217	0.36
6820	C	0.692	0.29
6821	A	0.38	0.28
6822	C	1.302	0.19
6823	A	0.841	0.45
6824	A	1.397	0.45
6825	A	1.316	0.23
6826	U	0.963	0.21
6827	U	1.031	0.36
6828	A	0.488	0.36
6829	U	0.705	0.26
6830	U	0.515	0.44
6831	C	0.543	0.5
6832	A	0.366	0.48
6833	G	0.054	0.27
6834	G	0	0.25

6835	C	0.475	0.26
6836	U	1.085	0.38
6837	U	0.787	0.45
6838	U	0.895	0.33
6839	A	0.732	0.38
6840	U	0.217	0.33
6841	G	0	0.3
6842	C		0.42
6843	C	0.312	0.43
6844	U	1.289	0.41
6845	A	0.963	0.43
6846	A	0.936	0.43
6847	A	0.244	0.43
6848	U	0.353	0.46
6849	G	0	0.46
6850	U	0.176	0.44
6851	U	0.217	0.94
6852	C	0.76	0.94
6853	U	1.641	0.53
6854	A	2.211	0.58
6855	A	1.241	0.55
6856	G	0.78	0.51
6857	G	1.146	0.65
6858	U	0.454	0.69
6859	G	0.482	0.66
6860	G	2.285	0.65
6861	U	0.258	0.51
6862	G	0.522	0.34
6863	G	0.298	0.3
6864	U	0.203	0.2
6865	C	0.095	0.12
6866	U	0.115	0.18
6867	C	0.115	0.44
6868	U	0.339	0.47
6869	U	0.359	0.53
6870	C	0.339	0.55
6871	A	0.665	0.4
6872	U	0.4	0.52
6873	G	1.214	0.46
6874	C	0.163	0.24
6875	A	0.855	0.6
6876	C	0.129	0.84
6877	A	2.157	0.92

6878	A	2.055	0.63
6879	G	0.834	0.28
6880	G	0.475	0.22
6881	A	0.285	0.53
6882	U	0.142	0.83
6883	G	0.149	0.82
6884	A	0.271	0.23
6885	U	0.244	0.81
6886	G	0.434	0.9
6887	G	0.326	0.64
6888	A	0.19	0.57
6889	G	0.298	0.06
6890	A	0.543	0.04
6891	C	0.014	0.17
6892	A	0.285	0.18
6893	C	0.149	0.21
6894	A	1.072	0.2
6895	G	0.732	0.07
6896	A	1.072	0.08
6897	C	0.326	0.1
6898	U	0.597	0.12
6899	U	0.326	0.69
6900	C	0.339	0.67
6901	U	0.366	0.53
6902	A	0.298	0.55
6903	C	0.054	0.93
6904	U	0.339	0.99
6905	U	0.543	0.95
6906	G	0.407	0.75
6907	G	0.461	0.77
6908	U	0.258	0.84
6909	U	0.258	0.59
6910	U	0.353	0.5
6911	G	0.461	0.37
6912	G	0.61	0.51
6913	C	0.068	0.48
6914	U	0.312	0.66
6915	U	0.624	0.58
6916	U	0.855	0.26
6917	A	0.705	0.14
6918	A	0.529	0.34
6919	U	0.326	0.59
6920	G	0.448	0.53

6921	G	0.298	0.17
6922	A	0.393	0.16
6923	A	0.393	0.12
6924	C	0.163	0.08
6925	U	0.692	0.11
6926	A	1.004	0.17
6927	G	0.773	0.26
6928	A	0.529	0.37
6929	G	0.285	0.44
6930	C	0	0.7
6931	A	0.692	0.51
6932	G	0.407	0.26
6933	A	0.746	0.26
6934	A	0.529	0.23
6935	A	0.719	0.27
6936	A	0.624	0.47
6937	U	0.109	0.54
6938	A	0.651	0.55
6939	G	0.732	0.32
6940	A	0.76	0.35
6941	A	0.488	0.38
6942	C	0.231	0.36
6943	U	0.678	0.34
6944	U	0.231	0.38
6945	A	1.221	0.19
6946	U	0	0.48
6947	A	0.61	0.42
6948	U	0.19	0.6
6949	U	0.76	0.55
6950	U	0.624	0.86
6951	A	0.339	0.9
6952	C	0.136	0.93
6953	U	0.149	0.95
6954	G	0.041	0.93
6955	G	0.041	0.85
6956	C	0	0.43
6957	A	0.136	0.57
6958	U	0.081	0.82
6959	G	0.122	0.67
6960	G	0.095	0.42
6961	U	0.054	0.34
6962	A	0	0.41
6963	G	0.163	0.5

6964	G	0.576	0.43
6965	G	0.488	0.35
6966	A	0.461	0.34
6967	U	0.197	0.65
6968	A	1.207	0.69
6969	A	0.888	0.75
6970	U	0.637	0.84
6971	A	1.743	0.73
6972	G	0.617	0.63
6973	G	0.305	0.58
6974	A	0.156	0.57
6975	C	0	0.58
6976	U	0.088	0.65
6977	A	0.353	0.7
6978	U	0.285	0.71
6979	A	1.18	0.62
6980	A	0.624	0.71
6981	U	0.488	0.71
6982	U	1.173	0.58
6983	A	0.692	0.61
6984	G	0.488	0.6
6985	U	0.115	0.65
6986	U	0.427	0.98
6987	U	1.173	0.98
6988	A	1.37	0.92
6989	A	0.841	0.51
6990	A	1.16	0.58
6991	U	0.543	0.56
6992	A	1.546	0.48
6993	A	0.753	0.39
6994	G	1.139	0.51
6995	U	0.332	0.77
6996	A	0.644	0.82
6997	U	0.136	0.65
6998	U	0.081	0.82
6999	A	0.563	0.58
7000	U	0.393	0.72
7001	A	0.922	0.3
7002	A	0.637	0.29
7003	U	0.115	0.26
7004	C	0.122	0.36
7005	U	0.434	0.42
7006	A	1.017	0.23

7007	A	0.726	0.37
7008	C	0	0.39
7009	A	0.556	0.47
7010	A	0.841	0.82
7011	U	0.454	0.86
7012	G	1.112	0.86
7013	A	1.139	0.55
7014	A	1.092	0.46
7015	A	1.207	0.48
7016	U	0.346	0.78
7017	G	0.57	0.72
7018	U	0.007	0.81
7019	A	0.834	0.82
7020	G	0.61	0.84
7021	A	0.807	0.77
7022	A	0.949	0.78
7023	G	0.563	0.8
7024	A	0.488	0.8
7025	C	0.034	0.81
7026	C	0.061	0.89
7027	A	0.563	0.87
7028	G	0.427	0.87
7029	G	0.692	0.86
7030	A	0.678	0.86
7031	A	0.61	0.86
7032	A	0.902	0.88
7033	U	0.753	0.88
7034	A	1.492	0.88
7035	A	1.119	0.87
7036	G	0.821	0.87
7037	A	0.963	0.89
7038	C	0.346	0.9
7039	A	0.793	0.93
7040	G	0.685	0.92
7041	U	0.332	0.92
7042	U	0.448	0.92
7043	U	0.597	0.92
7044	U	0.624	0.93
7045	A	0.746	0.91
7046	C	0.095	0.89
7047	C	0.292	0.87
7048	A	0.604	0.9
7049	G	0.264	0.91

7050	U	0.203	0.9
7051	C	0.149	0.74
7052	A	0.42	0.75
7053	C	0.061	0.78
7054	C	0.081	0.49
7055	A	0.393	0.1
7056	U	0.624	0.15
7057	U	0.644	0.33
7058	A	1.533	0.68
7059	U	0.488	0.85
7060	G	0.393	0.87
7061	U	0.149	0.83
7062	C	0.095	0.38
7063	U	0.271	0.46
7064	G	0.719	0.87
7065	G	0.332	0.87
7066	A	0.441	0.94
7067	U	0.305	0.98
7068	U	0.42	0.98
7069	G	0.217	0.98
7070	G	0.115	0.98
7071	U	0.041	0.99
7072	U	0.305	0.02
7073	U	0.766	0.97
7074	U	0.482	0.98
7075	C	0.109	0.98
7076	C	0.109	0.99
7077	A	0.549	0.99
7078	C	0.122	0.99
7079	U	0.624	0.99
7080	C	0.353	0.09
7081	A	0.78	0.08
7082	C	0.224	0.08
7083	A	0.42	0.24
7084	A	0.353	0.24
7085	C	0	0.09
7086	C	0.149	0.1
7087	A	0.305	0.58
7088	A	0.387	0.57
7089	U	0	0.18
7090	C	0.441	0.03
7091	A	1.757	0.1
7092	A	1.533	0.1

7093	U	0.726	0.04
7094	G	0.875	0.06
7095	A	0.78	0.12
7096	U	0.78	0.16
7097	A	0.936	0.35
7098	G	0.454	0.6
7099	G	0.02	0.69
7100	C	0	0.63
7101	C	0.061	0.62
7102	A	0.637	0.18
7103	A	1.16	0.18
7104	A	1.024	0.36
7105	G	0.766	0.76
7106	C	0.251	0.81
7107	A	1.01	0.26
7108	G	0.258	0.09
7109	G	0.312	0.1
7110	C	0.142	0.09
7111	A	1.519	0.03
7112	U	1.004	0.1
7113	G	0.495	0.14
7114	G	0.237	0.56
7115	U	0.047	0.68
7116	G	0.041	0.26
7117	U	0.061	0.33
7118	U	0.156	0.35
7119	G	0.034	0.58
7120	G	0.034	0.7
7121	U	0.237	0.7
7122	U	0.38	0.5
7123	U	0.359	0.12
7124	G	0.068	0.02
7125	G	0.149	0.04
7126	A	0.176	0.94
7127	G	0.176	0.94
7128	G	0.271	0.09
7129	A	0.373	0.07
7130	A	0.4	0.01
7131	A	1.35	0.05
7132	A	1.085	0.99
7133	U	0.258	0.99
7134	G	0.387	0.09
7135	G	0.4	0.08

7136	A	0.916	0.03
7137	A	1.119	0
7138	G	0.855	0.01
7139	G	0.8	0.01
7140	A	0.773	0.01
7141	U	0.244	0.02
7142	G	0.278	0.48
7143	C	1.506	0.97
7144	A	0.665	0.92
7145	A	1.017	0.03
7146	U	1.119	0.02
7147	A	1.831	0.01
7148	A	2.516	0.01
7149	A	0.671	0
7150	A	0.359	0.33
7151	G	0.17	0.34
7152	A	0.136	0.03
7153	G	0.061	0.65
7154	G	0.258	0.7
7155	U	0.014	0.53
7156	G	0.292	0
7157	A	0.326	0
7158	A	0.353	0
7159	G	0.312	0
7160	C	0.081	0
7161	A	0.041	0.16
7162	G	0.027	0.16
7163	A	0.068	0.07
7164	C	0.041	0.08
7165	C	0.081	0.07
7166	A	0.285	0.01
7167	U	0.027	0.18
7168	U	0.054	0.17
7169	G	0.027	0.01
7170	U	0.014	0.02
7171	C	0.149	0.16
7172	A	0.149	0.16
7173	A	0.244	0.18
7174	A	0.041	0.65
7175	C	0.163	0.6
7176	A	0.122	0.11
7177	U	0.054	0.2
7178	C	0	0.32

7179	C	0.041	0.65
7180	C	0.285	0.39
7181	A	0.054	0.01
7182	G	0	0.1
7183	G	0.8	0.13
7184	U	0.231	0.12
7185	A	0.922	0.01
7186	U	0.339	0.01
7187	A	0.719	0.01
7188	C	0.109	0.01
7189	U	0.393	0.05
7190	G	0.895	0.73
7191	G	0.692	0.75
7192	A	0.665	0.11
7193	A	0.61	0.02
7194	C	0.054	0.02
7195	U	0.76	0.02
7196	A	1.099	0.03
7197	A	0.651	0.64
7198	C	0	0.73
7199	A	0.393	0.1
7200	A	0.407	0
7201	U	0.163	0
7202	A	0.42	0
7203	C	0.081	0
7204	U	0.298	3.85E-05
7205	G	0.244	5.39E-06
7206	A	0.556	0
7207	U	0.57	0.01
7208	A	1.736	0.44
7209	A	1.234	0.83
7210	A	1.085	0.91
7211	A	0.922	0.15
7212	U	0.556	0
7213	C	0.475	0
7214	A	0.746	0.01
7215	A	0.909	0
7216	U	0.936	0
7217	U	0.922	0.94
7218	U	0.312	0.98
7219	G	0.244	0.4
7220	A	0.339	0
7221	C	0.109	3.80E-05

7222	G	0.109	1.04E-05
7223	G	0.054	5.21E-05
7224	C	0.027	0
7225	U	0.014	0.05
7226	C	0.014	0.14
7227	C	0.041	0.11
7228	U	0.231	0.01
7229	G	0.922	0.01
7230	G	0.882	0
7231	A	1.031	0
7232	G	0.366	0.15
7233	G	0.8	0.18
7234	A	0.732	0.14
7235	G	0.814	0.88
7236	G	0.841	0.84
7237	A	0.827	0.05
7238	G	1.058	0
7239	A	1.397	0.02
7240	U	0.285	0.03
7241	C	0.095	0.04
7242	C	0.068	0.03
7243	G	0.244	0.08
7244	G	0.407	0.85
7245	A	0.8	0.83
7246	A	0.787	0.06
7247	G	0.475	0.03
7248	U	0.529	0.03
7249	U	0.624	0
7250	A	0.99	0
7251	C	0.041	0
7252	C	0.014	0.75
7253	U	0.068	0.82
7254	U	0.231	0.33
7255	C	0.339	0.31
7256	A	0.448	0.31
7257	U	0.203	0.28
7258	G	0.271	0.57
7259	U	0.095	0.55
7260	G	0.149	0.27
7261	G	0.217	0.22
7262	A	0.705	0.15
7263	C	1.228	0.14
7264	A	1.017	0.23

7265	A	0.712	0.25
7266	A	0.726	0.2
7267	U	0.515	0.25
7268	U	0.346	0.61
7269	G	0.244	0.64
7270	C	0.387	0.62
7271	A	0.787	0.85
7272	G	0.99	0.81
7273	A	1.139	0.87
7274	G	1.35	0.92
7275	G	0.556	0.91
7276	A	0.509	0.85
7277	G	1.316	0.06
7278	A	1.051	0.02
7279	G	0.488	0.06
7280	U	0.597	0.06
7281	U	0.156	0.8
7282	C	0.041	0.88
7283	C	0.075	0.92
7284	U	0.061	0.96
7285	C	0.081	0.67
7286	U	0.339	0.91
7287	A	0.454	0.76
7288	C	0.156	0.4
7289	U	0.156	0.63
7290	G	0.454	0.73
7291	U	0.305	0.7
7292	A	0.943	0.67
7293	A	0.807	0.37
7294	A	0.821	0.48
7295	A	0.861	0.57
7296	U	0.882	0.59
7297	G	1.336	0.55
7298	A	1.126	0.59
7299	A	1.282	0.58
7300	U	0.366	0.57
7301	U	0.034	0.69
7302	G	0.102	0.72
7303	G	0.041	0.79
7304	U	0.102	0.81
7305	U	0.115	0.84
7306	U	0.054	0.6
7307	C	0.631	0.56

7308	U	1.35	0.11
7309	A	1.076	0.07
7310	A	0.983	0.39
7311	A	1.038	0.39
7312	U	0.59	0.35
7313	U	0.373	0.37
7314	G	0.068	0.8
7315	G	0.102	0.72
7316	G	0.258	0.89
7317	U	0.115	0.78
7318	A	0.176	0.82
7319	G	0.285	0.81
7320	A	0.685	0.76
7321	A	0.57	0
7322	G	0.522	0
7323	A	0.787	0
7324	U	0.658	0
7325	A	0.624	0
7326	G	0.393	0
7327	G	0.8	3.36E-05
7328	A	0.766	2.88E-05
7329	A	0.821	2.20E-05
7330	U	0.563	1.00E-06
7331	A	0.597	0
7332	C	0.298	0
7333	A	0.637	0
7334	G	0.556	0
7335	C	0.034	0
7336	U	0.251	4.90E-05
7337	A	0.292	0
7338	A	0.197	0.01
7339	C	0.081	0.64
7340	C	0.855	0.85
7341	A	0.251	0.39
7342	G	0.353	0.05
7343	A	0.482	0.04
7344	A	0.292	0.05
7345	G	0.068	0.4
7346	C	0.068	0.41
7347	C	0.129	0.13
7348	A	0.258	0.04
7349	A	0.38	0.07
7350	A	0.827	0.23

7351	G	0.712	0.3
7352	G	0.827	0.34
7353	A	0.821	0.32
7354	A	0.597	0.04
7355	C	0.631	0.07
7356	A	0.766	0.17
7357	G	0.448	0.3
7358	C	0.502	0.29
7359	A	1.343	0.22
7360	U	0.909	0.24
7361	A	1.472	0.2
7362	A	0.916	0.19
7363	A	1.092	0.19
7364	A	1.078	0.19
7365	G	0.868	0.62
7366	G	0.916	0.64
7367	A	0.909	0.89
7368	A	0.78	0.84
7369	U	0.549	0.87
7370	U	1.255	0.89
7371	A	1.16	0.65
7372	C	0.536	0.97
7373	G	0.359	0.98
7374	U	0.149	0.94
7375	G	0.068	0.86
7376	C	0.02	0.88
7377	C	0.156	0.88
7378	A	0.4	0.89
7379	U	0.203	0.99
7380	G	0.095	0.9
7381	U	0.122	0.14
7382	C	0.244	0.14
7383	A	1.051	0.41
7384	U	0.678	0.42
7385	A	0.888	0.48
7386	U	0.454	0.5
7387	U	0.97	0.12
7388	A	1.166	0.1
7389	G	0.678	0.04
7390	A	0.983	0.05
7391	C	0.515	0.04
7392	A	0.922	0.04
7393	A	0.787	0.02

7394	A	2.285	0.29
7395	U	0.712	0.38
7396	A	0.753	0.19
7397	A	0.821	0.45
7398	U	0.604	0.52
7399	C	1.16	0.11
7400	A	0.753	0.05
7401	A	0.963	0.01
7402	C	0.827	0.63
7403	A	0.502	0.63
7404	C	0.237	0.13
7405	U	0.237	0.17
7406	U	0.237	0.84
7407	G	0.285	0.93
7408	G	0.278	0.93
7409	C	0.373	0.89
7410	A	0.522	0.92
7411	U	0.726	0.82
7412	A	1.811	0.82
7413	A	1.682	0.57
7414	A	1.309	0.81
7415	G	1.112	0.81
7416	U	1.662	0.84
7417	A	0.882	0.76
7418	G	0.332	0.17
7419	G	0.454	0.15
7420	C	0.57	0.02
7421	A	0.841	0.06
7422	A	0.678	0.1
7423	A	0.685	0.93
7424	A	1.207	0.94
7425	A	1.499	0.85
7426	U	1.112	0.72
7427	G	0.787	0
7428	U	0.482	0
7429	U	0.59	0
7430	U	1.017	0.02
7431	A	0.949	0.73
7432	U	0.251	0.92
7433	U	0.237	0.99
7434	U	0.285	0.93
7435	G	0.217	0.01
7436	C	0.075	0.12

7437	C	0.156	0.17
7438	U	0.156	0.15
7439	C	0.712	0.22
7440	C	0.597	0.21
7441	A	1.166	0.14
7442	A	0.651	0.12
7443	G	0.888	0.05
7444	A	1.716	0.02
7445	G	0.712	0.06
7446	A	0.373	0.05
7447	G	0.488	0.29
7448	G	0.312	0.45
7449	G	0.448	0.21
7450	A	0.271	0.08
7451	G	0.271	0.07
7452	A	0.095	0.02
7453	C	0.027	0.02
7454	C	0.19	0.36
7455	U	0.57	0.44
7456	C	0.746	0.12
7457	A	0.326	0.01
7458	C	0.353	0.03
7459	G	0.231	0.66
7460	U	0.42	0.95
7461	G	0.76	0.88
7462	U	0.827	0.04
7463	A	0.543	0.01
7464	A	0.326	0.02
7465	C	0.163	0.02
7466	U	0.136	0.02
7467	C	0.027	0.09
7468	C	0.136	0.11
7469	A	0.231	0.06
7470	C	1.139	0.82
7471	A	1.072	0.92
7472	G	0.543	0.78
7473	U	0.597	0.17
7474	G	1.695	0.15
7475	A	0.041	0.13
7476	C	0.163	0
7477	C	0.217	0.01
7478	A	0.136	0.02
7479	G	0.054	0.15

7480	U	0.109	0.16
7481	C	0.312	0.15
7482	U	0.136	0.16
7483	C	0.488	0.15
7484	A	0.434	0.21
7485	U	1.289	0.21
7486	A	0.732	0.29
7487	G	0.529	0.28
7488	C	0.922	0.14
7489	A	1.37	0.01
7490	A	1.058	0.02
7491	A	0.678	0.01
7492	C	1.085	0.04
7493	A	0.787	0.1
7494	U	1.356	0.08
7495	A	1.085	0.02
7496	G	0.624	0.02
7497	A	0.217	0.02
7498	U	0.285	0.01
7499	U	0.203	1.10E-05
7500	G	0.149	0
7501	G	0.787	0
7502	A	0.692	0
7503	U	0.597	0
7504	U	0.882	0
7505	G	1.139	0
7506	A	0.434	9.76E-07
7507	U	0.814	9.71E-07
7508	G	0.787	3.50E-06
7509	G	1.37	0
7510	A	0.949	0
7511	A	0.692	0
7512	A	0.258	0
7513	C	0.353	0
7514	C	0.719	0.01
7515	A	0.963	0.45
7516	A	0.814	0.92
7517	A	0.285	0.84
7518	C	0.583	0.2
7519	U	0.936	0.19
7520	A	1.655	0.17
7521	A	1.207	0.03
7522	U	1.865	0.07

7523	A	0.753	0.19
7524	U	0.977	0.18
7525	C	0.427	0.66
7526	A	0.142	0.73
7527	C	0.068	0.42
7528	C	0.061	0.36
7529	A	0.102	0.44
7530	U	0.203	0.74
7531	G	1.695	0.66
7532	A	0.054	0.53
7533	G	0.237	0.87
7534	U	0.176	0.91
7535	G	0.095	0.79
7536	C	0.115	0.92
7537	A	0.19	0.9
7538	G	0.142	0.7
7539	A	0.136	0.18
7540	G	0.027	0.26
7541	G	0.271	0.72
7542	U	0.095	0.88
7543	G	0.095	0.84
7544	G	0.305	0.72
7545	C	0.095	0.8
7546	A	0.176	0.8
7547	G	0.61	0.74
7548	A	0.529	0.58
7549	A	0.203	0.48
7550	C	0.19	0.76
7551	U	0.414	0.75
7552	G	0.529	0.47
7553	U	0.943	0.54
7554	A	0.617	0.48
7555	U	0.441	0.31
7556	C	0.59	0.31
7557	G	0.427	0.42
7558	A	0.312	0.41
7559	U	0.319	0.59
7560	U	0.631	0.71
7561	G	0.719	0.73
7562	G	0.719	0.58
7563	A	0.509	0.46
7564	A	0.543	0.57
7565	U	0.414	0.78

7566	U	0.522	0.83
7567	G	0.468	0.81
7568	G	0.746	0.68
7569	G	0.719	0.7
7570	A	0.502	0.71
7571	G	0.448	0.65
7572	A	0.366	0.6
7573	U	0.827	0.62
7574	U	1.004	0.65
7575	A	0.631	0.64
7576	U	1.431	0.65
7577	A	1.004	0.64
7578	A	0.685	0.88
7579	A	0.536	0.89
7580	U	0.76	0.92
7581	U	1.207	0.9
7582	A	0.699	0.56
7583	G	0.509	0.35
7584	U	1.099	0.69
7585	A	1.092	0.49
7586	G	1.831	0.82
7587	A	0.658	0.26
7588	G	0.258	0.42
7589	A	0.387	0.29
7590	U	0.292	0.52
7591	C	0.224	0.48
7592	A	0.387	0.43
7593	C	0.149	0.34
7594	U	0.407	0.42
7595	C	0.258	0.64
7596	C	0.4	0.74
7597	A	0.461	0.85
7598	A	0.353	0.89
7599	U	0.529	0.95
7600	U	1.234	0.96
7601	G	0.163	0.81
7602	G	0.217	0.73
7603	C	0.149	0.77
7604	U	0.583	0.8
7605	U	1.112	0.69
7606	G	1.546	0.72
7607	G	0.136	0.73
7608	C	0.122	0.77

7609	C	0.122	0.78
7610	C	0.136	0.77
7611	C	0.136	0.7
7612	C	0.19	0.66
7613	A	0.461	0.59
7614	C	0.841	0.78
7615	A	0.665	0.8
7616	G	0.597	0.75
7617	A	0.719	0.74
7618	U	0.936	0.99
7619	G	1.072	0.98
7620	U	1.383	0.32
7621	G	0.773	0.13
7622	A	0.8	0.47
7623	A	1.078	0.47
7624	G	0.156	0.02
7625	A	0.197	0.27
7626	G	0.129	0.27
7627	G	0.095	0.02
7628	U	0.156	0.6
7629	A	0.17	0.61
7630	C	0.115	0.82
7631	A	0.068	0.8
7632	C	0.136	0.11
7633	U	0.332	0.67
7634	A	0.895	0.77
7635	C	0.244	0.93
7636	U	0.495	0.91
7637	G	0.387	0.7
7638	G	0.244	0.7
7639	U	0.122	0.74
7640	G	0.068	0.75
7641	G	0.095	0.73
7642	C	0.217	0.72
7643	A	0.122	0.71
7644	C	0.244	0.71
7645	C	0.19	0.73
7646	U	0.109	0.73
7647	C	0.122	0.73
7648	A	0.651	0.88
7649	A	0.705	0.94
7650	G	0.57	0.9
7651	A	0.617	0.33

7652	A	0.746	0.27
7653	A	1.112	0.58
7654	U	1.397	0.68
7655	A	1.051	0.56
7656	A	0.746	0.25
7657	A	0.726	0.19
7658	A	0.773	0.16
7659	G	0.529	0.2
7660	A	0.977	0.65
7661	G	0.115	0.76
7662	G	0.115	0.8
7663	G	0.17	0.88
7664	G	1.363	0.86
7665	U	0.203	0.86
7666	C	0.319	0.76
7667	U	1.16	0.75
7668	U	0.929	0.87
7669	U	0.387	0.92
7670	G	0.393	0.98
7671	U	0.326	0.99
7672	G	0.203	0.99
7673	C	0.115	0.94
7674	U	0.76	0.79
7675	A	1.275	0.77
7676	G	0.943	0.73
7677	G	0.19	0.86
7678	G	0.197	0.87
7679	U	0.109	0.94
7680	U	0.129	0.91
7681	C	0.136	0.9
7682	U	0.149	0.96
7683	U	1.044	0.97
7684	G	0.454	0.95
7685	G	0.176	0.97
7686	G	0.244	0.91
7687	U	0.102	0.69
7688	U	0.556	0.96
7689	U	0.285	0.99
7690	U	0.149	0.99
7691	C	0.19	1
7692	U	0.109	0.98
7693	C	0.305	0.82
7694	G	0.19	0.56

7695	C	1.343	0.1
7696	A	1.112	0.14
7697	A	0.665	0.74
7698	C	0.305	0.81
7699	G	0.644	1
7700	G	0.163	1
7701	C	0.251	1
7702	A	0.176	0.99
7703	G	0.197	0.87
7704	G	0.841	0.11
7705	U	0.522	0.11
7706	U	0.475	0.1
7707	C	0.183	0.95
7708	U	0.136	0.99
7709	G	0.414	0.99
7710	C	0.359	0.99
7711	A	0.821	0.9
7712	A	1.519	0.08
7713	U	0.536	0.85
7714	G	0.109	0.96
7715	G	0.129	0.95
7716	G	0.231	0.95
7717	C	0.285	0.83
7718	G	0.102	0.84
7719	C	0.081	0.98
7720	G	0.176	0.98
7721	G	0.264	0.97
7722	C	0.414	0.99
7723	G	0.793	0.98
7724	U	0.217	0.9
7725	C	1.519	0
7726	G	0.326	9.40E-05
7727	U	0.041	0
7728	U	0.868	0
7729	G	0.19	0
7730	A	0.041	0.9
7731	C	0.081	0.98
7732	G	0	0.99
7733	C	0.136	0.97
7734	U	0.109	0.98
7735	G	0.19	0.99
7736	A	1.207	0.84
7737	C	0.475	0.06

7738	C	0.19	0.06
7739	G	0.149	0.83
7740	C	0.081	0.92
7741	U	0.163	0.93
7742	C	0.231	0.93
7743	A	0.719	0.84
7744	G	0.217	0.21
7745	U	0.353	0.87
7746	C	0.149	0.91
7747	C	0.109	0.91
7748	C	0.041	0.91
7749	G	0.637	0.85
7750	A	0.285	0.8
7751	A	0.217	0.39
7752	C	0.068	0.34
7753	U	0.61	0.11
7754	U	1.519	0.03
7755	U	0.529	0.05
7756	A	0.57	0.06
7757	U	1.153	0.18
7758	U	0.353	0.14
7759	G	0.326	0.31
7760	G	0.122	0.32
7761	C	0.081	0.74
7762	U	0.231	0.85
7763	G	0.217	0.89
7764	G	0.244	0.9
7765	G	0.176	0.91
7766	A	0.407	0.87
7767	U	0.461	0.19
7768	A	2.536	0.08
7769	G	1.397	0.07
7770	U	0.434	0.89
7771	G	0.149	0.99
7772	C	0.244	0.99
7773	A	0.529	0.98
7774	G	0.475	0.92
7775	C	0.081	0.87
7776	A	0.136	0.98
7777	A	0.163	1
7778	C	0.109	1
7779	A	0.122	1
7780	G	0.109	1

7781	C	0.122	0.97
7782	A	0.61	0
7783	A	1.37	0
7784	C	0.732	0
7785	A	1.099	0
7786	G	0.081	0.97
7787	C	0.122	1
7788	U	0.109	1
7789	G	0.041	1
7790	U	0.258	1
7791	U	0.109	0.98
7792	G	0.136	0.86
7793	G	0.922	0.58
7794	A	0.665	0.64
7795	C	0	0.58
7796	G	1.668	0.13
7797	U	0.814	0.1
7798	G	0.163	0.48
7799	G	0.203	0.79
7800	U	0.298	0.97
7801	C	0.285	0.96
7802	A	0.407	0.46
7803	A	0.475	0.01
7804	G	0.407	0.01
7805	A	0.488	0.1
7806	G	0.556	0.28
7807	A	1.275	0.19
7808	C	0.041	0.97
7809	A	0.217	0.98
7810	A	0.231	0.99
7811	C	0.19	0.99
7812	A	0.556	0.87
7813	A	0.977	0
7814	G	1.492	0
7815	A	1.668	0
7816	A	1.655	0
7817	U	1.004	0
7818	U	0.448	0.87
7819	G	0.149	0.99
7820	U	0.149	1
7821	U	0.637	0.99
7822	G	0.149	0.93
7823	C	0.203	0.07

7824	G	0.38	0.27
7825	A	0.38	0.25
7826	C	0	0.34
7827	U	0.163	0.89
7828	G	0.163	0.93
7829	A	0.353	0.92
7830	C	0.136	0.87
7831	C	2.048	0.48
7832	G	0.109	0.88
7833	U	0.027	0.98
7834	C	0.298	1
7835	U	0.217	0.99
7836	G	0.095	0.99
7837	G	0.081	0.98
7838	G	0.068	0.95
7839	G	0.203	0.91
7840	A	1.058	0.02
7841	A	0.787	0
7842	C	0.556	0
7843	A	1.424	0
7844	A	2.007	7.80E-05
7845	A	1.139	8.50E-05
7846	G	0.868	5.70E-05
7847	A	0.922	0
7848	A	0.529	0.01
7849	C	0.339	0.59
7850	C	0.909	0.92
7851	U	0.176	0.39
7852	C	0.054	0.97
7853	C	0	0.99
7854	A	0.787	0.99
7855	G	1.465	0.99
7856	A	0.8	0.97
7857	C	0.434	0.84
7858	U	1.322	0.01
7859	A	3.35	0.02
7860	G	1.228	0.03
7861	G	1.295	0.08
7862	G	0.129	0.81
7863	U	0.041	0.93
7864	C	0.414	0.91
7865	A	0.217	0.32
7866	C	0.102	0.88

7867	U	0.197	1
7868	G	0.054	1
7869	C	0.068	1
7870	C	0.142	1
7871	A	0.359	0.81
7872	U	0.393	0.74
7873	C	0.332	0.7
7874	G	0.346	0.92
7875	A	0.319	0.98
7876	G	0.292	1
7877	A	0.387	0.99
7878	A	0.407	0.99
7879	G	1.289	0.96
7880	U	0.387	0.37
7881	A	0.353	0.33
7882	C	0.122	0.91
7883	U	0.454	0.97
7884	U	0.739	0.95
7885	A	2.041	0.87
7886	A	0.637	0.82
7887	A	0.59	0.77
7888	G	0.556	0.89
7889	G	0.4	0.9
7890	A	0.271	0.93
7891	C	0.081	0.92
7892	C	0.068	0.86
7893	A	0.509	0.81
7894	G	0.339	0.82
7895	G	0.054	0.79
7896	C	0.244	0.99
7897	G	0.251	0.99
7898	C	0.264	0.98
7899	A	0.604	0.92
7900	G	0.509	0.86
7901	C	0.17	0.78
7902	U	0.414	0.91
7903	G	0.57	0.83
7904	A	0.59	0.68
7905	A	0.814	0.63
7906	U	0.631	0.63
7907	G	0.434	0.61
7908	C	0.122	0.84
7909	U	0.454	0.89

7910	U	0.563	0.89
7911	G	0.529	0.66
7912	G	0.339	0.6
7913	G	0.298	0.17
7914	G	0.434	0.22
7915	A	0.637	0.53
7916	U	0.42	0.74
7917	G	0.746	0.72
7918	U	0.461	0.44
7919	G	0.549	0.4
7920	C	0.366	0.26
7921	G	0.543	0.3
7922	U	0.359	0.57
7923	U	0.631	0.59
7924	U	1.038	0.62
7925	A	1.899	0.85
7926	G	1.397	0.85
7927	A	0.671	0.73
7928	C	0.665	0.43
7929	A	0.732	0.44
7930	A	0.556	0.39
7931	G	0.597	0.46
7932	U	0.081	0.82
7933	C	0.02	0.9
7934	U	0.176	0.94
7935	G	0.007	0.84
7936	C	0.081	0.83
7937	C	0.027	0.89
7938	A	0.109	0.93
7939	C	0.231	0.88
7940	A	1.146	0.87
7941	C	0	0.09
7942	U	0.197	0.02
7943	A	2.394	0.02
7944	C	0.163	0.65
7945	U	0.17	0.89
7946	G	0.061	0.91
7947	U	0.102	0.84
7948	A	0.054	0.74
7949	C	0.014	0.83
7950	C	0.095	0.62
7951	A	0.075	0.62
7952	U	0.136	0.81

7953	G	0.427	0.82
7954	G	1.648	0.65
7955	C	0.231	0.36
7956	C	0.529	0.5
7957	A	0.888	0.62
7958	A	0.793	0.67
7959	A	1.309	0.51
7960	U	0.807	0.42
7961	G	0.502	0.36
7962	C	0.454	0.34
7963	A	0.637	0.35
7964	A	0.909	0.38
7965	G	0.373	0.36
7966	U	0.285	0.05
7967	C	0.149	0.07
7968	U	0.8	0.07
7969	A	1.072	0.01
7970	A	0.909	0
7971	C	0.231	0
7972	A	0.583	0.02
7973	C	0.19	0.97
7974	C	0.746	0.98
7975	A	1.194	0.05
7976	A	1.058	0.01
7977	A	1.492	0.01
7978	G	0.529	0.03
7979	U	0.461	0.07
7980	G	0.434	0.52
7981	G	0.529	0.92
7982	A	0.922	0.49
7983	A	0.787	0.01
7984	C	0.76	0.03
7985	A	0.882	0.03
7986	A	0.475	0
7987	U	0.366	0.06
7988	G	0.312	0.06
7989	A	0.19	0.05
7990	G	0.054	0.03
7991	A	0.027	0
7992	C	0.109	0.05
7993	U	0.366	0.06
7994	U	0.543	0.1
7995	G	0.298	0.09

7996	G	0.271	0.05
7997	C	0.326	0.04
7998	A	0.8	0.04
7999	A	1.465	0
8000	G	1.587	0
8001	A	1.343	0
8002	G	0.339	0.03
8003	U	0.231	0.05
8004	G	0.231	0.04
8005	G	0.149	0.01
8006	G	0.149	0.01
8007	A	0.366	0
8008	G	0.136	1.28E-05
8009	C	0.176	4.48E-06
8010	G	0.353	7.98E-06
8011	A	0.732	5.13E-06
8012	A	0.76	5.51E-05
8013	A	0.488	5.81E-05
8014	G	0.231	5.65E-06
8015	G	0.136	4.52E-05
8016	U	0.122	0
8017	U	0.217	0
8018	G	0.461	0.01
8019	A	0.244	0.01
8020	C	0.041	0
8021	U	0.163	0.84
8022	U	0.054	0.86
8023	C	0.054	0.15
8024	U	0.176	0.24
8025	U	0.298	0.15
8026	G	0.339	0.05
8027	G	0.353	0.06
8028	A	0.529	0.08
8029	A	0.732	0.62
8030	G	0.637	0.6
8031	A	0.855	0.04
8032	A	0.678	0.03
8033	A	0.61	0.02
8034	A	0.773	0.01
8035	U	0.543	0.03
8036	A	1.343	0.06
8037	U	0.827	0.53
8038	A	1.044	0.54

8039	A	0.814	0.11
8040	C	0.841	0.11
8041	A	0.99	0.86
8042	G	0.366	0.84
8043	C	0.366	0
8044	C	0.027	2.57E-05
8045	C	0.041	6.23E-05
8046	U	0.041	0.8
8047	C	0.176	1
8048	C	2.509	0.97
8049	U	1.343	0.83
8050	A	1.044	0.07
8051	G	1.655	0.17
8052	A	0.149	0.11
8053	G	0.109	0
8054	G	0.176	0
8055	A	0.027	0.05
8056	G	0.136	0.08
8057	G	0.163	0.03
8058	C	0.773	0.06
8059	A	0.665	0.16
8060	C	1.099	0.11
8061	A	0.773	0.03
8062	A	0.556	0.75
8063	A	0.298	0.88
8064	U	0.271	1
8065	U	0.298	0.8
8066	C	0.42	0
8067	A	0.529	0
8068	A	0.393	0
8069	C	0.868	0.01
8070	A	1.017	0.01
8071	A	1.397	0.01
8072	G	1.18	0.01
8073	A	1.166	0.01
8074	G	1.248	0.01
8075	A	1.234	0.05
8076	A	1.275	0.07
8077	G	1.221	0.08
8078	A	1.004	0.07
8079	A	0.57	0.06
8080	C	1.017	0.07
8081	A	0.448	0.08

8082	U	0.57	0.56
8083	G	0.353	0.77
8084	U	1.017	0.79
8085	A	0.583	0.69
8086	U	1.329	0.03
8087	G	0.963	0.02
8088	A	0.841	0.03
8089	A	0.597	0.04
8090	U	0.651	0.14
8091	U	1.221	0.82
8092	A	0.719	0.83
8093	C	3.54	0.77
8094	A	1.221	0.59
8095	A	1.316	0.13
8096	A	1.194	0.23
8097	A	0.407	0.28
8098	G	0.502	0.35
8099	U	0.529	0.64
8100	U	1.424	0.82
8101	G	0.868	0.82
8102	A	0.488	0.7
8103	A	0.488	0.66
8104	U	1.017	0.27
8105	A	1.004	0.26
8106	G	0.054	0.13
8107	C	0.054	0.1
8108	U	0.054	0.12
8109	G	0.041	0.1
8110	G	0.054	0.06
8111	G	0.244	0.06
8112	A	0.217	0.13
8113	U	0.8	0.34
8114	G	0.353	0.46
8115	U	0.461	0.34
8116	G	0.298	0.17
8117	U	0.109	0.72
8118	U	0.081	0.86
8119	U	0.041	0.86
8120	G	0.027	0.79
8121	G	0.122	0.58
8122	C	0.095	0.25
8123	A	0.149	0.25
8124	A	0.448	0.23

8125	U	0.488	0.11
8126	U	0.068	0.26
8127	G	0.027	0.69
8128	G	0.041	0.7
8129	U	0.068	0.62
8130	U	0.122	0.46
8131	U	0.244	0.47
8132	G	1.411	0.32
8133	A	0.109	0.08
8134	C	0.068	0.11
8135	C	0.122	0.13
8136	U	0.176	0.11
8137	U	0.081	0.31
8138	G	0.027	0.63
8139	C	0.054	0.79
8140	U	0.054	0.77
8141	U	0.041	0.23
8142	C	0.231	0.24
8143	U	0.42	0.25
8144	U	0.855	0.26
8145	G	0.637	0.41
8146	G	1.004	0.42
8147	A	0.705	0.24
8148	U	1.031	0.24
8149	A	0.855	0.21
8150	A	0.8	0.21
8151	A	0.665	0.21
8152	G	0.366	0.26
8153	U	0.719	0.97
8154	A	0.326	0.96
8155	U	1.18	0.67
8156	A	0.448	0.51
8157	U	0.787	0.53
8158	A	0.515	0.22
8159	C	0.705	0.21
8160	A	0.787	0.05
8161	A	0.665	0.05
8162	U	1.18	0.1
8163	A	0.42	0.12
8164	U	0.787	0.23
8165	G	0.719	0.37
8166	G	0.502	0.38
8167	A	0.312	0.2

8168	G	0.298	0.1
8169	U	0.42	0.33
8170	U	0.57	0.42
8171	U	0.936	0.33
8172	A	0.475	0.39
8173	U	0.977	0.44
8174	A	0.624	0.64
8175	U	0.882	0.73
8176	A	0.488	0.7
8177	G	0.366	0.29
8178	U	0.285	0.38
8179	U	0.393	0.37
8180	G	0.353	0.12
8181	U	0.597	0.1
8182	A	0.231	0.92
8183	G	0.841	0.97
8184	G	0.624	0.96
8185	A	0.556	0.93
8186	G	0.393	0.98
8187	U	0.597	0.8
8188	A	0.787	0.89
8189	A	0.57	0.79
8190	U	0.326	0.54
8191	A	0.095	0.53
8192	C	0.149	0.44
8193	U	0.326	0.41
8194	G	0.271	0.28
8195	U	0.556	0.96
8196	U	1.058	0.95
8197	A	0.943	0.81
8198	A	0.956	0.5
8199	G	0.753	0.36
8200	A	0.76	0.19
8201	A	0.631	0.6
8202	U	0.678	0.87
8203	A	0.651	0.86
8204	G	0.326	0.44
8205	U	0.224	0.34
8206	G	0.142	0.25
8207	A	0.427	0.23
8208	U	0.326	0.76
8209	C	0.203	0.66
8210	U	1.166	0.66

8211	A	1.166	0.16
8212	U	0.461	0.07
8213	A	0.909	0.46
8214	U	0.027	0.66
8215	A	0.515	0.66
8216	G	0.637	0.57
8217	U	0.271	0.57
8218	A	0.922	0.56
8219	C	0.271	0.32
8220	A	0.936	0.2
8221	A	0.678	0.25
8222	A	1.004	0.34
8223	U	0.57	0.56
8224	G	0.258	0.55
8225	C	0.19	0.97
8226	U	0.312	0.96
8227	A	0.678	0.83
8228	G	0.312	0.28
8229	C	0.176	0.05
8230	U	0.42	0.11
8231	A	0.678	0.27
8232	A	0.814	0.22
8233	G	0.407	0.04
8234	U	0.298	0.51
8235	U	0.366	0.7
8236	A	0.746	0.87
8237	A	0.57	0.81
8238	G	0.393	0.7
8239	G	0.448	0.21
8240	C	0.176	0.24
8241	A	0.393	0.3
8242	G	0.271	0.27
8243	G	0.19	0.41
8244	G	0.122	0.51
8245	G	0.149	0.68
8246	U	0.163	0.64
8247	A	0.637	0.55
8248	U	0.475	0.66
8249	A	0.949	0.65
8250	G	0.353	0.49
8251	G	0.081	0.4
8252	C	0.027	0.25
8253	C	0.054	0.36

8254	A	0.298	0.43
8255	G	0.922	0.39
8256	U	0.095	0.42
8257	G	0.244	0.91
8258	U	0.298	0.94
8259	U	0.203	0.93
8260	C	0.244	0.93
8261	U	0.488	0.93
8262	C	0.217	0.25
8263	U	0.163	0.26
8264	U	0.231	0.34
8265	C	0.163	0.27
8266	C	0.109	0.26
8267	C	0.122	0.14
8268	C	0.109	0.11
8269	A	0.149	0.1
8270	C	0.176	0.13
8271	C	0.122	0.29
8272	C	0	0.44
8273	U	0.163	0.45
8274	C	0.176	0.27
8275	U	0.122	0.87
8276	U	0.624	0.97
8277	A	2.279	0.98
8278	U	0.366	0.98
8279	U	0.366	0.98
8280	U	0.258	0.29
8281	C	0.027	0.12
8282	C	0.353	0.11
8283	A	0.665	0.6
8284	G	0.393	0.6
8285	C	0.38	0.16
8286	A	0.637	0.24
8287	G	0.61	0.76
8288	A	0.732	0.61
8289	C	0.068	0.84
8290	C	0.122	0.44
8291	C	0.488	0.42
8292	A	1.587	0.4
8293	U	0.895	0.38
8294	A	1.112	0.54
8295	U	0.502	0.63
8296	C	0.285	0.63

8297	C	0.298	0.14
8298	A	0.76	0.1
8299	A	1.641	0.08
8300	C	0.502	0.42
8301	A	1.234	0.45
8302	G	0.203	0.51
8303	G	0.298	0.53
8304	A	0.298	0.44
8305	C	0.163	0.29
8306	C	0.217	0.41
8307	C	0.217	0.43
8308	G	0.393	0.89
8309	G	0.244	0.92
8310	C	0.298	0.48
8311	A	0.719	0.14
8312	C	0	0.19
8313	U	0.475	0.28
8314	G	0.217	0.53
8315	C	0.041	0.67
8316	C	0.163	0.67
8317	A	0.515	0.57
8318	A	1.614	0.56
8319	C	0.298	0.74
8320	C	0.231	0.76
8321	A	1.004	0.43
8322	G	1.397	0.28
8323	A	1.655	0.5
8324	G	1.126	0.47
8325	A	1.194	0.19
8326	A	1.397	0.17
8327	G	0.597	0.12
8328	G	0.393	0.08
8329	C	0.271	0.12
8330	A	1.234	0.08
8331	A	1.777	0.05
8332	A	1.126	0.06
8333	G	0.855	0.08
8334	A	1.004	0.1
8335	A	0.678	0.13
8336	A	0.963	0.17
8337	G	0.732	0.18
8338	A	0.434	0.29
8339	G	0.353	0.35

8340	A	0.746	0.38
8341	C	0.42	0.61
8342	G	0.19	0.71
8343	G	0.176	0.54
8344	U	0.231	0.62
8345	G	0.231	0.77
8346	G	0.393	0.31
8347	A	0.339	0.18
8348	G	0.502	0.03
8349	A	0.61	0.03
8350	A	0.692	0.05
8351	G	0	0.05
8352	G	0.217	0.04
8353	C	0.353	0.07
8354	G	0.461	0.09
8355	G	0.895	0.09
8356	U	0.285	0.06
8357	G	0.366	0.11
8358	G	0.231	0.09
8359	C	0.095	0.04
8360	A	0.99	0.03
8361	A	1.221	0.04
8362	C	0.651	0.49
8363	A	1.234	0.51
8364	G	0.488	0.73
8365	C	0.109	0.89
8366	U	0.081	0.92
8367	C	0.136	0.94
8368	C	0.136	0.88
8369	U	0.122	0.52
8370	G	0	0.51
8371	G	0.882	0.84
8372	C	0.109	0.47
8373	C	0.122	0.04
8374	U	0.475	0.43
8375	U	0.407	0.49
8376	G	0.339	0.5
8377	G	0.203	0.9
8378	C	0.095	0.81
8379	A	0.692	0.46
8380	G	0.773	0.49
8381	A	1.166	0.54
8382	U	1.017	0.59

8383	A	1.75	0.42
8384	G	1.275	0.39
8385	A	1.044	0.46
8386	A	1.397	0.99
8387	U	0.787	0.96
8388	A	2.116	0
8389	U	0.909	0
8390	A	1.885	0
8391	U	1.004	0
8392	U	1.221	0
8393	C	0.977	0.01
8394	A	1.099	0.96
8395	U	0.515	0.99
8396	U	0.366	0.47
8397	U	0.271	0.39
8398	C	0.217	0.14
8399	C	0.068	0.22
8400	U	0.448	0.27
8401	G	0.922	0.24
8402	A	0.746	0.12
8403	U	0.244	0.25
8404	C	0.136	0.23
8405	C	0.136	0.24
8406	G	0.298	0.1
8407	C	0.109	0.13
8408	C	0.244	0.17
8409	A	0.855	0.13
8410	A	1.072	0.01
8411	C	0.312	0
8412	U	0.719	0.01
8413	G	1.221	0.01
8414	A	1.289	0
8415	U	1.017	0
8416	A	1.017	0.01
8417	C	0.366	0.29
8418	G	0.461	0.3
8419	C	0.068	0.03
8420	C	0.081	0.5
8421	U	0.217	0.52
8422	C	0.258	0.07
8423	U	0.583	0.08
8424	U	0.936	0.03
8425	G	0.949	0.02

8426	A	0.8	0.01
8427	C	0.258	0.01
8428	U	0.475	0.29
8429	U	0.868	0.77
8430	G	0.475	0.53
8431	G	0.014	0.09
8432	C	0.434	0.12
8433	U	1.248	0.12
8434	A	0.787	0.01
8435	U	0.76	0.01
8436	U	0.678	0.01
8437	C	0.936	0.15
8438	A	0.746	0.26
8439	G	0.746	0.11
8440	C	1.126	0.02
8441	A	0.936	0.02
8442	A	0.57	0.01
8443	C	0.488	0.09
8444	U		0.29
8445	G	0.353	0.21
8446	C	0.732	0
8447	A	0.529	0.11
8448	G	0.448	0.13
8449	A	0.515	0.03
8450	A	0.312	0.01
8451	C	0.068	0.02
8452	C	0.068	0.82
8453	U	0.258	0.87
8454	U	0.488	0.78
8455	G	0.271	0.02
8456	C	0	0.03
8457	U	0.407	0.07
8458	A	1.153	0.04
8459	U	0.556	0.03
8460	C	0.529	0.03
8461	G	1.044	0
8462	A	1.221	0.75
8463	G	0.99	0.94
8464	A	0.556	0.9
8465	G	0.909	0.02
8466	U	0.61	0
8467	A	1.695	0
8468	U	0.868	0.07

8469	A		0.07
8470	C	1.004	0
8471	C	0	0
8472	A	0.353	0
8473	G	0.8	0.03
8474	A	0.773	0.09
8475	U	0	0.09
8476	C	0	0
8477	C	0.014	0
8478	U	0.109	0
8479	C	0.041	7.10E-05
8480	C	0.041	0
8481	A	0.271	0.16
8482	A	0.298	0.16
8483	C	0.081	0.03
8484	C	0.312	0
8485	A	0.339	0
8486	A	0.57	0
8487	U	0.502	0.02
8488	A	0.583	0.03
8489	C	0.068	0.05
8490	U	0.787	0.15
8491	C	0.502	0.14
8492	C	0.461	0.06
8493	A	0.99	0.07
8494	G	0.732	0.22
8495	A	0.8	0.22
8496	G	0.624	0.16
8497	G	0.176	0.2
8498	C	0.014	0.17
8499	U	0.095	0.14
8500	C	0	0.06
8501	U	0.014	0.06
8502	C	0	0.1
8503	U	1.343	0.15
8504	G	0.461	0.39
8505	C	0.203	0.37
8506	G	0.393	0.12
8507	A	0.353	0.06
8508	C	0.122	0.02
8509	C	0	0.02
8510	C	0.095	0.39
8511	U	0.787	0.4

8512	A	1.072	0.07
8513	C	2.048	0.06
8514	A	1.329	0.07
8515	G	0.224	0.15
8516	A	0.692	0.15
8517	G	0.176	0.05
8518	G	0.217	0.1
8519	A	0.332	0.13
8520	U	0.482	0.26
8521	U	0	0.26
8522	C	0.814	0.3
8523	G	1.194	0.39
8524	A	0.99	0.12
8525	G	0.732	0.01
8526	A	1.234	0
8527	A	0.502	0.01
8528	G	0.081	0.07
8529	U	0.041	0.09
8530	C	0.014	0.1
8531	C	0.176	0.21
8532	U	0.339	0.21
8533	C	0.407	0
8534	A	1.099	0.01
8535	G	0.637	0
8536	G	0.651	0
8537	A	0.556	0
8538	C	0.095	0
8539	U	0.38	0
8540	G	0.529	1.10E-05
8541	A	0.773	9.90E-07
8542	A	0.841	2.99E-06
8543	C	0.122	9.85E-06
8544	U	0.339	0.1
8545	G	0.38	0.95
8546	A	0.61	0.94
8547	C	0.176	0.09
8548	C	0.068	0.02
8549	U	0.434	0.05
8550	A	0.583	0.04
8551	C	0.122	0.15
8552	C	0.366	0.95
8553	U	0.543	0.92
8554	A	0.8	0.21

8555	C	0.285	0.23
8556	A	0.99	0.23
8557	A	0.949	0.22
8558	U	0.692	0.49
8559	A	1.37	0.5
8560	U	1.072	0.87
8561	G	0.57	0.84
8562	G	0.339	0.94
8563	G	0.231	0.87
8564	U	0.529	0.92
8565	G	0.366	0.88
8566	G	0.61	0.79
8567	A	0.868	0.67
8568	G	0.42	0.67
8569	C	0	0.68
8570	U	0.543	0.75
8571	A	0.529	0.75
8572	U	0.42	0.8
8573	U	0.326	0.8
8574	U	0.38	0.82
8575	C	0.149	0.97
8576	C	0	0.97
8577	A	1.112	0.83
8578	U	1.465	0.64
8579	G	2.279	0.61
8580	A	1.044	0.07
8581	G	0.081	0.06
8582	G	0.353	0.08
8583	C	0.176	0.11
8584	G	0.312	0.1
8585	G	0.312	0.31
8586	U	0.122	0.71
8587	C	0.57	0.84
8588	C	0.176	0.84
8589	A	1.017	0.79
8590	G	0.448	0.02
8591	G	0.353	0.02
8592	C	0.244	0.01
8593	C	0.366	0
8594	G	0.366	0
8595	U	0.217	0
8596	C	0.678	0
8597	U	0.515	0

8598	G	0.543	0
8599	G	0.61	0
8600	A	0.326	0.1
8601	G	0.407	0.1
8602	A	0.719	0.07
8603	U	0.312	0.02
8604	C	0.326	0.08
8605	U	0.936	0.08
8606	G	1.085	0
8607	C	0.366	0
8608	G	0.814	0.3
8609	A	0.882	0.31
8610	C		0.65
8611	A	1.506	0.96
8612	G	0.502	0.52
8613	A	0.977	0.02
8614	G	0.827	0.02
8615	A	1.533	0
8616	C	0.244	0
8617	U	0.556	0.43
8618	C	0.393	0.75
8619	U	0.76	0.9
8620	U	0.773	0.84
8621	G	0.855	0
8622	C	0.285	0
8623	G	0.271	8.23E-05
8624	G	0.19	4.86E-06
8625	G	0.353	9.24E-07
8626	C	0.963	1.60E-05
8627	G	0.637	1.60E-05
8628	C	0.271	5.85E-06
8629	G	0.475	1.63E-05
8630	U	0.597	2.92E-05
8631	G	0.326	2.10E-05
8632	G	0.081	1.91E-07
8633	G	0.271	0
8634	G	0.203	0
8635	A	0.963	2.59E-07
8636	G	1.478	2.39E-07
8637	A	0.99	2.26E-06
8638	C	0.285	0
8639	U	0.76	0.04
8640	U	0.99	0.04

8641	A	1.926	3.50E-06
8642	U	1.126	8.80E-06
8643	G	0.678	9.20E-06
8644	G	0.868	1.51E-05
8645	G	0.515	0.03
8646	A	0.719	0.04
8647	G	0.692	0.01
8648	A	0.787	0
8649	C	0.203	4.73E-06
8650	U	0.217	1.73E-06
8651	C	0.163	9.04E-07
8652	U	1.004	8.73E-07
8653	U	1.695	0
8654	A	1.845	0
8655	G	1.058	0
8656	G	1.085	2.83E-06
8657	A	0.719	2.08E-05
8658	G	1.017	0
8659	A	1.031	0
8660	G	0.678	0
8661	G	0.895	0
8662	U	0.61	0
8663	G	0.285	0.1
8664	G	0.556	0.14
8665	A	0.909	0.23
8666	A	1.044	0.25
8667	G	0.8	0.67
8668	A	1.383	0.61
8669	U	0.922	0.07
8670	G	0.895	0.04
8671	G	0.543	0.01
8672	A	1.438	0.14
8673	U	0.963	0.82
8674	A	1.058	0.85
8675	C	0.258	0.35
8676	U	0.583	0.27
8677	C	1.207	0.01
8678	G	0.76	0
8679	C	0	0
8680	A	0.529	0.05
8681	A	0.678	0.12
8682	U	0.312	0.12
8683	C	0.068	0.02

8684	C	0.054	0.01
8685	C	0.122	0.01
8686	C	0.339	0
8687	A	0.949	0
8688	G	0.515	0.01
8689	G	0.705	0.01
8690	A	1.044	0
8691	G	0.61	0
8692	G	0.637	0
8693	A	0.719	0.01
8694	U	0.665	0.28
8695	U	1.356	0.28
8696	A	2.184	0.19
8697	G	1.763	0.03
8698	A	1.221	0.05
8699	C	0	0.15
8700	A	1.085	0.23
8701	A	1.316	0.27
8702	G	0.746	0.26
8703	G	0.597	0.19
8704	G	0.298	0.1
8705	C	0.19	0.2
8706	U	1.275	0.31
8707	U	0.773	0.37
8708	G	1.316	0.37
8709	A	1.058	0.07
8710	G	0.488	0.01
8711	C	0.136	0.06
8712	U	0.258	0.23
8713	C	0.109	0.21
8714	A	0.448	0.17
8715	C	0.027	0.31
8716	U	0.19	0.44
8717	C	0.176	0.5
8718	U	0.095	0.63
8719	C	0.136	0.67
8720	U	1.072	0.32
8721	U	0.529	0.29
8722	G	0.977	0.22
8723	U	0.61	0.39
8724	G	0.963	0.77
8725	A	1.234	0.73
8726	G	0.583	0.36

8727	G	0.543	0.5
8728	G	0.8	0.48
8729	A	1.139	0.09
8730	C	0.149	0.33
8731	A	1.234	0.36
8732	G	1.302	0.27
8733	A	1.614	0.16
8734	A	1.302	0.03
8735	A	0.977	0.23
8736	U	0.719	0.52
8737	A	0.841	0.31
8738	C	0.136	0.12
8739	A	0.99	0.05
8740	A	1.153	0.01
8741	U	0.597	0.19
8742	C	0.203	0.31
8743	A	1.085	0.33
8744	G	0.665	0.26
8745	G	0.529	0.13
8746	G	0.502	0.15
8747	A	0.922	0.11
8748	C	0.176	0.09
8749	A	0.692	0.11
8750	G	0.665	0.07
8751	U	0.529	0.3
8752	A	1.031	0.3
8753	U	0.556	0.15
8754	A	2.089	0.16
8755	U	0.719	0.2
8756	G	1.506	0.17
8757	A	1.031	0.03
8758	A	1.275	0.02
8759	U	0.909	0.01
8760	A	0.773	0.01
8761	C	0.068	0.07
8762	U	0.041	0.08
8763	C	0.095	0.03
8764	C	0	0.15
8765	A	0.203	0.24
8766	U	0.258	0.78
8767	G	0.231	0.89
8768	G	0.285	0.77
8769	A	0.475	0.08

8770	G	0.502	0.07
8771	A	0.963	0.07
8772	A	0.76	0.02
8773	A	0.637	0.04
8774	C	0.149	0.07
8775	C	0.041	0.63
8776	C	0.109	0.63
8777	A	0.529	0.59
8778	G	0.448	0.07
8779	C	0.258	0.04
8780	U	0.461	0.03
8781	G	0.8	0
8782	A	0.502	0
8783	A	0.543	0
8784	G	0.38	0
8785	A	0.475	0
8786	G	0.637	0
8787	A	0.529	0
8788	G	0.624	0
8789	A	0.841	4.16E-05
8790	G	0.787	4.97E-05
8791	A	0.814	7.00E-05
8792	A	1.099	0
8793	A	0.787	0
8794	A	0.637	3.47E-05
8795	A	0.827	8.07E-06
8796	U	0.665	0
8797	U	0.651	0
8798	A	0.739	0.01
8799	G	0.448	0.02
8800	C	0.007	0.03
8801	A	0.515	0.24
8802	U	0.176	0.65
8803	A	0.685	0.47
8804	C	0	0.35
8805	A	0.692	0.33
8806	G	0.522	0.25
8807	A	0.61	0.08
8808	A	0.631	0.35
8809	A	0.346	0.35
8810	A	0.339	0.08
8811	C	0.373	0.32
8812	A	0.678	0.29

8813	A	0.488	0
8814	A	0.326	0
8815	A	0.909	0.12
8816	U	0.054	0.2
8817	A	0.353	0.11
8818	U	0.312	0.06
8819	G	0.488	0.03
8820	G	0.515	0.04
8821	A	0.719	0.24
8822	U	0.488	0.82
8823	G	0.556	0.61
8824	A	1.763	0.2
8825	U	1.37	0.48
8826	A	1.316	0.32
8827	U	0.393	0.66
8828	A	0.868	0.69
8829	G	0.556	0.09
8830	A	0.61	0.86
8831	U	0.502	0.88
8832	G	0.637	0.04
8833	A	0.583	0.08
8834	G	0.556	0.15
8835	U	0.339	0.13
8836	A	0.637	0.05
8837	A	0.637	0.06
8838	G	0.38	0.01
8839	A	0.57	0.66
8840	U	0.38	0.74
8841	G	0.339	0.09
8842	A	0.692	0.09
8843	U	0.461	0.67
8844	G	0.583	0.61
8845	A	0.637	0.02
8846	C	0.231	0.04
8847	U	0.366	0.35
8848	U	0.298	0.77
8849	G	0.339	0.5
8850	G	0.353	0.39
8851	U	0.231	0.43
8852	A	0.719	0.24
8853	G	1.072	0.02
8854	G	0.461	0.02
8855	G	0.339	0

8856	G	0.217	0.01
8857	U	0.136	0
8858	A	0.556	0
8859	U	0.366	0
8860	C	0.19	0.01
8861	A	0.76	1
8862	G	0.583	1
8863	U	0.353	0.71
8864	G	0.665	0.24
8865	A	0.597	0.14
8866	G	0.61	0.01
8867	G	0	0.02
8868	C	0	0.06
8869	C	0.054	0.59
8870	A	0.57	0.73
8871	A	0.461	0.59
8872	A	1.085	0.63
8873	A	0.746	0.81
8874	G	0.203	0.8
8875	U	0.176	0.29
8876	U	0	0.29
8877	C	0.054	0.28
8878	C	0.176	0.63
8879	C	0.217	0.68
8880	C	0.041	0.88
8881	U	0.42	0.89
8882	A	0.76	0.36
8883	A	0.895	0.02
8884	G	0.855	0.02
8885	A	1.044	0.02
8886	A	0.827	0.01
8887	C	0.38	0.32
8888	A	0.502	0.84
8889	A	0.665	0.87
8890	U	0.366	0.91
8891	G	0.773	0.8
8892	A	0.434	0.56
8893	G	0.624	0.79
8894	U	0.678	0.84
8895	U	0.746	0.12
8896	A	0.583	0.08
8897	C	0.298	0.08
8898	A	0.678	0.04

8899	A	0.814	0.03
8900	A	1.194	0.16
8901	U	0.773	0.24
8902	U	0.665	0.36
8903	G	0.448	0.25
8904	G	0.393	0.08
8905	C	0.312	0.09
8906	A	0.8	0.07
8907	A	1.18	0.19
8908	U	0.855	0.92
8909	A	1.411	0.82
8910	G	0.76	0.52
8911	A	0.515	0.2
8912	C	0.163	0.1
8913	A	0.271	0.54
8914	U	0.19	0.62
8915	G	0.054	0.66
8916	U	0.122	0.79
8917	C	0	0.74
8918	U	0.027	0.36
8919	C	0.285	0.43
8920	A	0	0.37
8921	U	0	0.66
8922	U	0.353	0.95
8923	U	0.651	0.92
8924	U	0.922	0.82
8925	A	1.411	0.21
8926	U	0.787	0.2
8927	A	1.614	0.12
8928	A	1.044	0.13
8929	A	1.221	0.13
8930	A	1.506	0.19
8931	G	1.329	0.17
8932	A	1.451	0.39
8933	A	0.936	0.84
8934	A	1.478	0.81
8935	A	0.57	0.68
8936	G	0.393	0.24
8937	G	0.203	0.19
8938	G	0.109	0.06
8939	G	0.136	0.09
8940	G	0.054	0.09
8941	G	0.176	0.2

8942	A	0.149	0.35
8943	C	0	0.63
8944	U	0	0.68
8945	G	0.651	0.39
8946	G	0.827	0.21
8947	A	0.977	0.23
8948	A	1.234	0.24
8949	G	0.543	0.17
8950	G	0.42	0.22
8951	G	0.556	0.31
8952	A	0.461	0.34
8953	U	0.339	0.48
8954	U	0.8	0.65
8955	U	0.556	0.82
8956	A	1.451	0.8
8957	U	0.732	0.71
8958	U	0.882	0.68
8959	A	1.248	0.44
8960	C	0.434	0.09
8961	A	0.936	0.3
8962	G	0.515	0.59
8963	U	0.176	0.73
8964	G	0.678	0.37
8965	C	0.19	0.31
8966	A	0.882	0.21
8967	A	1.465	0.15
8968	G	1.655	0.14
8969	A	1.289	0.22
8970	A	0.692	0.32
8971	G	0.488	0.43
8972	A	0.841	0.51
8973	C	0.081	0.42
8974	A	0.882	0.36
8975	U	0.61	0.49
8976	A	1.383	0.35
8977	G	1.058	0.33
8978	A	0.814	0.14
8979	A	0.637	0.2
8980	U	0.244	0.36
8981	C	0.19	0.27
8982	U	0.231	0.96
8983	U	0.637	0.94
8984	A	1.641	0.63

8985	G	0.841	0.09
8986	A	0.739	0.11
8987	C	0.21	0.22
8988	A	0.936	0.51
8989	U	0.176	0.55
8990	A	1.533	0.55
8991	U	0.631	0.68
8992	A	0.875	0.61
8993	C	0.068	0.86
8994	U	0.339	0.93
8995	U	0.814	0.87
8996	A	1.818	0.26
8997	G	1.275	0.03
8998	A	1.343	0.04
8999	A	1.153	0.04
9000	A	0.882	0.03
9001	A	0.97	0.04
9002	G	0.699	0.05
9003	G	0.936	0.05
9004	A	1.01	0.08
9005	A	0.834	0.09
9006	G	0.902	0.08
9007	A	1.065	0.24
9008	A	0.821	0.77
9009	G	0.353	0.82
9010	G	0.237	0.79
9011	C	0.115	0.52
9012	A	0.448	0.59
9013	U	0.387	0.68
9014	C	0.373	0.45
9015	A	1.065	0.7
9016	U	0.57	0.72
9017	A	0.644	0.17
9018	C	0.136	0.36
9019	C	0.38	0.29
9020	A	0.699	0.14
9021	G	0.78	0.11
9022	A	0.902	0.53
9023	U	0.454	0.62
9024	U	0.305	0.48
9025	G	0.163	0.46
9026	G	0.271	0.34
9027	C	0.224	0.13

9028	A	0.414	0.07
9029	G	0.298	0.11
9030	G	0.414	0.13
9031	A	0.556	0.13
9032	U	0.366	0.19
9033	U	0.875	0.21
9034	A	1.153	0.14
9035	C	0.407	0.15
9036	A	0.678	0.17
9037	C	0.102	0.29
9038	C	0.183	0.37
9039	U	0.217	0.17
9040	C	0.102	0.11
9041	A	0.97	0.73
9042	G	0.827	0.71
9043	G	0.61	0.11
9044	A	0.502	0.06
9045	C	0.197	0.16
9046	C	0.278	0.17
9047	A	0.604	0.23
9048	G	0.359	0.21
9049	G	0.651	0.17
9050	A	0.658	0.09
9051	A	0.861	0.12
9052	U	0.705	0.69
9053	U	0.949	0.79
9054	A	1.343	0.28
9055	G	0.509	0.27
9056	A	0.644	0.19
9057	U	0.61	0.22
9058	A	0.916	0.12
9059	C	0.102	0.08
9060	C	0.088	0.14
9061	C	0.068	0.15
9062	A	0.8	0.1
9063	A	0.773	0.08
9064	A	0.997	0.1
9065	G	0.726	0.13
9066	A	0.949	0.19
9067	C	0.556	0.15
9068	A	0.868	0.5
9069	U	0.387	0.91
9070	U	0.624	0.63

9071	U	0.997	0.14
9072	G	0.4	0.14
9073	G	0.068	0.13
9074	C	0.21	0.07
9075	U	0.305	0.53
9076	G	0.21	0.74
9077	G	0.258	0.71
9078	C	0.556	0.25
9079	U	0.861	0.59
9080	A	0.739	0.52
9081	U	0.109	0.39
9082	G	0.57	0.28
9083	G	1.085	0.23
9084	A	1.275	0.03
9085	A	1.065	0.06
9086	A	1.377	0.05
9087	U	1.37	0.1
9088	U	1.105	0.39
9089	A	0.637	0.38
9090	G	0.115	0.3
9091	U	0.461	0.44
9092	C	0.57	0.43
9093	C	0.434	0.72
9094	C	0.475	0.75
9095	U	0.231	0.76
9096	G	0.787	0.23
9097	U	0.882	0.37
9098	A	2.13	0.52
9099	A	1.506	0.35
9100	A	1.166	0.18
9101	U	0.61	0.02
9102	G	0.57	0.03
9103	U	0.515	0.03
9104	A	0.773	0
9105	U	0.597	0.23
9106	C	0.773	0.24
9107	A	1.072	0.07
9108	G	1.289	0.04
9109	A	1.668	0.04
9110	U	0.868	0.04
9111	G	1.099	0.19
9112	A	0.8	0.18
9113	G	0.42	0.01

9114	G	0.543	0
9115	C	0.515	0
9116	A	1.194	0.01
9117	C	0.705	0
9118	A	0.882	0
9119	G	0.543	0
9120	G	1.261	0
9121	A	0.827	0
9122	G	0.583	0.01
9123	G	0.787	0.01
9124	A	1.275	0
9125	U	1.004	0.01
9126	G	1.75	0.01
9127	A	1.94	0.01
9128	G	0.285	0.01
9129	G	0.285	0
9130	A	0.61	0
9131	G	0	0
9132	C	0	0.01
9133	A	0.407	0.01
9134	U	0.393	0.02
9135	U	1.153	0.02
9136	A	1.845	0
9137	U	0.583	0
9138	U	1.166	0.01
9139	U	1.397	0.01
9140	A	0.624	0
9141	A	0.312	0
9142	U	0.312	0.98
9143	G	0.163	0.98
9144	C	0.475	0.81
9145	A	1.695	0
9146	U	0.122	0
9147	C	0.109	0
9148	C	0.366	0.01
9149	A	0.895	0.01
9150	G	0.8	0.81
9151	C	0.312	0.94
9152	U	0.217	0.94
9153	C	0.231	0.04
9154	A	0.773	0.04
9155	A	0.773	0
9156	A	0.068	0

9157	C	0.271	0.01
9158	U	0.312	0.01
9159	U	0.163	0.01
9160	C	0.339	0
9161	C	0.38	0
9162	C	0.326	0
9163	A	0.665	0.03
9164	G	0.868	0.03
9165	U	0.529	0.01
9166	G	0.448	0.02
9167	G	0.163	0.81
9168	G	0.841	0.81
9169	A	1.153	0.03
9170	U	0.556	0.06
9171	G	0.827	0.06
9172	A	1.072	0.03
9173	C	0.122	0.7
9174	C	0.163	0.7
9175	C	0.136	0.18
9176	U	0.448	0.18
9177	U	0.583	0.04
9178	G	0.746	0.39
9179	G	0.57	0.44
9180	G	1.723	0.46
9181	G	0.448	0.08
9182	A	0.434	0.07
9183	G	2.238	0.06
9184	A	1.18	0.34
9185	G	0.475	0.35
9186	G	0.475	0.33
9187	U	0.203	0.68
9188	U	0.19	0.49
9189	C	0.244	0.72
9190	U	0.76	0.31
9191	A	0.597	0
9192	G	0.583	7.00E-04
9193	C	0.298	0
9194	A	1.75	0.08
9195	U	1.072	0.15
9196	G	0.732	0.2
9197	G	0.8	0.16
9198	A	1.316	0.36
9199	A	0.895	0.82

9200	G	0.488	0.77
9201	U	0.312	0.38
9202	U	0.529	0.39
9203	U	0.475	0.38
9204	G	0.692	0.05
9205	A	0.895	0.04
9206	U	0.678	0.54
9207	C	0.326	0.6
9208	C	0.42	0.27
9209	A	0.42	0.24
9210	A	0.665	0.11
9211	C	0.095	0.04
9212	U	0.285	0.01
9213	C	0.271	0.13
9214	U	0.61	0.18
9215	G	0.827	0.1
9216	G	0.258	0.09
9217	C	0.095	0.04
9218	C	0.271	0.22
9219	U	0.217	0.25
9220	A	0.909	0.25
9221	C	0.475	0.17
9222	A	0.502	0.07
9223	C	0.176	0.06
9224	U	0.543	0.13
9225	U	1.024	0.14
9226	A	1.445	0.22
9227	U	0.583	0.2
9228	G	0.712	0.17
9229	A	0.373	0.17
9230	G	0.203	0.19
9231	G	0.183	0.21
9232	C	0.163	0.03
9233	A	0.78	0
9234	U	0.929	0
9235	A	1.424	0
9236	U	0.197	0.01
9237	G	0.576	0.01
9238	U	0.4	0
9239	U	0.536	0
9240	A	0.956	0
9241	G	0.59	0
9242	A	0.665	0

9243	U	0.522	0.08
9244	A	0.549	0.11
9245	C	0.149	0.34
9246	C	0.21	0.99
9247	C	0.258	0.95
9248	A	1.004	0.83
9249	G	0.949	0.75
9250	A	1.085	0.71
9251	A	1.621	0.02
9252	G	0.631	0
9253	A	0.312	0
9254	G	0.231	0
9255	U	0.359	0.64
9256	U	0.57	0.77
9257	U	0.461	0.85
9258	G	1.004	0.9
9259	G	1.445	0.99
9260	A	0.651	0.44
9261	A	0.637	0.11
9262	G	0.366	0.07
9263	C	0.38	0.01
9264	A	0.543	0.01
9265	A	0.597	0.87
9266	G	0.176	0.93
9267	U	0.149	0.98
9268	C	0.285	0.06
9269	A	0.502	0.03
9270	G	0.407	0.04
9271	G	0.475	0.04
9272	C	0.095	0.92
9273	C	0.176	0.94
9274	U	0.163	0.88
9275	G	0.271	0.01
9276	U	0.312	0.18
9277	C	0.231	0.26
9278	A	0.556	0.22
9279	G	0.773	0.05
9280	A	0.705	0.05
9281	G	0.705	0.01
9282	G	0.692	0.01
9283	A	1.044	0.01
9284	A	0.42	0.02
9285	G	0.936	0.02

9286	A	1.194	0.01
9287	G	0.488	0
9288	G	0.258	0.21
9289	U	0.258	0.22
9290	U	0.855	0.13
9291	A	1.316	0
9292	G	2.021	0
9293	A	0.963	0.01
9294	A	0.136	0.01
9295	G	0.176	0.01
9296	A	0.271	0.01
9297	A	0.122	0.01
9298	G	0.42	0.01
9299	G	0.027	0.01
9300	C	0.014	0.99
9301	U	0.298	0.99
9302	A	0.909	0.01
9303	A	0.665	0
9304	C	0.122	0
9305	C	0.271	0
9306	G	0.57	0
9307	C	0.502	0.01
9308	A	0.692	0.01
9309	A	0.529	0.56
9310	G	0.583	0.56
9311	A	0.651	0.41
9312	G	0.326	0.43
9313	G	0.149	0.06
9314	C	0.163	0
9315	C	0.298	0.18
9316	U	0.203	0.25
9317	U	0.109	0.08
9318	C	0.081	0.04
9319	U	0.203	0.18
9320	U	0.624	0.72
9321	A	1.37	0.65
9322	A	0.529	0.17
9323	C	0.515	0.03
9324	A	0.651	0.02
9325	U	0.61	0.14
9326	G	0.434	0.18
9327	G	0.231	0.07
9328	C	0.014	0.16

9329	U	0.217	0.64
9330	G	0.38	0.71
9331	A	0.393	0.19
9332	C	0.258	0.04
9333	A	0.814	0.03
9334	A	0.76	0.03
9335	G	0.855	0
9336	A	1.031	0
9337	A	1.004	0
9338	G	0.705	0
9339	G	0.746	0
9340	A	0.665	0
9341	A	0.529	4.61E-06
9342	A	0.38	4.98E-06
9343	C	0	2.02E-05
9344	U	0.203	2.63E-05
9345	C	0.136	1.67E-05
9346	G	0.258	0
9347	C	0.027	0.05
9348	U	0.326	0.05
9349	G	0.732	0.04
9350	A	1.112	0.14
9351	A	0.99	0.15
9352	A	0.746	0.07
9353	C	0.61	0.07
9354	A	0.475	0.07
9355	G	0.326	0.13
9356	C	0.203	0.85
9357	A	0.597	0.97
9358	G	0.136	1
9359	G	0.109	1
9360	G	0.19	1
9361	A	0.461	0.96
9362	C	0.081	0.84
9363	U	0.176	0.96
9364	U	0.217	1
9365	U	0.081	1
9366	C	0.041	0.99
9367	C	0.109	0.96
9368	A	0.502	0.28
9369	C	0.42	0.39
9370	A	0.855	0.56
9371	A	1.044	0.89

9372	G	0.353	0.89
9373	G	0.122	0.46
9374	G	0.041	0.97
9375	G	0.19	0.96
9376	A	0.475	0.86
9377	U	0.312	0.73
9378	G	0.651	0.73
9379	U	0.461	0.55
9380	U	0.705	0.45
9381	A	0.882	0.59
9382	C	0.448	0.6
9383	G	0.298	0.32
9384	G	0.149	0.13
9385	G	0.081	0.12
9386	G	0.339	0.37
9387	A	0.597	0.41
9388	G	0.163	0.17
9389	G	0.231	0.22
9390	U	0.217	0.13
9391	A	0.556	0.1
9392	C	0.136	0.15
9393	U	0.163	0.25
9394	G	0.095	0.86
9395	G	0.027	0.91
9396	G	0.081	0.98
9397	G	0.624	0.98
9398	A	0.678	0.97
9399	G	0.949	0.9
9400	G	0.583	0.62
9401	A	0.637	0.15
9402	G	0.041	0.06
9403	C	0.203	0.05
9404	C	0.109	0.18
9405	G	0.529	0.85
9406	G	0.827	0.88
9407	U	0.407	0.85
9408	C	0.312	0.32
9409	G	0.298	0.76
9410	G	0.149	0.96
9411	G	0.366	0.96
9412	A	0.787	0.92
9413	A	0.882	0.93
9414	C	0.515	0.92

9415	G	0.393	0.79
9416	C	0.054	0.9
9417	C	0.054	0.97
9418	C	0.163	0.87
9419	A	0.705	0.22
9420	C	0.041	0.02
9421	U	0.543	0.03
9422	U	0.515	0.15
9423	U	0.42	0.45
9424	C	0.203	0.42
9425	U	0.665	0.46
9426	U	0.665	0.32
9427	G	0.787	0.54
9428	A	0.895	0.83
9429	U	0.285	0.68
9430	G	0.882	0.8
9431	U	0.651	0.78
9432	A	1.506	0.84
9433	U	1.031	0.78
9434	A	1.777	0.7
9435	A	1.234	0.43
9436	A	1.397	0.29
9437	U	1.18	0.06
9438	A	1.316	0.29
9439	U	0.461	0.65
9440	C	0.597	0.7
9441	A	0.705	0.58
9442	C	0.122	0.31
9443	U	0.312	0.31
9444	G	0.339	0.62
9445	C	0.637	0.6
9446	A	1.031	0.34
9447	U	0.529	0.34
9448	U	0.692	0.01
9449	U	0.719	0.03
9450	C	0.461	0.03
9451	G	0.841	0.53
9452	C	0.136	0.85
9453	U	0.19	0.44
9454	C	0.149	0.28
9455	U	0.353	0.62
9456	G	0.882	0.81
9457	U	1.031	0.87

9458	A	1.519	0.83
9459	U	1.017	0.59
9460	U	1.885	0.21
9461	C	0.855	0.71
9462	A	0.461	1
9463	G	0.054	1
9464	U	0.068	0.87
9465	C	0.054	0.93
9466	G	0	1
9467	C	0	1
9468	U	0	1
9469	C	0	1
9470	U	0.014	1
9471	G	0	1
9472	C	0.217	0.98
9473	G	3.459	0.62
9474	G	0.109	0.98
9475	A	0.041	1
9476	G	0.068	1
9477	A	0.095	1
9478	G	0.258	0.98
9479	G	0.068	0.99
9480	C	0.014	1
9481	U	0.922	1
9482	G	0.068	1
9483	G	0.095	0.98
9484	C	0.909	0
9485	A	0.176	0.97
9486	G	0.122	1
9487	A	0.949	0.97
9488	U	2.17	3.10E-05
9489	U	0.814	7.00E-06
9490	G	0.203	0.98
9491	A	0.203	1
9492	G	0.027	0.98
9493	C	0.014	0.98
9494	C	0.014	0.98
9495	C	0.244	0.03
9496	U	1.506	0
9497	G	2.143	0
9498	G	1.438	0
9499	G	0.787	0
9500	A	3.676	0

9501	G	0.136	0.99
9502	G	0.054	1
9503	U	0.109	0.98
9504	U	0.054	1
9505	C	0.054	0.98
9506	U	0.041	0.97
9507	C	0.027	1
9508	U	0	0.97
9509	C	0.014	0.99
9510	C	0.095	1
9511	A	0.041	1
9512	G	0	1
9513	C	0.326	0.98
9514	A	2.523	0.03
9515	C	0.149	0.97
9516	U	0.163	0.99
9517	A	0.19	1
9518	G	0.041	1
9519	C	0.095	1
9520	A	0.149	1
9521	G	0.054	1
9522	G	0.339	0.98
9523	U	1.343	0
9524	A	1.18	0
9525	G	0.271	0.95
9526	A	0.285	0.97
9527	G	0.122	0.96
9528	C	0.081	0.97
9529	C	0.326	0.03
9530	U	1.234	0.02
9531	G	2.116	0.03
9532	G	1.546	0.03
9533	G	0.583	0.05
9534	U	0.583	0.02
9535	G	0.068	0.93
9536	U	0.081	0.95
9537	U	0.054	0.97
9538	C	0.054	0.98
9539	C	0.041	0.98
9540	C	1.329	1
9541	U	0.149	0.99
9542	G	0.014	1
9543	C	0.095	1

9544	U	0.081	1
9545	A	0.217	1
9546	G	0.231	0.98
9547	A	0.312	0.03
9548	C	0.068	0.98
9549	U	0.014	1
9550	C	0.014	1
9551	U	0.014	1
9552	C	0.014	0.98
9553	A	0.149	0.05
9554	C	0.217	0.98
9555	C	0.041	1
9556	A	0.095	1
9557	G	0.054	1
9558	C	0.054	1
9559	A	0.014	1
9560	C	0.76	0.99
9561	U	1.804	0.59
9562	U	0.475	0
9563	G	0.448	0
9564	G	0.163	0
9565	C	0.176	0
9566	C	0.081	1.00E-06
9567	G	0.027	0.59
9568	G	0.014	0.99
9569	U	0.014	1
9570	G	0.014	1
9571	C	0.122	1
9572	U	0.353	1
9573	G	1.099	1
9574	G	0.122	0.98
9575	G	0.095	0.98
9576	C	0.122	1
9577	A	0.014	1
9578	G	0.136	1
9579	A	0.027	1
9580	G	0.095	1
9581	U	0.136	1
9582	G	0.068	1
9583	A	0.285	0.99
9584	C	0.502	0.98
9585	U	0.502	0.85
9586	C	0.61	0.01

9587	C	1.058	0.77
9588	A	0.515	0.99
9589	C	0.61	0.98
9590	G	0.258	0.97
9591	C	0.61	0.99
9592	U	0.583	1
9593	U	0.353	0.99
9594	G	0.122	0.99
9595	C	0.339	1
9596	U	0.814	1
9597	U	0.027	1
9598	G	0.42	0.99
9599	C	0.99	0.99
9600	U	1.492	1
9601	U	1.194	0.96
9602	A	1.044	0.1
9603	A	0.149	0.1
9604	A	0	0.05
9605	G	0.095	0.02
9606	C	0.054	0
9607	C	0.014	0
9608	C	0.095	0.11
9609	U	0.475	0.11
9610	C	0.597	0.06
9611	U	0.515	0.59
9612	U	1.112	0.8
9613	C	1.451	0.8
9614	A	0.868	0.79
9615	A	1.424	0.77
9616	U	1.044	0.76
9617	A	1.112	0.34
9618	A	0.529	0.22
9619	A	0.977	0.19
9620	G	0.732	0.03
9621	C		0.04
9622	U		0.13
9623	G		0.13
9624	C		0
9625	C		0
9626	A		0.07
9627	U		0.51
9628	U		0.94
9629	U		0.95

9630	U	0.95
9631	A	0.97
9632	G	0.72
9633	A	0.92
9634	A	0.97
9635	G	1
9636	U	1
9637	A	1
9638	A	1
9639	A	1
9640	A	0.98
9641	A	0.98
9642	A	0.99
9643	A	0.98
9644	A	0.96
9645	A	0.98
9646	A	0.86
9647	A	0.97
