# Supplementary material for: Comparison of SIV and HIV-1 Genomic RNA Structures Reveals Impact of Sequence Evolution on Conserved and Non-Conserved Structural Motifs
Source: PLoS Pathog. 2013 Apr 4;9(4):e1003294. doi: 10.1371/journal.ppat.1003294 (PMC3616985; doi:10.1371/journal.ppat.1003294)
Supplement: Dataset S3 — Helix file for the HIV-1NL4-3 RNA genome structure model folded with parameters m = 1.9 and b = −0.7. (PDF) [file ppat.1003294.s007.pdf]

DATASET S3 Helix file for the HIV-1NL4-3 RNA genome structure model folded with parameters m = 1.9 and b = -0.7.

First nucleotide in helix	Last nucleotide in helix	Number of base pairs in helix
1	57	3
5	54	11
17	43	5
25	38	4
58	104	8
67	94	3
70	90	4
105	344	10
125	223	7
134	178	8
143	167	2
146	164	2
148	160	3
206	213	2
228	334	6
236	282	3
243	277	4
248	270	7
283	299	3
286	295	3
312	325	5
346	749	3
350	746	2
352	743	1
354	741	6
363	734	4
382	537	4
394	532	4
399	484	9
501	526	6
510	518	2
547	565	4
582	657	2
586	652	9
595	625	4
599	616	5
627	637	4
678	691	2
693	722	6

702	714	2
752	1172	5
759	775	5
795	849	9
821	831	4
855	994	2
871	913	2
874	911	6
915	970	3
921	964	5
926	935	3
946	957	2
979	991	5
997	1014	5
1026	1068	2
1028	1064	5
1035	1055	4
1076	1100	3
1080	1097	5
1102	1142	5
1110	1137	3
1116	1132	6
1174	1764	2
1177	1761	2
1180	1759	6
1193	1299	6
1200	1206	2
1211	1251	3
1214	1247	5
1223	1239	6
1252	1283	4
1260	1273	5
1301	1351	5
1308	1345	5
1319	1335	4
1360	1394	4
1375	1387	5
1396	1558	4
1401	1554	3
1405	1414	2
1418	1457	3
1421	1443	7
1459	1522	6

1465	1515	3
1469	1511	3
1476	1505	4
1481	1500	4
1531	1541	3
1568	1707	10
1583	1694	3
1593	1599	2
1604	1636	8
1615	1625	3
1638	1682	7
1645	1672	12
1709	1728	3
1713	1724	4
1731	1752	5
1767	1779	2
1781	1973	5
1788	1953	7
1796	1946	7
1805	1938	4
1810	1918	2
1813	1916	7
1823	1849	6
1829	1842	3
1862	1881	5
1922	1930	3
1991	2326	9
2015	2121	8
2024	2112	10
2042	2070	7
2051	2062	3
2072	2082	3
2135	2144	3
2145	2171	6
2192	2199	2
2201	2238	7
2245	2260	4
2268	2310	5
2273	2301	4
2278	2297	2
2330	2366	6
2340	2357	5
2378	2429	8

2392	2415	3
2398	2412	2
2401	2409	2
2430	2958	2
2434	2954	2
2447	2952	8
2468	2944	7
2476	2532	5
2482	2527	2
2492	2517	3
2496	2513	2
2537	2586	5
2544	2580	3
2548	2576	6
2558	2565	2
2602	2927	5
2619	2916	4
2625	2657	3
2629	2654	10
2659	2911	4
2663	2906	11
2683	2895	2
2702	2771	4
2708	2761	4
2721	2750	4
2727	2745	4
2731	2740	2
2772	2887	2
2774	2882	5
2781	2802	7
2810	2836	3
2814	2833	6
2843	2857	5
2959	2970	3
2972	3303	2
2975	3301	6
2995	3294	3
3001	3036	5
3014	3024	4
3041	3255	5
3055	3245	5
3074	3089	2
3092	3176	4

3099	3172	3
3103	3131	4
3139	3150	4
3178	3190	3
3205	3223	3
3269	3290	5
3307	3351	12
3319	3336	3
3323	3332	3
3353	3726	5
3367	3710	2
3371	3706	7
3392	3427	5
3398	3418	7
3429	3699	2
3431	3696	4
3438	3692	2
3443	3687	3
3458	3468	3
3470	3668	4
3491	3510	1
3493	3509	5
3525	3563	7
3536	3554	4
3582	3650	4
3587	3645	5
3593	3604	4
3606	3639	2
3609	3636	6
3652	3663	2
3745	4058	3
3749	4053	3
3754	4048	9
3773	3791	7
3796	3922	3
3799	3917	2
3811	3906	3
3818	3899	9
3830	3838	2
3840	3890	6
3847	3883	2
3850	3880	3
3857	3872	4

3924	4012	7
3939	4005	5
3945	3999	1
3947	3998	6
3956	3992	4
3962	3980	5
3968	3975	2
4014	4020	2
4031	4038	2
4068	4098	3
4072	4095	6
4078	4088	2
4099	4129	4
4131	4518	2
4135	4514	7
4143	4357	4
4158	4175	6
4183	4195	2
4199	4353	4
4204	4349	8
4215	4265	3
4280	4299	4
4301	4316	4
4321	4341	2
4362	4503	7
4447	4488	5
4453	4482	1
4456	4479	3
4459	4473	3
4490	4496	2
4551	5036	10
4573	4586	4
4588	4934	10
4601	4914	8
4614	4902	2
4617	4692	4
4633	4682	5
4642	4674	3
4646	4670	4
4694	4732	3
4698	4728	4
4702	4721	7
4741	4752	2

4754	4770	6
4797	4899	7
4807	4822	3
4829	4891	9
4840	4856	7
4938	4999	6
4951	4985	5
4960	4980	6
5010	5022	5
5079	5435	3
5083	5431	5
5091	5426	5
5099	5208	3
5103	5205	5
5114	5132	4
5139	5163	2
5144	5158	4
5166	5194	6
5209	5394	5
5216	5384	9
5234	5265	3
5239	5261	5
5245	5255	2
5267	5297	6
5273	5283	4
5303	5343	4
5307	5337	4
5311	5331	6
5349	5370	2
5352	5367	1
5355	5364	3
5404	5419	6
5436	5791	3
5440	5787	4
5444	5455	4
5467	5774	13
5482	5755	7
5499	5519	3
5503	5515	4
5530	5581	5
5536	5576	3
5542	5571	2
5545	5568	3

5549	5565	2
5588	5659	2
5599	5645	3
5605	5639	6
5612	5632	1
5614	5631	4
5664	5691	3
5668	5687	6
5725	5740	4
5793	6013	7
5803	6004	5
5816	5830	6
5846	5861	6
5866	5998	8
5874	5989	4
5889	5985	4
5895	5981	7
5904	5913	2
5915	5973	3
5927	5962	4
5931	5952	4
5936	5947	4
6020	6318	8
6029	6050	4
6051	6134	2
6062	6123	7
6070	6098	4
6075	6093	3
6101	6113	3
6135	6201	3
6139	6197	2
6141	6194	3
6144	6190	4
6149	6185	2
6155	6180	4
6159	6175	2
6207	6249	4
6212	6244	3
6219	6241	5
6224	6235	2
6251	6258	2
6270	6290	6
6328	6798	5

6334	6355	4
6358	6371	3
6372	6792	4
6381	6780	4
6385	6775	3
6390	6770	1
6393	6767	2
6398	6704	5
6404	6415	3
6416	6695	4
6422	6690	4
6429	6685	3
6432	6681	3
6436	6677	2
6456	6467	4
6469	6657	2
6475	6651	7
6483	6628	2
6485	6625	5
6499	6620	5
6506	6611	4
6515	6603	3
6518	6537	4
6522	6531	3
6538	6595	4
6543	6590	3
6546	6586	6
6552	6579	4
6706	6762	2
6711	6757	9
6801	6820	6
6839	7188	4
6846	7179	2
6850	7176	5
6855	7170	3
6861	7155	7
6869	6887	7
6893	7074	1
6895	7073	7
6904	7056	10
6923	7040	4
6929	7036	5
6937	6957	6

6959	7009	5
6965	6989	2
6971	6984	3
6992	7004	4
7076	7112	5
7081	7097	5
7113	7137	9
7235	7778	5
7245	7599	1
7247	7597	5
7256	7590	8
7272	7578	11
7283	7566	3
7291	7557	7
7298	7547	4
7304	7539	8
7312	7530	3
7316	7526	5
7321	7520	4
7325	7515	4
7333	7508	5
7338	7502	4
7343	7408	6
7350	7378	1
7353	7375	3
7356	7371	2
7358	7368	4
7383	7399	7
7411	7428	7
7437	7465	4
7443	7459	7
7468	7493	9
7601	7616	4
7621	7643	3
7627	7636	2
7647	7692	6
7658	7682	3
7705	7770	6
7712	7764	2
7716	7760	2
7732	7743	3
7792	8218	12
7825	8161	8

7852	7894	9
7863	7883	3
7866	7877	3
7896	8152	7
7916	8131	9
7928	8118	4
7933	8114	5
7940	8107	3
7944	8049	5
7949	8043	3
7954	8039	3
7965	8029	3
7968	8025	5
7975	8018	3
7991	8015	5
7997	8009	4
8052	8100	4
8059	8071	3
8073	8087	5
8169	8205	4
8173	8194	3
8178	8190	4
8224	8555	3
8228	8551	6
8235	8247	3
8262	8280	2
8265	8277	4
8282	8348	5
8288	8339	6
8302	8327	2
8305	8325	5
8310	8319	3
8359	8545	10
8370	8534	8
8399	8412	4
8418	8442	4
8422	8435	4
8461	8498	11
8499	8519	3
8502	8513	1
8503	8511	3
8578	9139	5
8591	8624	2

8596	8619	2
8599	8616	5
8626	9036	6
8635	9028	8
8649	8666	2
8651	8663	4
8667	8681	5
8686	9009	7
8694	8999	5
8700	8803	6
8710	8792	4
8721	8781	3
8724	8750	10
8753	8773	8
8807	8994	6
8817	8985	5
8830	8973	4
8837	8966	3
8842	8961	2
8845	8932	4
8851	8859	2
8867	8906	10
8878	8896	4
8884	8890	2
8912	8927	5
8934	8951	4
9010	9019	2
9042	9057	5
9059	9067	2
9074	9134	5
9080	9129	11
9092	9118	5
9100	9113	4
9141	9170	4
9145	9165	5
