# Supplementary material for: Comparison of SIV and HIV-1 Genomic RNA Structures Reveals Impact of Sequence Evolution on Conserved and Non-Conserved Structural Motifs
Source: PLoS Pathog. 2013 Apr 4;9(4):e1003294. doi: 10.1371/journal.ppat.1003294 (PMC3616985; doi:10.1371/journal.ppat.1003294)
Supplement: Dataset S4 — SHAPE reactivites for the HIV-1NL4-3 genome normalized by box-plot normalization. (PDF) [file ppat.1003294.s008.pdf]

DATASET S4 SHAPE reactivities for the HIV-1NL4-3 genome normalized by box-plot normalization.

Nucleotide position	Nucleotide identity	SHAPE reactivity
1	G	
2	G	
3	U	
4	C	
5	U	
6	C	
7	U	
8	C	
9	U	
10	G	
11	G	
12	U	0.093
13	U	0.013
14	A	0.12
15	G	0.453
16	A	3.823
17	C	0.107
18	C	0.186
19	A	0.04
20	G	0.04
21	A	1.705
22	U	1.825
23	C	4.835
24	U	0.293
25	G	0.067
26	A	0.067
27	G	0
28	C	0
29	C	0.32
30	U	1.425
31	G	1.771
32	G	0.839
33	G	0.186
34	A	2.251
35	G	0
36	C	0
37	U	0.053
38	C	0.013
39	U	0.133

40	C	0.107
41	U	0.213
42	G	0
43	G	0
44	C	0
45	U	0.053
46	A	0.08
47	A	0.053
48	C	0
49	U	0.186
50	A	0.306
51	G	0.812
52	G	0.067
53	G	0.08
54	A	0.053
55	A	0.013
56	C	0.04
57	C	0.559
58	C	0.013
59	A	0.04
60	C	0.133
61	U	0.426
62	G	0.293
63	C	0.013
64	U	0.107
65	U	0.253
66	A	0.346
67	A	0.173
68	G	0.027
69	C	0
70	C	0.013
71	U	0.013
72	C	0.013
73	A	0.32
74	A	0.799
75	U	0.413
76	A	1.079
77	A	1.079
78	A	1.319
79	G	0.12
80	C	0.08
81	U	0.213
82	U	0.226

83	G	0.306
84	C	0.053
85	C	0.093
86	U	0.413
87	U	0.333
88	G	0.266
89	A	0.08
90	G	0.16
91	U	0.506
92	G	0
93	C	0.013
94	U	0.053
95	C	0.226
96	A	0.986
97	A	0.373
98	A	0.173
99	G	0.12
100	U	0.16
101	A	0.253
102	G	0.16
103	U	0.013
104	G	0.107
105	U	0.333
106	G	0.799
107	U	0.16
108	G	0.133
109	C	0.04
110	C	0.107
111	C	0.067
112	G	0.266
113	U	0.08
114	C	0.08
115	U	0.226
116	G	0.666
117	U	0.226
118	U	0.373
119	G	0.733
120	U	0.506
121	G	0.786
122	U	0.266
123	G	0.679
124	A	0.613
125	C	0.027

126	U	0.08
127	C	0.107
128	U	0.08
129	G	0.133
130	G	0.16
131	U	0.573
132	A	1.185
133	A	0.666
134	C	0.04
135	U	0.12
136	A	0.253
137	G	0.133
138	A	0.133
139	G	0.08
140	A	0.293
141	U	0.16
142	C	0.013
143	C	0
144	C	0
145	U	0.16
146	C	0.08
147	A	0.186
148	G	0.08
149	A	0.053
150	C	0.053
151	C	0.266
152	C	0.16
153	U	0.386
154	U	0.453
155	U	0.839
156	U	0.759
157	A	0.666
158	G	0.067
159	U	0.067
160	C	0.04
161	A	0.506
162	G	0.493
163	U	0.253
164	G	0.586
165	U	0.373
166	G	0.253
167	G	0.186
168	A	0.932

169	A	1.598
170	A	0.852
171	A	0.226
172	U	0.04
173	C	0.013
174	U	0.013
175	C	0.093
176	U	0.067
177	A	0.186
178	G	0.346
179	C	0.266
180	A	1.039
181	G	0.866
182	U	0.386
183	G	0.053
184	G	0
185	C	0
186	G	0.013
187	C	0.12
188	C	0.067
189	C	0.013
190	G	0.027
191	A	0.027
192	A	0.12
193	C	0.186
194	A	0.107
195	G	0
196	G	0
197	G	0.013
198	A	0.013
199	C	0.147
200	U	1.345
201	U	0.799
202	G	0.546
203	A	0.839
204	A	1.145
205	A	1.132
206	G	1.119
207	C	0.226
208	G	0.839
209	A	1.012
210	A	1.052
211	A	0.892

212	G	0.812
213	U	0.773
214	A	1.185
215	A	1.079
216	A	1.518
217	G	0.226
218	C	0.12
219	C	0.067
220	A	0.4
221	G	0.333
222	A	0.546
223	G	0.333
224	G	0.599
225	A	0.759
226	G	0.852
227	A	0.533
228	U	0.186
229	C	0.013
230	U	0.186
231	C	0.213
232	U	0.186
233	C	0.12
234	G	0.44
235	A	0.719
236	C	0.067
237	G	0.226
238	C	0.293
239	A	1.638
240	G	7.006
241	G	2.531
242	A	1.145
243	C	0.067
244	U	0.067
245	C	0.04
246	G	0.133
247	G	0.519
248	C	0
249	U	0.133
250	U	0.04
251	G	0
252	C	0.013
253	U	0.12
254	G	0.213

255	A	1.185
256	A	1.678
257	G	0.08
258	C	0.013
259	G	0.053
260	C	0.12
261	G	0.12
262	C	0.093
263	A	0.093
264	C	0
265	G	0.133
266	G	0
267	C	0.16
268	A	0.08
269	A	0.12
270	G	0.213
271	A	1.598
272	G	1.585
273	G	2.051
274	C	0.053
275	G	0.12
276	A	0.093
277	G	0.173
278	G	0.413
279	G	0.426
280	G	0.533
281	C	0.107
282	G	0.16
283	G	0.24
284	C	0.107
285	G	0.906
286	A	0.733
287	C	0.306
288	U	0.373
289	G	0.12
290	G	0.293
291	U	0.972
292	G	1.385
293	A	0.666
294	G	0.306
295	U	0.493
296	A	0.479
297	C	0.386

298	G	0.386
299	C	0.08
300	C	0.639
301	A	0.986
302	A	0.799
303	A	0.613
304	A	0.812
305	A	0.533
306	U	0.546
307	U	0.373
308	U	0.546
309	U	0.679
310	G	1.039
311	A	0.386
312	C	0.186
313	U	0.213
314	A	0.253
315	G	0.067
316	C	0.333
317	G	1.252
318	G	0.826
319	A	1.385
320	G	0.133
321	G	0.186
322	C	0.16
323	U	0.173
324	A	0.186
325	G	0.613
326	A	1.132
327	A	0.653
328	G	0.972
329	G	0.346
330	A	0.426
331	G	1.066
332	A	0.852
333	G	0.519
334	A	0.413
335	G	0.613
336	A	0.373
337	U	0.346
338	G	0.067
339	G	0.04
340	G	0.067

341	U	0.173
342	G	0.053
343	C	0.733
344	G	0.759
345	A	0.653
346	G	0.906
347	A	0.306
348	G	0.04
349	C	0.253
350	G	0.186
351	U	0.253
352	C	0.346
353	G	0.586
354	G	0.16
355	U	0.866
356	A	0.266
357	U	0.466
358	U	0.626
359	A	0.586
360	A	0.226
361	G	0.12
362	C	0.08
363	G	0.013
364	G	0.16
365	G	0
366	G	0.186
367	G	0.653
368	A	0.453
369	G	0.573
370	A	0.559
371	A	0.107
372	U	0.586
373	U	1.079
374	A	0.812
375	G	1.265
376	A	0.426
377	U	1.079
378	A	0.44
379	A	0.706
380	A	0.426
381	U	0.493
382	G	0.586
383	G	0.226

384	G	1.132
385	A	0.639
386	A	0.759
387	A	0.932
388	A	0.639
389	A	0.666
390	A	0.466
391	U	0.546
392	U	0.253
393	C	0.413
394	G	0.826
395	G	0.839
396	U	0.679
397	U	0.293
398	A	0.24
399	A	0.08
400	G	0.2
401	G	0.36
402	C	0.053
403	C	0
404	A	0.093
405	G	0.186
406	G	0.067
407	G	0.24
408	G	0.812
409	G	0.759
410	A	0.906
411	A	1.212
412	A	0.986
413	G	1.199
414	A	1.039
415	A	0.666
416	A	0.253
417	C	0.932
418	A	1.145
419	A	0.053
420	U	0.999
421	A	0.067
422	U	1.199
423	A	0.786
424	A	0.533
425	A	0
426	C	0.546

427	U	0.573
428	A	0.653
429	A	0.826
430	A	0.719
431	A	0
432	C	1.185
433	A	0.08
434	U	1.718
435	A	0
436	U	0.852
437	A	0.586
438	G	0.28
439	U	0.932
440	A	0.466
441	U	0.44
442	G	0.2
443	G	0.04
444	G	0.027
445	C	0.413
446	A	0.32
447	A	0.32
448	G	0.013
449	C	0.706
450	A	0.533
451	G	0.812
452	G	0.186
453	G	0.759
454	A	0.2
455	G	0.053
456	C	0.226
457	U	1.292
458	A	0.812
459	G	1.185
460	A	0.946
461	A	0.373
462	C	0.866
463	G	1.026
464	A	0.333
465	U	0.546
466	U	0.24
467	C	0.932
468	G	0.013
469	C	0.773

470	A	0.44
471	G	0.426
472	U	0.746
473	U	0.746
474	A	0.746
475	A	0.293
476	U	0.107
477	C	0.16
478	C	0.213
479	U	0.12
480	G	0
481	G	0.786
482	C	1.745
483	C	0.08
484	U	0.36
485	U	1.425
486	U	1.665
487	U	1.811
488	A	0.799
489	G	1.292
490	A	0.852
491	G	1.172
492	A	0.013
493	C	0.866
494	A	0.426
495	U	0.12
496	C	1.225
497	A	0.719
498	G	1.012
499	A	1.598
500	A	0.413
501	G	0
502	G	0.093
503	C	0.107
504	U	0.493
505	G	0.479
506	U	1.159
507	A	0.693
508	G	1.052
509	A	0.346
510	C	0.879
511	A	0.666
512	A	0.972

513	A	0.506
514	U	0.613
515	A	0.133
516	C	0.173
517	U	0.2
518	G	0.2
519	G	0.36
520	G	0.466
521	A	0.053
522	C	0.773
523	A	0
524	G	0.053
525	C	0.306
526	U	0.613
527	A	0.027
528	C	0.559
529	A	0.666
530	A	0.12
531	C	0.12
532	C	0.293
533	A	0.147
534	U	0
535	C	0.053
536	C	0
537	C	0.147
538	U	1.012
539	U	0.333
540	C	1.132
541	A	1.052
542	G	0.932
543	A	0.133
544	C	0.759
545	A	0.826
546	G	0.12
547	G	0.599
548	A	0.4
549	U	0.053
550	C	0.812
551	A	1.185
552	G	1.172
553	A	0.959
554	A	1.092
555	G	1.252

556	A	0.759
557	A	0.107
558	C	0.413
559	U	0.693
560	U	1.092
561	A	0.44
562	G	0.653
563	A	0.16
564	U	0
565	C	1.092
566	A	0.613
567	U	0.679
568	U	1.412
569	A	0.067
570	U	1.758
571	A	0.506
572	U	1.066
573	A	1.159
574	A	0.799
575	U	0.613
576	A	0.373
577	C	0.493
578	A	0.826
579	A	0.479
580	U	0.932
581	A	0.16
582	G	0.013
583	C	0.613
584	A	0.533
585	G	0.186
586	U	0.12
587	C	0
588	C	0.147
589	U	0.147
590	C	0.2
591	U	0.107
592	A	0.2
593	U	0.133
594	U	0.559
595	G	1.079
596	U	0.107
597	G	0.759
598	U	0.306

599	G	0.36
600	C	0.067
601	A	0.213
602	U	0
603	C	0.173
604	A	0.413
605	A	0.559
606	A	0.586
607	G	0.32
608	G	0.386
609	A	0.639
610	U	0.719
611	A	1.478
612	G	0.413
613	A	0.4
614	U	0.08
615	G	0.173
616	U	0.226
617	A	1.412
618	A	0.852
619	A	1.012
620	A	0.999
621	G	0.839
622	A	0.573
623	C	0
624	A	0.306
625	C	0
626	C	0.12
627	A	0.932
628	A	0.693
629	G	0.759
630	G	0.493
631	A	0.906
632	A	1.332
633	G	0.107
634	C	0.133
635	C	0.107
636	U	0.08
637	U	0.426
638	A	0.986
639	G	0.546
640	A	0.466
641	U	0.253

642	A	1.105
643	A	1.598
644	G	0.733
645	A	0.493
646	U	0.226
647	A	0.426
648	G	0.133
649	A	0.226
650	G	0.333
651	G	0.573
652	A	0.4
653	A	0.986
654	G	1.532
655	A	1.305
656	G	0.453
657	C	0.107
658	A	1.212
659	A	0.972
660	A	0.906
661	A	0.506
662	C	0.067
663	A	0.946
664	A	0.706
665	A	1.199
666	A	1.052
667	G	0.959
668	U	0.013
669	A	1.039
670	A	0.759
671	G	
672	A	
673	A	1.105
674	A	1.239
675	A	1.132
676	A	0.932
677	G	0.626
678	G	0.013
679	C	0.186
680	A	0.466
681	C	0.013
682	A	0.653
683	G	1.212
684	C	0.386

685	A	0.839
686	A	1.159
687	G	0.28
688	C	0
689	A	0.573
690	G	0.107
691	C	0.08
692	A	0.506
693	G	0.147
694	C	0.426
695	U	0.133
696	G	0.226
697	A	0.266
698	C	0
699	A	1.039
700	C	0
701	A	1.159
702	G	0.506
703	G	0.746
704	A	0.959
705	A	1.172
706	A	0.799
707	C	0.08
708	A	0.759
709	A	0.746
710	C	0.067
711	A	0.759
712	G	0.32
713	C	0
714	C	0.093
715	A	0.426
716	G	0.266
717	G	0.12
718	U	0
719	C	0.4
720	A	0.293
721	G	0.16
722	C	0
723	C	0.613
724	A	0.666
725	A	0.573
726	A	0.599
727	A	0.306

728	U	0.559
729	U	0.613
730	A	0
731	C	0.08
732	C	0.093
733	C	0.013
734	U	0.266
735	A	0.839
736	U	0.706
737	A	1.026
738	G	0.706
739	U	0.28
740	G	0.12
741	C	0
742	A	1.572
743	G	2.97
744	A	1.026
745	A	1.026
746	C	0.386
747	C	0.253
748	U	0.28
749	C	0.12
750	C	0.133
751	A	0.293
752	G	0.226
753	G	0.08
754	G	0.213
755	G	0.573
756	C	0.067
757	A	0.719
758	A	1.039
759	A	0.866
760	U	0.453
761	G	0.386
762	G	0.333
763	U	0.107
764	A	0.586
765	C	0
766	A	0.293
767	U	0.147
768	C	0
769	A	0.266
770	G	0.506

771	G	0.027
772	C	0.133
773	C	0.053
774	A	0.506
775	U	0.413
776	A	0.373
777	U	0.28
778	C	0.16
779	A	0.479
780	C	0.067
781	C	0.24
782	U	0.466
783	A	1.372
784	G	2.064
785	A	1.185
786	A	0.693
787	C	0.186
788	U	0.386
789	U	0.626
790	U	0.972
791	A	1.412
792	A	0.866
793	A	1.239
794	U	0.852
795	G	0.173
796	C	0.173
797	A	0.04
798	U	0.027
799	G	0.226
800	G	0.253
801	G	0.226
802	U	0.08
803	A	0.559
804	A	0.906
805	A	0.986
806	A	0.879
807	G	1.052
808	U	0.706
809	A	0.799
810	G	0.986
811	U	0.706
812	A	1.478
813	G	1.132

814	A	1.239
815	A	1.212
816	G	1.332
817	A	1.265
818	G	1.172
819	A	0
820	A	1.851
821	G	1.398
822	G	0.093
823	C	0.346
824	U	0.426
825	U	0.493
826	U	0.213
827	C	0.333
828	A	0.599
829	G	0.093
830	C	0.173
831	C	0.453
832	C	0.186
833	A	0.706
834	G	0.919
835	A	1.092
836	A	1.026
837	G	1.066
838	U	0.759
839	A	1.212
840	A	1.319
841	U	0.519
842	A	0.293
843	C	0.053
844	C	0.133
845	C	0.12
846	A	0.16
847	U	0.346
848	G	0.493
849	U	0.213
850	U	0.892
851	U	0.599
852	U	0.346
853	C	0.906
854	A	0.493
855	G	0
856	C	0.693

857	A	0.293
858	U	0.413
859	U	1.185
860	A	0.639
861	U	0.107
862	C	1.319
863	A	1.012
864	G	1.425
865	A	1.425
866	A	1.132
867	G	1.478
868	G	1.931
869	A	0.093
870	G	0.2
871	C	0.266
872	C	0.932
873	A	0.16
874	C	0.093
875	C	0.013
876	C	0.04
877	C	0.333
878	A	0.067
879	C	0.986
880	A	1.105
881	A	0.746
882	G	1.026
883	A	0.24
884	U	0.533
885	U	0.626
886	U	0.826
887	A	0.733
888	A	1.039
889	A	0.613
890	U	0.812
891	A	0.04
892	C	0
893	C	0
894	A	0.666
895	U	0.479
896	G	0.32
897	C	0.306
898	U	0.559
899	A	0.879

900	A	0.559
901	A	0.533
902	C	0.24
903	A	1.105
904	C	0.453
905	A	1.132
906	G	0.16
907	U	0.24
908	G	0.533
909	G	0.333
910	G	0.306
911	G	0.266
912	G	0.346
913	G	0.293
914	A	0
915	C	0.639
916	A	0.453
917	U	0.386
918	C	1.105
919	A	1.305
920	A	0.253
921	G	0.08
922	C	0.44
923	A	0.12
924	G	0.067
925	C	0.04
926	C	0.493
927	A	0.306
928	U	0.533
929	G	0.173
930	C	0.693
931	A	0.626
932	A	0.679
933	A	0.213
934	U	0.466
935	G	0.24
936	U	0.866
937	U	1.265
938	A	0.626
939	A	0.826
940	A	0.892
941	A	0.932
942	G	1.079

943	A	0.812
944	G	0.693
945	A	0.147
946	C	0.107
947	C	0.373
948	A	0.226
949	U	0.32
950	C	0.946
951	A	0.999
952	A	0.599
953	U	1.026
954	G	0.679
955	A	0.453
956	G	0.413
957	G	0.746
958	A	0.812
959	A	0.333
960	G	0.093
961	C	0.08
962	U	0.067
963	G	0
964	C	0.733
965	A	0.972
966	G	1.225
967	A	0.839
968	A	0.413
969	U	0.333
970	G	0.24
971	G	0.333
972	G	0.599
973	A	0.666
974	U	1.265
975	A	0.773
976	G	0.799
977	A	0.559
978	U	0.586
979	U	0.226
980	G	0.067
981	C	0.373
982	A	0.12
983	U	0.107
984	C	0.173
985	C	0.479

986	A	0.466
987	G	0.266
988	U	0.253
989	G	0.093
990	C	0.346
991	A	0.133
992	U	0.346
993	G	0
994	C	0.213
995	A	0.746
996	G	0.946
997	G	0.107
998	G	0.027
999	C	0.053
1000	C	0.24
1001	U	0.812
1002	A	0.479
1003	U	0.333
1004	U	0.413
1005	G	0.04
1006	C	0.413
1007	A	0.027
1008	C	0.133
1009	C	0.293
1010	A	0.107
1011	G	0.04
1012	G	0.053
1013	C	0.053
1014	C	0.852
1015	A	0.706
1016	G	0.866
1017	A	0.333
1018	U	1.092
1019	G	0.759
1020	A	1.119
1021	G	0.892
1022	A	0.879
1023	G	0.759
1024	A	0.546
1025	A	0.027
1026	C	0
1027	C	0.426
1028	A	0.559

1029	A	0.253
1030	G	0.16
1031	G	0.08
1032	G	0.24
1033	G	0.466
1034	A	0.519
1035	A	0.493
1036	G	0.2
1037	U	0.586
1038	G	0.453
1039	A	0
1040	C	0.107
1041	A	0.826
1042	U	0.453
1043	A	0.839
1044	G	0.4
1045	C	0.013
1046	A	0.679
1047	G	0.466
1048	G	0.666
1049	A	0.812
1050	A	0.533
1051	C	0.12
1052	U	0.386
1053	A	0.466
1054	C	0.053
1055	U	0.586
1056	A	1.172
1057	G	0.519
1058	U	0.426
1059	A	0.333
1060	C	0
1061	C	0
1062	C	0.027
1063	U	0.067
1064	U	0.2
1065	C	0.093
1066	A	0.493
1067	G	0.226
1068	G	0.36
1069	A	0.573
1070	A	0.653
1071	C	0.186

1072	A	1.159
1073	A	1.079
1074	A	0.826
1075	U	0.586
1076	A	0.426
1077	G	0.147
1078	G	0.067
1079	A	1.505
1080	U	0.12
1081	G	0.067
1082	G	0.04
1083	A	0.04
1084	U	0.12
1085	G	0.679
1086	A	0.959
1087	C	0.253
1088	A	1.012
1089	C	0
1090	A	0.719
1091	U	0.519
1092	A	1.771
1093	A	0.147
1094	U	0.027
1095	C	0.013
1096	C	0.013
1097	A	0.093
1098	C	0.027
1099	C	0.053
1100	U	0.2
1101	A	0.293
1102	U	0.28
1103	C	0.12
1104	C	0.053
1105	C	0.013
1106	A	0.426
1107	G	2.304
1108	U	0.892
1109	A	0.786
1110	G	0.186
1111	G	0.333
1112	A	0.306
1113	G	0.186
1114	A	0.453

1115	A	0.799
1116	A	0.173
1117	U	0.053
1118	C	0.027
1119	U	0.173
1120	A	0.812
1121	U	0.599
1122	A	1.478
1123	A	0.852
1124	A	1.239
1125	A	0.786
1126	G	0.866
1127	A	0.466
1128	U	0.306
1129	G	0.266
1130	G	0.44
1131	A	0.586
1132	U	0.24
1133	A	0.559
1134	A	0.373
1135	U	0.12
1136	C	0.013
1137	C	0.013
1138	U	0.107
1139	G	0.186
1140	G	0.093
1141	G	0.107
1142	A	0.479
1143	U	0.679
1144	U	1.825
1145	A	1.385
1146	A	0.892
1147	A	1.372
1148	U	1.012
1149	A	1.452
1150	A	0.44
1151	A	0.812
1152	A	0.879
1153	U	0.599
1154	A	0.919
1155	G	0.679
1156	U	0.546
1157	A	1.385

1158	A	1.212
1159	G	0.986
1160	A	0.932
1161	A	1.052
1162	U	0.573
1163	G	1.225
1164	U	0.653
1165	A	1.119
1166	U	0.586
1167	A	0.906
1168	G	0.213
1169	C	0.093
1170	C	0.027
1171	C	0.093
1172	U	0.413
1173	A	0.786
1174	C	0.24
1175	C	0.479
1176	A	2.318
1177	G	0.24
1178	C	0.08
1179	A	0.226
1180	U	0.12
1181	U	0.067
1182	C	0
1183	U	0.013
1184	G	0.107
1185	G	0.173
1186	A	0.16
1187	C	0.04
1188	A	1.279
1189	U	0.866
1190	A	1.971
1191	A	2.091
1192	G	0.506
1193	A	0.333
1194	C	0.08
1195	A	0.16
1196	A	0.16
1197	G	0.107
1198	G	0.16
1199	A	0.746
1200	C	0.147

1201	C	0.186
1202	A	0.479
1203	A	0.639
1204	A	1.225
1205	G	0.719
1206	G	0.786
1207	A	0.932
1208	A	0.333
1209	C	0.133
1210	C	0.426
1211	C	0.12
1212	U	0.453
1213	U	0.44
1214	U	0.613
1215	A	0.586
1216	G	0.36
1217	A	0.386
1218	G	0.386
1219	A	0.373
1220	C	0.293
1221	U	0.32
1222	A	0.626
1223	U	0.226
1224	G	0.173
1225	U	0.107
1226	A	0.4
1227	G	0.32
1228	A	0.519
1229	C	1.572
1230	C	0.293
1231	G	0.946
1232	A	0.866
1233	U	0.719
1234	U	0.333
1235	C	0.133
1236	U	0.213
1237	A	0.466
1238	U	0.4
1239	A	0.759
1240	A	0.759
1241	A	0.799
1242	A	0.44
1243	C	0

1244	U	0.067
1245	C	0.053
1246	U	0.226
1247	A	0.613
1248	A	1.066
1249	G	0.266
1250	A	0.253
1251	G	0
1252	C	0.08
1253	C	0.2
1254	G	0.773
1255	A	0.892
1256	G	0.373
1257	C	0.28
1258	A	0.519
1259	A	0.519
1260	G	0
1261	C	0.013
1262	U	0.067
1263	U	0.293
1264	C	0.44
1265	A	1.438
1266	C	1.265
1267	A	1.172
1268	A	1.452
1269	G	0.773
1270	A	0.852
1271	G	1.185
1272	G	0.639
1273	U	0.746
1274	A	1.239
1275	A	1.159
1276	A	1.212
1277	A	0.999
1278	A	0.999
1279	A	0.986
1280	U	0.413
1281	U	0.333
1282	G	0.386
1283	G	0.626
1284	A	0.852
1285	U	0.746
1286	G	1.185

1287	A	1.145
1288	C	0.919
1289	A	0.999
1290	G	0.773
1291	A	1.159
1292	A	0.679
1293	A	0.666
1294	C	0.333
1295	C	0
1296	U	0.093
1297	U	0.306
1298	G	0.573
1299	U	0.626
1300	U	0.546
1301	G	0.639
1302	G	0.226
1303	U	0.226
1304	C	0.16
1305	C	0.266
1306	A	0.852
1307	A	0.906
1308	A	0.946
1309	A	0.479
1310	U	0.28
1311	G	0.173
1312	C	0.4
1313	G	0.479
1314	A	0.586
1315	A	0.639
1316	C	0.759
1317	C	0.519
1318	C	0.173
1319	A	1.478
1320	G	0.44
1321	A	0.253
1322	U	0.613
1323	U	1.652
1324	G	0.866
1325	U	0.999
1326	A	2.797
1327	A	1.505
1328	G	1.518
1329	A	0.2

1330	C	0.306
1331	U	0.386
1332	A	0.093
1333	U	0
1334	U	0.346
1335	U	0
1336	U	0.453
1337	A	0.573
1338	A	0.426
1339	A	0.559
1340	A	0.812
1341	G	1.159
1342	C	0.16
1343	A	0.586
1344	U	0.466
1345	U	0.173
1346	G	0.346
1347	G	0.706
1348	G	0.559
1349	A	0.479
1350	C	0.186
1351	C	0.147
1352	A	0.44
1353	G	0.786
1354	G	0.28
1355	A	0.453
1356	G	0.293
1357	C	0.147
1358	G	0.16
1359	A	0.24
1360	C	0.186
1361	A	0.253
1362	C	0.133
1363	U	0.266
1364	A	0.866
1365	G	0.24
1366	A	0.32
1367	A	0.373
1368	G	0.546
1369	A	0.733
1370	A	0.693
1371	A	0.559
1372	U	0.133

1373	G	0.866
1374	A	0.719
1375	U	0.2
1376	G	0.266
1377	A	0.293
1378	C	0.053
1379	A	0.08
1380	G	0.346
1381	C	0.107
1382	A	0.706
1383	U	0.12
1384	G	0.32
1385	U	0.36
1386	C	0.12
1387	A	0.4
1388	G	0.506
1389	G	0.639
1390	G	0.386
1391	A	0.613
1392	G	0.466
1393	U	0.4
1394	G	0.293
1395	G	0.253
1396	G	0.16
1397	G	0.346
1398	G	0.173
1399	G	0
1400	A	0.28
1401	C	0.28
1402	C	0.2
1403	C	0.24
1404	G	0.253
1405	G	0.16
1406	C	0.093
1407	C	0.453
1408	A	0.679
1409	U	0.266
1410	A	0.586
1411	A	0.919
1412	A	0.999
1413	G	0.666
1414	C	0
1415	A	0.453

1416	A	0.493
1417	G	0.892
1418	A	0.4
1419	G	0.053
1420	U	0.306
1421	U	0.013
1422	U	0.107
1423	U	0.213
1424	G	0.107
1425	G	0.027
1426	C	0.04
1427	U	0.346
1428	G	0.852
1429	A	0.826
1430	A	0.746
1431	G	0.852
1432	C	0
1433	A	0.293
1434	A	0.466
1435	U	0.28
1436	G	0.173
1437	A	0.253
1438	G	0.16
1439	C	0.147
1440	C	0.08
1441	A	0.186
1442	A	0.546
1443	G	0.32
1444	U	0.413
1445	A	0.346
1446	A	0.759
1447	C	0.386
1448	A	0.932
1449	A	0.919
1450	A	0.879
1451	U	0.613
1452	C	0.2
1453	C	0.306
1454	A	0.493
1455	G	0.253
1456	C	0.226
1457	U	0.24
1458	A	0.36

1459	C	0.253
1460	C	0.107
1461	A	0.053
1462	U	0.546
1463	A	0.426
1464	A	0.44
1465	U	0.426
1466	G	0.44
1467	A	0.559
1468	U	0.12
1469	A	0.799
1470	C	0.04
1471	A	0.586
1472	G	1.265
1473	A	0.986
1474	A	0.812
1475	A	0.16
1476	G	0.759
1477	G	0.919
1478	C	0
1479	A	0.533
1480	A	0.559
1481	U	0.24
1482	U	0.2
1483	U	0.013
1484	U	0.826
1485	A	0.746
1486	G	0.746
1487	G	0.679
1488	A	0.559
1489	A	0.293
1490	C	0.147
1491	C	0
1492	A	0.533
1493	A	0.586
1494	A	0.4
1495	G	0.599
1496	A	0.586
1497	A	0.613
1498	A	0.413
1499	G	0.479
1500	A	0.373
1501	C	0.28

1502	U	0.32
1503	G	0.44
1504	U	0.386
1505	U	0.32
1506	A	0.32
1507	A	0.32
1508	G	0.306
1509	U	0.147
1510	G	0.186
1511	U	0.107
1512	U	0.12
1513	U	0.253
1514	C	0.133
1515	A	0.186
1516	A	0.413
1517	U	0.746
1518	U	0.266
1519	G	0.147
1520	U	0.053
1521	G	0.293
1522	G	0.24
1523	C	0.413
1524	A	0.946
1525	A	0.986
1526	A	1.052
1527	G	0.799
1528	A	0.866
1529	A	1.079
1530	G	1.052
1531	G	0.786
1532	G	0.24
1533	C	0.573
1534	A	0.333
1535	C	0.493
1536	A	1.105
1537	U	1.092
1538	A	0.639
1539	G	0.253
1540	C	0
1541	C	0.16
1542	A	0.413
1543	A	0.586
1544	A	0.666

1545	A	1.079
1546	A	0.626
1547	U	0.373
1548	U	0.639
1549	G	1.145
1550	C	1.771
1551	A	1.452
1552	G	1.292
1553	G	0.306
1554	G	0.133
1555	C	0.16
1556	C	0.133
1557	C	0.226
1558	C	0.253
1559	U	1.026
1560	A	2.304
1561	G	1.279
1562	G	0.746
1563	A	0.773
1564	A	0.506
1565	A	1.092
1566	A	1.785
1567	A	1.172
1568	G	0.067
1569	G	0.12
1570	G	0.08
1571	C	0
1572	U	0.173
1573	G	0.16
1574	U	0.107
1575	U	0.067
1576	G	0.266
1577	G	0.2
1578	A	0.679
1579	A	0.999
1580	A	1.239
1581	U	0.413
1582	G	0.746
1583	U	0.679
1584	G	0.546
1585	G	0.413
1586	A	0.546
1587	A	0.613

1588	A	0.839
1589	G	0.759
1590	G	0.306
1591	A	0.226
1592	A	0.306
1593	G	0.346
1594	G	0.253
1595	A	0.346
1596	C	0.08
1597	A	1.066
1598	C	0.013
1599	C	0
1600	A	0.506
1601	A	1.865
1602	A	1.718
1603	U	0.946
1604	G	0.586
1605	A	0.373
1606	A	0.226
1607	A	0.466
1608	G	0.479
1609	A	0.173
1610	U	0
1611	U	0.133
1612	G	2.797
1613	U	1.039
1614	A	2.357
1615	C	0
1616	U	0.053
1617	G	0.12
1618	A	2.504
1619	G	2.038
1620	A	1.332
1621	G	1.652
1622	A	1.465
1623	C	0
1624	A	0.226
1625	G	0.147
1626	G	0.093
1627	C	0.173
1628	U	0.972
1629	A	1.505
1630	A	0.04

1631	U	0.346
1632	U	0.546
1633	U	0.426
1634	U	0.266
1635	U	0.28
1636	U	0.426
1637	A	1.105
1638	G	0.4
1639	G	0.093
1640	G	0.24
1641	A	0.679
1642	A	0.666
1643	G	0.453
1644	A	0.799
1645	U	0.479
1646	C	0
1647	U	0.08
1648	G	0.067
1649	G	0.04
1650	C	0.067
1651	C	0.053
1652	U	0.053
1653	U	0.04
1654	C	0.013
1655	C	0
1656	C	0
1657	A	1.652
1658	C	0.386
1659	A	1.079
1660	A	1.612
1661	G	0.013
1662	G	0.027
1663	G	0.08
1664	A	0.053
1665	A	0.027
1666	G	0.173
1667	G	0.107
1668	C	0.173
1669	C	0
1670	A	0
1671	G	0
1672	G	0.186
1673	G	0.666

1674	A	0.653
1675	A	0.773
1676	U	0.24
1677	U	0.186
1678	U	0.067
1679	U	0.107
1680	C	0
1681	U	0.107
1682	U	0.413
1683	C	0
1684	A	1.798
1685	G	2.264
1686	A	1.905
1687	G	1.039
1688	C	0
1689	A	0.826
1690	G	1.119
1691	A	0.919
1692	C	0.08
1693	C	0
1694	A	0.373
1695	G	0.679
1696	A	0.36
1697	G	0.546
1698	C	0
1699	C	0.04
1700	A	0.093
1701	A	0.107
1702	C	0
1703	A	0.08
1704	G	0.04
1705	C	0
1706	C	0
1707	C	0
1708	C	0.266
1709	A	1.438
1710	C	0.173
1711	C	0
1712	A	0.706
1713	G	0.986
1714	A	1.012
1715	A	0.812
1716	G	0.932

1717	A	1.012
1718	G	0.999
1719	A	1.012
1720	G	0.28
1721	C	0.027
1722	U	0.107
1723	U	0.293
1724	C	0.093
1725	A	0.812
1726	G	0.32
1727	G	0.24
1728	U	0.253
1729	U	0.453
1730	U	0.373
1731	G	0.147
1732	G	0.027
1733	G	0.013
1734	G	0.04
1735	A	0.2
1736	A	0.493
1737	G	0.426
1738	A	0.693
1739	G	0.226
1740	A	0.186
1741	C	0
1742	A	0.573
1743	A	2.158
1744	C	0.186
1745	A	0.373
1746	A	0.12
1747	C	0
1748	U	0.027
1749	C	0.013
1750	C	0.013
1751	C	0.013
1752	U	0.013
1753	C	0.04
1754	U	0.173
1755	C	0
1756	A	0.28
1757	G	0.173
1758	A	0.466
1759	A	1.012

1760	G	0.28
1761	C	0
1762	A	0.346
1763	G	0.213
1764	G	0.4
1765	A	0.599
1766	G	0.293
1767	C	0.013
1768	C	0.053
1769	G	0.386
1770	A	0.559
1771	U	0.226
1772	A	0.999
1773	G	0.946
1774	A	0.919
1775	C	0
1776	A	0.719
1777	A	1.159
1778	G	0.693
1779	G	0.906
1780	A	0.879
1781	A	0.919
1782	C	0.107
1783	U	0.053
1784	G	0.107
1785	U	0.107
1786	A	0.373
1787	U	0.04
1788	C	0.04
1789	C	0.08
1790	U	0.506
1791	U	0.453
1792	U	0.546
1793	A	0.852
1794	G	0.053
1795	C	0.027
1796	U	0.04
1797	U	0.04
1798	C	0.027
1799	C	0.013
1800	C	0.053
1801	U	0.519
1802	C	0.213

1803	A	0.773
1804	G	0.479
1805	A	0.626
1806	U	0.28
1807	C	0.067
1808	A	0.306
1809	C	0.093
1810	U	0.186
1811	C	0.027
1812	U	1.159
1813	U	0.426
1814	U	0.506
1815	G	0.373
1816	G	0.346
1817	C	0.107
1818	A	0.479
1819	G	0.479
1820	C	0.253
1821	G	0.506
1822	A	1.385
1823	C	0.013
1824	C	0.027
1825	C	0.04
1826	C	0.04
1827	U	0.067
1828	C	0.067
1829	G	0.093
1830	U	0.067
1831	C	0.04
1832	A	0.466
1833	C	0.04
1834	A	0.852
1835	A	0.786
1836	U	0.333
1837	A	0.826
1838	A	0.812
1839	A	1.612
1840	G	0.2
1841	A	0.32
1842	U	0.972
1843	A	0.426
1844	G	0
1845	G	0.013

1846	G	0.027
1847	G	0.053
1848	G	0
1849	G	0.053
1850	C	0.04
1851	A	0.693
1852	A	0.626
1853	U	0.373
1854	U	0.799
1855	A	0.892
1856	A	0.786
1857	A	0.826
1858	G	0.693
1859	G	0.546
1860	A	0.533
1861	A	0.666
1862	G	0.027
1863	C	0
1864	U	0.053
1865	C	0.08
1866	U	0.346
1867	A	0.586
1868	U	0.213
1869	U	0.533
1870	A	0.786
1871	G	0.546
1872	A	0.746
1873	U	0.346
1874	A	0.666
1875	C	0.186
1876	A	0.706
1877	G	0.253
1878	G	0.533
1879	A	0.879
1880	G	0.173
1881	C	0.04
1882	A	0.493
1883	G	0.4
1884	A	0.719
1885	U	0.346
1886	G	0.573
1887	A	0.639
1888	U	0.36

1889	A	0.679
1890	C	0.08
1891	A	0.586
1892	G	0.466
1893	U	0.32
1894	A	0.879
1895	U	0.28
1896	U	0.892
1897	A	1.372
1898	G	1.105
1899	A	1.252
1900	A	1.252
1901	G	1.172
1902	A	1.252
1903	A	0.919
1904	A	1.092
1905	U	0.679
1906	G	1.039
1907	A	0.946
1908	A	0.639
1909	U	0.599
1910	U	0.506
1911	U	0.226
1912	G	0.173
1913	C	0
1914	C	0.107
1915	A	0.333
1916	G	0.373
1917	G	0.44
1918	A	0.653
1919	A	0.626
1920	G	0.293
1921	A	0.559
1922	U	0.226
1923	G	0.679
1924	G	0.479
1925	A	0.453
1926	A	0.413
1927	A	0.346
1928	C	0.067
1929	C	0.107
1930	A	0.519
1931	A	0.586

1932	A	0.599
1933	A	0.599
1934	A	0.733
1935	U	0.466
1936	G	0.839
1937	A	1.012
1938	U	0.773
1939	A	1.105
1940	G	0.639
1941	G	0.426
1942	G	0.067
1943	G	0.027
1944	G	0.093
1945	A	0.2
1946	A	0.186
1947	U	0.306
1948	U	0.253
1949	G	0.133
1950	G	0.2
1951	A	0.213
1952	G	0.093
1953	G	0.053
1954	U	0.04
1955	U	0.013
1956	U	0.306
1957	U	0.639
1958	A	0.773
1959	U	0.24
1960	C	0.213
1961	A	0.826
1962	A	0.852
1963	A	0.972
1964	G	0.852
1965	U	0.333
1966	A	1.558
1967	A	1.758
1968	G	0.773
1969	A	0.666
1970	C	0.147
1971	A	0.626
1972	G	0.679
1973	U	0.333
1974	A	0.826

1975	U	0.16
1976	G	0.932
1977	A	1.105
1978	U	0.546
1979	C	0.28
1980	A	0.719
1981	G	0.653
1982	A	0.506
1983	U	0.559
1984	A	0.679
1985	C	0.293
1986	U	0.333
1987	C	0.107
1988	A	1.305
1989	U	0.879
1990	A	1.505
1991	G	0.479
1992	A	0.719
1993	A	0.586
1994	A	0.413
1995	U	0.133
1996	C	0.027
1997	U	0.173
1998	G	0.16
1999	C	0.226
2000	G	0.573
2001	G	0.839
2002	A	0.826
2003	C	0
2004	A	1.798
2005	U	0.4
2006	A	1.385
2007	A	1.039
2008	A	1.598
2009	G	0.666
2010	C	0.4
2011	U	0.519
2012	A	0.746
2013	U	1.425
2014	A	2.411
2015	G	0.08
2016	G	0
2017	U	0.133

2018	A	0.186
2019	C	0.213
2020	A	0.053
2021	G	0
2022	U	0.306
2023	A	0.44
2024	U	0.293
2025	U	0.453
2026	A	0.133
2027	G	0.067
2028	U	0.306
2029	A	0.133
2030	G	0.28
2031	G	0.16
2032	A	0.027
2033	C	0.186
2034	C	0.919
2035	U	1.412
2036	A	1.718
2037	C	0.12
2038	A	1.145
2039	C	0.573
2040	C	0.08
2041	U	0.373
2042	G	0.133
2043	U	0.12
2044	C	0.28
2045	A	0.36
2046	A	0.24
2047	C	0.027
2048	A	0.972
2049	U	0.786
2050	A	0.826
2051	A	0.506
2052	U	0.253
2053	U	0.386
2054	G	0.693
2055	G	1.159
2056	A	1.252
2057	A	0.879
2058	G	1.119
2059	A	1.745
2060	A	0.706

2061	A	0.32
2062	U	0.107
2063	C	0.013
2064	U	0.213
2065	G	0.067
2066	U	0.346
2067	U	0.4
2068	G	0.32
2069	A	0.186
2070	C	0.426
2071	U	1.505
2072	C	0
2073	A	1.079
2074	G	0.733
2075	A	0.519
2076	U	0.28
2077	U	0.293
2078	G	0.413
2079	G	0.04
2080	C	0
2081	U	0.24
2082	G	0.306
2083	C	0.093
2084	A	0.892
2085	C	0.16
2086	U	0.293
2087	U	0.626
2088	U	0.773
2089	A	1.425
2090	A	0.639
2091	A	0.679
2092	U	0.4
2093	U	0.24
2094	U	0.16
2095	U	0.147
2096	C	0.12
2097	C	0.2
2098	C	0.067
2099	A	0.653
2100	U	0.426
2101	U	0.852
2102	A	1.012
2103	G	0.08

2104	U	0.08
2105	C	0.147
2106	C	0.04
2107	U	0
2108	A	0.067
2109	U	0.013
2110	U	0.08
2111	G	0.093
2112	A	0.12
2113	G	0.147
2114	A	0.04
2115	C	0.253
2116	U	0.24
2117	G	0.173
2118	U	0.04
2119	A	0.107
2120	C	0
2121	C	0
2122	A	0.666
2123	G	1.026
2124	U	0.413
2125	A	1.185
2126	A	0.799
2127	A	0.759
2128	A	0.839
2129	U	0.546
2130	U	0.519
2131	A	1.092
2132	A	1.119
2133	A	1.119
2134	G	0.533
2135	C	0.12
2136	C	0
2137	A	0.852
2138	G	0.333
2139	G	0.573
2140	A	0.746
2141	A	0.533
2142	U	0.373
2143	G	0.32
2144	G	0.266
2145	A	0.333
2146	U	0.147

2147	G	0.067
2148	G	0
2149	C	0
2150	C	0.013
2151	C	0.08
2152	A	0.413
2153	A	0.453
2154	A	0.852
2155	A	0.812
2156	G	0.719
2157	U	0.533
2158	U	0.44
2159	A	0.986
2160	A	0.999
2161	A	1.265
2162	C	0.107
2163	A	0.133
2164	A	0.28
2165	U	0.213
2166	G	0.133
2167	G	0.12
2168	C	0
2169	C	0
2170	A	0.04
2171	U	0.067
2172	U	0.16
2173	G	0.493
2174	A	0.812
2175	C	0
2176	A	0.972
2177	G	0.852
2178	A	1.265
2179	A	1.705
2180	G	0.04
2181	A	1.265
2182	A	0.759
2183	A	0.839
2184	A	0.746
2185	A	0.719
2186	A	0.959
2187	U	0.506
2188	A	1.279
2189	A	0.906

2190	A	1.026
2191	A	0.906
2192	G	0.786
2193	C	0.013
2194	A	0.773
2195	U	0.493
2196	U	0.639
2197	A	1.412
2198	G	0.866
2199	U	0.599
2200	A	1.359
2201	G	0.466
2202	A	0.466
2203	A	0.466
2204	A	0.36
2205	U	0.133
2206	U	0.28
2207	U	0.253
2208	G	0.413
2209	U	0.466
2210	A	0.666
2211	C	0.12
2212	A	0.719
2213	G	0.946
2214	A	0.999
2215	A	0.639
2216	A	0.653
2217	U	0.266
2218	G	0.693
2219	G	0.972
2220	A	1.105
2221	A	0.946
2222	A	1.052
2223	A	0.959
2224	G	0.919
2225	G	0.866
2226	A	0.879
2227	A	0.866
2228	G	0.693
2229	G	0.733
2230	A	0.946
2231	A	0.892
2232	A	0.613

2233	A	0.599
2234	A	0.426
2235	U	0.173
2236	U	0.16
2237	U	0.253
2238	C	0.253
2239	A	1.945
2240	A	1.252
2241	A	0.759
2242	A	0.866
2243	A	1.465
2244	U	0.173
2245	U	0.053
2246	G	0.053
2247	G	0.16
2248	G	0.08
2249	C	0.253
2250	C	0.013
2251	U	0.04
2252	G	0.506
2253	A	0.426
2254	A	0.386
2255	A	0.373
2256	A	0.453
2257	U	0.173
2258	C	0.093
2259	C	0.12
2260	A	0.932
2261	U	0.546
2262	A	1.412
2263	C	0
2264	A	0.546
2265	A	0.626
2266	U	0.586
2267	A	0.546
2268	C	0.027
2269	U	0.067
2270	C	0.013
2271	C	0.266
2272	A	0.333
2273	G	0.12
2274	U	0.12
2275	A	0.053

2276	U	0
2277	U	0.293
2278	U	0.093
2279	G	0.12
2280	C	0.053
2281	C	0.067
2282	A	0.999
2283	U	0.786
2284	A	1.066
2285	A	0.719
2286	A	0.879
2287	G	0.293
2288	A	1.012
2289	A	0.613
2290	A	0.812
2291	A	0.719
2292	A	1.026
2293	A	2.318
2294	G	0.333
2295	A	0.293
2296	C	0.107
2297	A	0.226
2298	G	0.12
2299	U	0.067
2300	A	0.12
2301	C	0.013
2302	U	0.746
2303	A	0.866
2304	A	1.652
2305	A	2.038
2306	U	0.479
2307	G	0.053
2308	G	0.12
2309	A	0.506
2310	G	0.12
2311	A	0.906
2312	A	1.105
2313	A	0.759
2314	A	0.386
2315	U	0.24
2316	U	0.773
2317	A	2.611
2318	G	0.32

2319	U	0.16
2320	A	0.386
2321	G	0.28
2322	A	0.373
2323	U	0.16
2324	U	0.186
2325	U	0.226
2326	C	0.067
2327	A	1.119
2328	G	0.426
2329	A	0.666
2330	G	0.666
2331	A	0.746
2332	A	0.666
2333	C	0.053
2334	U	0.28
2335	U	0.466
2336	A	0.959
2337	A	1.305
2338	U	0.466
2339	A	0.799
2340	A	0.666
2341	G	0.826
2342	A	0.599
2343	G	0.4
2344	A	0.4
2345	A	0.306
2346	C	0.12
2347	U	0.24
2348	C	0.12
2349	A	1.159
2350	A	0.972
2351	G	0.493
2352	A	0.506
2353	U	0.213
2354	U	0.16
2355	U	0.16
2356	C	0.04
2357	U	0.133
2358	G	0.266
2359	G	0.306
2360	G	0.533
2361	A	0.693

2362	A	0.506
2363	G	0.12
2364	U	0.147
2365	U	0.2
2366	C	0.12
2367	A	0.826
2368	A	0.693
2369	U	0.373
2370	U	0.493
2371	A	1.079
2372	G	0.906
2373	G	0.679
2374	A	0.453
2375	A	0.733
2376	U	0.773
2377	A	0.932
2378	C	0.12
2379	C	0.226
2380	A	0.306
2381	C	0.04
2382	A	0.28
2383	U	0.067
2384	C	0.013
2385	C	0.04
2386	U	0.173
2387	G	0.36
2388	C	0.16
2389	A	0.799
2390	G	0.733
2391	G	0.333
2392	G	0.08
2393	U	0.053
2394	U	0.493
2395	A	1.145
2396	A	1.705
2397	A	0.653
2398	A	0.013
2399	C	0.266
2400	A	1.612
2401	G	0.027
2402	A	1.079
2403	A	1.092
2404	A	1.172

2405	A	0.959
2406	A	0.852
2407	A	0.959
2408	U	0.546
2409	C	0.32
2410	A	0.879
2411	G	0.932
2412	U	0.253
2413	A	0.719
2414	A	0.599
2415	C	0.44
2416	A	1.092
2417	G	0.866
2418	U	0.493
2419	A	0.413
2420	C	0.093
2421	U	0.027
2422	G	0.04
2423	G	0.093
2424	A	0.266
2425	U	0.04
2426	G	0.186
2427	U	0.093
2428	G	0.107
2429	G	1.052
2430	G	0.107
2431	C	0.107
2432	G	0.426
2433	A	0.466
2434	U	0.147
2435	G	0.373
2436	C	0.133
2437	A	0.586
2438	U	0.2
2439	A	0.706
2440	U	0.266
2441	U	0.466
2442	U	0.466
2443	U	0.4
2444	U	0.4
2445	C	0.28
2446	A	0.466
2447	G	0.36

2448	U	0.173
2449	U	0.147
2450	C	0.067
2451	C	0.027
2452	C	0.053
2453	U	0.506
2454	U	0.972
2455	A	1.438
2456	G	0.719
2457	A	1.199
2458	U	0.519
2459	A	1.159
2460	A	0.719
2461	A	1.026
2462	G	0.999
2463	A	0.693
2464	C	0.147
2465	U	0.373
2466	U	0.533
2467	C	0.28
2468	A	0.932
2469	G	0.44
2470	G	0.32
2471	A	0.373
2472	A	0.426
2473	G	0.506
2474	U	0.266
2475	A	0.666
2476	U	0.253
2477	A	0.479
2478	C	0.093
2479	U	0.213
2480	G	0.28
2481	C	0.213
2482	A	0.466
2483	U	0.12
2484	U	0.346
2485	U	0.879
2486	A	0.666
2487	C	0.16
2488	C	0.12
2489	A	0.466
2490	U	0.346

2491	A	0.799
2492	C	0.04
2493	C	0.16
2494	U	0.213
2495	A	0.599
2496	G	0.333
2497	U	0.133
2498	A	1.039
2499	U	0.666
2500	A	1.785
2501	A	1.132
2502	A	1.132
2503	C	0.266
2504	A	1.319
2505	A	0.919
2506	U	0.533
2507	G	1.359
2508	A	1.066
2509	G	0.746
2510	A	0.666
2511	C	0.373
2512	A	0.28
2513	C	0.053
2514	C	0.107
2515	A	0.466
2516	G	0.28
2517	G	0.293
2518	G	0.373
2519	A	0.573
2520	U	0.519
2521	U	1.199
2522	A	1.612
2523	G	0.892
2524	A	1.372
2525	U	0.599
2526	A	1.305
2527	U	0.533
2528	C	0.613
2529	A	0.986
2530	G	0.733
2531	U	0.4
2532	A	0.799
2533	C	0.4

2534	A	0.812
2535	A	0.892
2536	U	0.719
2537	G	0.4
2538	U	0.253
2539	G	0.053
2540	C	0
2541	U	0.173
2542	U	0.293
2543	C	0.346
2544	C	0.44
2545	A	0.573
2546	C	0.333
2547	A	0.812
2548	G	0.453
2549	G	0.306
2550	G	0.346
2551	A	0.799
2552	U	0.24
2553	G	0.413
2554	G	0.32
2555	A	0.826
2556	A	1.026
2557	A	1.012
2558	G	0.493
2559	G	0.493
2560	A	0.693
2561	U	0.28
2562	C	0.28
2563	A	0.906
2564	C	0.12
2565	C	0.147
2566	A	0.613
2567	G	0.613
2568	C	0.2
2569	A	0.999
2570	A	1.518
2571	U	0.852
2572	A	0.866
2573	U	0.373
2574	U	0.186
2575	C	0.067
2576	C	0.067

2577	A	0.4
2578	G	0.44
2579	U	0.2
2580	G	0.36
2581	U	0.24
2582	A	0.453
2583	G	0.293
2584	C	0.147
2585	A	0.826
2586	U	0.479
2587	G	1.079
2588	A	1.172
2589	C	0.533
2590	A	1.545
2591	A	1.132
2592	A	0.879
2593	A	1.105
2594	A	0.972
2595	U	0.293
2596	C	0.08
2597	U	0.293
2598	U	0.559
2599	A	1.105
2600	G	0.812
2601	A	0.919
2602	G	0.04
2603	C	0.013
2604	C	0.133
2605	U	0.067
2606	U	0.266
2607	U	1.652
2608	U	2.051
2609	A	2.504
2610	G	0.466
2611	A	1.252
2612	A	1.105
2613	A	1.185
2614	A	0.879
2615	C	0.226
2616	A	1.359
2617	A	1.478
2618	A	1.518
2619	A	0.32

2620	U	0.013
2621	C	0.133
2622	C	0.133
2623	A	1.518
2624	G	2.877
2625	A	0.559
2626	C	0.04
2627	A	0.866
2628	U	0.453
2629	A	0.186
2630	G	0.013
2631	U	0
2632	C	0.04
2633	A	0.013
2634	U	0.067
2635	C	0.013
2636	U	0
2637	A	0.107
2638	U	0.067
2639	C	1.505
2640	A	1.172
2641	A	1.279
2642	U	0.373
2643	A	1.452
2644	C	0.346
2645	A	0.16
2646	U	0.147
2647	G	0
2648	G	0.067
2649	A	0.16
2650	U	0.133
2651	G	0.013
2652	A	0.08
2653	U	0.253
2654	U	0.506
2655	U	0.932
2656	G	1.292
2657	U	0.133
2658	A	0.919
2659	U	0.107
2660	G	0.12
2661	U	0.253
2662	A	0.999

2663	G	0.186
2664	G	0.053
2665	A	0.107
2666	U	0
2667	C	0
2668	U	0.08
2669	G	0.226
2670	A	0.293
2671	C	0
2672	U	0.08
2673	U	0.306
2674	A	1.465
2675	G	0.892
2676	A	0.892
2677	A	0.626
2678	A	0.626
2679	U	0.373
2680	A	0.759
2681	G	0.626
2682	G	0.04
2683	G	0.067
2684	C	0
2685	A	0.213
2686	G	0.266
2687	C	0.186
2688	A	0.719
2689	U	0.453
2690	A	1.718
2691	G	0.946
2692	A	1.039
2693	A	0.586
2694	C	0.186
2695	A	0.932
2696	A	0.919
2697	A	0.852
2698	A	0.919
2699	A	1.052
2700	U	0.653
2701	A	0.932
2702	G	0.493
2703	A	0.932
2704	G	0.466
2705	G	0.413

2706	A	0.493
2707	A	0.479
2708	C	0
2709	U	0
2710	G	0.213
2711	A	0.093
2712	G	0.4
2713	A	0.839
2714	C	0
2715	A	0.719
2716	A	0.653
2717	C	0.107
2718	A	0.826
2719	U	0.226
2720	C	0.293
2721	U	0.226
2722	G	0.16
2723	U	0.293
2724	U	0.4
2725	G	1.332
2726	A	1.545
2727	G	0.16
2728	G	0
2729	U	0.04
2730	G	0.013
2731	G	0
2732	G	0
2733	G	0.067
2734	A	0.639
2735	U	0.653
2736	U	0.413
2737	U	0.946
2738	A	2.597
2739	C	0.013
2740	C	0
2741	A	0.573
2742	C	0
2743	A	0.2
2744	C	0.013
2745	C	0.147
2746	A	0.826
2747	G	0.733
2748	A	0.719

2749	C	0
2750	A	0.906
2751	A	0.866
2752	A	0.746
2753	A	0.533
2754	A	0.999
2755	A	0.706
2756	C	0.08
2757	A	1.026
2758	U	0.479
2759	C	0.133
2760	A	0.852
2761	G	0.666
2762	A	0.959
2763	A	1.265
2764	A	1.119
2765	G	0.666
2766	A	0.4
2767	A	0.426
2768	C	0.107
2769	C	0
2770	U	0
2771	C	0.093
2772	C	0.053
2773	A	0.08
2774	U	0.16
2775	U	0.04
2776	C	0.027
2777	C	0
2778	U	0.04
2779	U	1.319
2780	U	0.666
2781	G	0.466
2782	G	0.067
2783	A	0.107
2784	U	0
2785	G	0.12
2786	G	0
2787	G	0.107
2788	U	0.107
2789	U	1.612
2790	A	1.105
2791	U	0.32

2792	G	0.999
2793	A	0.759
2794	A	0.213
2795	C	0.08
2796	U	0.426
2797	C	0.08
2798	C	0
2799	A	0.053
2800	U	0.08
2801	C	0.027
2802	C	0
2803	U	0.759
2804	G	0.906
2805	A	1.199
2806	U	0.626
2807	A	0.986
2808	A	0.919
2809	A	1.079
2810	U	0.693
2811	G	0.826
2812	G	0.08
2813	A	0.36
2814	C	0.107
2815	A	0.027
2816	G	0
2817	U	0.053
2818	A	0.107
2819	C	0.013
2820	A	0.266
2821	G	0.16
2822	C	0.053
2823	C	0.186
2824	U	0.519
2825	A	1.105
2826	U	0.786
2827	A	1.545
2828	G	0.506
2829	U	0.12
2830	G	0.013
2831	C	0.027
2832	U	0.053
2833	G	0.027
2834	C	0.013

2835	C	0.013
2836	A	0.599
2837	G	0.932
2838	A	0.986
2839	A	0.839
2840	A	1.012
2841	A	1.012
2842	G	0.892
2843	G	0.586
2844	A	0.653
2845	C	0.333
2846	A	0
2847	G	0
2848	C	0.107
2849	U	0
2850	G	0.213
2851	G	0.133
2852	A	0.386
2853	C	0
2854	U	0.08
2855	G	0.053
2856	U	0.12
2857	C	0.08
2858	A	1.039
2859	A	0.972
2860	U	0.44
2861	G	1.052
2862	A	0.786
2863	C	0.12
2864	A	1.092
2865	U	0.386
2866	A	0.972
2867	C	0.093
2868	A	1.092
2869	G	0.892
2870	A	1.092
2871	A	1.185
2872	A	0.879
2873	U	0.519
2874	U	0.533
2875	A	0.413
2876	G	0.413
2877	U	0.173

2878	G	0.24
2879	G	0.213
2880	G	0.107
2881	A	0.373
2882	A	0.533
2883	A	0.559
2884	A	0.626
2885	U	0.533
2886	U	0.653
2887	G	1.212
2888	A	1.212
2889	A	0.573
2890	U	0.506
2891	U	0.4
2892	G	0.493
2893	G	0.573
2894	G	0.333
2895	C	0
2896	A	0.426
2897	A	0.453
2898	G	0.226
2899	U	0.226
2900	C	0.2
2901	A	0.573
2902	G	0.519
2903	A	0.573
2904	U	0.28
2905	U	0.453
2906	U	0.573
2907	A	1.372
2908	U	0.36
2909	G	0.333
2910	C	0.107
2911	A	0.466
2912	G	0.826
2913	G	0.266
2914	G	0.44
2915	A	0.599
2916	U	0.573
2917	U	0.999
2918	A	1.412
2919	A	1.252
2920	A	1.345

2921	G	1.052
2922	U	0.786
2923	A	1.039
2924	A	0.812
2925	G	0.559
2926	G	0.426
2927	C	0.24
2928	A	0.826
2929	A	0.892
2930	U	0.653
2931	U	0.719
2932	A	1.319
2933	U	0.44
2934	G	1.145
2935	U	0.639
2936	A	1.372
2937	A	0.986
2938	A	0.453
2939	C	0.133
2940	U	0.107
2941	U	0.067
2942	C	0.08
2943	U	0.4
2944	U	0.546
2945	A	0.613
2946	G	0.226
2947	G	0.067
2948	G	0.013
2949	G	0.306
2950	A	0.519
2951	A	0.719
2952	C	0.053
2953	C	0.266
2954	A	0.733
2955	A	0.932
2956	A	0.799
2957	G	0.506
2958	C	0.373
2959	A	0.639
2960	C	0.093
2961	U	0.426
2962	A	0.746
2963	A	1.052

2964	C	0.346
2965	A	0.879
2966	G	0.799
2967	A	0.839
2968	A	0.839
2969	G	0.666
2970	U	0.426
2971	A	0.919
2972	G	0.173
2973	U	0.36
2974	A	0.213
2975	C	0.067
2976	C	0
2977	A	0
2978	C	0.346
2979	U	0.067
2980	A	0.266
2981	A	0.479
2982	C	0.799
2983	A	0.519
2984	G	1.425
2985	A	0.733
2986	A	0.852
2987	G	0.946
2988	A	0.986
2989	A	1.052
2990	G	1.159
2991	C	0.226
2992	A	0.573
2993	G	0.866
2994	A	0.733
2995	G	0.28
2996	C	0.186
2997	U	0.333
2998	A	0.812
2999	G	1.265
3000	A	0.986
3001	A	0.773
3002	C	0.133
3003	U	0.04
3004	G	0.173
3005	G	0.32
3006	C	0.226

3007	A	1.225
3008	G	0.546
3009	A	1.105
3010	A	0.972
3011	A	0.999
3012	A	0.839
3013	C	0.093
3014	A	0.839
3015	G	0.266
3016	G	0.373
3017	G	0.626
3018	A	1.079
3019	G	0.346
3020	A	0.626
3021	U	0.067
3022	U	0.08
3023	C	0.306
3024	U	1.066
3025	A	1.359
3026	A	0.906
3027	A	0.733
3028	A	0.972
3029	G	0.506
3030	A	0.613
3031	A	0.413
3032	C	0.333
3033	C	0.213
3034	G	0.333
3035	G	0.533
3036	U	0.546
3037	A	0.826
3038	C	0.786
3039	A	0.639
3040	U	0.613
3041	G	0.173
3042	G	0.32
3043	A	0.173
3044	G	0.173
3045	U	0
3046	G	0.599
3047	U	0.426
3048	A	0.573
3049	U	0.107

3050	U	0.24
3051	A	0.799
3052	U	0.373
3053	G	0.306
3054	A	0.586
3055	C	0.266
3056	C	0.173
3057	C	0.12
3058	A	0.16
3059	U	0.147
3060	C	0.693
3061	A	2.224
3062	A	0.466
3063	A	0.626
3064	A	0.679
3065	G	0.733
3066	A	0.613
3067	C	0
3068	U	0.226
3069	U	0.373
3070	A	0.839
3071	A	0.639
3072	U	0.306
3073	A	0.706
3074	G	0.147
3075	C	0
3076	A	0.386
3077	G	0.666
3078	A	0.639
3079	A	0.493
3080	A	0.826
3081	U	0.413
3082	A	0.586
3083	C	0
3084	A	0.466
3085	G	0.346
3086	A	0.666
3087	A	0.546
3088	G	0.453
3089	C	0.093
3090	A	0
3091	G	0.386
3092	G	0.28

3093	G	0.213
3094	G	0.253
3095	C	0.093
3096	A	0.786
3097	A	1.012
3098	G	0.44
3099	G	0.266
3100	C	0.16
3101	C	0.213
3102	A	0.533
3103	A	0.493
3104	U	0.16
3105	G	0.386
3106	G	0.479
3107	A	0.573
3108	C	0.04
3109	A	0.799
3110	U	0.266
3111	A	0.866
3112	U	0.24
3113	C	0.093
3114	A	0.599
3115	A	0.693
3116	A	0.599
3117	U	0.373
3118	U	0.559
3119	U	0.333
3120	A	0.892
3121	U	0.4
3122	C	0.107
3123	A	0.746
3124	A	0.986
3125	G	1.225
3126	A	1.665
3127	G	0.266
3128	C	0.226
3129	C	0
3130	A	0.346
3131	U	0.213
3132	U	0.653
3133	U	0.946
3134	A	1.225
3135	A	0.879

3136	A	0.879
3137	A	0.773
3138	A	0.799
3139	U	0.346
3140	C	0.2
3141	U	0.346
3142	G	0.346
3143	A	0.719
3144	A	0.852
3145	A	0.773
3146	A	0.693
3147	C	0.16
3148	A	0.586
3149	G	0.4
3150	G	0.679
3151	A	0.559
3152	A	0.706
3153	A	0.733
3154	A	0.533
3155	U	0.386
3156	A	0.693
3157	U	0.213
3158	G	0.226
3159	C	0.08
3160	A	0.719
3161	A	0.759
3162	G	0.786
3163	A	0.693
3164	A	0.786
3165	U	0.519
3166	G	0.573
3167	A	0.466
3168	A	0.506
3169	G	0.759
3170	G	0.453
3171	G	0.2
3172	U	0.2
3173	G	0.213
3174	C	0.013
3175	C	0.093
3176	C	0.12
3177	A	0.613
3178	C	0.107

3179	A	0.519
3180	C	0.04
3181	U	0.866
3182	A	1.572
3183	A	1.279
3184	U	0.586
3185	G	0.919
3186	A	1.252
3187	U	0.426
3188	G	0.866
3189	U	0.226
3190	G	0.466
3191	A	0.666
3192	A	0.719
3193	A	0.559
3194	C	0.12
3195	A	0.626
3196	A	0.693
3197	U	0.466
3198	U	0.626
3199	A	0.653
3200	A	0.653
3201	C	0.293
3202	A	0.746
3203	G	0.666
3204	A	1.438
3205	G	0.266
3206	G	0.306
3207	C	0.186
3208	A	0.626
3209	G	0.906
3210	U	0.733
3211	A	1.145
3212	C	0.173
3213	A	1.145
3214	A	0.986
3215	A	1.185
3216	A	0.839
3217	A	0.999
3218	A	0.972
3219	U	0.906
3220	A	1.438
3221	G	0.133

3222	C	0.067
3223	C	0.147
3224	A	0.626
3225	C	0.373
3226	A	1.412
3227	G	1.145
3228	A	1.305
3229	A	1.319
3230	A	1.345
3231	G	0.679
3232	C	0.28
3233	A	1.026
3234	U	0.626
3235	A	1.252
3236	G	1.292
3237	U	1.012
3238	A	1.692
3239	A	1.425
3240	U	0.693
3241	A	0.639
3242	U	0.266
3243	G	0.16
3244	G	0.08
3245	G	0.093
3246	G	0.413
3247	A	0.959
3248	A	1.438
3249	A	1.505
3250	G	1.052
3251	A	0.719
3252	C	0.2
3253	U	0.24
3254	C	0.12
3255	C	0.133
3256	U	0.653
3257	A	1.532
3258	A	0.972
3259	A	1.026
3260	U	0.653
3261	U	0.946
3262	U	1.159
3263	A	1.545
3264	A	1.066

3265	A	1.305
3266	U	0.826
3267	U	1.012
3268	A	0.892
3269	C	0.013
3270	C	0.04
3271	C	0.027
3272	A	0.226
3273	U	0.253
3274	A	1.239
3275	C	0.733
3276	A	1.279
3277	A	0.999
3278	A	1.385
3279	A	1.398
3280	G	1.119
3281	G	1.305
3282	A	1.185
3283	A	1.066
3284	A	0.932
3285	C	0.559
3286	A	0.653
3287	U	0.28
3288	G	0.067
3289	G	0.067
3290	G	0.213
3291	A	0.453
3292	A	0.599
3293	G	0.4
3294	C	0.293
3295	A	0.866
3296	U	0.386
3297	G	0.32
3298	G	0.306
3299	U	0.253
3300	G	0.493
3301	G	0.493
3302	A	0.679
3303	C	0.32
3304	A	0.879
3305	G	0.972
3306	A	1.212
3307	G	1.252

3308	U	0.653
3309	A	0.599
3310	U	0.386
3311	U	0.533
3312	G	0.253
3313	G	0.16
3314	C	0.666
3315	A	0.746
3316	A	0.519
3317	G	0.12
3318	C	0.226
3319	C	0.919
3320	A	0
3321	C	0.013
3322	C	0.067
3323	U	0.453
3324	G	0.506
3325	G	0.839
3326	A	1.438
3327	U	0.453
3328	U	0.559
3329	C	0.346
3330	C	0.093
3331	U	0.067
3332	G	0.186
3333	A	0.173
3334	G	0.2
3335	U	0.147
3336	G	0.04
3337	G	0.067
3338	G	0.266
3339	A	0.08
3340	G	0.107
3341	U	0.28
3342	U	0.16
3343	U	0.36
3344	G	0.08
3345	U	0.04
3346	C	0.12
3347	A	0.533
3348	A	0.653
3349	U	0.32
3350	A	0.306

3351	C	0.04
3352	C	0.04
3353	C	0
3354	C	0.04
3355	U	0.426
3356	C	0.173
3357	C	0.093
3358	C	0.12
3359	U	0.746
3360	U	1.265
3361	A	2.211
3362	G	1.305
3363	U	0.4
3364	G	0.626
3365	A	0.826
3366	A	1.585
3367	G	0.453
3368	U	0.28
3369	U	0.626
3370	A	1.319
3371	U	0.16
3372	G	0.186
3373	G	0.293
3374	U	0.333
3375	A	0.666
3376	C	0.093
3377	C	0
3378	A	0.413
3379	G	0.333
3380	U	0.053
3381	U	0
3382	A	1.332
3383	G	0.826
3384	A	1.172
3385	G	0.959
3386	A	0.786
3387	A	0.333
3388	A	1.239
3389	G	1.692
3390	A	1.865
3391	A	0.679
3392	C	0.16
3393	C	0.027

3394	C	0
3395	A	0.386
3396	U	0.493
3397	A	1.452
3398	A	0.44
3399	U	0
3400	A	0.533
3401	G	0.44
3402	G	0.04
3403	A	0.599
3404	G	0.24
3405	C	0
3406	A	1.212
3407	G	0.586
3408	A	0.679
3409	A	0.946
3410	A	0
3411	C	0
3412	U	0.213
3413	U	0.186
3414	U	0.346
3415	C	0
3416	U	0.04
3417	A	0.466
3418	U	0.16
3419	G	0.639
3420	U	0
3421	A	2.517
3422	G	1.718
3423	A	0.12
3424	U	0.04
3425	G	0.2
3426	G	0.173
3427	G	0.346
3428	G	0.12
3429	C	0
3430	A	0.892
3431	G	0.586
3432	C	0.173
3433	C	0
3434	A	0.36
3435	A	0.972
3436	U	0.107

3437	A	1.026
3438	G	0.107
3439	G	0.32
3440	G	0.32
3441	A	0.44
3442	A	0.626
3443	A	0.799
3444	C	0.293
3445	U	0.613
3446	A	1.265
3447	A	1.039
3448	A	0.759
3449	U	0.4
3450	U	0.2
3451	A	1.185
3452	G	1.452
3453	G	0.986
3454	A	1.345
3455	A	0.986
3456	A	0.839
3457	A	1.279
3458	G	0.453
3459	C	0
3460	A	0.559
3461	G	0.546
3462	G	0.706
3463	A	0.972
3464	U	0.306
3465	A	1.132
3466	U	0.36
3467	G	0.4
3468	U	0.133
3469	A	0.613
3470	A	0.719
3471	C	0.013
3472	U	0.599
3473	G	1.412
3474	A	1.385
3475	C	0
3476	A	0.693
3477	G	0.533
3478	A	0.986
3479	G	0.879

3480	G	0.906
3481	A	0.759
3482	A	1.092
3483	G	0.906
3484	A	1.345
3485	C	0.107
3486	A	1.279
3487	A	1.145
3488	A	1.478
3489	A	1.332
3490	A	1.998
3491	G	0.733
3492	U	0.293
3493	U	0.373
3494	G	0.147
3495	U	0
3496	C	0.08
3497	C	0
3498	C	0.067
3499	C	0.16
3500	C	0.093
3501	U	0.44
3502	A	1.305
3503	A	0.972
3504	C	0.519
3505	G	0.906
3506	G	0.759
3507	A	0.773
3508	C	0.213
3509	A	0.693
3510	C	0
3511	A	0.812
3512	A	0.839
3513	C	0.147
3514	A	1.092
3515	A	0.786
3516	A	0.879
3517	U	0.506
3518	C	0.173
3519	A	1.319
3520	G	0.959
3521	A	0.906
3522	A	1.585

3523	G	0.426
3524	A	0.533
3525	C	0.16
3526	U	0.173
3527	G	0.16
3528	A	0.28
3529	G	0.44
3530	U	0.32
3531	U	0.44
3532	A	0.999
3533	C	0.107
3534	A	0.866
3535	A	0.852
3536	G	0.559
3537	C	0
3538	A	0.599
3539	A	0.719
3540	U	0.466
3541	U	0.586
3542	C	0.266
3543	A	0.986
3544	U	0.373
3545	C	0
3546	U	0.16
3547	A	0.373
3548	G	0.679
3549	C	0.027
3550	U	0.4
3551	U	0.186
3552	U	0.573
3553	G	0.613
3554	C	0.533
3555	A	0.32
3556	G	0.932
3557	G	1.039
3558	A	0.493
3559	U	0.559
3560	U	0.466
3561	C	0.4
3562	G	0.053
3563	G	0.879
3564	G	0.839
3565	A	0.44

3566	U	0.706
3567	U	0.346
3568	A	0.906
3569	G	1.105
3570	A	0.999
3571	A	1.398
3572	G	1.185
3573	U	1.066
3574	A	1.359
3575	A	0.852
3576	A	0.826
3577	C	0.067
3578	A	0.999
3579	U	0.333
3580	A	1.665
3581	G	0.919
3582	U	0.293
3583	G	0.16
3584	A	0.266
3585	C	0.32
3586	A	0.546
3587	G	0.253
3588	A	0.386
3589	C	0.067
3590	U	0.12
3591	C	0.24
3592	A	0.892
3593	C	0.067
3594	A	0.626
3595	A	0.679
3596	U	0.386
3597	A	1.705
3598	U	0.266
3599	G	0.546
3600	C	0.147
3601	A	0.719
3602	U	0.213
3603	U	0.266
3604	G	0.493
3605	G	0.44
3606	G	0.506
3607	A	0.506
3608	A	0.693

3609	U	0.186
3610	C	0.067
3611	A	0.626
3612	U	0.28
3613	U	0.28
3614	C	0.04
3615	A	0.959
3616	A	0.773
3617	G	0.44
3618	C	0.16
3619	A	0.16
3620	C	0.107
3621	A	0.839
3622	A	0.639
3623	C	0.093
3624	C	0.04
3625	A	0.479
3626	G	0.626
3627	A	1.558
3628	U	0.32
3629	A	0.919
3630	A	0.946
3631	G	0.493
3632	A	0.546
3633	G	0.373
3634	U	0.093
3635	G	0.28
3636	A	0.413
3637	A	0.613
3638	U	0.413
3639	C	0.12
3640	A	1.119
3641	G	0.679
3642	A	0.733
3643	G	0.107
3644	U	0.4
3645	U	0.573
3646	A	0.693
3647	G	0.266
3648	U	0.12
3649	C	0.027
3650	A	0.453
3651	G	0.386

3652	U	0.293
3653	C	0.067
3654	A	0.693
3655	A	0.719
3656	A	1.105
3657	U	0.253
3658	A	0.599
3659	A	0.546
3660	U	0.186
3661	A	0.759
3662	G	0.759
3663	A	0.666
3664	G	0.346
3665	C	0.053
3666	A	0.453
3667	G	0.413
3668	U	0.373
3669	U	0.653
3670	A	1.319
3671	A	1.598
3672	U	0.053
3673	A	1.505
3674	A	0.959
3675	A	1.026
3676	A	0.866
3677	A	0.932
3678	A	1.172
3679	G	0.799
3680	G	0.906
3681	A	0.892
3682	A	0.573
3683	A	0.666
3684	A	0.759
3685	A	1.119
3686	G	0.213
3687	U	0.173
3688	C	0.4
3689	U	0.36
3690	A	0.786
3691	C	0.013
3692	C	0.12
3693	U	0.147
3694	G	0.107

3695	G	0.213
3696	C	0.133
3697	A	0.746
3698	U	0.573
3699	G	0.639
3700	G	0.147
3701	G	0.693
3702	U	0.32
3703	A	0.546
3704	C	0.053
3705	C	0
3706	A	0.213
3707	G	0.226
3708	C	0
3709	A	0.147
3710	C	0
3711	A	1.092
3712	C	0
3713	A	0.826
3714	A	0.892
3715	A	0.986
3716	G	0.972
3717	G	0.892
3718	A	0.826
3719	A	0.546
3720	U	0.479
3721	U	0.4
3722	G	0.253
3723	G	0.626
3724	A	0.666
3725	G	0.373
3726	G	0.493
3727	A	0.879
3728	A	1.052
3729	A	1.119
3730	U	0.453
3731	G	0.999
3732	A	0.812
3733	A	0.946
3734	C	0
3735	A	0.759
3736	A	0.653
3737	G	0.746

3738	U	0.44
3739	A	1.359
3740	G	0.852
3741	A	1.159
3742	U	0.253
3743	G	1.465
3744	G	1.252
3745	G	0.946
3746	U	0.613
3747	U	0.413
3748	G	0.599
3749	G	0.479
3750	U	0.333
3751	C	0.107
3752	A	1.252
3753	G	0.533
3754	U	0.12
3755	G	0.16
3756	C	0.24
3757	U	0.213
3758	G	0.093
3759	G	0.147
3760	A	0.493
3761	A	0.44
3762	U	0.413
3763	C	0
3764	A	0.932
3765	G	1.066
3766	G	1.265
3767	A	1.119
3768	A	1.319
3769	A	1.145
3770	G	0.999
3771	U	0.679
3772	A	0.679
3773	C	0.12
3774	U	0.493
3775	A	0.546
3776	U	0.173
3777	U	0.306
3778	U	0.333
3779	U	0.812
3780	U	2.011

3781	A	1.372
3782	G	1.105
3783	A	0.919
3784	U	0.506
3785	G	0.693
3786	G	0.373
3787	A	0.506
3788	A	0.746
3789	U	0.972
3790	A	0.959
3791	G	0.666
3792	A	1.052
3793	U	0.519
3794	A	1.239
3795	A	0.839
3796	G	0.493
3797	G	0.133
3798	C	0.027
3799	C	0
3800	C	0.04
3801	A	0.639
3802	A	0.719
3803	G	0.786
3804	A	0.919
3805	A	0.972
3806	G	1.092
3807	A	1.132
3808	A	0.493
3809	C	0
3810	A	1.066
3811	U	0.293
3812	G	1.838
3813	A	1.092
3814	G	1.758
3815	A	1.159
3816	A	1.345
3817	A	1.558
3818	U	0.733
3819	A	0.186
3820	U	0.08
3821	C	0.173
3822	A	0.266
3823	C	0.067

3824	A	0.133
3825	G	0.173
3826	U	0.346
3827	A	1.279
3828	A	0.946
3829	U	0.666
3830	U	0.333
3831	G	0.746
3832	G	2.411
3833	A	1.079
3834	G	1.625
3835	A	1.212
3836	G	1.478
3837	C	0.213
3838	A	0.559
3839	A	0.653
3840	U	0.306
3841	G	0.107
3842	G	0.053
3843	C	0.04
3844	U	0.173
3845	A	0.746
3846	G	1.492
3847	U	0.12
3848	G	0.4
3849	A	0.44
3850	U	0.226
3851	U	0.36
3852	U	0.546
3853	U	0.879
3854	A	0.852
3855	A	0.759
3856	C	0.107
3857	C	0.027
3858	U	0.027
3859	A	0.186
3860	C	0.107
3861	C	0.2
3862	A	0.613
3863	C	0.133
3864	C	0.266
3865	U	0.093
3866	G	0.599

3867	U	0.413
3868	A	0.733
3869	G	0.373
3870	U	0.346
3871	A	0.426
3872	G	0.253
3873	C	0.24
3874	A	0.866
3875	A	0.892
3876	A	1.212
3877	A	1.771
3878	G	1.092
3879	A	0.639
3880	A	0.586
3881	A	0.613
3882	U	0.733
3883	A	1.092
3884	G	0.999
3885	U	0.36
3886	A	0.36
3887	G	0.24
3888	C	0.053
3889	C	0.067
3890	A	0.306
3891	G	0.2
3892	C	0.2
3893	U	0.147
3894	G	0.293
3895	U	0.333
3896	G	0.413
3897	A	0.386
3898	U	0.093
3899	A	0.812
3900	A	1.745
3901	A	1.052
3902	U	0.333
3903	G	0.506
3904	U	0.16
3905	C	0.16
3906	A	0.679
3907	G	0.186
3908	C	0.067
3909	U	0.826

3910	A	1.572
3911	A	0.959
3912	A	0.653
3913	A	0.693
3914	G	0.346
3915	G	0.2
3916	G	0.147
3917	G	0.479
3918	A	1.212
3919	A	1.438
3920	G	0.28
3921	C	0.093
3922	C	0.093
3923	A	0.173
3924	U	0.173
3925	G	0.027
3926	C	0.08
3927	A	0.293
3928	U	0.027
3929	G	0.107
3930	G	0.226
3931	A	0.773
3932	C	0.067
3933	A	0.733
3934	A	0.679
3935	G	0.506
3936	U	0.32
3937	A	0.999
3938	G	0.533
3939	A	0.706
3940	C	0.067
3941	U	0.067
3942	G	0.453
3943	U	0.693
3944	A	1.185
3945	G	0.16
3946	C	0.426
3947	C	0.08
3948	C	0.107
3949	A	0.413
3950	G	0.533
3951	G	0.426
3952	A	0.533

3953	A	0.639
3954	U	0.493
3955	A	0.892
3956	U	0.573
3957	G	0.413
3958	G	0.226
3959	C	0.16
3960	A	0.36
3961	G	0.466
3962	C	0.253
3963	U	0.16
3964	A	0.733
3965	G	0.479
3966	A	0.44
3967	U	0.293
3968	U	0.2
3969	G	0.519
3970	U	0.346
3971	A	0.679
3972	C	0.293
3973	A	0.453
3974	C	0.493
3975	A	0.386
3976	U	0.373
3977	U	0.466
3978	U	0.466
3979	A	1.079
3980	G	0.946
3981	A	0.946
3982	A	1.052
3983	G	1.012
3984	G	1.039
3985	A	0.946
3986	A	0.653
3987	A	0.786
3988	A	0.879
3989	G	1.172
3990	U	0.453
3991	U	0.333
3992	A	0.892
3993	U	0.533
3994	C	0.226
3995	U	0.24

3996	U	0.24
3997	G	0.173
3998	G	0.147
3999	U	0.24
4000	A	0.626
4001	G	0.28
4002	C	0.186
4003	A	0.24
4004	G	0.24
4005	U	0.36
4006	U	0.493
4007	C	0.972
4008	A	0.946
4009	U	0.147
4010	G	0.373
4011	U	0.107
4012	A	1.012
4013	G	1.718
4014	C	1.079
4015	C	0
4016	A	0.346
4017	G	1.199
4018	U	0.746
4019	G	0.093
4020	G	0.08
4021	A	1.012
4022	U	0.546
4023	A	1.079
4024	U	0.466
4025	A	1.665
4026	U	0.386
4027	A	1.532
4028	G	1.066
4029	A	1.199
4030	A	1.145
4031	G	0.666
4032	C	0.093
4033	A	0.986
4034	G	0.919
4035	A	0.946
4036	A	0.852
4037	G	0.839
4038	U	0.573

4039	A	1.092
4040	A	0.519
4041	U	0.173
4042	U	0.08
4043	C	0.413
4044	C	0.16
4045	A	0.786
4046	G	0.266
4047	C	0.147
4048	A	0.693
4049	G	0.759
4050	A	1.145
4051	G	0.773
4052	A	0.653
4053	C	0.373
4054	A	1.132
4055	G	0.426
4056	G	0.186
4057	G	0.133
4058	C	0
4059	A	0.919
4060	A	0.906
4061	G	1.012
4062	A	1.185
4063	A	1.305
4064	A	0.946
4065	C	0.373
4066	A	0.932
4067	G	0.44
4068	C	0.346
4069	A	0.679
4070	U	0.479
4071	A	1.279
4072	C	0.093
4073	U	0.147
4074	U	0.12
4075	C	0.133
4076	C	0.093
4077	U	0.12
4078	C	0.147
4079	U	0.666
4080	U	0.906
4081	A	1.558

4082	A	0.999
4083	A	1.612
4084	A	0.812
4085	U	0.626
4086	U	0.626
4087	A	0.546
4088	G	0.32
4089	C	0.453
4090	A	0.426
4091	G	0.306
4092	G	0.413
4093	A	0.533
4094	A	0.546
4095	G	0.493
4096	A	0.733
4097	U	0.413
4098	G	0.333
4099	G	0.013
4100	C	0.013
4101	C	0.04
4102	A	0.746
4103	G	0.852
4104	U	0.559
4105	A	1.438
4106	A	1.119
4107	A	1.199
4108	A	0.906
4109	A	0.719
4110	C	0.333
4111	A	0.693
4112	G	0.746
4113	U	0.453
4114	A	0.826
4115	C	0.093
4116	A	1.172
4117	U	0.546
4118	A	1.172
4119	C	0.333
4120	A	1.305
4121	G	1.292
4122	A	1.332
4123	C	0.373
4124	A	0.972

4125	A	1.199
4126	U	0.533
4127	G	0.453
4128	G	0.36
4129	C	0.253
4130	A	0.653
4131	G	0.453
4132	C	0.293
4133	A	0.773
4134	A	1.172
4135	U	0.333
4136	U	0.373
4137	U	0.333
4138	C	0.333
4139	A	0.493
4140	C	0.24
4141	C	0.226
4142	A	0.466
4143	G	0.346
4144	U	0.44
4145	A	0.453
4146	C	0.32
4147	U	0.493
4148	A	0.773
4149	C	0.253
4150	A	1.092
4151	G	0.919
4152	U	0.666
4153	U	0.959
4154	A	1.705
4155	A	1.545
4156	G	1.319
4157	G	1.119
4158	C	0.2
4159	C	0.12
4160	G	0.147
4161	C	0.12
4162	C	0.133
4163	U	0.346
4164	G	0.653
4165	U	0.666
4166	U	0.506
4167	G	0.586

4168	G	0.986
4169	U	0.786
4170	G	0.333
4171	G	0.293
4172	G	0.226
4173	C	0.16
4174	G	0.186
4175	G	0
4176	G	0.253
4177	G	0.373
4178	A	0.826
4179	U	0.346
4180	C	0.333
4181	A	0.826
4182	A	0.746
4183	G	0.453
4184	C	0.293
4185	A	0.413
4186	G	0.266
4187	G	0.653
4188	A	0.519
4189	A	1.532
4190	U	0.373
4191	U	2.171
4192	U	0.333
4193	G	0.506
4194	G	0.44
4195	C	0.546
4196	A	0.613
4197	U	0.679
4198	U	0.626
4199	C	0.44
4200	C	0.293
4201	C	0.426
4202	U	0.573
4203	A	1.745
4204	C	0.626
4205	A	0.626
4206	A	0.253
4207	U	0.147
4208	C	0.147
4209	C	0.186
4210	C	0.226

4211	C	0.253
4212	A	1.066
4213	A	1.252
4214	A	1.092
4215	G	0.653
4216	U	0.36
4217	C	0.266
4218	A	0.799
4219	A	1.026
4220	G	0.693
4221	G	0.892
4222	A	0.746
4223	G	0.706
4224	U	0.426
4225	A	0.919
4226	A	0.892
4227	U	0.666
4228	A	2.397
4229	G	1.145
4230	A	0.866
4231	A	0.839
4232	U	0.333
4233	C	0.253
4234	U	0.44
4235	A	1.172
4236	U	0.493
4237	G	0.892
4238	A	0.812
4239	A	0.932
4240	U	0.666
4241	A	1.225
4242	A	1.092
4243	A	1.279
4244	G	1.172
4245	A	0.972
4246	A	0.972
4247	U	0.626
4248	U	0.919
4249	A	1.159
4250	A	1.079
4251	A	1.612
4252	G	0.826
4253	A	1.199

4254	A	0.919
4255	A	0.733
4256	A	0.773
4257	U	0.493
4258	U	0.639
4259	A	1.265
4260	U	0.826
4261	A	1.225
4262	G	0.479
4263	G	0.533
4264	A	0.506
4265	C	0.28
4266	A	1.052
4267	G	0.719
4268	G	0.626
4269	U	0.306
4270	A	0.812
4271	A	0.839
4272	G	1.066
4273	A	0.906
4274	G	0.839
4275	A	0.839
4276	U	0.466
4277	C	0.333
4278	A	0.759
4279	G	0.719
4280	G	0.147
4281	C	0.12
4282	U	0.12
4283	G	0.226
4284	A	0.466
4285	A	0.733
4286	C	0.346
4287	A	0.346
4288	U	0.519
4289	C	0.346
4290	U	0.506
4291	U	0.826
4292	A	1.119
4293	A	1.452
4294	G	1.332
4295	A	0.826
4296	C	0.413

4297	A	0.253
4298	G	0.226
4299	C	0.373
4300	A	0.173
4301	G	0.32
4302	U	0.346
4303	A	0.746
4304	C	0.533
4305	A	0.892
4306	A	0.586
4307	A	0.919
4308	U	0.733
4309	G	0.919
4310	G	0.373
4311	C	0.346
4312	A	0.373
4313	G	0.493
4314	U	0.24
4315	A	0.453
4316	U	0.479
4317	U	0.36
4318	C	0.373
4319	A	0.759
4320	U	0.333
4321	C	0.186
4322	C	0.12
4323	A	0.36
4324	C	0.253
4325	A	0.719
4326	A	0.812
4327	U	0.453
4328	U	0.666
4329	U	0.773
4330	U	0.799
4331	A	1.212
4332	A	0.879
4333	A	0.906
4334	A	0.986
4335	G	0.826
4336	A	0.839
4337	A	0.653
4338	A	0.826
4339	A	0.266

4340	G	0.36
4341	G	0.706
4342	G	0.373
4343	G	0.027
4344	G	0.04
4345	G	0.067
4346	A	0.067
4347	U	0.12
4348	U	0.133
4349	G	0.093
4350	G	0.04
4351	G	0.107
4352	G	0.04
4353	G	0.293
4354	G	0.133
4355	U	0.226
4356	A	0.413
4357	C	1.252
4358	A	0.626
4359	G	0.426
4360	U	0.4
4361	G	0.333
4362	C	0.4
4363	A	0.253
4364	G	0
4365	G	0.253
4366	G	0.28
4367	G	0.333
4368	A	0
4369	A	1.119
4370	A	0.519
4371	G	0.546
4372	A	0.759
4373	A	0.799
4374	U	0.706
4375	A	0.932
4376	G	0.746
4377	U	0.852
4378	A	0.706
4379	G	0.586
4380	A	0.773
4381	C	0.386
4382	A	1.359

4383	U	0.706
4384	A	1.145
4385	A	0.839
4386	U	1.012
4387	A	1.159
4388	G	0.586
4389	C	0.653
4390	A	0.533
4391	A	0.493
4392	C	0.266
4393	A	0.733
4394	G	0.413
4395	A	0.919
4396	C	0.453
4397	A	1.372
4398	U	0.679
4399	A	1.398
4400	C	0.546
4401	A	1.132
4402	A	0.586
4403	A	0.479
4404	C	0.293
4405	U	0.519
4406	A	0.693
4407	A	0.826
4408	A	1.145
4409	G	0.892
4410	A	1.079
4411	A	0.906
4412	U	0.693
4413	U	0.799
4414	A	0.839
4415	C	0.413
4416	A	0.932
4417	A	0.826
4418	A	0.932
4419	A	0.746
4420	A	0.866
4421	C	0.293
4422	A	0.666
4423	A	0.466
4424	A	0.626
4425	U	0.373

4426	U	0.559
4427	A	1.665
4428	C	0.2
4429	A	1.026
4430	A	1.026
4431	A	1.172
4432	A	0.653
4433	A	0.866
4434	U	1.039
4435	U	0.919
4436	C	0.493
4437	A	1.012
4438	A	0.746
4439	A	0.799
4440	A	0.653
4441	U	0.613
4442	U	0.573
4443	U	0.373
4444	U	0.333
4445	C	0.426
4446	G	0.746
4447	G	0.36
4448	G	0.36
4449	U	0.027
4450	U	0.559
4451	U	1.239
4452	A	1.279
4453	U	0.799
4454	U	1.558
4455	A	0.546
4456	C	0.08
4457	A	0.32
4458	G	0
4459	G	0.4
4460	G	0.28
4461	A	0.386
4462	C	0.04
4463	A	1.066
4464	G	0.586
4465	C	0.333
4466	A	0.906
4467	G	0.666
4468	A	0.906

4469	G	0.306
4470	A	0.826
4471	U	0.027
4472	C	0
4473	C	0
4474	A	0.906
4475	G	0.373
4476	U	0.373
4477	U	0.733
4478	U	0.266
4479	G	0.559
4480	G	0.519
4481	A	0.693
4482	A	0.919
4483	A	1.212
4484	G	0.4
4485	G	0.599
4486	A	0.479
4487	C	0.107
4488	C	0.333
4489	A	0.826
4490	G	0.373
4491	C	0.24
4492	A	0.919
4493	A	1.026
4494	A	1.492
4495	G	0.133
4496	C	0.333
4497	U	0.16
4498	C	0.373
4499	C	0.16
4500	U	0.173
4501	C	0.093
4502	U	0.386
4503	G	0.799
4504	G	0.293
4505	A	1.172
4506	A	1.185
4507	A	0.759
4508	G	0.04
4509	G	0.266
4510	U	0.226
4511	G	0.373

4512	A	0.599
4513	A	0.466
4514	G	0.2
4515	G	0.306
4516	G	0.107
4517	G	0
4518	C	0
4519	A	0.613
4520	G	0.892
4521	U	0.626
4522	A	0.959
4523	G	0.892
4524	U	0.519
4525	A	1.012
4526	A	1.172
4527	U	0.919
4528	A	0.759
4529	C	0.093
4530	A	1.225
4531	A	0.879
4532	G	1.039
4533	A	1.652
4534	U	0.986
4535	A	1.052
4536	A	1.185
4537	U	0.799
4538	A	0.852
4539	G	0.693
4540	U	0.226
4541	G	0.812
4542	A	1.305
4543	C	0.466
4544	A	1.758
4545	U	0.559
4546	A	1.638
4547	A	0.812
4548	A	1.292
4549	A	1.199
4550	G	1.212
4551	U	0.107
4552	A	0.133
4553	G	0.173
4554	U	0.013

4555	G	0.133
4556	C	0.08
4557	C	0
4558	A	0.173
4559	A	0.2
4560	G	0.147
4561	A	1.066
4562	A	1.958
4563	G	0.453
4564	A	1.185
4565	A	0.906
4566	A	0.986
4567	A	1.092
4568	G	0.666
4569	C	0.173
4570	A	1.105
4571	A	0.932
4572	A	1.105
4573	G	0.546
4574	A	0.506
4575	U	0.173
4576	C	0.293
4577	A	1.026
4578	U	0.453
4579	C	0.533
4580	A	0.932
4581	G	1.119
4582	G	0.719
4583	G	0.28
4584	A	0.666
4585	U	0.546
4586	U	0.466
4587	A	1.545
4588	U	0.44
4589	G	0.4
4590	G	0.453
4591	A	0.639
4592	A	0.533
4593	A	0.4
4594	A	0.266
4595	C	0.186
4596	A	0.559
4597	G	0.586

4598	A	0.906
4599	U	0.426
4600	G	0.426
4601	G	0.147
4602	C	0.013
4603	A	0.426
4604	G	0.133
4605	G	0.386
4606	U	0.133
4607	G	0.306
4608	A	0.852
4609	U	0.693
4610	G	0.719
4611	A	0.946
4612	U	0.693
4613	U	0.533
4614	G	0.613
4615	U	0.186
4616	G	0.932
4617	U	0.133
4618	G	0.28
4619	G	0.453
4620	C	0.333
4621	A	1.105
4622	A	0.906
4623	G	1.012
4624	U	0.879
4625	A	1.319
4626	G	1.092
4627	A	1.026
4628	C	0.386
4629	A	1.079
4630	G	0.839
4631	G	0.919
4632	A	1.105
4633	U	0.506
4634	G	1.052
4635	A	0.879
4636	G	0.506
4637	G	0.333
4638	A	0.613
4639	U	0.506
4640	U	0.839

4641	A	1.079
4642	A	0.586
4643	C	0.253
4644	A	0.333
4645	C	0.08
4646	A	0.333
4647	U	0.12
4648	G	0.546
4649	G	0.666
4650	A	1.039
4651	A	0.946
4652	A	1.239
4653	A	1.172
4654	G	0.719
4655	A	0.866
4656	U	0.44
4657	U	0.906
4658	A	0.919
4659	G	0.852
4660	U	0.533
4661	A	1.345
4662	A	1.052
4663	A	0.733
4664	A	0.573
4665	C	0.147
4666	A	0.44
4667	C	0.013
4668	C	0
4669	A	0.226
4670	U	0.107
4671	A	0.453
4672	U	0.093
4673	G	0.253
4674	U	0.346
4675	A	1.385
4676	U	0.12
4677	A	0.919
4678	U	0.226
4679	U	0.293
4680	U	0.519
4681	C	0.2
4682	A	0.892
4683	A	0.986

4684	G	1.132
4685	G	0.746
4686	A	0.773
4687	A	0.826
4688	A	0.826
4689	G	0.266
4690	C	0.04
4691	U	0.306
4692	A	0.972
4693	A	1.212
4694	G	0.586
4695	G	0.426
4696	A	0.506
4697	C	0.173
4698	U	0.253
4699	G	0.346
4700	G	0.173
4701	U	0.16
4702	U	0.373
4703	U	0.426
4704	U	0.506
4705	A	0.919
4706	U	0.453
4707	A	1.145
4708	G	0.626
4709	A	1.372
4710	C	0.306
4711	A	0.453
4712	U	0.173
4713	C	0.16
4714	A	0.506
4715	C	0.08
4716	U	0.107
4717	A	0.666
4718	U	0.067
4719	G	0.346
4720	A	0.453
4721	A	0.666
4722	A	0.586
4723	G	1.692
4724	U	0.333
4725	A	0.413
4726	C	0.08

4727	U	0.386
4728	A	0.559
4729	A	0.493
4730	U	0.186
4731	C	0.186
4732	C	0.36
4733	A	0.906
4734	A	0.879
4735	A	0.892
4736	A	0.679
4737	A	0.746
4738	U	0.586
4739	A	1.172
4740	A	0.906
4741	G	0.413
4742	U	0.293
4743	U	0.44
4744	C	0.226
4745	A	1.039
4746	G	0.932
4747	A	1.026
4748	A	0.986
4749	G	1.105
4750	U	0.666
4751	A	1.465
4752	C	0.306
4753	A	0.346
4754	C	0.04
4755	A	0.186
4756	U	0.133
4757	C	0.067
4758	C	0.08
4759	C	0.16
4760	A	1.132
4761	C	0.266
4762	U	0.706
4763	A	0.826
4764	G	1.811
4765	G	0.226
4766	G	0.107
4767	G	0
4768	A	0
4769	U	0.08

4770	G	0
4771	C	0.12
4772	U	0.466
4773	A	1.199
4774	A	0.693
4775	A	0.773
4776	U	0.266
4777	U	0.346
4778	A	0.786
4779	G	0.586
4780	U	0.28
4781	A	0.986
4782	A	0.932
4783	U	0.213
4784	A	1.199
4785	A	0.959
4786	C	0
4787	A	0.812
4788	A	0.639
4789	C	0
4790	A	0.972
4791	U	0.147
4792	A	0.919
4793	U	0.506
4794	U	0.693
4795	G	2.571
4796	G	0.693
4797	G	0.093
4798	G	0
4799	U	0.053
4800	C	0.053
4801	U	0
4802	G	0.067
4803	C	0.093
4804	A	1.798
4805	U	0.599
4806	A	1.478
4807	C	0
4808	A	0.213
4809	G	0.093
4810	G	0.759
4811	A	0.759
4812	G	0.906

4813	A	1.452
4814	A	1.838
4815	A	1.119
4816	G	1.026
4817	A	1.718
4818	G	1.558
4819	A	1.199
4820	C	0.027
4821	U	0.08
4822	G	0.053
4823	G	0.479
4824	C	0.04
4825	A	1.305
4826	U	0.746
4827	U	1.332
4828	U	0.679
4829	G	0
4830	G	0.067
4831	G	0.013
4832	U	0
4833	C	0.093
4834	A	0.013
4835	G	0.027
4836	G	0
4837	G	0.053
4838	A	0.666
4839	G	1.239
4840	U	0.32
4841	C	0
4842	U	0.04
4843	C	0
4844	C	0.013
4845	A	0.346
4846	U	1.265
4847	A	1.225
4848	G	1.359
4849	A	1.172
4850	A	0.852
4851	U	0.226
4852	G	0.013
4853	G	0.053
4854	A	0.067
4855	G	0

4856	G	0.852
4857	A	1.385
4858	A	1.119
4859	A	1.066
4860	A	1.119
4861	A	1.079
4862	G	1.532
4863	A	1.319
4864	G	1.066
4865	A	1.319
4866	U	0.386
4867	A	1.465
4868	U	0.546
4869	A	1.359
4870	G	0.946
4871	C	0.253
4872	A	1.199
4873	C	0
4874	A	0.852
4875	C	0.053
4876	A	1.079
4877	A	0.906
4878	G	1.185
4879	U	0.773
4880	A	1.518
4881	G	1.172
4882	A	0.812
4883	C	0.027
4884	C	0
4885	C	0.067
4886	U	0.067
4887	G	0.013
4888	A	0.013
4889	C	0
4890	C	0.04
4891	U	0.293
4892	A	2.198
4893	G	0.133
4894	C	0.013
4895	A	0.173
4896	G	0.053
4897	A	0.2
4898	C	0

4899	C	0.107
4900	A	1.039
4901	A	0.679
4902	C	0.16
4903	U	0.373
4904	A	0.693
4905	A	0.706
4906	U	0.36
4907	U	0.36
4908	C	0.107
4909	A	0.306
4910	U	0
4911	C	0.04
4912	U	0.013
4913	G	0.067
4914	C	0.04
4915	A	0.786
4916	C	0.067
4917	U	0.32
4918	A	0.599
4919	U	0.253
4920	U	0.466
4921	U	0.932
4922	U	0.519
4923	G	0.799
4924	A	1.252
4925	U	0.759
4926	U	0.24
4927	G	0.107
4928	U	0.04
4929	U	0.173
4930	U	0.28
4931	U	0.173
4932	U	0.16
4933	C	0.04
4934	A	0.839
4935	G	0.906
4936	A	1.185
4937	A	0.892
4938	U	0.4
4939	C	0.08
4940	U	0.12
4941	G	0.32

4942	C	0
4943	U	0.093
4944	A	0.693
4945	U	0.506
4946	A	0.946
4947	A	0.773
4948	G	0.666
4949	A	0.679
4950	A	1.692
4951	A	0.4
4952	U	0.266
4953	A	0.133
4954	C	0
4955	C	0
4956	A	0.679
4957	U	0.559
4958	A	3.596
4959	U	1.332
4960	U	0.266
4961	A	0
4962	G	0.027
4963	G	0.04
4964	A	0.067
4965	C	0.093
4966	G	0.839
4967	U	0.586
4968	A	1.385
4969	U	0.573
4970	A	1.252
4971	G	1.052
4972	U	0.666
4973	U	0.573
4974	A	1.718
4975	G	0
4976	U	0.013
4977	C	0.027
4978	C	0.013
4979	U	0
4980	A	0.04
4981	G	0.067
4982	G	0.746
4983	U	0.24
4984	G	0.067

4985	U	0.067
4986	G	0.213
4987	A	0.133
4988	A	0.4
4989	U	0.573
4990	A	0.693
4991	U	0.306
4992	C	0.719
4993	A	0.573
4994	A	0.346
4995	G	1.105
4996	C	0.493
4997	A	0.173
4998	G	0.04
4999	G	0.24
5000	A	0.28
5001	C	0.586
5002	A	0.719
5003	U	0.173
5004	A	1.252
5005	A	0.546
5006	C	1.066
5007	A	0.826
5008	A	0.12
5009	G	0.999
5010	G	0.906
5011	U	0.693
5012	A	0.653
5013	G	0.519
5014	G	0.892
5015	A	0.586
5016	U	0.226
5017	C	0.32
5018	U	0.107
5019	C	0.186
5020	U	0.067
5021	A	0.093
5022	C	0.333
5023	A	0.693
5024	G	0.2
5025	U	0.852
5026	A	0.826
5027	C	0.706

5028	U	0.733
5029	U	0.053
5030	G	0.12
5031	G	0.027
5032	C	0
5033	A	0
5034	C	0.013
5035	U	0.08
5036	A	0.053
5037	G	0.133
5038	C	0.466
5039	A	1.079
5040	G	0.333
5041	C	0.839
5042	A	0.533
5043	U	0.2
5044	U	0.573
5045	A	0.346
5046	A	0.453
5047	U	0.932
5048	A	0.746
5049	A	0.453
5050	A	0.986
5051	A	1.478
5052	C	0.826
5053	C	1.132
5054	A	0.919
5055	A	0.426
5056	A	0.413
5057	A	0.919
5058	C	1.092
5059	A	1.825
5060	G	0.333
5061	A	1.185
5062	U	0.746
5063	A	1.558
5064	A	0.493
5065	A	0.746
5066	G	0.706
5067	C	1.865
5068	C	0.253
5069	A	0.346
5070	C	0.426

5071	C	1.305
5072	U	0.546
5073	U	0.253
5074	U	0.027
5075	G	0.186
5076	C	0.213
5077	C	1.132
5078	U	1.252
5079	A	0.226
5080	G	0.413
5081	U	0.666
5082	G	0.799
5083	U	0.666
5084	U	0.067
5085	A	0.173
5086	G	0.866
5087	G	1.026
5088	A	0.36
5089	A	0.013
5090	A	0.426
5091	C	0.373
5092	U	0.639
5093	G	0.04
5094	A	0.08
5095	C	0.533
5096	A	0.28
5097	G	0
5098	A	0.186
5099	G	0.466
5100	G	0
5101	A	1.079
5102	C	0.706
5103	A	1.066
5104	G	0.386
5105	A	0.413
5106	U	0.36
5107	G	0.559
5108	G	0.852
5109	A	0
5110	A	1.132
5111	C	1.212
5112	A	0.306
5113	A	0.226

5114	G	0.107
5115	C	0
5116	C	0.04
5117	C	
5118	C	
5119	A	
5120	G	1.012
5121	A	1.105
5122	A	0.786
5123	G	0.613
5124	A	0
5125	C	0
5126	C	0.746
5127	A	1.638
5128	A	0.533
5129	G	0.28
5130	G	0.013
5131	G	0.107
5132	C	0.16
5133	C	0.666
5134	A	0
5135	C	1.079
5136	A	0.919
5137	G	0.386
5138	A	0.812
5139	G	0.107
5140	G	1.039
5141	G	1.438
5142	A	0.226
5143	G	0
5144	C	0.306
5145	C	0.493
5146	A	0.346
5147	U	0.919
5148	A	0.013
5149	C	1.145
5150	A	1.052
5151	A	0.693
5152	U	0.679
5153	G	0.759
5154	A	0.599
5155	A	0.093
5156	U	0.186

5157	G	0.147
5158	G	0.559
5159	A	0.12
5160	C	0.599
5161	A	0.04
5162	C	0.733
5163	U	1.026
5164	A	0.733
5165	G	0.693
5166	A	0
5167	G	0
5168	C	0
5169	U	0
5170	U	0.36
5171	U	1.105
5172	U	0.666
5173	A	0.533
5174	G	0.626
5175	A	0.28
5176	G	0.573
5177	G	0.892
5178	A	0.679
5179	A	0.32
5180	C	0.559
5181	U	0.493
5182	U	0.892
5183	A	0.932
5184	A	0.866
5185	G	0.733
5186	A	0.453
5187	G	0.213
5188	U	0.519
5189	G	0.613
5190	A	0.639
5191	A	0.147
5192	G	0.067
5193	C	0.08
5194	U	0.213
5195	G	0.293
5196	U	0.519
5197	U	1.305
5198	A	0.932
5199	G	0.906

5200	A	0.107
5201	C	0.733
5202	A	0.08
5203	U	0.226
5204	U	0.346
5205	U	0.799
5206	U	0.2
5207	C	0.093
5208	C	0.186
5209	U	0.4
5210	A	0
5211	G	0.04
5212	G	0.386
5213	A	0.2
5214	U	1.359
5215	A	0.226
5216	U	0.28
5217	G	0
5218	G	0.107
5219	C	0.08
5220	U	0.133
5221	C	0
5222	C	0.186
5223	A	0.44
5224	U	0.879
5225	A	0.493
5226	A	0.107
5227	C	0.333
5228	U	0.586
5229	U	1.585
5230	A	0.999
5231	G	0.559
5232	G	0.573
5233	A	0.186
5234	C	0.653
5235	A	0.519
5236	A	0
5237	C	0.679
5238	A	0.16
5239	U	0.919
5240	A	0.08
5241	U	0.013
5242	C	0.013

5243	U	0.2
5244	A	0.972
5245	U	0.24
5246	G	0.826
5247	A	0.786
5248	A	0.892
5249	A	0.559
5250	C	0.107
5251	U	0.333
5252	U	0.44
5253	A	1.012
5254	C	0.226
5255	G	0.653
5256	G	0.24
5257	G	0.107
5258	G	0.08
5259	A	0.173
5260	U	0.2
5261	A	0.2
5262	C	0.213
5263	U	0.226
5264	U	0.213
5265	G	0.173
5266	G	0.479
5267	G	0.147
5268	C	0.027
5269	A	0.147
5270	G	0.266
5271	G	0.226
5272	A	0.373
5273	G	0.879
5274	U	0.346
5275	G	0.12
5276	G	0.28
5277	A	0.533
5278	A	0.639
5279	G	0.639
5280	C	0.28
5281	C	0.213
5282	A	0.293
5283	U	0.866
5284	A	0.693
5285	A	1.372

5286	U	1.292
5287	A	0.946
5288	A	1.532
5289	G	1.332
5290	A	1.398
5291	A	1.212
5292	U	0.519
5293	U	0.293
5294	C	0.266
5295	U	0.173
5296	G	0.28
5297	C	0.093
5298	A	0.2
5299	A	1.225
5300	C	1.039
5301	A	0.546
5302	A	1.092
5303	C	0.546
5304	U	0.266
5305	G	0.266
5306	C	0.067
5307	U	0.24
5308	G	0.093
5309	U	0.44
5310	U	0.426
5311	U	0.293
5312	A	0.733
5313	U	0.706
5314	C	0.133
5315	C	0.253
5316	A	0.293
5317	U	0.346
5318	U	0.28
5319	U	0.746
5320	C	0.706
5321	A	0.173
5322	G	0.746
5323	A	1.225
5324	A	0.773
5325	U	0.506
5326	U	0.253
5327	G	0.28
5328	G	0.506

5329	G	0.36
5330	U	0.386
5331	G	0.133
5332	U	0.213
5333	C	0.293
5334	G	0
5335	A	0.32
5336	C	0.32
5337	A	0.24
5338	U	1.066
5339	A	0.559
5340	G	0.906
5341	C	0.173
5342	A	0.08
5343	G	0.653
5344	A	0.613
5345	A	0.919
5346	U	0.679
5347	A	0.599
5348	G	1.398
5349	G	0.773
5350	C	0.36
5351	G	0.186
5352	U	0.546
5353	U	0.932
5354	A	1.159
5355	C	0.852
5356	U	0.226
5357	C	0.373
5358	G	0.213
5359	A	0.733
5360	C	1.345
5361	A	0.426
5362	G	0.679
5363	A	0.906
5364	G	0.786
5365	G	0.666
5366	A	0.839
5367	G	0.919
5368	A	1.225
5369	G	1.185
5370	C	0.773
5371	A	0.533

5372	A	1.039
5373	G	0.706
5374	A	0.733
5375	A	0.946
5376	A	1.132
5377	U	0.226
5378	G	0.08
5379	G	0.226
5380	A	0.479
5381	G	0.186
5382	C	0.16
5383	C	0.147
5384	A	0.173
5385	G	0.226
5386	U	0.679
5387	A	0.839
5388	G	2.171
5389	A	1.438
5390	U	0.373
5391	C	0.346
5392	C	0.36
5393	U	0.413
5394	A	0.546
5395	G	0.4
5396	A	0.373
5397	C	0.866
5398	U	0.12
5399	A	0.759
5400	G	1.425
5401	A	1.558
5402	G	1.132
5403	C	0.573
5404	C	0.32
5405	C	0.12
5406	U	0.186
5407	G	0.186
5408	G	0.2
5409	A	0.413
5410	A	0.839
5411	G	1.252
5412	C	1.052
5413	A	0.986
5414	U	0.426

5415	C	0.946
5416	C	0.253
5417	A	0.16
5418	G	0.133
5419	G	0.16
5420	A	0.493
5421	A	1.958
5422	G	1.026
5423	U	0.852
5424	C	0.466
5425	A	0.333
5426	G	0.213
5427	C	0.759
5428	C	0.706
5429	U	0.479
5430	A	0
5431	A	0.24
5432	A	0.706
5433	A	0.879
5434	C	0.799
5435	U	0.879
5436	G	0.999
5437	C	0.36
5438	U	0.573
5439	U	0.599
5440	G	0.36
5441	U	0.133
5442	A	0.306
5443	C	0.266
5444	C	0.44
5445	A	0.759
5446	A	0.599
5447	U	0.08
5448	U	0.186
5449	G	0.12
5450	C	0.453
5451	U	0.546
5452	A	0.852
5453	U	0.133
5454	U	0.253
5455	G	0.799
5456	U	0.666
5457	A	1.292

5458	A	1.026
5459	A	1.332
5460	A	1.132
5461	A	0.573
5462	G	1.185
5463	U	0.413
5464	G	0.892
5465	U	0.773
5466	U	1.119
5467	G	0.666
5468	C	0.453
5469	U	0.666
5470	U	0.16
5471	U	0.786
5472	C	0.04
5473	A	0
5474	U	0.173
5475	U	0.373
5476	G	0.226
5477	C	0.453
5478	C	0.16
5479	A	0.559
5480	A	0.812
5481	G	0.32
5482	U	0.08
5483	U	0.546
5484	U	0.812
5485	G	0.546
5486	U	0.306
5487	U	0
5488	U	1.572
5489	C	0
5490	A	1.545
5491	U	0.759
5492	G	1.092
5493	A	1.279
5494	C	0.093
5495	A	1.798
5496	A	0.986
5497	A	1.865
5498	A	2.158
5499	G	0.053
5500	C	0.147

5501	C	0.226
5502	U	0.573
5503	U	0.253
5504	A	1.332
5505	G	0.826
5506	G	0.426
5507	C	0.786
5508	A	2.118
5509	U	0.107
5510	C	0.107
5511	U	0.773
5512	C	0.293
5513	C	0.12
5514	U	0.027
5515	A	0.852
5516	U	0.559
5517	G	0.786
5518	G	0.373
5519	C	0.173
5520	A	0.759
5521	G	0.866
5522	G	0.746
5523	A	1.505
5524	A	0.879
5525	G	0.586
5526	A	0.746
5527	A	0.919
5528	G	0.626
5529	C	0.573
5530	G	0.453
5531	G	0.413
5532	A	0.226
5533	G	0.013
5534	A	0.067
5535	C	0
5536	A	0.147
5537	G	0.533
5538	C	0.479
5539	G	0.333
5540	A	0.32
5541	C	0.453
5542	G	0.147
5543	A	0.027

5544	A	0.599
5545	G	0.599
5546	A	0.44
5547	G	0.373
5548	C	0.067
5549	U	0
5550	C	0
5551	A	0.12
5552	U	0.306
5553	C	0.253
5554	A	1.398
5555	G	1.119
5556	A	1.105
5557	A	0.906
5558	C	0.36
5559	A	1.532
5560	G	0.533
5561	U	0.639
5562	C	0.413
5563	A	0.999
5564	G	0.746
5565	A	0.639
5566	C	0
5567	U	0.24
5568	C	0.226
5569	A	0.4
5570	U	0.666
5571	C	0.186
5572	A	0.879
5573	A	0.999
5574	G	0.24
5575	C	0
5576	U	0.027
5577	U	
5578	C	
5579	U	
5580	C	0
5581	U	0.866
5582	A	0.906
5583	U	0.453
5584	C	0.24
5585	A	0.573
5586	A	0.812

5587	A	0.613
5588	G	0.24
5589	C	0.213
5590	A	0.852
5591	G	0.812
5592	U	0.533
5593	A	1.279
5594	A	0.733
5595	G	1.039
5596	U	0.773
5597	A	1.052
5598	G	0.679
5599	U	0.147
5600	A	0.186
5601	C	0.08
5602	A	0.466
5603	U	0.826
5604	G	0.653
5605	U	0.093
5606	A	0.013
5607	A	0.306
5608	U	0.067
5609	G	0.053
5610	C	0
5611	A	0.506
5612	A	0.746
5613	C	0.306
5614	C	0.16
5615	U	0.173
5616	A	0.186
5617	U	0.253
5618	A	0.746
5619	A	0.693
5620	U	0.506
5621	A	0.759
5622	G	0.346
5623	U	0.4
5624	A	0.746
5625	G	0.559
5626	C	0.16
5627	A	0.586
5628	A	0.133
5629	U	0.2

5630	A	0.147
5631	G	0.027
5632	U	0.293
5633	A	0.573
5634	G	0.333
5635	C	0
5636	A	0.067
5637	U	0.32
5638	U	0.2
5639	A	0.28
5640	G	0.466
5641	U	0.812
5642	A	0.466
5643	G	0.266
5644	U	0.373
5645	A	0.786
5646	G	0.706
5647	C	0.453
5648	A	0.253
5649	A	0.639
5650	U	0.093
5651	A	0.693
5652	A	0.773
5653	U	0.386
5654	A	0.586
5655	A	0.626
5656	U	0.333
5657	A	0.719
5658	G	0.586
5659	C	0.16
5660	A	0.546
5661	A	0.706
5662	U	0.413
5663	A	1.012
5664	G	0.733
5665	U	0.44
5666	U	0.147
5667	G	0.213
5668	U	0.253
5669	G	0.599
5670	U	0.293
5671	G	0.466
5672	G	0.186

5673	U	0.133
5674	C	0.36
5675	C	0.346
5676	A	0.839
5677	U	0.946
5678	A	0.959
5679	G	0.773
5680	U	0.426
5681	A	0.892
5682	A	0.719
5683	U	0.32
5684	C	0.266
5685	A	0.719
5686	U	0.626
5687	A	1.132
5688	G	0.679
5689	A	0.493
5690	A	0.653
5691	U	0.36
5692	A	0.866
5693	U	0.373
5694	A	0.919
5695	G	0.706
5696	G	0.919
5697	A	1.052
5698	A	0.719
5699	A	0.852
5700	A	0.759
5701	U	0.626
5702	A	0.839
5703	U	0.253
5704	U	0.36
5705	A	1.225
5706	A	1.012
5707	G	1.239
5708	A	0.959
5709	C	0.44
5710	A	0.693
5711	A	0.892
5712	A	0.906
5713	G	0.733
5714	A	1.026
5715	A	0.986

5716	A	0.759
5717	A	0.812
5718	A	0.773
5719	U	0.373
5720	A	1.012
5721	G	0.773
5722	A	0.733
5723	C	0.186
5724	A	0.679
5725	G	0.413
5726	G	0.266
5727	U	0.306
5728	U	0.493
5729	A	0.986
5730	A	0.932
5731	U	0.653
5732	U	0.333
5733	G	0.426
5734	A	0.666
5735	U	0.453
5736	A	0.972
5737	G	0.453
5738	A	0.546
5739	C	0.213
5740	U	0.466
5741	A	0.826
5742	A	0.839
5743	U	0.573
5744	A	1.292
5745	G	0.16
5746	A	0.306
5747	A	0.479
5748	A	0.626
5749	G	0.546
5750	A	0.506
5751	G	0.147
5752	C	0
5753	A	0.4
5754	G	0.293
5755	A	0.892
5756	A	0.932
5757	G	0.746
5758	A	0.759

5759	C	0.253
5760	A	0.906
5761	G	1.079
5762	U	0.386
5763	G	0.107
5764	G	0.133
5765	C	0.08
5766	A	0.293
5767	A	0.36
5768	U	0.12
5769	G	0.266
5770	A	0.4
5771	G	0.266
5772	A	0.373
5773	G	0.32
5774	U	0.186
5775	G	1.079
5776	A	1.066
5777	A	1.292
5778	G	0.972
5779	G	1.305
5780	A	1.145
5781	G	1.079
5782	A	1.425
5783	A	1.159
5784	G	1.039
5785	U	0.386
5786	A	0.266
5787	U	0.586
5788	C	0.253
5789	A	0.812
5790	G	0.879
5791	C	0.413
5792	A	0.559
5793	C	0.027
5794	U	0
5795	U	0.08
5796	G	0.093
5797	U	0.053
5798	G	0.067
5799	G	0.173
5800	A	1.092
5801	G	0.346

5802	A	0.866
5803	U	0
5804	G	0
5805	G	0
5806	G	0
5807	G	0.013
5808	G	0.852
5809	U	0.693
5810	G	1.625
5811	G	1.532
5812	A	1.718
5813	A	1.478
5814	A	1.492
5815	U	0.759
5816	G	0.599
5817	G	0.546
5818	G	0.053
5819	G	0
5820	C	0
5821	A	0.426
5822	C	0.2
5823	C	0.186
5824	A	0.586
5825	U	0.453
5826	G	0
5827	C	0
5828	U	0.04
5829	C	0.027
5830	C	0.186
5831	U	1.132
5832	U	1.319
5833	G	1.212
5834	G	0.866
5835	G	0.932
5836	A	1.398
5837	U	0.506
5838	A	1.705
5839	U	1.052
5840	U	0.839
5841	G	1.319
5842	A	1.558
5843	U	0.706
5844	G	1.705

5845	A	1.345
5846	U	0.293
5847	C	0.08
5848	U	0
5849	G	0.16
5850	U	0.226
5851	A	0.599
5852	G	1.678
5853	U	0.773
5854	G	0.733
5855	C	0.36
5856	U	0.2
5857	A	0.226
5858	C	0.027
5859	A	0.266
5860	G	0.266
5861	A	0.666
5862	A	1.305
5863	A	1.145
5864	A	0.919
5865	A	1.478
5866	U	1.026
5867	U	0.346
5868	G	0.013
5869	U	0.053
5870	G	0
5871	G	0.04
5872	G	0
5873	U	0.04
5874	C	0.067
5875	A	0.133
5876	C	0.04
5877	A	0.186
5878	G	0.24
5879	U	0.666
5880	C	0.266
5881	U	0.799
5882	A	1.026
5883	U	0.466
5884	U	0.613
5885	A	1.212
5886	U	0.466
5887	G	0.999

5888	G	1.172
5889	G	0.679
5890	G	0.16
5891	U	0.133
5892	A	0.519
5893	C	0.04
5894	C	0.08
5895	U	0.426
5896	G	0.44
5897	U	0.253
5898	G	0.067
5899	U	0.107
5900	G	0.107
5901	G	0.266
5902	A	0.866
5903	A	1.185
5904	G	0.413
5905	G	0.599
5906	A	1.092
5907	A	0.999
5908	G	0.786
5909	C	0.306
5910	A	0.786
5911	A	0.559
5912	C	0
5913	C	0.093
5914	A	0.107
5915	C	0.027
5916	C	0.093
5917	A	0.479
5918	C	0.093
5919	U	0.826
5920	C	0.293
5921	U	0.999
5922	A	1.572
5923	U	0.639
5924	U	0.613
5925	U	0.706
5926	U	0.559
5927	G	1.052
5928	U	0.253
5929	G	0.173
5930	C	0.12

5931	A	0.213
5932	U	0.12
5933	C	0.08
5934	A	0.28
5935	G	0.293
5936	A	0.213
5937	U	0.133
5938	G	0
5939	C	1.305
5940	U	2.118
5941	A	1.066
5942	A	1.478
5943	A	0.04
5944	G	0.027
5945	C	0.12
5946	A	0.04
5947	U	0.599
5948	A	0.093
5949	U	0.226
5950	G	0.506
5951	A	0.306
5952	U	0.626
5953	A	0.16
5954	C	0.919
5955	A	1.385
5956	G	1.172
5957	A	0.759
5958	G	0.373
5959	G	0.24
5960	U	0.559
5961	A	0.173
5962	C	1.758
5963	A	0.959
5964	U	1.465
5965	A	1.292
5966	A	0.759
5967	U	1.252
5968	G	0.639
5969	U	0.653
5970	U	0.373
5971	U	0.213
5972	G	0.067
5973	G	0.053

5974	G	0
5975	C	0
5976	C	0.013
5977	A	0.12
5978	C	0
5979	A	0.226
5980	C	0.107
5981	A	1.066
5982	U	0.679
5983	G	0.559
5984	C	0.147
5985	C	0
5986	U	0.08
5987	G	0
5988	U	0.027
5989	G	0.2
5990	U	0.639
5991	A	0.28
5992	C	0
5993	C	0
5994	C	0.013
5995	A	0.08
5996	C	0
5997	A	0.733
5998	G	0.932
5999	A	0.546
6000	C	0
6001	C	0
6002	C	0
6003	C	0.053
6004	A	0.226
6005	A	0.306
6006	C	0.04
6007	C	0
6008	C	0
6009	A	0.013
6010	C	0.04
6011	A	0.226
6012	A	0.186
6013	G	0.44
6014	A	1.225
6015	A	1.811
6016	G	0.986

6017	U	0.533
6018	A	0.839
6019	G	0.533
6020	U	0.107
6021	A	0.613
6022	U	0.373
6023	U	0.426
6024	G	0.293
6025	G	0.32
6026	U	0.173
6027	A	0.826
6028	A	1.066
6029	A	0.892
6030	U	0.413
6031	G	0.786
6032	U	0.36
6033	G	0.919
6034	A	0.919
6035	C	0.147
6036	A	1.958
6037	G	0.466
6038	A	1.252
6039	A	1.026
6040	A	0.852
6041	A	0.706
6042	U	0.386
6043	U	0.413
6044	U	0.44
6045	U	0.506
6046	A	0.919
6047	A	0.653
6048	C	0.12
6049	A	0.333
6050	U	0.173
6051	G	0.373
6052	U	0.226
6053	G	0.986
6054	G	0.719
6055	A	1.132
6056	A	1.079
6057	A	0.972
6058	A	1.092
6059	A	1.172

6060	U	1.039
6061	G	1.132
6062	A	0.493
6063	C	0.12
6064	A	0.346
6065	U	0.213
6066	G	0.147
6067	G	0.4
6068	U	0.24
6069	A	1.079
6070	G	1.079
6071	A	0.812
6072	A	0.533
6073	C	0.12
6074	A	0.666
6075	G	0.333
6076	A	0.693
6077	U	0.373
6078	G	0.733
6079	C	0.173
6080	A	0.999
6081	U	0.533
6082	G	1.239
6083	A	0.706
6084	G	0.586
6085	G	0.533
6086	A	0.706
6087	U	0.12
6088	A	1.398
6089	U	0.666
6090	A	1.012
6091	A	0.919
6092	U	0.44
6093	C	0.213
6094	A	0.693
6095	G	0.653
6096	U	0.506
6097	U	0.546
6098	U	0.746
6099	A	1.265
6100	U	0.546
6101	G	0.519
6102	G	0.306

6103	G	0.24
6104	A	0.613
6105	U	0.2
6106	C	0.373
6107	A	0.932
6108	A	0.892
6109	A	1.319
6110	G	0.346
6111	C	0.027
6112	C	0.067
6113	U	0.653
6114	A	1.398
6115	A	1.172
6116	A	0.972
6117	G	0.266
6118	C	0
6119	C	0.027
6120	A	0.24
6121	U	0.346
6122	G	0.4
6123	U	0.16
6124	G	0.706
6125	U	0.226
6126	A	1.039
6127	A	0.946
6128	A	0.946
6129	A	0.626
6130	U	0.4
6131	U	0.892
6132	A	1.039
6133	A	0.733
6134	C	0.186
6135	C	0.08
6136	C	0.013
6137	C	0.04
6138	A	0.16
6139	C	0.053
6140	U	0.306
6141	C	0.413
6142	U	0.586
6143	G	0.879
6144	U	0.493
6145	G	0.28

6146	U	0.333
6147	U	0.519
6148	A	0.919
6149	G	0.586
6150	U	0.333
6151	U	0.786
6152	U	1.425
6153	A	1.265
6154	A	1.305
6155	A	1.012
6156	G	0.479
6157	U	0.253
6158	G	0.36
6159	C	0.173
6160	A	0.426
6161	C	0.107
6162	U	0.466
6163	G	0.773
6164	A	0.719
6165	U	0.506
6166	U	1.052
6167	U	0.733
6168	G	1.132
6169	A	1.292
6170	A	1.292
6171	G	1.145
6172	A	1.172
6173	A	1.252
6174	U	0.626
6175	G	0.719
6176	A	0.879
6177	U	0.493
6178	A	0.32
6179	C	0.053
6180	U	0.4
6181	A	0.733
6182	A	1.079
6183	U	0.599
6184	A	0.533
6185	C	0.173
6186	C	0.306
6187	A	0.533
6188	A	0.852

6189	U	0.586
6190	A	0.799
6191	G	0.746
6192	U	0.719
6193	A	0.733
6194	G	0.573
6195	U	0.679
6196	A	0.879
6197	G	0.719
6198	C	0.253
6199	G	0.386
6200	G	0.253
6201	G	0.559
6202	A	0.759
6203	G	0.786
6204	A	0.852
6205	A	1.145
6206	U	0.546
6207	G	0.626
6208	A	0.866
6209	U	0.559
6210	A	0.946
6211	A	0.879
6212	U	0.586
6213	G	0.613
6214	G	0.919
6215	A	1.159
6216	G	0.852
6217	A	1.225
6218	A	1.066
6219	A	0.946
6220	G	0.653
6221	G	1.012
6222	A	0.812
6223	G	0.892
6224	A	0.906
6225	G	0.946
6226	A	2.131
6227	U	0.107
6228	A	1.825
6229	A	1.385
6230	A	0.999
6231	A	1.159

6232	A	1.145
6233	A	0.719
6234	C	0.16
6235	U	0.12
6236	G	0.306
6237	C	0.053
6238	U	0.013
6239	C	0.013
6240	U	0.147
6241	U	0.266
6242	U	0.346
6243	C	0.213
6244	A	1.105
6245	A	1.385
6246	U	0.613
6247	A	0.719
6248	U	0.213
6249	C	0.293
6250	A	0.693
6251	G	0.626
6252	C	0.533
6253	A	0.613
6254	C	0.093
6255	A	0.919
6256	A	0.839
6257	G	0.559
6258	C	0.24
6259	A	0.932
6260	U	0.599
6261	A	1.145
6262	A	0.986
6263	G	1.279
6264	A	1.132
6265	G	0.906
6266	A	1.438
6267	U	0.972
6268	A	1.239
6269	A	1.026
6270	G	0.613
6271	G	0.107
6272	U	0.08
6273	G	0.013
6274	C	0.093

6275	A	0.733
6276	G	0.706
6277	A	0.786
6278	A	1.026
6279	A	0.906
6280	G	0.812
6281	A	0.706
6282	A	0.812
6283	U	0.666
6284	A	3.037
6285	U	0.466
6286	G	0.346
6287	C	0.213
6288	A	0.346
6289	U	0.28
6290	U	0.453
6291	C	0.12
6292	U	0.16
6293	U	0.32
6294	U	0.999
6295	U	0.839
6296	A	1.865
6297	U	0.506
6298	A	1.705
6299	A	2.331
6300	A	0.533
6301	C	0.16
6302	U	0.346
6303	U	0.546
6304	G	1.079
6305	A	1.558
6306	U	0.386
6307	A	1.359
6308	U	0.679
6309	A	1.279
6310	G	1.345
6311	U	0.546
6312	A	0.586
6313	C	0.16
6314	C	0.173
6315	A	0.479
6316	A	0.799
6317	U	0.533

6318	A	0.866
6319	G	0.546
6320	A	1.319
6321	U	0.919
6322	A	1.252
6323	A	1.305
6324	U	0.786
6325	A	0.746
6326	C	0.12
6327	C	0.266
6328	A	0.866
6329	G	0.493
6330	C	0.28
6331	U	0.386
6332	A	0.213
6333	U	0.493
6334	A	0.999
6335	G	0.266
6336	G	0.12
6337	U	0.306
6338	U	0.626
6339	G	0.826
6340	A	1.079
6341	U	0.919
6342	A	0.986
6343	A	0.946
6344	G	0.666
6345	U	0.266
6346	U	0.333
6347	G	1.119
6348	U	0.839
6349	A	1.385
6350	A	0.786
6351	C	0.333
6352	A	0.733
6353	C	0.173
6354	C	0.107
6355	U	0.226
6356	C	0.226
6357	A	0.799
6358	G	0.413
6359	U	0.253
6360	C	0.746

6361	A	0.599
6362	U	0.493
6363	U	0.679
6364	A	1.572
6365	C	0.253
6366	A	0.746
6367	C	0.346
6368	A	1.279
6369	G	1.505
6370	G	0.386
6371	C	0.04
6372	C	0
6373	U	0.16
6374	G	0.173
6375	U	0.147
6376	C	0.12
6377	C	0.226
6378	A	0.932
6379	A	1.825
6380	A	1.678
6381	G	0.773
6382	G	0.453
6383	U	0.186
6384	A	0.733
6385	U	0.253
6386	C	0.213
6387	C	0.053
6388	U	0.266
6389	U	0.906
6390	U	1.145
6391	G	1.066
6392	A	0.559
6393	G	0.053
6394	C	0.08
6395	C	0.12
6396	A	0.186
6397	A	0.16
6398	U	0.107
6399	U	0.067
6400	C	0.027
6401	C	0.013
6402	C	0.053
6403	A	0.693

6404	U	0.586
6405	A	0.679
6406	C	0.293
6407	A	1.145
6408	U	0.892
6409	U	0.693
6410	A	1.558
6411	U	0.693
6412	U	0.826
6413	G	1.345
6414	U	0.426
6415	G	0
6416	C	0.027
6417	C	0.053
6418	C	0.013
6419	C	0.639
6420	G	1.079
6421	G	0.053
6422	C	0
6423	U	0.107
6424	G	0.133
6425	G	0.12
6426	U	0.093
6427	U	0
6428	U	0
6429	U	0
6430	G	0
6431	C	0.493
6432	G	0
6433	A	0
6434	U	0.04
6435	U	0.04
6436	C	0.027
6437	U	0.746
6438	A	1.585
6439	A	0.959
6440	A	0.773
6441	A	0.799
6442	U	0.32
6443	G	1.292
6444	U	0.559
6445	A	1.239
6446	A	1.252

6447	U	0.932
6448	A	1.452
6449	A	1.372
6450	U	1.026
6451	A	1.385
6452	A	1.252
6453	G	1.185
6454	A	1.026
6455	C	0.426
6456	G	0.306
6457	U	0.173
6458	U	0.253
6459	C	0.16
6460	A	1.012
6461	A	0.906
6462	U	0.519
6463	G	0.959
6464	G	0.506
6465	A	0.626
6466	A	0.706
6467	C	0.226
6468	A	1.252
6469	G	0.839
6470	G	0.773
6471	A	0.679
6472	C	0.186
6473	C	0.147
6474	A	0.466
6475	U	0.173
6476	G	0.44
6477	U	0.24
6478	A	0.559
6479	C	0.186
6480	A	0.639
6481	A	0.719
6482	A	0.999
6483	U	0.479
6484	G	0.373
6485	U	0.213
6486	C	0.266
6487	A	0.852
6488	G	0.479
6489	C	0.253

6490	A	0.879
6491	C	0.293
6492	A	1.026
6493	G	0.4
6494	U	0.453
6495	A	0.533
6496	C	0.133
6497	A	0.666
6498	A	0.879
6499	U	0.386
6500	G	0.759
6501	U	0.306
6502	A	0.546
6503	C	0.186
6504	A	0.852
6505	C	0.4
6506	A	0.386
6507	U	0.12
6508	G	0.133
6509	G	0.107
6510	A	0.493
6511	A	0.826
6512	U	0.479
6513	C	0.373
6514	A	0.626
6515	G	0.293
6516	G	0.147
6517	C	0.04
6518	C	0.186
6519	A	0.519
6520	G	0.426
6521	U	0.413
6522	A	0.653
6523	G	0.533
6524	U	0.4
6525	A	1.132
6526	U	0.346
6527	C	0.466
6528	A	0.906
6529	A	0.693
6530	C	0.253
6531	U	0.413
6532	C	0.426

6533	A	0.906
6534	A	0.426
6535	C	0.453
6536	U	0.333
6537	G	0.266
6538	C	0.053
6539	U	0.067
6540	G	0.226
6541	U	0.373
6542	U	0.706
6543	A	0.706
6544	A	0.613
6545	A	0.786
6546	U	0.44
6547	G	0.067
6548	G	0.08
6549	C	0.067
6550	A	0.093
6551	G	0.067
6552	U	0.053
6553	C	0.053
6554	U	0.24
6555	A	0.573
6556	G	0.613
6557	C	0.28
6558	A	0.879
6559	G	1.052
6560	A	1.159
6561	A	1.132
6562	G	1.225
6563	A	1.319
6564	A	1.066
6565	G	1.039
6566	A	1.558
6567	U	0.4
6568	G	0.959
6569	U	0.799
6570	A	1.172
6571	G	1.079
6572	U	0.812
6573	A	1.292
6574	A	1.518
6575	U	1.052

6576	U	0.413
6577	A	0.479
6578	G	0.333
6579	A	0.573
6580	U	0.506
6581	C	0.093
6582	U	0.053
6583	G	0.027
6584	C	0.04
6585	C	0.067
6586	A	0.573
6587	A	0.453
6588	U	0.2
6589	U	0.226
6590	U	0.32
6591	C	0.16
6592	A	0.266
6593	C	0.107
6594	A	0.32
6595	G	0.32
6596	A	0.786
6597	C	0.16
6598	A	0.812
6599	A	1.092
6600	U	0.44
6601	G	0.253
6602	C	0.133
6603	U	0.546
6604	A	1.145
6605	A	0.932
6606	A	0.946
6607	A	0.613
6608	C	0.107
6609	C	0.28
6610	A	0.693
6611	U	0.4
6612	A	0.972
6613	A	1.398
6614	U	0.972
6615	A	1.199
6616	G	0.4
6617	U	0.16
6618	A	0.266

6619	C	0.186
6620	A	0.533
6621	G	0.133
6622	C	0.08
6623	U	0.2
6624	G	0.533
6625	A	0.879
6626	A	1.132
6627	C	0.306
6628	A	0.839
6629	C	0.32
6630	A	0.599
6631	U	0.2
6632	C	0.28
6633	U	0.186
6634	G	0.546
6635	U	0.306
6636	A	2.544
6637	G	1.891
6638	A	1.465
6639	A	0.826
6640	A	0.892
6641	U	0.653
6642	U	0.932
6643	A	1.492
6644	A	1.492
6645	U	0.453
6646	U	0.333
6647	G	0.666
6648	U	0.226
6649	A	0.773
6650	C	0.093
6651	A	0.892
6652	A	1.265
6653	G	0.932
6654	A	0.719
6655	C	0.067
6656	C	0.067
6657	C	0.12
6658	A	0.892
6659	A	0.519
6660	C	0.226
6661	A	0.999

6662	A	0.786
6663	C	0.213
6664	A	0.706
6665	A	0.839
6666	U	0.466
6667	A	0.826
6668	C	0.12
6669	A	0.972
6670	A	1.279
6671	G	1.412
6672	A	1.172
6673	A	1.012
6674	A	1.066
6675	A	1.039
6676	A	1.279
6677	G	0.693
6678	U	0.413
6679	A	0.613
6680	U	0.253
6681	C	0.16
6682	C	0.32
6683	G	0.972
6684	U	0.666
6685	A	1.159
6686	U	0.16
6687	C	0.12
6688	C	0.093
6689	A	0.666
6690	G	0.613
6691	A	0.679
6692	G	0.32
6693	G	0.253
6694	G	0.226
6695	G	0.226
6696	A	0.453
6697	C	0.133
6698	C	0.173
6699	A	0.626
6700	G	0.36
6701	G	0.373
6702	G	0.453
6703	A	0.626
6704	G	0.852

6705	A	0.972
6706	G	0.613
6707	C	0.226
6708	A	0.546
6709	U	0.373
6710	U	0.266
6711	U	0.186
6712	G	0.067
6713	U	0.04
6714	U	0.12
6715	A	0.306
6716	C	0.133
6717	A	0.373
6718	A	0.32
6719	U	0.053
6720	A	1.398
6721	G	1.026
6722	G	0.653
6723	A	0.986
6724	A	0.812
6725	A	0.653
6726	A	0.852
6727	A	0.679
6728	U	0.186
6729	A	1.066
6730	G	0.879
6731	G	1.252
6732	A	0.986
6733	A	0.666
6734	A	0.972
6735	U	0.2
6736	A	1.105
6737	U	0.306
6738	G	1.305
6739	A	0.999
6740	G	0.852
6741	A	0.959
6742	C	0.12
6743	A	0.493
6744	A	0.533
6745	G	0.546
6746	C	0.173
6747	A	0.879

6748	C	0.08
6749	A	0.093
6750	U	0.027
6751	U	0.093
6752	G	0.067
6753	U	0.04
6754	A	0.253
6755	A	0.413
6756	C	0
6757	A	0.559
6758	U	0.426
6759	U	0.453
6760	A	0.879
6761	G	0.479
6762	U	0.133
6763	A	1.239
6764	G	0.932
6765	A	0.986
6766	G	0.36
6767	C	0
6768	A	0.959
6769	A	0.28
6770	A	0.879
6771	A	0.879
6772	U	0.36
6773	G	0.679
6774	G	0.599
6775	A	0.666
6776	A	0.812
6777	U	0.293
6778	G	0.12
6779	C	0.013
6780	C	0.067
6781	A	0.386
6782	C	0.04
6783	U	0.4
6784	U	0.653
6785	U	0.919
6786	A	1.132
6787	A	0.773
6788	A	0.839
6789	A	0.626
6790	C	0

6791	A	0.866
6792	G	0.613
6793	A	0.972
6794	U	0.573
6795	A	1.239
6796	G	0.253
6797	C	0.067
6798	U	0.173
6799	A	0.946
6800	G	0.759
6801	C	0
6802	A	0.586
6803	A	0.559
6804	A	0.599
6805	U	0.293
6806	U	0.4
6807	A	1.026
6808	A	0.906
6809	G	1.052
6810	A	1.066
6811	G	0.879
6812	A	0.866
6813	A	0.839
6814	C	0.213
6815	A	0.519
6816	A	0.493
6817	U	0.333
6818	U	0.453
6819	U	0.413
6820	G	0.746
6821	G	1.265
6822	A	1.172
6823	A	0.879
6824	A	1.066
6825	U	0.493
6826	A	1.105
6827	A	0.972
6828	U	0.626
6829	A	1.438
6830	A	0.932
6831	A	1.145
6832	A	1.185
6833	C	0

6834	A	0.693
6835	A	1.066
6836	U	0.44
6837	A	0.986
6838	A	1.039
6839	U	0.373
6840	C	0.2
6841	U	0.306
6842	U	0.653
6843	U	0.812
6844	A	1.292
6845	A	2.051
6846	G	0.613
6847	C	0.2
6848	A	0.759
6849	A	1.092
6850	U	0.16
6851	C	0
6852	C	0.027
6853	U	0.133
6854	C	0.093
6855	A	1.092
6856	G	0.719
6857	G	0.719
6858	A	1.092
6859	G	1.159
6860	G	1.305
6861	G	0.08
6862	G	0.053
6863	A	0.2
6864	C	0.04
6865	C	0.08
6866	C	0.12
6867	A	0.693
6868	G	0.573
6869	A	0.653
6870	A	0.733
6871	A	0.466
6872	U	0.266
6873	U	0.266
6874	G	0.4
6875	U	0.293
6876	A	0.959

6877	A	0.932
6878	C	0.413
6879	G	0.746
6880	C	0.16
6881	A	0.733
6882	C	0.16
6883	A	0.546
6884	G	0.466
6885	U	0.28
6886	U	0.346
6887	U	0.613
6888	U	0.613
6889	A	1.039
6890	A	1.239
6891	U	0.626
6892	U	0.466
6893	G	0.36
6894	U	0.986
6895	G	0.093
6896	G	0.107
6897	A	0.107
6898	G	0.04
6899	G	0.04
6900	G	0.067
6901	G	0.12
6902	A	0.839
6903	A	1.119
6904	U	0.213
6905	U	0.067
6906	U	0.067
6907	U	0.067
6908	U	0.027
6909	C	0.013
6910	U	0.053
6911	A	0.16
6912	C	0.027
6913	U	0.36
6914	G	1.225
6915	U	1.279
6916	A	0.986
6917	A	0.786
6918	U	0.533
6919	U	0.599

6920	C	1.385
6921	A	0.812
6922	A	0.852
6923	C	0.067
6924	A	0.373
6925	C	0.133
6926	A	0.586
6927	A	1.225
6928	C	0.306
6929	U	0.373
6930	G	0.293
6931	U	0.2
6932	U	0.426
6933	U	0.613
6934	A	1.332
6935	A	1.891
6936	U	1.438
6937	A	0.253
6938	G	0.4
6939	U	0.186
6940	A	0.373
6941	C	0.053
6942	U	0.386
6943	U	0.812
6944	G	0.812
6945	G	0.693
6946	U	0.453
6947	U	0.746
6948	U	0.746
6949	A	1.159
6950	A	1.865
6951	U	1.265
6952	A	0.186
6953	G	0.346
6954	U	0.16
6955	A	0.133
6956	C	0.133
6957	U	2.677
6958	U	1.465
6959	G	1.731
6960	G	1.332
6961	A	0.879
6962	G	0.226

6963	U	0.413
6964	A	0.839
6965	C	0.027
6966	U	0.373
6967	G	0.693
6968	A	0.679
6969	A	0.812
6970	G	0.293
6971	G	0.107
6972	G	0.186
6973	U	0.12
6974	C	0.373
6975	A	1.265
6976	A	0.932
6977	A	1.532
6978	U	0.906
6979	A	1.359
6980	A	1.092
6981	C	0.32
6982	A	0.453
6983	C	0.133
6984	U	0.333
6985	G	0.533
6986	A	1.172
6987	A	1.079
6988	G	0.32
6989	G	0.413
6990	A	0.999
6991	A	0.44
6992	G	0.173
6993	U	0.213
6994	G	0.626
6995	A	0.266
6996	C	1.079
6997	A	0.133
6998	C	0.679
6999	A	0.746
7000	A	0.346
7001	U	0.293
7002	C	0.519
7003	A	0.08
7004	C	0.213
7005	A	0.186

7006	C	0.12
7007	U	0.027
7008	C	0.053
7009	C	0.12
7010	C	0.839
7011	A	0.666
7012	U	0.426
7013	G	0.293
7014	C	0.493
7015	A	0.413
7016	G	0.826
7017	A	1.452
7018	A	0.746
7019	U	1.332
7020	A	0.919
7021	A	0.906
7022	A	0.879
7023	A	0.346
7024	C	0.839
7025	A	0.866
7026	A	0.613
7027	U	0.826
7028	U	0.826
7029	U	1.958
7030	A	0.773
7031	U	0.919
7032	A	0.479
7033	A	0.586
7034	A	0.44
7035	C	0.586
7036	A	0.32
7037	U	0.253
7038	G	0.093
7039	U	0.133
7040	G	0.386
7041	G	0.426
7042	C	0.733
7043	A	0.586
7044	G	1.105
7045	G	1.465
7046	A	0.32
7047	A	0.093
7048	G	0.12

7049	U	0.293
7050	A	0.333
7051	G	0.16
7052	G	0.28
7053	A	0.213
7054	A	0.28
7055	A	0.546
7056	A	0.946
7057	G	0.413
7058	C	0.946
7059	A	0.986
7060	A	0.253
7061	U	1.252
7062	G	0.626
7063	U	1.425
7064	A	0.759
7065	U	0.44
7066	G	0.013
7067	C	0
7068	C	0.027
7069	C	0.053
7070	C	0.093
7071	U	0.08
7072	C	0.04
7073	C	0.2
7074	C	0.812
7075	A	0.293
7076	U	0.653
7077	C	0.812
7078	A	0.36
7079	G	0.147
7080	U	0.08
7081	G	0.093
7082	G	0.213
7083	A	0.053
7084	C	0.373
7085	A	0.613
7086	A	0.706
7087	A	0.426
7088	U	0.613
7089	U	0.906
7090	A	0.986
7091	G	1.012

7092	A	0.16
7093	U	0
7094	G	0
7095	U	0.08
7096	U	0.16
7097	C	0.32
7098	A	0.573
7099	U	0.879
7100	C	1.079
7101	A	0.639
7102	A	1.092
7103	A	0.799
7104	U	0.679
7105	A	0.559
7106	U	0.693
7107	U	0.44
7108	A	0.067
7109	C	0.186
7110	U	0.213
7111	G	0
7112	G	0
7113	G	0
7114	C	0.133
7115	U	0.173
7116	G	0.16
7117	C	0.413
7118	U	0.4
7119	A	0.027
7120	U	0.839
7121	U	1.132
7122	A	0.706
7123	A	0.426
7124	C	0.879
7125	A	0.852
7126	A	1.012
7127	G	1.012
7128	A	0.719
7129	G	0.706
7130	A	0.28
7131	U	0.186
7132	G	0.253
7133	G	0.107
7134	U	0.133

7135	G	0.147
7136	G	0.44
7137	U	0.999
7138	A	1.159
7139	A	0.866
7140	U	1.159
7141	A	0.626
7142	A	0.253
7143	C	0.546
7144	A	0.573
7145	A	0.253
7146	C	0.586
7147	A	0.972
7148	A	0.546
7149	U	0.04
7150	G	0
7151	G	0
7152	G	0
7153	U	0
7154	C	0.013
7155	C	1.185
7156	G	0.706
7157	A	0.599
7158	G	0.693
7159	A	0.226
7160	U	0.24
7161	C	0.639
7162	U	0.546
7163	U	0.32
7164	C	0.812
7165	A	0.826
7166	G	0.866
7167	A	0
7168	C	0
7169	C	0
7170	U	0.479
7171	G	0.16
7172	G	0.186
7173	A	0.013
7174	G	0.093
7175	G	0.067
7176	A	0.28
7177	G	0.24

7178	G	0
7179	C	0.453
7180	G	0.799
7181	A	0.573
7182	U	1.319
7183	A	0.373
7184	U	0.892
7185	G	0.44
7186	A	0.186
7187	G	0.067
7188	G	0.213
7189	G	0.506
7190	A	0.213
7191	C	0.586
7192	A	0.613
7193	A	0.493
7194	U	0.533
7195	U	0.706
7196	G	0.946
7197	G	1.012
7198	A	1.039
7199	G	0.879
7200	A	0.826
7201	A	1.052
7202	G	0.479
7203	U	1.145
7204	G	0.919
7205	A	0.986
7206	A	0.28
7207	U	1.066
7208	U	0.773
7209	A	0.2
7210	U	1.612
7211	A	0.493
7212	U	1.212
7213	A	0.866
7214	A	1.185
7215	A	0.839
7216	U	1.332
7217	A	0.626
7218	U	1.159
7219	A	0.719
7220	A	0.986

7221	A	1.252
7222	G	1.159
7223	U	1.359
7224	A	1.092
7225	G	1.105
7226	U	1.279
7227	A	1.119
7228	A	1.345
7229	A	1.132
7230	A	0.666
7231	A	0.613
7232	U	0.653
7233	U	1.092
7234	G	0.986
7235	A	0.719
7236	A	0.12
7237	C	0.12
7238	C	0.746
7239	A	0.519
7240	U	1.212
7241	U	1.345
7242	A	0.719
7243	G	0.866
7244	G	0.759
7245	A	1.026
7246	G	0.506
7247	U	0.413
7248	A	0.12
7249	G	0.133
7250	C	0.346
7251	A	0.133
7252	C	0.173
7253	C	0.4
7254	C	0.413
7255	A	0.027
7256	C	0.027
7257	C	0.067
7258	A	0.107
7259	A	0.173
7260	G	0.08
7261	G	0.08
7262	C	0.107
7263	A	0.506

7264	A	1.185
7265	A	1.385
7266	G	0.999
7267	A	0.999
7268	G	1.265
7269	A	2.091
7270	A	2.304
7271	G	2.531
7272	A	0.08
7273	G	0.027
7274	U	0.013
7275	G	0
7276	G	0.067
7277	U	0.12
7278	G	0
7279	C	0.08
7280	A	0.053
7281	G	0.186
7282	A	0.107
7283	G	0
7284	A	0.04
7285	G	0.186
7286	A	0.533
7287	A	0.346
7288	A	0.386
7289	A	0.746
7290	A	0.932
7291	A	0.08
7292	G	0.053
7293	A	0.08
7294	G	0.053
7295	C	0.04
7296	A	0.133
7297	G	0.173
7298	U	0.12
7299	G	0.053
7300	G	0.373
7301	G	0.746
7302	A	1.678
7303	A	2.824
7304	U	3.583
7305	A	0
7306	G	0

7307	G	0.053
7308	A	0.133
7309	G	0.053
7310	C	0.093
7311	U	0.519
7312	U	0.253
7313	U	0
7314	G	0.067
7315	U	0
7316	U	0.067
7317	C	0.027
7318	C	0.04
7319	U	0
7320	U	0.213
7321	G	0.04
7322	G	0.013
7323	G	0.147
7324	U	0.013
7325	U	0.107
7326	C	0
7327	U	0.107
7328	U	0.173
7329	G	2.384
7330	G	5.341
7331	G	0.999
7332	A	1.252
7333	G	0
7334	C	0.04
7335	A	0.013
7336	G	0.067
7337	C	0
7338	A	0.12
7339	G	0.173
7340	G	0.107
7341	A	1.012
7342	A	2.437
7343	G	0
7344	C	0
7345	A	0.08
7346	C	0.013
7347	U	0.173
7348	A	0.919
7349	U	0.493

7350	G	1.572
7351	G	0.599
7352	G	0.959
7353	C	0.08
7354	U	0.2
7355	G	0.12
7356	C	0
7357	A	0.173
7358	C	0.027
7359	G	0.053
7360	U	0
7361	C	0.147
7362	A	2.917
7363	A	2.064
7364	U	1.052
7365	G	0.133
7366	A	0.093
7367	C	0.027
7368	G	0.173
7369	C	0.027
7370	U	0.027
7371	G	0.12
7372	A	2.65
7373	C	0
7374	G	0.147
7375	G	1.185
7376	U	1.212
7377	A	1.505
7378	C	0.08
7379	A	0.972
7380	G	1.092
7381	G	0.839
7382	C	0.266
7383	C	0
7384	A	0.107
7385	G	0.053
7386	A	0.067
7387	C	0
7388	A	0.093
7389	A	0.586
7390	U	0.4
7391	U	0.679
7392	A	1.731

7393	U	0.946
7394	U	0.226
7395	G	0.027
7396	U	0.067
7397	C	0
7398	U	0.053
7399	G	0.666
7400	A	2.331
7401	U	0.959
7402	A	0.932
7403	U	0.186
7404	A	0.306
7405	G	0.053
7406	U	0.013
7407	G	0.093
7408	C	0.24
7409	A	1.185
7410	G	0.293
7411	C	0
7412	A	0.16
7413	G	0.133
7414	C	0
7415	A	0.226
7416	G	0.44
7417	A	0.679
7418	A	0.812
7419	C	0.027
7420	A	1.066
7421	A	1.066
7422	U	0.573
7423	U	0.812
7424	U	0.333
7425	G	0.08
7426	C	0
7427	U	0.04
7428	G	0.173
7429	A	0.826
7430	G	1.092
7431	G	1.132
7432	G	0.466
7433	C	0.093
7434	U	0.799
7435	A	2.677

7436	U	1.612
7437	U	0.559
7438	G	0.28
7439	A	0.559
7440	G	0.24
7441	G	0.147
7442	C	0.519
7443	G	0.04
7444	C	0.12
7445	A	0.426
7446	A	0.493
7447	C	0.04
7448	A	0.04
7449	G	0.04
7450	C	0.36
7451	A	1.052
7452	U	0.679
7453	C	0.24
7454	U	0.053
7455	G	0.027
7456	U	0.013
7457	U	0.08
7458	G	0
7459	C	0.013
7460	A	0.839
7461	A	0.559
7462	C	0.173
7463	U	0.253
7464	C	0.226
7465	A	1.319
7466	C	0.852
7467	A	1.492
7468	G	0
7469	U	0.04
7470	C	0.013
7471	U	0.067
7472	G	0.067
7473	G	0.013
7474	G	0.067
7475	G	0.013
7476	C	0.013
7477	A	0.679
7478	U	0.786

7479	C	0.839
7480	A	0.946
7481	A	0.812
7482	A	0.799
7483	C	0.36
7484	A	1.052
7485	G	0.013
7486	C	0.04
7487	U	0.053
7488	C	0.053
7489	C	0.027
7490	A	0.12
7491	G	0.173
7492	G	0.04
7493	C	0.12
7494	A	0.852
7495	A	1.398
7496	G	0.999
7497	A	1.105
7498	A	1.119
7499	U	0.147
7500	C	0.04
7501	C	0
7502	U	0.147
7503	G	1.212
7504	G	1.705
7505	C	0.04
7506	U	0.027
7507	G	0.053
7508	U	0.16
7509	G	0.746
7510	G	1.292
7511	A	1.279
7512	A	0.093
7513	A	0.027
7514	G	0
7515	A	0.24
7516	U	0.426
7517	A	0.08
7518	C	0.013
7519	C	0.027
7520	U	0.147
7521	A	1.532

7522	A	0.213
7523	A	0.107
7524	G	0.013
7525	G	0
7526	A	0.053
7527	U	0.027
7528	C	0.027
7529	A	0.067
7530	A	0.639
7531	C	0.36
7532	A	0.027
7533	G	0.027
7534	C	0
7535	U	0.027
7536	C	0.027
7537	C	0.04
7538	U	0.186
7539	G	0.639
7540	G	0.133
7541	G	0.027
7542	G	0.093
7543	A	0.826
7544	U	0.36
7545	U	0.506
7546	U	0.24
7547	G	0.08
7548	G	0.053
7549	G	0.093
7550	G	0.36
7551	U	0.28
7552	U	0.08
7553	G	0.013
7554	C	0.027
7555	U	0.04
7556	C	0.04
7557	U	0.093
7558	G	1.052
7559	G	0.946
7560	A	0.506
7561	A	0.413
7562	A	0.293
7563	A	0.386
7564	C	0.013

7565	U	0.027
7566	C	0.053
7567	A	1.438
7568	U	0.293
7569	U	0.16
7570	U	0.027
7571	G	0.013
7572	C	0
7573	A	0.04
7574	C	0.04
7575	C	0.107
7576	A	0.027
7577	C	0.093
7578	U	0.386
7579	G	0.826
7580	C	0.08
7581	U	0.147
7582	G	0.559
7583	U	0.133
7584	G	0.067
7585	C	0.107
7586	C	0.186
7587	U	0.16
7588	U	0.186
7589	G	0.133
7590	G	0.213
7591	A	0.613
7592	A	0.773
7593	U	0.213
7594	G	0.12
7595	C	0.12
7596	U	0.16
7597	A	0.666
7598	G	0.773
7599	U	0.4
7600	U	0.493
7601	G	0.533
7602	G	0.653
7603	A	0.693
7604	G	0.306
7605	U	0.2
7606	A	0.626
7607	A	0.693

7608	U	0.653
7609	A	0.826
7610	A	0.559
7611	A	0.946
7612	U	0.16
7613	C	0.08
7614	U	0.08
7615	C	0.04
7616	U	0.12
7617	G	0.44
7618	G	0.386
7619	A	0.506
7620	A	0.493
7621	C	0.133
7622	A	0.759
7623	G	0.799
7624	A	0.799
7625	U	0.253
7626	U	0.812
7627	U	0.32
7628	G	0.546
7629	G	0.586
7630	A	0.679
7631	A	0.746
7632	U	0.413
7633	A	0.906
7634	A	0.826
7635	C	0.12
7636	A	0.679
7637	U	0.293
7638	G	0.546
7639	A	0.426
7640	C	0.067
7641	C	0.067
7642	U	0.213
7643	G	0.253
7644	G	0.36
7645	A	0.386
7646	U	0.226
7647	G	0.12
7648	G	0.493
7649	A	0.626
7650	G	0.506

7651	U	0.08
7652	G	0.173
7653	G	0.253
7654	G	0.333
7655	A	0.493
7656	C	0.107
7657	A	0.413
7658	G	0.253
7659	A	0.653
7660	G	0.333
7661	A	0.546
7662	A	0.626
7663	A	0.706
7664	U	0.426
7665	U	0.466
7666	A	0.773
7667	A	0.719
7668	C	0.213
7669	A	0.626
7670	A	0.453
7671	U	0.226
7672	U	0.373
7673	A	0.773
7674	C	0.093
7675	A	0.653
7676	C	0.133
7677	A	0.879
7678	A	0.839
7679	G	0.733
7680	C	0.133
7681	U	0.44
7682	U	0.546
7683	A	0.519
7684	A	0.493
7685	U	0.373
7686	A	0.746
7687	C	0.12
7688	A	0.093
7689	C	0.107
7690	U	0.16
7691	C	0.067
7692	C	0.067
7693	U	0.932

7694	U	1.572
7695	A	1.225
7696	A	0.746
7697	U	0.306
7698	U	0.453
7699	G	1.079
7700	A	1.066
7701	A	0.426
7702	G	0.013
7703	A	0.067
7704	A	0.067
7705	U	0.013
7706	C	0.04
7707	G	0.013
7708	C	0.013
7709	A	0.067
7710	A	0.053
7711	A	0.12
7712	A	0.053
7713	C	0.013
7714	C	0.067
7715	A	0
7716	G	0.04
7717	C	0.08
7718	A	1.052
7719	A	1.252
7720	G	0.719
7721	A	0.879
7722	A	0.639
7723	A	0.799
7724	A	0.959
7725	G	1.026
7726	A	0.773
7727	A	0.679
7728	U	0.333
7729	G	0.839
7730	A	0.773
7731	A	0.746
7732	C	0.173
7733	A	0.479
7734	A	0.506
7735	G	0.746
7736	A	0.946

7737	A	0.879
7738	U	0.386
7739	U	0.559
7740	A	2.198
7741	U	0.373
7742	U	0.346
7743	G	0.586
7744	G	0.706
7745	A	0.786
7746	A	0.613
7747	U	0.333
7748	U	0.746
7749	A	1.425
7750	G	0.919
7751	A	1.185
7752	U	0.799
7753	A	1.039
7754	A	0.786
7755	A	1.172
7756	U	0.719
7757	G	0.12
7758	G	0.04
7759	G	0.04
7760	C	0.08
7761	A	0.666
7762	A	1.305
7763	G	0.253
7764	U	0.226
7765	U	0.226
7766	U	0.173
7767	G	0.186
7768	U	0.639
7769	G	0.013
7770	G	0.04
7771	A	0.107
7772	A	0.067
7773	U	0.013
7774	U	0.107
7775	G	0.013
7776	G	0.04
7777	U	0.28
7778	U	0.773
7779	U	0.999

7780	A	1.132
7781	A	0.959
7782	C	0.373
7783	A	1.492
7784	U	0.666
7785	A	1.678
7786	A	0.999
7787	C	0.679
7788	A	1.225
7789	A	0.906
7790	A	1.518
7791	U	0.733
7792	U	0.147
7793	G	0.053
7794	G	0
7795	C	0.093
7796	U	0.067
7797	G	0.053
7798	U	0.013
7799	G	0.027
7800	G	0.12
7801	U	0.107
7802	A	0.586
7803	U	0.546
7804	A	3.09
7805	U	0.812
7806	A	1.185
7807	A	0.426
7808	A	0.679
7809	A	0.666
7810	U	0.373
7811	U	0.346
7812	A	0.599
7813	U	0.2
7814	U	0.373
7815	C	0.306
7816	A	1.265
7817	U	0.426
7818	A	0.999
7819	A	1.239
7820	U	0.306
7821	G	0.533
7822	A	0.773

7823	U	0.666
7824	A	0.799
7825	G	0.266
7826	U	0.679
7827	A	0.972
7828	G	0.466
7829	G	1.292
7830	A	0.693
7831	G	0.12
7832	G	0.293
7833	C	0.186
7834	U	0.799
7835	U	0.719
7836	G	0.186
7837	G	0.293
7838	U	0.653
7839	A	0.826
7840	G	0.12
7841	G	0.226
7842	U	0.133
7843	U	0.413
7844	U	1.105
7845	A	2.171
7846	A	1.279
7847	G	0.36
7848	A	0.466
7849	A	0.653
7850	U	0.866
7851	A	0.946
7852	G	0.626
7853	U	0.133
7854	U	0.293
7855	U	0.333
7856	U	1.119
7857	U	0.253
7858	G	0.28
7859	C	0.4
7860	U	0.093
7861	G	0.493
7862	U	0.293
7863	A	0.293
7864	C	0.053
7865	U	0.533

7866	U	0.546
7867	U	0.386
7868	C	0.053
7869	U	0.266
7870	A	0.413
7871	U	0.453
7872	A	1.145
7873	G	0.892
7874	U	0.186
7875	G	0.626
7876	A	0.679
7877	A	0.679
7878	U	0.919
7879	A	1.172
7880	G	1.105
7881	A	0.986
7882	G	0.226
7883	U	0.586
7884	U	1.319
7885	A	1.465
7886	G	0.413
7887	G	0.373
7888	C	0.746
7889	A	0.839
7890	G	0.679
7891	G	0.04
7892	G	0.027
7893	A	0.253
7894	U	0.16
7895	A	1.425
7896	U	0
7897	U	0.067
7898	C	0.107
7899	A	0.24
7900	C	0
7901	C	0.133
7902	A	0.373
7903	U	
7904	U	
7905	A	
7906	U	0.519
7907	C	0.253
7908	G	0.786

7909	U	0.32
7910	U	0.226
7911	U	0.346
7912	C	0.839
7913	A	1.039
7914	G	0.906
7915	A	0.892
7916	C	0
7917	C	0
7918	C	0.16
7919	A	0
7920	C	0.04
7921	C	0
7922	U	0.067
7923	C	0.013
7924	C	0.027
7925	C	0.253
7926	A	0.373
7927	A	0.24
7928	U	0
7929	C	0.506
7930	C	0
7931	C	2.531
7932	G	1.545
7933	A	1.172
7934	G	0.706
7935	G	0.04
7936	G	0.32
7937	G	0.559
7938	A	0
7939	C	0.559
7940	C	0
7941	C	0.733
7942	G	0.36
7943	A	0.839
7944	C	1.185
7945	A	0.08
7946	G	0
7947	G	0
7948	C	0
7949	C	0.4
7950	C	0.04
7951	G	0.373

7952	A	1.545
7953	A	1.438
7954	G	0.413
7955	G	0.186
7956	A	0.519
7957	A	0.679
7958	U	1.039
7959	A	1.199
7960	G	0.733
7961	A	1.585
7962	A	0.986
7963	G	1.132
7964	A	1.239
7965	A	0.413
7966	G	0.533
7967	A	1.625
7968	A	1.718
7969	G	0
7970	G	0.653
7971	U	0.546
7972	G	0
7973	G	0.906
7974	A	0.346
7975	G	1.092
7976	A	0.573
7977	G	1.465
7978	A	0.666
7979	G	1.039
7980	A	0.719
7981	G	0.759
7982	A	0.493
7983	C	0.653
7984	A	0.373
7985	G	1.105
7986	A	0.546
7987	G	0.453
7988	A	0.879
7989	C	1.558
7990	A	0.479
7991	G	0
7992	A	0
7993	U	0
7994	C	0

7995	C	0.013
7996	A	0.32
7997	U	0.226
7998	U	0.946
7999	C	0
8000	G	1.026
8001	A	0.493
8002	U	0.306
8003	U	0.866
8004	A	1.052
8005	G	1.185
8006	U	0.892
8007	G	0
8008	A	0.147
8009	A	0.852
8010	C	0.373
8011	G	0.04
8012	G	0.027
8013	A	0.253
8014	U	0
8015	C	0.093
8016	C	0.186
8017	U	0.706
8018	U	2.011
8019	A	2.318
8020	G	0.506
8021	C	0.306
8022	A	0.173
8023	C	0.027
8024	U	0.36
8025	U	0.293
8026	A	0.852
8027	U	0
8028	C	0.293
8029	U	0.799
8030	G	0.613
8031	G	0.12
8032	G	0.426
8033	A	1.212
8034	C	0.266
8035	G	0.546
8036	A	0.679
8037	U	0.453

8038	C	0.36
8039	U	1.252
8040	G	0.4
8041	C	0
8042	G	0.679
8043	G	1.385
8044	A	1.572
8045	G	0.226
8046	C	0.027
8047	C	0.28
8048	U	0.533
8049	G	0.626
8050	U	0.107
8051	G	0.08
8052	C	0
8053	C	0.013
8054	U	0
8055	C	0
8056	U	0.173
8057	U	0.346
8058	C	0.786
8059	A	0.919
8060	G	0.24
8061	C	0.027
8062	U	0.373
8063	A	0.986
8064	C	0.08
8065	C	0.24
8066	A	0.386
8067	C	0.666
8068	C	0.133
8069	G	0.306
8070	C	0.333
8071	U	0.36
8072	U	0.919
8073	G	0.426
8074	A	0.826
8075	G	0.213
8076	A	0.2
8077	G	0.12
8078	A	0.679
8079	C	0.2
8080	U	0.733

8081	U	0.626
8082	A	1.345
8083	C	0.04
8084	U	0.08
8085	C	0.573
8086	U	0.373
8087	U	0.333
8088	G	0.773
8089	A	0.892
8090	U	0.506
8091	U	0.466
8092	G	0.879
8093	U	0.546
8094	A	0.892
8095	A	0.919
8096	C	0.932
8097	G	0.972
8098	A	0.679
8099	G	0.173
8100	G	0.08
8101	A	0.799
8102	U	0.453
8103	U	0.773
8104	G	0.932
8105	U	0.186
8106	G	0.213
8107	G	0.266
8108	A	0.679
8109	A	0.626
8110	C	0.04
8111	U	0.133
8112	U	0.519
8113	C	0.546
8114	U	0.799
8115	G	0.027
8116	G	0
8117	G	0.44
8118	A	0.333
8119	C	0.08
8120	G	0.559
8121	C	0.919
8122	A	0.826
8123	G	0.226

8124	G	0.08
8125	G	0
8126	G	0.453
8127	G	0.426
8128	U	0.16
8129	G	0.053
8130	G	0.253
8131	G	0.986
8132	A	0.586
8133	A	0.693
8134	G	0.226
8135	C	0.12
8136	C	0.12
8137	C	0.213
8138	U	0.213
8139	C	0.4
8140	A	1.079
8141	A	0.733
8142	A	0.812
8143	U	0.506
8144	A	1.279
8145	U	0.852
8146	U	0.32
8147	G	0.773
8148	G	0.453
8149	U	0.04
8150	G	0.28
8151	G	0.226
8152	A	0.346
8153	A	0.519
8154	U	0.373
8155	C	0.16
8156	U	0.253
8157	C	0.013
8158	C	0
8159	U	0.173
8160	A	1.079
8161	C	0.346
8162	A	0.919
8163	G	0.666
8164	U	0.613
8165	A	1.319
8166	U	0.746

8167	U	0.839
8168	G	0.892
8169	G	0.719
8170	A	0.453
8171	G	0.04
8172	U	0.586
8173	C	0.386
8174	A	0.386
8175	G	0.666
8176	G	0.573
8177	A	0.959
8178	A	1.026
8179	C	0.413
8180	U	0.173
8181	A	0.679
8182	A	1.052
8183	A	1.252
8184	G	1.319
8185	A	0.932
8186	A	0.986
8187	U	1.292
8188	A	1.279
8189	G	0
8190	U	0.413
8191	G	0.413
8192	C	0.107
8193	U	0.093
8194	G	0.16
8195	U	0.293
8196	U	0.4
8197	A	1.026
8198	A	1.292
8199	C	1.159
8200	U	0
8201	U	0.293
8202	G	0.293
8203	C	0.426
8204	U	0.133
8205	C	0.373
8206	A	1.265
8207	A	1.012
8208	U	0.573
8209	G	0.599

8210	C	0
8211	C	0.373
8212	A	0
8213	C	0.04
8214	A	0.133
8215	G	0.08
8216	C	0.013
8217	C	0.293
8218	A	0
8219	U	0.466
8220	A	1.212
8221	G	1.359
8222	C	0.759
8223	A	1.052
8224	G	0.626
8225	U	0.333
8226	A	0.4
8227	G	0.693
8228	C	0.04
8229	U	0.147
8230	G	0.32
8231	A	0.44
8232	G	0.36
8233	G	0.186
8234	G	0.12
8235	G	0.546
8236	A	0.466
8237	C	0.733
8238	A	1.319
8239	G	1.279
8240	A	1.319
8241	U	0.519
8242	A	0.733
8243	G	0.133
8244	G	0.04
8245	G	0.213
8246	U	0.306
8247	U	0.546
8248	A	1.372
8249	U	0.666
8250	A	1.239
8251	G	0.852
8252	A	0.812

8253	A	0.679
8254	G	0.546
8255	U	0.466
8256	A	0.826
8257	U	0.506
8258	U	0.44
8259	A	0.639
8260	C	0.679
8261	A	0.559
8262	A	0.586
8263	G	0.213
8264	C	0.053
8265	A	0.266
8266	G	0.12
8267	C	0.053
8268	U	0.293
8269	U	0.986
8270	A	1.678
8271	U	0.733
8272	A	1.412
8273	G	1.239
8274	A	1.239
8275	G	0.013
8276	C	0
8277	U	0.32
8278	A	0.746
8279	U	0.426
8280	U	0.453
8281	C	0.333
8282	G	0.093
8283	C	0
8284	C	0.027
8285	A	0.253
8286	C	0.147
8287	A	1.159
8288	U	0.826
8289	A	0.706
8290	C	0
8291	C	0.013
8292	U	0.306
8293	A	0.879
8294	G	0.999
8295	A	0.986

8296	A	0.866
8297	G	1.079
8298	A	0.906
8299	A	0.879
8300	U	0.799
8301	A	1.172
8302	A	0.759
8303	G	0.826
8304	A	0.866
8305	C	0.519
8306	A	0.466
8307	G	0.28
8308	G	0
8309	G	0.133
8310	C	0.027
8311	U	0.36
8312	U	0.466
8313	G	0.626
8314	G	0.533
8315	A	0.879
8316	A	0.946
8317	A	0.959
8318	G	0.733
8319	G	0.586
8320	A	0.559
8321	U	0.107
8322	U	0.519
8323	U	0.706
8324	U	0.506
8325	G	0.346
8326	C	0.16
8327	U	0.386
8328	A	1.026
8329	U	0.639
8330	A	1.212
8331	A	0.892
8332	G	0.826
8333	A	0.746
8334	U	0.586
8335	G	0.24
8336	G	0.107
8337	G	0.08
8338	U	0.067

8339	G	0.093
8340	G	0.133
8341	C	0.28
8342	A	0.706
8343	A	0.852
8344	G	0.733
8345	U	0.253
8346	G	0.12
8347	G	0.16
8348	U	0.266
8349	C	0.693
8350	A	1.185
8351	A	1.132
8352	A	1.305
8353	A	0.959
8354	A	1.079
8355	G	1.079
8356	U	0.706
8357	A	0.719
8358	G	0.44
8359	U	0.333
8360	G	0.226
8361	U	0.173
8362	G	0.373
8363	A	0.253
8364	U	0.147
8365	U	1.066
8366	G	6.566
8367	G	1.252
8368	A	0.333
8369	U	0.12
8370	G	0.08
8371	G	0.053
8372	C	0
8373	C	0
8374	U	0.107
8375	G	0
8376	C	0.08
8377	U	0.147
8378	G	0.386
8379	U	0.586
8380	A	0.959
8381	A	0.999

8382	G	0.972
8383	G	0.653
8384	G	0.706
8385	A	0.839
8386	A	0.986
8387	A	1.105
8388	G	1.066
8389	A	1.079
8390	A	1.212
8391	U	0.866
8392	G	1.359
8393	A	1.052
8394	G	0.892
8395	A	1.105
8396	C	0.4
8397	G	1.172
8398	A	1.105
8399	G	0.027
8400	C	0
8401	U	0.186
8402	G	0.919
8403	A	0.879
8404	G	0.32
8405	C	0.027
8406	C	0.133
8407	A	0.266
8408	G	0.36
8409	C	0.186
8410	A	0.333
8411	G	0.186
8412	C	0.453
8413	A	0.746
8414	G	0.773
8415	A	1.066
8416	U	0.4
8417	G	0.346
8418	G	0.147
8419	G	0.053
8420	G	0.413
8421	U	0.213
8422	G	0.36
8423	G	0.4
8424	G	1.545

8425	A	0.466
8426	G	0.373
8427	C	0.506
8428	A	0.786
8429	G	0.719
8430	U	0.666
8431	A	1.039
8432	U	0.173
8433	C	0.067
8434	U	0.173
8435	C	0.266
8436	G	1.359
8437	A	2.264
8438	G	0.639
8439	A	0.426
8440	C	0.053
8441	C	0.12
8442	U	0.719
8443	A	1.758
8444	G	1.398
8445	A	1.345
8446	A	1.039
8447	A	0.986
8448	A	1.012
8449	A	0.959
8450	C	0.852
8451	A	0.999
8452	U	0.479
8453	G	0.852
8454	G	0.839
8455	A	0.706
8456	G	0.626
8457	C	0.533
8458	A	0.719
8459	A	1.026
8460	U	0.546
8461	C	0.333
8462	A	0.386
8463	C	0.573
8464	A	0.493
8465	A	0.4
8466	G	0.4
8467	U	0.346

8468	A	0.346
8469	G	0.173
8470	C	0.666
8471	A	0.586
8472	A	1.398
8473	U	0.959
8474	A	0.866
8475	C	0.559
8476	A	1.185
8477	G	0.053
8478	C	0.226
8479	A	0.506
8480	G	0.373
8481	C	0.226
8482	U	0.906
8483	A	1.319
8484	A	1.145
8485	C	0.919
8486	A	0.719
8487	A	0.999
8488	U	0.426
8489	G	0.013
8490	C	0.04
8491	U	0.32
8492	G	0.4
8493	C	1.971
8494	U	0.186
8495	U	0.293
8496	G	0.16
8497	U	0.12
8498	G	0.186
8499	C	0.173
8500	C	0.2
8501	U	0.839
8502	G	0.559
8503	G	0.253
8504	C	0.16
8505	U	0.972
8506	A	1.771
8507	G	1.665
8508	A	1.412
8509	A	0.826
8510	G	0.426

8511	C	0.639
8512	A	0.613
8513	C	1.372
8514	A	1.092
8515	A	0.986
8516	G	0.866
8517	A	0.799
8518	G	0.306
8519	G	0.812
8520	A	0.639
8521	G	0.546
8522	G	1.279
8523	A	0.946
8524	A	1.145
8525	G	0.666
8526	A	0.839
8527	G	0.147
8528	G	0.2
8529	U	0.666
8530	G	0.32
8531	G	0.44
8532	G	0.839
8533	U	0
8534	U	0
8535	U	0.12
8536	U	0.213
8537	C	0
8538	C	0
8539	A	0.346
8540	G	0.24
8541	U	0.12
8542	C	0.306
8543	A	0.559
8544	C	0.213
8545	A	0.293
8546	C	0.186
8547	C	0.253
8548	U	0.36
8549	C	0.599
8550	A	0.546
8551	G	0.373
8552	G	0.506
8553	U	0.306

8554	A	0.373
8555	C	0.107
8556	C	0.133
8557	U	0.306
8558	U	0.719
8559	U	1.305
8560	A	0.972
8561	A	0.799
8562	G	0.666
8563	A	0.626
8564	C	0.253
8565	C	0.839
8566	A	0.959
8567	A	0.866
8568	U	0.733
8569	G	0.932
8570	A	0.759
8571	C	0.12
8572	U	0.613
8573	U	0.919
8574	A	1.105
8575	C	1.545
8576	A	0.639
8577	A	0.533
8578	G	0.2
8579	G	0.053
8580	C	0.373
8581	A	0.266
8582	G	0.36
8583	C	0.093
8584	U	0.067
8585	G	0.32
8586	U	0.733
8587	A	0.906
8588	G	0.679
8589	A	0.786
8590	U	0.2
8591	C	0.226
8592	U	0.639
8593	U	1.319
8594	A	1.825
8595	G	0.346
8596	C	0.04

8597	C	0.28
8598	A	0.746
8599	C	1.039
8600	U	1.412
8601	U	0.44
8602	U	0.826
8603	U	0.919
8604	U	1.731
8605	A	1.665
8606	A	0.812
8607	A	1.092
8608	A	0.972
8609	G	1.012
8610	A	0.693
8611	A	0.613
8612	A	1.172
8613	A	0.333
8614	G	0.173
8615	G	0.133
8616	G	0.213
8617	G	0.226
8618	G	0.453
8619	G	0.293
8620	A	0.373
8621	C	0.12
8622	U	0.719
8623	G	0.346
8624	G	0.293
8625	A	0.693
8626	A	0.453
8627	G	0.293
8628	G	0.226
8629	G	0.213
8630	C	0.013
8631	U	0.533
8632	A	1.998
8633	A	1.665
8634	U	0.226
8635	U	0.306
8636	C	0.186
8637	A	0.2
8638	C	0.053
8639	U	0.093

8640	C	0.053
8641	C	0.067
8642	C	0
8643	A	1.119
8644	A	1.092
8645	A	0.826
8646	G	0.799
8647	A	0.932
8648	A	0.693
8649	G	0.479
8650	A	0.639
8651	C	0.013
8652	A	0.4
8653	A	0.173
8654	G	0.306
8655	A	1.039
8656	U	0.373
8657	A	0.906
8658	U	0.16
8659	C	0.173
8660	C	0.013
8661	U	0.16
8662	U	0.186
8663	G	0.373
8664	A	1.745
8665	U	0.306
8666	C	0.093
8667	U	0.266
8668	G	0.466
8669	U	0.053
8670	G	0
8671	G	0.12
8672	A	0.932
8673	U	3.383
8674	C	0.12
8675	U	0.413
8676	A	1.265
8677	C	0
8678	C	0.027
8679	A	0.08
8680	C	0.053
8681	A	0.493
8682	C	0

8683	A	0.493
8684	C	0.027
8685	A	0.946
8686	A	0.719
8687	G	0.147
8688	G	0
8689	C	0.08
8690	U	0.506
8691	A	0.373
8692	C	0.107
8693	U	0.573
8694	U	0.306
8695	C	0.093
8696	C	0.027
8697	C	0.12
8698	U	0.213
8699	G	0.599
8700	A	0.466
8701	U	0.333
8702	U	0.173
8703	G	0.067
8704	G	0.08
8705	C	0.04
8706	A	0.426
8707	G	0.733
8708	A	0.573
8709	A	0.373
8710	C	0.053
8711	U	0.453
8712	A	0.599
8713	C	0.08
8714	A	0.812
8715	C	0.107
8716	A	0.386
8717	C	0.093
8718	C	0.306
8719	A	0.932
8720	G	1.412
8721	G	0.493
8722	G	0.253
8723	C	0.04
8724	C	0.08
8725	A	0.226

8726	G	0.027
8727	G	0
8728	G	0
8729	G	0.053
8730	U	0.013
8731	C	0.213
8732	A	0.067
8733	G	0.107
8734	A	0.932
8735	U	0.586
8736	A	1.199
8737	U	0.28
8738	C	0.08
8739	C	0.2
8740	A	0.519
8741	C	0.067
8742	U	0
8743	G	0.027
8744	A	0
8745	C	0
8746	C	0.04
8747	U	0.08
8748	U	0.293
8749	U	0.2
8750	G	0.186
8751	G	0.306
8752	A	1.239
8753	U	0.24
8754	G	0.027
8755	G	0.053
8756	U	0
8757	G	0
8758	C	0.04
8759	U	0.053
8760	A	0.133
8761	C	0.253
8762	A	1.398
8763	A	0.679
8764	G	0.186
8765	C	0.519
8766	U	0.12
8767	A	0.08
8768	G	0.147

8769	U	0.067
8770	A	0.067
8771	C	0.067
8772	C	0.093
8773	A	0.666
8774	G	0.479
8775	U	0.586
8776	U	0.386
8777	G	1.558
8778	A	0.706
8779	G	0.373
8780	C	0
8781	C	0.053
8782	A	0.586
8783	G	0.786
8784	A	1.105
8785	U	0.639
8786	A	0.906
8787	A	0.719
8788	G	0.28
8789	G	0.107
8790	U	0.226
8791	A	0.413
8792	G	0.333
8793	A	0.879
8794	A	0.759
8795	G	1.092
8796	A	0.666
8797	G	0.493
8798	G	0.067
8799	C	0
8800	C	0.067
8801	A	0.28
8802	A	0.466
8803	U	0.599
8804	A	1.851
8805	A	1.678
8806	A	0.773
8807	G	0.08
8808	G	0.173
8809	A	0.386
8810	G	0.453
8811	A	0.506

8812	G	0.426
8813	A	0.733
8814	A	0.506
8815	C	0.266
8816	A	0.586
8817	C	0.08
8818	C	0.147
8819	A	0.28
8820	G	0.2
8821	C	0.093
8822	U	0.599
8823	U	0.24
8824	G	0.373
8825	U	0.226
8826	U	0.493
8827	A	0.826
8828	C	0.2
8829	A	0.373
8830	C	0.04
8831	C	0.013
8832	C	0.04
8833	U	0.32
8834	G	0.44
8835	U	0.306
8836	G	0.906
8837	A	0.679
8838	G	0.253
8839	C	0.04
8840	C	0.093
8841	U	0.226
8842	G	0.573
8843	C	0.093
8844	A	0.639
8845	U	0.28
8846	G	0.36
8847	G	0.36
8848	A	0.559
8849	A	0.679
8850	U	0.213
8851	G	0.306
8852	G	0.293
8853	A	0.453
8854	U	0.186

8855	G	0.373
8856	A	0.36
8857	C	0.053
8858	C	0.053
8859	C	0.12
8860	U	0.36
8861	G	0.733
8862	A	1.066
8863	G	0.613
8864	A	1.012
8865	G	0.333
8866	A	1.012
8867	G	0.226
8868	A	0.053
8869	A	0.133
8870	G	0.147
8871	U	0.08
8872	G	0.08
8873	U	0.173
8874	U	0.24
8875	A	0.253
8876	G	0.107
8877	A	1.199
8878	G	0.173
8879	U	0.013
8880	G	0.08
8881	G	0.107
8882	A	0.213
8883	G	0.226
8884	G	0.147
8885	U	0.999
8886	U	1.319
8887	U	0.546
8888	G	0.839
8889	A	0.946
8890	C	0.466
8891	A	1.012
8892	G	0.186
8893	C	0.053
8894	C	0.04
8895	G	0.067
8896	C	0
8897	C	0.067

8898	U	0.186
8899	A	0.186
8900	G	0.12
8901	C	0.147
8902	A	0.147
8903	U	0.013
8904	U	0.093
8905	U	0.133
8906	C	0.24
8907	A	1.185
8908	U	0.28
8909	C	1.545
8910	A	0.799
8911	C	0.293
8912	G	0.466
8913	U	0.16
8914	G	0.08
8915	G	0.013
8916	C	0.12
8917	C	0.12
8918	C	0.346
8919	G	0.906
8920	A	0.626
8921	G	0.466
8922	A	0.812
8923	G	0.12
8924	C	0.053
8925	U	0.24
8926	G	0.32
8927	C	0.213
8928	A	0.626
8929	U	0.453
8930	C	0.386
8931	C	0.16
8932	G	0.373
8933	G	0.759
8934	A	0.506
8935	G	0.453
8936	U	1.185
8937	A	0.706
8938	C	0.24
8939	U	0.36
8940	U	0.493

8941	C	1.319
8942	A	1.225
8943	A	1.039
8944	G	0.959
8945	A	0.986
8946	A	0.573
8947	C	0.213
8948	U	0.24
8949	G	0.16
8950	C	0.08
8951	U	0.226
8952	G	0.4
8953	A	0.573
8954	C	2.704
8955	A	1.052
8956	U	0.666
8957	C	0.466
8958	G	0.852
8959	A	0.786
8960	G	0.107
8961	C	0.12
8962	U	0.36
8963	U	0.32
8964	G	0.173
8965	C	0.2
8966	U	3.13
8967	A	1.012
8968	C	3.37
8969	A	1.558
8970	A	0.866
8971	G	0.333
8972	G	0.186
8973	G	0.32
8974	A	0.786
8975	C	1.092
8976	U	0.333
8977	U	1.305
8978	U	1.105
8979	C	0.799
8980	C	0.346
8981	G	0.36
8982	C	0.067
8983	U	0.107

8984	G	0.053
8985	G	0.013
8986	G	0.013
8987	G	0.4
8988	A	0.693
8989	C	0.213
8990	U	0.426
8991	U	0.666
8992	U	0.466
8993	C	0.133
8994	C	0.04
8995	A	1.039
8996	G	0.519
8997	G	0.44
8998	G	0.413
8999	A	0.626
9000	G	1.145
9001	G	0.479
9002	C	0.479
9003	G	0.4
9004	U	0.479
9005	G	0.28
9006	G	0.573
9007	C	0.147
9008	C	0.173
9009	U	0.08
9010	G	0.16
9011	G	0.12
9012	G	0.293
9013	C	0.16
9014	G	0.186
9015	G	0.586
9016	G	0.706
9017	A	0.826
9018	C	0.559
9019	U	0.639
9020	G	0.519
9021	G	0
9022	G	0.892
9023	G	0.946
9024	A	0.253
9025	G	0.067
9026	U	0.266

9027	G	0.12
9028	G	0.773
9029	C	0.2
9030	G	0.586
9031	A	0.32
9032	G	0.12
9033	C	0.013
9034	C	0.027
9035	C	0
9036	U	0.027
9037	C	0.786
9038	A	6.46
9039	G	1.305
9040	A	0.519
9041	U	1.305
9042	G	0.879
9043	C	0.04
9044	U	0.08
9045	G	0.107
9046	C	1.239
9047	A	1.811
9048	U	6.313
9049	A	1.971
9050	U	10.735
9051	A	2.131
9052	A	0.946
9053	G	0.053
9054	C	0.626
9055	A	0.16
9056	G	0.04
9057	C	0.08
9058	U	0.466
9059	G	0.173
9060	C	0.413
9061	U	0.44
9062	U	0.333
9063	U	1.039
9064	U	0.733
9065	U	0.599
9066	G	0.027
9067	C	0.466
9068	C	0.613
9069	U	0.266

9070	G	0.573
9071	U	14.971
9072	A	2.198
9073	C	0.36
9074	U	0.773
9075	G	0.093
9076	G	0
9077	G	0
9078	U	0.093
9079	C	0.506
9080	U	0.2
9081	C	0.013
9082	U	0
9083	C	0.04
9084	U	0.04
9085	G	0.013
9086	G	0
9087	U	0.027
9088	U	0.04
9089	A	0.013
9090	G	0.04
9091	A	1.851
9092	C	0.253
9093	C	0.027
9094	A	0.013
9095	G	0
9096	A	0.719
9097	U	1.465
9098	C	2.824
9099	U	0.773
9100	G	0
9101	A	0
9102	G	0
9103	C	0.013
9104	C	0.346
9105	U	1.412
9106	G	1.292
9107	G	0.746
9108	G	0.2
9109	A	1.505
9110	G	0.147
9111	C	0
9112	U	0

9113	C	0
9114	U	0.147
9115	C	0
9116	U	0
9117	G	0
9118	G	0.027
9119	C	0
9120	U	0.067
9121	A	0
9122	A	0
9123	C	0.013
9124	U	0
9125	A	0.013
9126	G	0.067
9127	G	0
9128	G	0
9129	A	0.027
9130	A	0
9131	C	0.093
9132	C	0
9133	C	0
9134	A	0
9135	C	0.053
9136	U	0.08
9137	G	0.16
9138	C	0
9139	U	0.213
9140	U	0.693
9141	A	0.093
9142	A	0.08
9143	G	
9144	C	
9145	C	
9146	U	
9147	C	
9148	A	
9149	A	
9150	U	
9151	A	
9152	A	
9153	A	
9154	G	
9155	C	

9156	U
9157	U
9158	G
9159	C
9160	C
9161	U
9162	U
9163	G
9164	A
9165	G
9166	U
9167	G
9168	C
9169	U
9170	U
9171	C
9172	A
9173	A
