# Supplementary material for: Comparison of SIV and HIV-1 Genomic RNA Structures Reveals Impact of Sequence Evolution on Conserved and Non-Conserved Structural Motifs
Source: PLoS Pathog. 2013 Apr 4;9(4):e1003294. doi: 10.1371/journal.ppat.1003294 (PMC3616985; doi:10.1371/journal.ppat.1003294)
Supplement: Table S1 — Sequences of primers used for SHAPE. (PDF) [file ppat.1003294.s009.pdf]

Table S1 Sequences of primers used for SHAPE

Name	Primer Sequence
SIV309	TCCTTCAAGTCCCTGTTCAGGC
SIV443	AACCGGAGGCCTCTTCCTCTCC
SIV593	CTTTCCGTTGGGTCGTAGCCT
SIV897	GATGGTGCTGTTGGTCTACTTG
SIV1193	GTCCTTGTTGTGGAGCTGGTTG
SIV1475	GTTTGAGTCATCCAATTCTTTAC
SIV1761	TCCAGCATCCCTGTCTTCTTG
SIV2095	CTGTATCCAGTAATACTTCTAC
SIV2138	GTGGACCTAACTCTATTCCTG
SIV2386	GCCACTGCTTCAATTTTGGTCC
SIV2674	CTAGAGGTATGGAGAAATATGC
SIV2952	CCCTATGCTATTCAAGAGTTCC
SIV3225	CTCATATTCTGCTTCTGCCATC
SIV3505	CCCATACATCCTTCTCAACTGG
SIV3780	CCCTGAGTCTGTCAATGCCATG
SIV4027	CATGTTCTTCTTGTGCTGGCTC
SIV4289	AATAGTGCTGTCTGTCTTCCTG
SIV4576	GAGTCATATCCCCTATTCCTCC
SIV4831	AACTGCTATCCACCTCTTTTCC
SIV5112	TAGTTTGGTGTTACATCTGTCC
SIV5382	CCGCCTCTCTGTTTATCTCCTC
SIV5647	TGTGGTCCTTCATTTTCTGGAG
SIV5904	TAGAGGGCGGTATAGCTGAGAG
SIV6184	ATTGTCGCATTCCTCCAAGCTG
SIV6350	CTCAAAGAGTTGCCATACATCC
SIV6635	TGCAGATGACCAAGTTTCATTG
SIV6894	AGCCAAACCAAGTAGAAGTCTG
SIV7170	CAGTATACCTGGGATGTTTGAC
SIV7462	AGACTGGTCACTGTGGAGTTAC
SIV7745	CCCAGCCAATAAAGTTCGGGAC
SIV8001	AGTCAACCTTTCGCTCCCACTC
SIV8261	GAAATAAGAGGGTGGGGAAGAG
SIV8536	TGTAGGTAGGTCAGTTCAGTCC
SIV8830	CCAAGTCATCATCTTACTCATC
SIV9107	TCATCCTCCTGTGCCTCATCTG
SIV9282	TAGCCTTCTTCTAACCTCTTCC
SIV9485	GAACCTCCCAGGGCTCAATCTG
SIV9621	TTTTTACTTCTAAAATGGCAGC

Numbers indicate the 5' position in the SIVmac239 genome to which each anneals.
