# Supplementary material for: Comparison of SIV and HIV-1 Genomic RNA Structures Reveals Impact of Sequence Evolution on Conserved and Non-Conserved Structural Motifs
Source: PLoS Pathog. 2013 Apr 4;9(4):e1003294. doi: 10.1371/journal.ppat.1003294 (PMC3616985; doi:10.1371/journal.ppat.1003294)
Supplement: Table S2 — Start and end points corresponding to regions in the 75-nt moving window of median SIVmac239 SHAPE reactivities with values lower than 0.3 (from Figure 2B). (PDF) [file ppat.1003294.s010.pdf]

Table S2 Start and end points corresponding to regions in the 75-nt moving window of median SIVmac239 SHAPE reactivities with values lower than 0.3 (from Figure 2B).

Region	Start	End	Nucleotides in Structure	GC	AU	GU
1*	1	488	1-539	99	60	16
2	720	804				
3#	2091	2193	2098-2187	11	14	4
4#	2434	2548	2462-2497	9	7	0
5#	2616	2871	2641-2895	32	33	10
6#	2969	3044	2996-3038	6	3	1
7#	3254	3361	3285-3323	5	5	0
8#	4679	4756	4679-4726	8	3	4
9#	4819	4914	4862-4907	5	6	1
10#	4926	5000	4976-4988; 5220-5232	7	4	2
11#	5409	5522	5392-5527	54	35	8
12#	5818	5987	5786-5946			
13#	7095	7236	7166-7223	6	8	2
14#	8200	8332	8233-8359	28	14	7
15	9433	9655				
G-P-P FS*	1793	1974	1793-1879	17	6	1
RRE*	7617	7853	7601-7909	47	25	13

GC: G-C base pair; AU: A-U base pair; GU: G-U base pair; G-P-P FS: Gag-Pro-Pol frameshift; RRE: Rev response element. Asterisks (*) indicate structures of known function and pound signs (#) indicate structures of unknown function used for GC versus AU/GU and G versus A comparisons. For the GC versus AU/GU analysis, the valley at position 10, which corresponds to the 5' side of the longest continuous helix, was included along with the paired nucleotides on the 3' side. Nucleotides corresponding to valleys 11 and 12 were also taken together since they spanned both sides of the resultant stems. In all cases, we included 37 nucleotides before and after each region to include all of the nucleotides that are included in the 75-nt window.
